# Propionate reinforces epithelial identity and reduces aggressiveness of lung carcinoma

**DOI:** 10.15252/emmm.202317836

**Published:** 2023-09-28

**Authors:** Vignesh Ramesh, Paradesi Naidu Gollavilli, Luisa Pinna, Mohammad Aarif Siddiqui, Adriana Martinez Turtos, Francesca Napoli, Yasmin Antonelli, Aldo Leal‐Egaña, Jesper Foged Havelund, Simon Toftholm Jakobsen, Elisa Le Boiteux, Marco Volante, Nils Joakim Færgeman, Ole N Jensen, Rasmus Siersbæk, Kumar Somyajit, Paolo Ceppi

**Affiliations:** ^1^ Department of Biochemistry and Molecular Biology University of Southern Denmark Odense Denmark; ^2^ Interdisciplinary Centre for Clinical Research University Hospital Erlangen, FAU‐Erlangen‐Nuremberg Erlangen Germany; ^3^ Department of Oncology at San Luigi Hospital University of Turin Turin Italy; ^4^ Institute for Molecular Systems Engineering and Advanced Materials Heidelberg University Heidelberg Germany

**Keywords:** epithelial–mesenchymal transition, H3K27 acetylation, metastasis, metabolic inhibitor, propionate, Cancer, Metabolism, Respiratory System

## Abstract

The epithelial‐to‐mesenchymal transition (EMT) plays a central role in the development of cancer metastasis and resistance to chemotherapy. However, its pharmacological treatment remains challenging. Here, we used an EMT‐focused integrative functional genomic approach and identified an inverse association between short‐chain fatty acids (propionate and butanoate) and EMT in non‐small cell lung cancer (NSCLC) patients. Remarkably, treatment with propionate *in vitro* reinforced the epithelial transcriptional program promoting cell‐to‐cell contact and cell adhesion, while reducing the aggressive and chemo‐resistant EMT phenotype in lung cancer cell lines. Propionate treatment also decreased the metastatic potential and limited lymph node spread in both nude mice and a genetic NSCLC mouse model. Further analysis revealed that chromatin remodeling through H3K27 acetylation (mediated by p300) is the mechanism underlying the shift toward an epithelial state upon propionate treatment. The results suggest that propionate administration has therapeutic potential in reducing NSCLC aggressiveness and warrants further clinical testing.

## Graphical abstract

An EMT‐centric investigation of metabolic processes in a comprehensive lung cancer transcriptome profiles collection identified an inverse association between EMT and SCFAs propionate and butyrate.

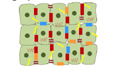

**The paper explained**

**Problem**

Epithelial‐to‐mesenchymal transition (EMT), a phenotypic developmental process, contributes to various highly aggressive features in cancer, including metastasis and chemoresistance with poor patient survival. However, pharmacological targeting of EMT in malignancies has proven very challenging. In recent years, targeting deregulated metabolic processes in cancer emerged as a realistic therapeutic strategy, though identification of precise EMT metabolic processes remains difficult.

**Results**

Taking advantage of large numbers of non‐small cell lung cancer (NSCLC) patient‐derived gene expression profiles and of numerous metabolic processes gene‐sets, we performed an EMT‐focused functional genomic analysis and identified negative association between EMT and short‐chain fatty acids, especially propionate. Strikingly, treatment of lung cancer cell lines with propionate reinforced the epithelial transcriptional program, promoting cell–cell contact features along with the inhibition of aggressive EMT process, and sensitized the cells to cisplatin treatment. In addition, propionate reduced metastasis ability in a lung experimental metastasis model and limited lymph node metastatic spread in a genetic NSCLC mouse model. Finally, propionate was involved in chromatin remodeling with increased histone acetylation.

**Impact**

In this study, we identify in pre‐clinical models the therapeutic relevance of the short‐chain fatty acid propionate in reducing EMT‐induced aggressive features of lung cancer.

## Introduction

Lung cancer is the second most frequently diagnosed cancer and a leading cause of cancer deaths with non‐small‐cell lung cancer (NSCLC) accounting for 85% of cases (Herbst *et al*, 2018; Sung *et al*, 2021). Development of next‐generation molecular targeted therapeutics and immunotherapy for lung cancer have shown improvements in the treatment of advanced patients in recent years (Herbst *et al*, 2020; Ramalingam *et al*, 2020). However, NSCLC prognosis remains poor with a 5‐year overall survival rate of 25% (Caini *et al*, 2022).

Epithelial‐to‐mesenchymal transition (EMT) is a developmental phenotypic plasticity program, which in cancer confers aggressive features including migration, chemoresistance, and metastatic colonization, leading to poor patient survival (Tan *et al*, 2014; Brabletz *et al*, 2021). In lung, the fundamental importance of the EMT process in the early tumorigenic steps has also been highlighted (Sato *et al*, 2013; Vaz *et al*, 2017), and embryonic transcription factors (TFs) like ZEB1, TWIST, and SNAIL have been clearly functionally connected to EMT promotion and maintenance (Brabletz *et al*, 2021). However, targeting EMT‐associated TFs with drugs remains an unresolved clinical issue (Dang *et al*, 2017). Apart from the TFs, microRNAs (Gollavilli *et al*, 2021) and more recently metabolic reprogramming have been shown as crucial hallmarks of EMT regulation in several cancer types (Colvin *et al*, 2016; Schwab *et al*, 2018). Numerous metabolic pathway inhibitors have been identified in preclinical studies, some of which with a potential for EMT targeting *in vivo* (Ramesh *et al*, 2020). However, many of these inhibitors face limitations including poor uptake or high toxicity, resulting in low clinical therapeutic efficacy (Sun & Yang, 2020; Lemberg *et al*, 2022). An additional pitfall is represented by our limited understanding of the complex metabolic alterations occurring during the advanced stages of tumorigenesis (Sun & Yang, 2020). This emphasizes the need for a comprehensive characterization of cellular, molecular, and functional level processes associated with the metabolic control of EMT to improve drug targeting.

Assessing EMT state in patient‐derived samples is technically difficult due to its dynamic nature (Vasaikar *et al*, 2021) and a similar level of technical limitations also exists in detecting and quantifying the global altered metabolic state in tumors (Han *et al*, 2021; Rohatgi *et al*, 2022). Nevertheless, transcriptomic approaches can effectively portrait the metabolic reprogramming in cancer as the metabolic enzyme regulation also occurs at the transcriptional level (Leeuwenburgh *et al*, 2021; Rohatgi *et al*, 2022). On this basis, the present study conducted a comprehensive lung cancer transcriptome analysis in the context of EMT‐associated metabolic processes. Further, it investigated the mechanistic role of propionate as a metabolite coupled epigenetic modifier in preventing aggressive EMT processes including metastasis, by strengthening lung epithelial identity.

## Results

### Short‐chain fatty acids show negative association with EMT in lung cancer

**Figure 1:**
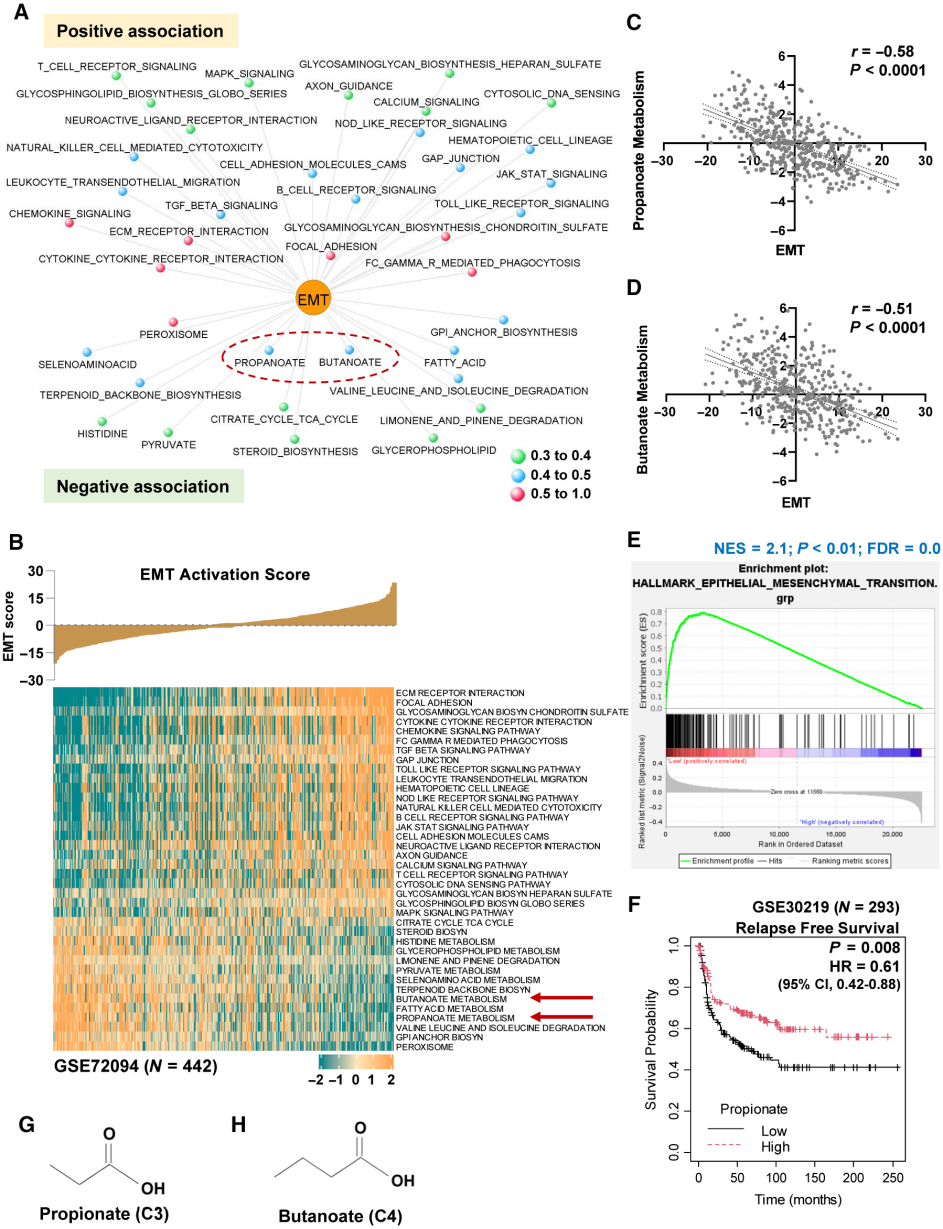
Propionate and butanoate are negatively associated with EMT in lung cancer gene expression profiles identified from integrative functional genomic analysis ANetwork visualization of positively and negatively associated metabolic processes with EMT inferred from the meta‐correlation analysis of the activation scores of the gene‐sets in lung cancer gene expression profiles (*N* = 1,476) from six datasets. meta‐*r* < −0.3 and meta‐*r* > 0.3; meta‐*P* < 0.05.BRepresentative heatmap visualization of significant positively and negatively associated metabolic processes activation with the increasing activation levels of EMT gene signature as a bar plot in GSE72094 (*N* = 442) above heatmap. Red arrows indicate propionate and butanoate metabolisms negatively associated with EMT activation.C, DCorrelation plot between the metabolic process activation scores of short‐chain fatty acids (propionate (C) or butanoate (D)) with EMT gene signature activation in GSE72094 profile (*N* = 442).EGene set enrichment analysis of hallmark EMT gene‐set with the lung cancer patient samples (GSE72094; *N* = 442) categorized as low and high based on the propionate gene‐set activation levels showed EMT enrichment in low propionate patient samples. Ranking of genes with signal2noise metric was used for GSEA.FRelapse free survival analysis in lung cancer patient samples (GSE30219; *N* = 293) categorized as low‐ and high‐propionate levels based on the median showed good prognosis for propionate gene‐set. HR – Hazard ratio for high propionate group was calculated using Cox proportional hazards model. *P*‐value was calculated using log‐rank method.G, HStructure of short‐chain fatty acids, propionate (G) and butanoate (H). Source data are available online for this figure.

Toward exploration of inherent metabolic processes that could inhibit EMT, an integrative functional genomic analysis was performed in a comprehensive collection of patient‐derived lung cancer gene expression profiles from six different datasets (*N* = 1,476) (Appendix Table S1) (Data ref: Lee *et al*, 2008; Data ref: Okayama *et al*, 2012; Data ref: Botling *et al*, 2013; Data ref: Rousseaux *et al*, 2013; Data ref: Der *et al*, 2014; Data ref: Schabath *et al*, 2016). First, a robust pan‐cancer derived EMT gene signature was obtained (Mak *et al*, 2016) (Appendix Table S2), and its overall expression pattern with increased mesenchymal and decreased epithelial genes expression in NSCLC cell line expression profiles (A549, NCI‐H358 and HCC827) treated with TGF‐β1 was confirmed (Appendix Fig S1A–C). Second, 335 metabolic oriented gene‐sets representing diverse metabolic processes were extracted from MSigDB (Appendix Table S3). Third, pan‐cancer EMT gene signature and metabolic process gene‐sets activation pattern in lung cancer gene expression profiles was computed using *z*‐score based approach. Finally, an EMT‐centered investigation, with a clinically accepted meta‐correlation (meta‐*r* < −0.3 and meta‐*r* > 0.3; meta‐*P* value < 0.05), has identified several positively and negatively associated metabolic processes (Fig 1A, Appendix Tables S4 and S5) including previously reported chondroitin sulphate (Chang *et al*, 2022) and heparan (Zhang *et al*, 2018) biosynthetic processes. Interestingly, EMT was found negatively correlated with short‐chain fatty acids (SCFAs), propionate (meta‐*r* = −0.46) and butanoate (meta‐*r* = −0.46) (Appendix Table S4) which have not been investigated in detail previously in the context of lung cancer mediated EMT process. A representative lung cancer dataset (GSE72094; *N* = 442) clearly showed the inverse association between EMT and SCFAs by heatmap and correlation plots (Fig 1B–D). Notably, there was no overlapping of gene content between EMT genes and propionate (or butanoate) gene‐sets (Appendix Fig S1D and E). A similar negative trend between SCFAs and EMT was further validated by independent approaches: i) in The Cancer Genome Atlas (TCGA) lung adenocarcinoma patients (*N* = 510) sequenced using RNA‐seq (Appendix Fig S1F and G), ii) with different source of EMT gene signature from MSigDB (Appendix Fig S1H and I), and iii) with Gene Set Enrichment Analysis (GSEA) of hallmark EMT gene signature significantly enriched in low‐propionate or low‐butanoate categorized lung cancer patients (Fig 1E and Appendix Fig S1J). Subsequently, a good prognosis in lung cancer patients was observed with high‐propionate or high‐butanoate gene‐set expression (Fig 1F and Appendix Fig S1K). These short‐chain fatty acids propionate and butanoate are 3‐carbon and 4‐carbon compounds, respectively (Fig 1G and H).

### Propionate and butanoate, but not acetate, inhibit EMT

**Figure 2:**
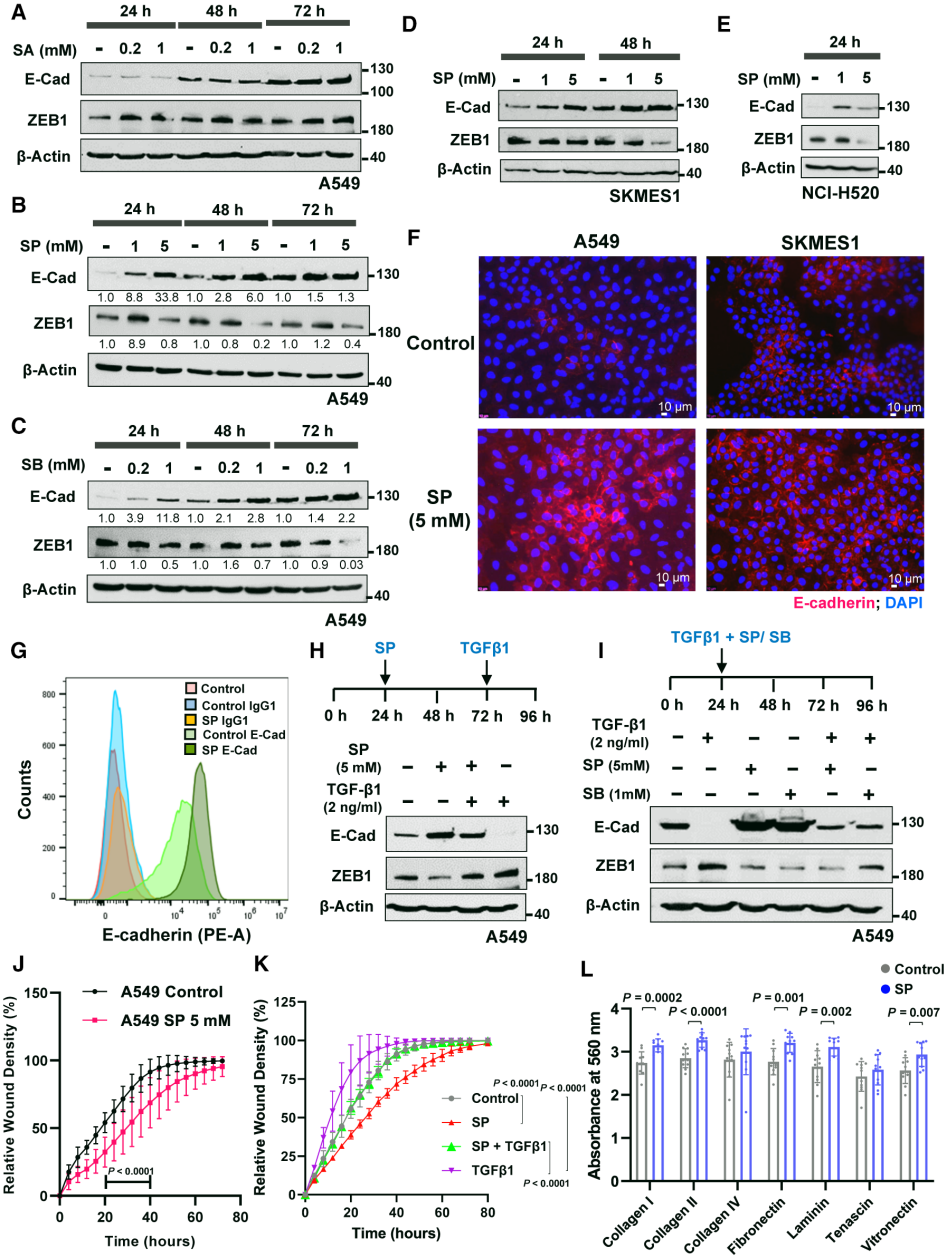
Propionate and butanoate modulates the expression of EMT markers with enhanced E‐cadherin expression A–CWestern blot analysis of E‐cadherin and ZEB1 protein levels in A549 cells treated with sodium acetate (SA) (A), sodium propionate (SP) (B) or sodium butanoate (SB) (C) in the indicated dose‐ and time‐dependent manner. β‐Actin was used as an internal control. Numbers indicate the fold change compared to the control of the respective time points.D, EWestern blot analysis of E‐cadherin and ZEB1 in NSCLC cell lines SKMES1 (D) and NCI‐H520 (E) treated with sodium propionate (SP) in the indicated dose and time points. β‐Actin was used as an internal control.FImmunofluorescence staining of E‐cadherin in NSCLC cell lines, A549 and SKMES1, treated with sodium propionate (5 mM) for 3 days. DAPI was used as a nuclear stain. Scale bars: 10 μm.GFlow cytometry analysis of membrane‐associated PE‐conjugated E‐cadherin in A549 cells treated with sodium propionate (SP) at 5 mM concentration for 48 h. PE‐conjugated IgG1 was used as a control for flow cytometry.HWestern blot analysis of E‐cadherin and ZEB1 protein levels in A549 cells pre‐treated with sodium propionate (SP) for 48 h followed by TGF‐β1 for 24 h. β‐Actin was used as an internal control.IWestern blot analysis of E‐cadherin and ZEB1 protein levels in A549 cells co‐treated with sodium propionate (SP) or sodium butanoate (SB) in combination with TGF‐β1 for 72 h. β‐Actin was used as an internal control.JLine plot depicts the rate of wound healing, expressed as relative wound density in A549 cells treated with sodium propionate (SP) (*n* = 10) over the course of 72 h from making the wound for migration assay.KLine plot depicts the rate of wound healing, expressed as relative wound density in A549 cells treated with sodium propionate (SP, 5 mM) for 48 h followed by TGF‐β1 (2 ng/ml) for 24 h from making the wound for migration assay. *P*‐value was calculated from two‐way ANOVA followed by Tukey's multiple comparison test.LCell adhesion assay in A549 cells treated with sodium propionate (5 mM) for 72 h. Data points (*n* = 8) are represented as mean ± SD and *P*‐value was calculated from *t*‐test. Data information: All results are representative data of three independent experiments. Data are represented as mean ± SD (J–L). *P*‐value was calculated from two‐way ANOVA (J, K) or unpaired two‐tailed *t*‐test (L). Source data are available online for this figure.

**Figure EV1:**
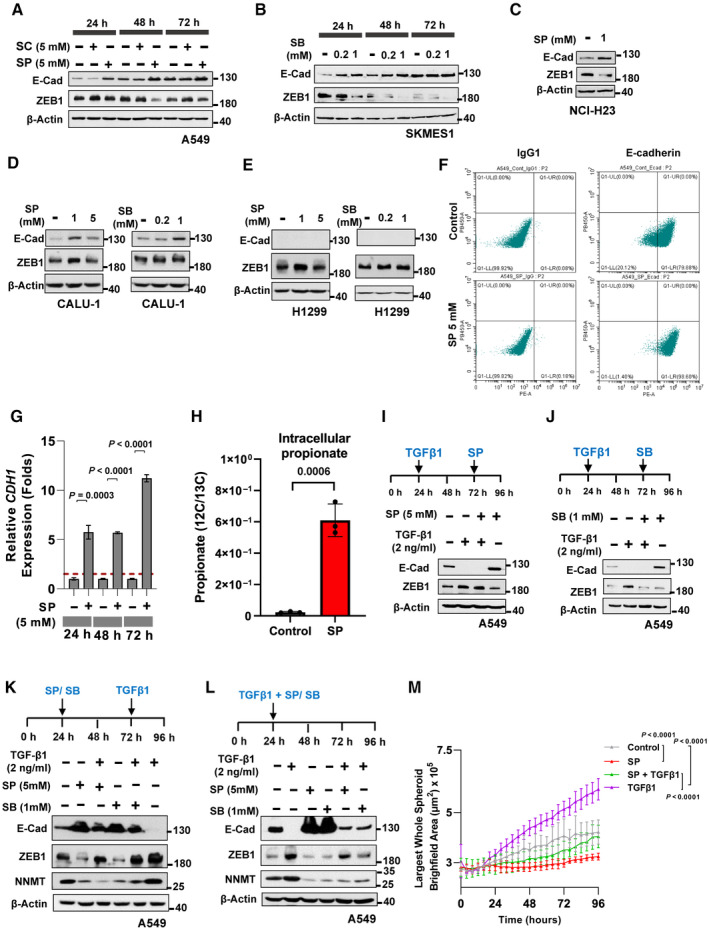
*In vitro* treatment effect of SCFAs, propionate or butanoate, in EMT marker gene expression AWestern blot analysis of E‐cadherin and ZEB1 in A549 cell line treated with indicated equimolar concentration of sodium chloride (SC) or sodium propionate (SP) in a time‐dependent manner. β‐Actin was used as an internal control.BWestern blot analysis of E‐cadherin and ZEB1 in SKMES1 cells treated with sodium butanoate (SB) in the indicated dose‐ and time‐dependent manner. β‐Actin was used as an internal control. The experiment was performed three independent times.CWestern blot analysis of E‐cadherin and ZEB1 in NCI‐H23 cell line treated with 1 mM of sodium propionate for 72 h. β‐Actin was used as an internal control. The experiment was performed three independent times.D, EWestern blot analysis of E‐cadherin and ZEB1 in CALU‐1 (D) and in H1299 (E) cell lines treated with sodium propionate (SP) or sodium butanoate (SB) in the indicated dose‐dependent manner for 48 h. β‐Actin was used as an internal control. The experiment was performed three independent times.FFlow cytometry analysis of membrane‐associated E‐cadherin (PE‐conjugated E‐cadherin) in A549 cells treated with sodium propionate at 5 mM concentration for 48 h. Mean fluorescence intensity of PE‐E‐cadherin in SP treated cells and controls were 62,858 and 21,999, respectively. PE‐conjugated IgG1 was used as a control for flow cytometry. The experiment was performed three independent times.GReal‐time quantitative PCR analysis of epithelial genes *CDH1* in A549 cell line treated with sodium propionate (SP) at 5 mM for 3 days. *GAPDH* was used as an internal control. Red dotted line represents the fold change cut‐off at 1.5. Data points (*n* = 3) are technical replicates represented as mean ± SD of one experiment, and the experiment was performed three independent times. Significance was calculated using unpaired *t*‐test.HDetection of intracellular levels of propionate in A549 cell line treated with SP (5 mM) for 3 days (*n* = 3). 13C propionate was used as an internal standard. Data is represented as mean ± SD of technical replicates and significance was calculated using unpaired *t*‐test.I, JWestern blot analysis of E‐cadherin and ZEB1 in A549 cells treated with TGF‐β1 (2 ng/ml) for 48 h followed by treatment with 5 mM SP (I) or 1 mM SB (J) for 24 h. β‐Actin was used as an internal control. The experiments were repeated three independent times.KWestern blot analysis of E‐cadherin, ZEB1 and NNMT protein levels in A549 cells pre‐treated with sodium propionate (SP, 5 mM) or sodium butanoate (SB, 1 mM) for 48 h followed by TGF‐β1 (2 ng/ml) for 24 h. β‐Actin was used as an internal control. The experiment was performed three independent times.LWestern blot analysis of E‐cadherin, ZEB1 and NNMT protein levels in A549 cells co‐treated with sodium propionate (SP, 5 mM) or sodium butanoate (SB, 1 mM) in combination with TGF‐β1 (2 ng/ml) for 72 h. β‐Actin was used as an internal control. The experiment was performed three independent times.MLine Plot depicts the quantification of spheroid brightfield area of A549 spheroids treated with sodium propionate (SP, 5 mM) and TGF‐β1 (2 ng/ml). Data points (*n* = 4) are technical replicates represented as mean ± SD and the experiment was performed three independent times. *P*‐value was calculated from two‐way ANOVA followed by multiple comparison between conditions using Tukey's multiple comparison test.

Prompted by the negative association of SCFAs and EMT, we investigated a potential EMT‐inhibitory effect for the SCFAs predominantly found in humans (acetate, propionate, and butanoate). Treatment of a partial EMT adenocarcinoma cell line A549 with sodium acetate (SA), sodium propionate (SP) and sodium butanoate (SB) showed a drastic increase in the key epithelial gene marker E‐cadherin, with a decrease in the mesenchymal master regulator ZEB1 for propionate and butanoate, but not for acetate (Fig 2A–C), in line with the genomic analysis. E‐cadherin peaks generally with SP or SB at early time points and is maintained higher within the 72 h treatment. The confluency, which increases E‐cadherin expression due to the establishment of cell–cell contacts, was not observed to be altered by the treatment. The experiment was controlled by sodium chloride to rule out sodium ion's effect (Fig EV1A). EMT inhibition by SP and SB was further experimented in multiple NSCLC cell lines representing two main histological subtypes, squamous cell carcinoma (SKMES1, NCI‐H520, and CALU‐1) and in two additional adenocarcinoma cell lines (NCI‐H23 and H1299), and was observed in SKMES1, NCI‐H520 and NCI‐H23 (Figs 2D and E, and EV1B and C). Interestingly, while mesenchymal‐like cell line CALU‐1 showed an increase in E‐cadherin, there was no change in the ZEB1 level with SCFAs treatment (Fig EV1D). On the other hand, highly metastatic H1299 (with no baseline E‐cadherin) displayed no effect with SP and SB (Fig EV1E). Overall, mesenchymal‐like cells showed no or lower effect compared to partial EMT cell lines like A549.

Immunofluorescence staining was conducted to show a functional E‐cadherin increase at the membrane region by SP treatment in NSCLC cell lines (Fig 2F). Similar EMT inhibitory effects of SP and SB were confirmed in another cancer type using the pancreatic cancer cell line, PANC1 (Appendix Fig S2A–C). Flow cytometry analysis indicated a substantial increase (2.9 folds) of cell surface stained E‐cadherin with SP treatment in A549 cell line (Figs 2G and EV1F). Similarly, real‐time quantitative PCR analysis showed a significant fold increase in the E‐cadherin mRNA with SP treatment till 72 h (~11 folds) (Fig EV1G). With regard to EMT transcription factors (EMT‐TFs), an overall and consistent reduction in the level of ZEB1, ZEB2, TWIST1 and (to a minimal extent) SLUG was observed with SP treatment in the 72 h time course, while SNAIL was found up‐regulated, indicating the potential supporting role of EMT‐TFs inhibition to EMT reversal (Appendix Fig S2D). The EMT inhibition by SP was further validated by additional EMT‐specific marker, NNMT (Shaul *et al*, 2014; Yang *et al*, 2022) (Appendix Fig S2E). Finally, mass spectrometry analysis of A549 cells treated with SP confirmed a significant increase in the intracellular propionate level (~27 folds) indicating the cellular uptake of exogenously supplemented propionate (Fig EV1H).

Next, these SCFAs were tested with TGFβ1, a strong EMT promoter in cancer (Deshmukh *et al*, 2021). Treatment of A549 cells with SP or SB could not reverse TGFβ1‐induced EMT, implying that these metabolites have no ability to revert a fully transitioned mesenchymal phenotype (Fig EV1I and J). However, pre‐treatment of A549 cells with SP or SB for 48 h followed by TGFβ1 treatment showed a substantial inhibitory effect on the TGFβ1‐mediated EMT program (Figs 2H and EV1K). A similar level of inhibition was even observed when SCFAs were co‐treated along with TGF‐β1 (Figs 2I and EV1L), yet the pre‐treatment scenario was more effective in TGFβ1‐mediated EMT inhibition. In line with EMT attenuation, wound‐healing assay revealed a significant inhibition in the migration ability in cells treated with SP (Fig 2J and Appendix Fig S2F). Interestingly, SP treatment showed a significant reduction in the TGFβ1‐induced migratory ability in the 2D culture (Fig 2K) and also with Matrigel‐coated A549 spheroid culture (Fig EV1M). All these convincingly show at the functional level the protective effect of SCFAs propionate and butanoate from TGF‐β1 induced EMT program. Further adhesion assays showed a significant increase in the binding to extracellular matrix (ECM) proteins (collagen I/II, fibronectin, laminin, and vitronectin) implying that propionate enhances the cell‐to‐cell contact and cell‐to‐surface contacts (Fig 2L).

Overall, these results strongly indicate that propionate and butanoate treatment increase functional E‐cadherin and foster the establishment of cell contacts while inhibiting EMT features.

### Propionate inhibits lung colonization and reduces lymph node metastasis *in vivo*

Both propionate and butanoate have been tested in clinical trials for various conditions like diabetes and obesity. However, butanoate's suitability in the clinical and nutritional context is limited due to its unpleasant odor and rapid absorption rate in the upper gastrointestinal tract (Paparo *et al*, 2022; van Deuren *et al*, 2022), while propionate is a safe food ingredient with clinical benefits (Rangan & Mondino, 2022). Therefore, we set out to perform further *in vivo* and detailed mechanistic investigations more focused toward propionate's role in lung cancer.

**Figure 3:**
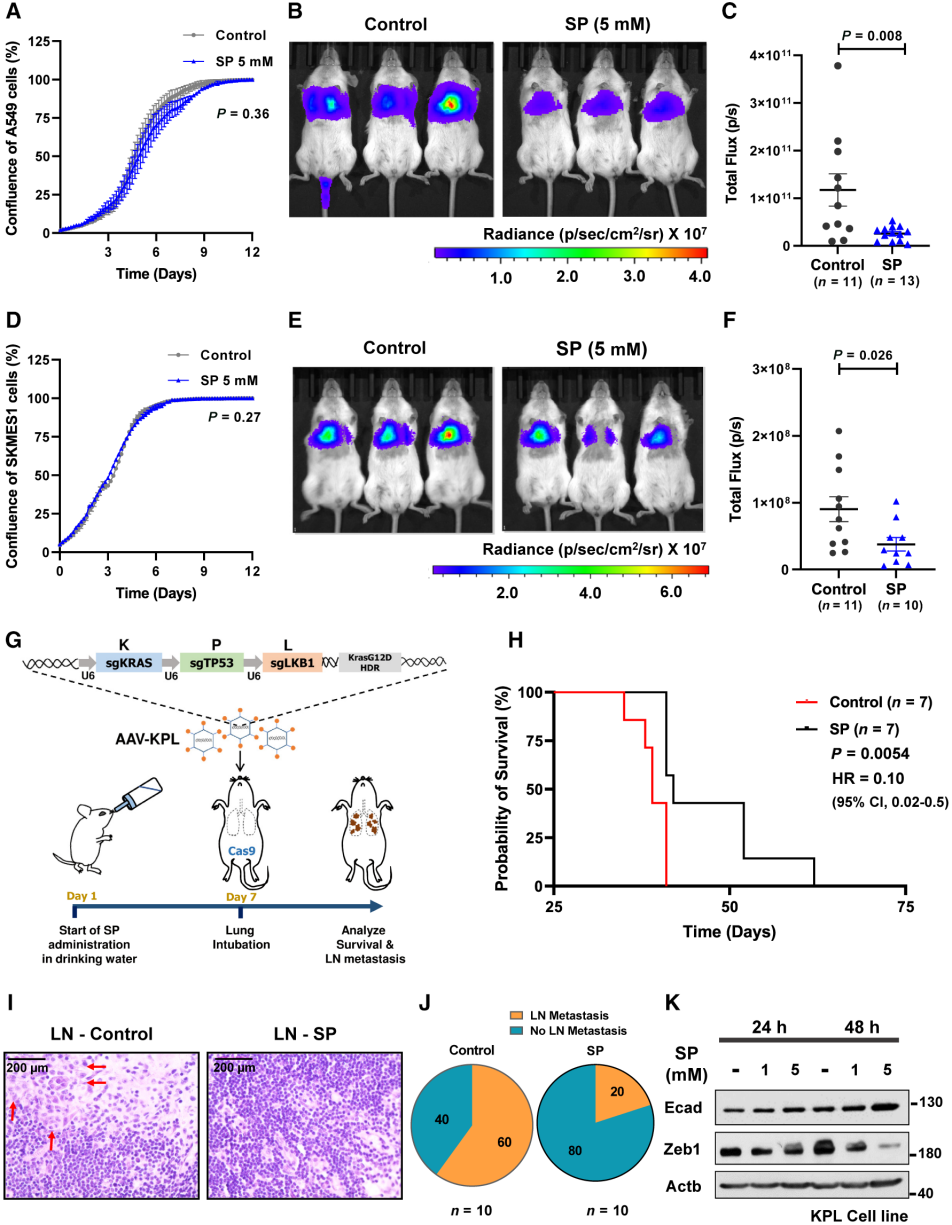
*In vivo* effect of propionate in lung metastatic mouse models AReal‐time cell proliferation analysis of A549 cells treated with sodium propionate (5 mM) for 12 days. Data points (*n* = 3) are technical replicates represented as mean ± SD of one experiment and the experiment was performed at least three independent times. *P*‐value was calculated from two‐way ANOVA.B, C
*In vivo* imaging of lung colonization ability of A549‐pFUL2G cells treated *in vitro* with sodium propionate (5 mM) for 3 days followed by tail‐vein injection in NSG mice (B). Quantification of luciferase activity as bioluminescence signal (total flux) in the lung metastatic NSG mice injected with A549‐pFUL2G cells treated *in vitro* with sodium propionate (5 mM) for 3 days (C). Data (*n* = 11 in control and *n* = 13 in SP) are represented as mean ± SEM with significance calculated using un‐paired two tailed *t*‐test.DReal‐time cell proliferation analysis of SKMES1 cells treated with sodium propionate (5 mM) for 12 days. Data points (*n* = 3) are technical replicates represented as mean ± SD of one experiment and the experiment was performed three independent times. *P*‐value was calculated from two‐way ANOVA.E, F
*In vivo* imaging of lung colonization ability of SKMES1‐pFUL2G cells treated *in vitro* with sodium propionate (5 mM) for 3 days followed by tail‐vein injection in NSG mice (E). Quantification of luciferase activity as bioluminescence signal (total flux) in the lung metastatic NSG mice injected with SKMES1‐pFUL2G cells treated *in vitro* with sodium propionate (5 mM) for 3 days (F). Data (*n* = 11 in control and *n* = 10 in SP) are represented as mean ± SEM with significance calculated using un‐paired two tailed *t*‐test.GSchematic representation of analysis of survival probability and lymph node metastatic inhibitory activity of sodium propionate by administering in drinking water to AAV‐KPL virus lung intubated Cas9‐C57BL/6 mice.HSurvival analysis of Cas9‐C57BL/6 mice with lung tumorigenesis intubated with AAV‐KPL virus and administered with sodium propionate (SP) in drinking water (*n* = 7 per group). HR – Hazard ratio for SP was calculated using Mantel–Haenszel method. Significance was calculated using log‐rank method.IRepresentative images of lung cancer metastasis to axial lymph nodes in KPL mice of control (LN‐Control) condition while no metastasis in the sodium propionate (LN‐SP) condition. Scale bar: 200 μm. Red arrows point to the metastatic cells in the lymph nodes.JPie chart representation of percent lung cancer metastasis to axial lymph nodes (*n* = 10 per group) in AAV‐KPL virus lung intubated Cas9‐C57BL/6 mice administered with sodium propionate (SP) in drinking water.KWestern blot analysis of E‐cadherin and Zeb1 levels in KPL cell line derived from Cas9‐C57BL/6 mice with lung tumorigenesis intubated with AAV‐KPL virus. β‐Actin was used as an internal control. The experiment was performed three independent times. Source data are available online for this figure.

SP treatment was not found to alter the proliferation of A549 cells *in vitro* (Fig 3A). Therefore, an experimental lung metastasis model was assessed wherein A549‐pFUL2G cells, expressing luciferase, were treated with SP and injected in the tail vein of NSG mice to monitor lung colonization ability. A marked decrease in the lung colonization ability of SP treated cells was observed compared to the control (Fig 3B and C) implying that propionate can inhibit metastasis independent of proliferation. Similar metastatic inhibitory ability of SP was also verified with another NSCLC cell line of squamous cell carcinoma origin, SKMES1 (Fig 3D–F).

In addition, to assess the effects of oral propionate administration *in vivo*, a different mouse model was employed wherein Cas9 knock‐in mice were administered with SP in drinking water as pre‐treatment from 1 week prior to lung intubation of an adeno‐associated virus inducing *Kras* ^*G12D/G12D*^ *p53* ^*Δ/Δ*^ *Lkb1* ^*Δ/Δ*^ (KPL) lung tumors (Fig 3G) prone to form lymph nodal (LN) metastases. Interestingly, oral propionate significantly extended the survival of the mice (Fig 3H), with no difference in drinking water consumption or body weight compared to control (Appendix Fig S3A and B). Further, a reduced LN metastasis was observed in SP administered mice (Fig 3I and J). Necroscopic analysis showed no significant difference in the number of primary tumor lesions between the groups (Appendix Fig S3C). Immunohistochemistry staining of epithelial cell adhesion molecule (EPCAM) protein in the KPL mouse lung tumor tissues showed an increasing trend for significance with SP administration (Appendix Fig S3D). Moreover, RNA‐seq analysis in a genome‐wide manner showed cell adhesion as a top significantly enriched molecular process in the SP administered KPL mouse lung tumor tissues (Appendix Fig S3E and F). In addition, *in vitro* treatment of a KPL‐derived cell line showed a similar suppression of EMT markers as observed with human cell lines upon SP (Fig 3K).

### Propionate increased the cisplatin sensitivity *in vitro*

**Figure 4:**
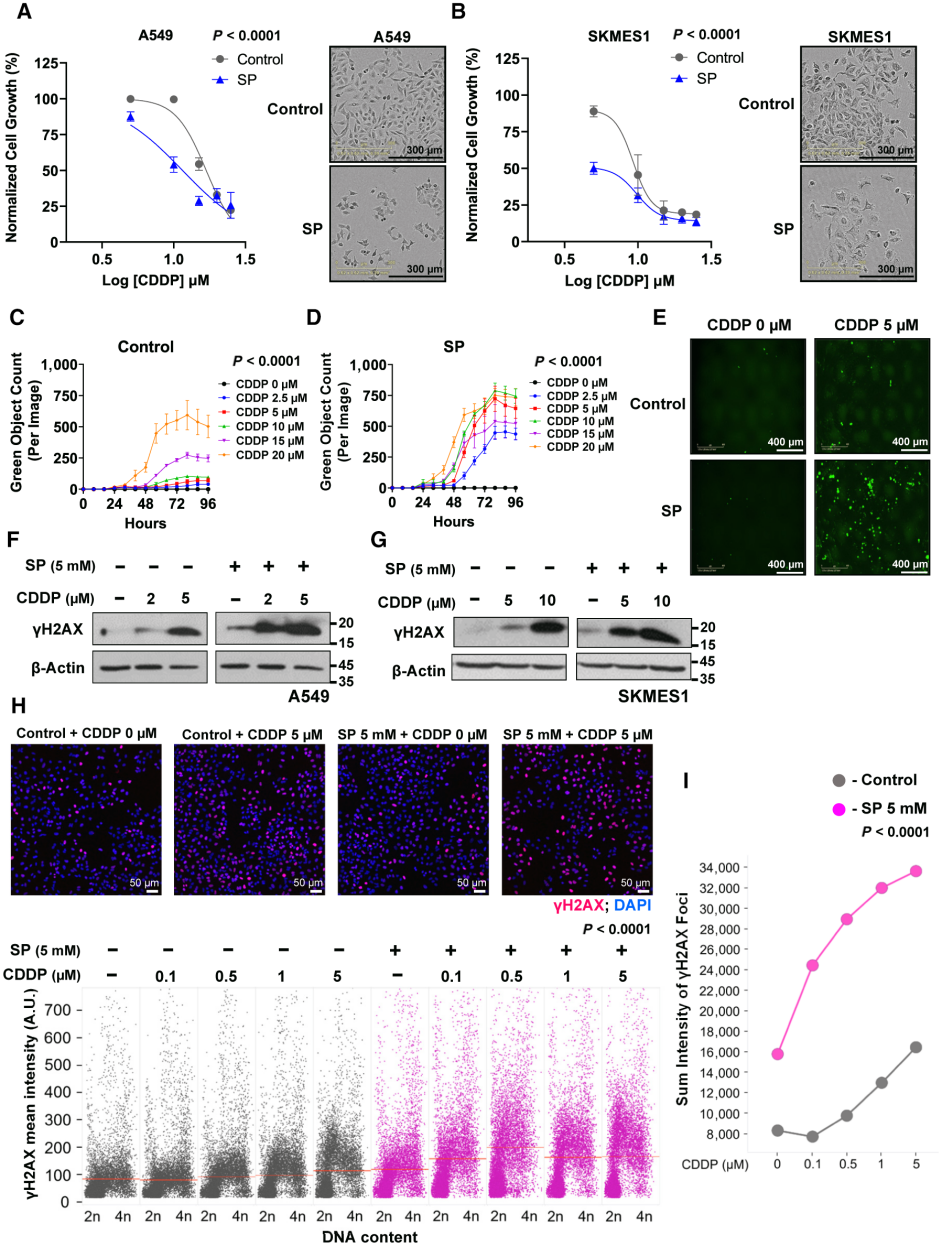
Sodium propionate sensitizes cells to cisplatin in lung cancer cell lines ADose responsive curve of cisplatin (CDDP) treatment in combination with SP (5 mM) in A549 cell line with pre‐treatment condition for 48 h. Data points (*n* = 3) are technical replicates represented as mean ± SD normalized to control of one experiment and the experiment was performed three independent times. *P*‐value was calculated from two‐way ANOVA analysis. In the right, images represent growth inhibition of A549 cell line pre‐treated with SP for 48 h followed by cisplatin (10 μM). Scale bars: 300 μm.BDose responsive curve of cisplatin (CDDP) treatment in combination with SP (5 mM) in SKMES1 cell line with pre‐treatment condition for 48 h. Data points (*n* = 3) are technical replicates represented as mean ± SD normalized to control of one experiment and the experiment was performed three independent times. *P*‐value was calculated from two‐way ANOVA analysis. In the right, images represent growth inhibition of SKMES1 cell line pre‐treated with SP for 48 h followed by cisplatin (5 μM). Scale bars: 300 μm.C–EQuantification of dead cells (green object count) using Cytotox Green in A549 cells treated with dose‐dependent cisplatin (CDDP) in the absence (C) and presence of SP (D) with pre‐treatment condition for 48 h. Data points (*n* = 3) are technical replicates represented as mean ± SD of one experiment and the experiment was performed three independent times. *P*‐value was calculated from two‐way ANOVA analysis. Representative images of A549 cells treated with cisplatin (CDDP, 5 μM) in combination with SP (5 mM). Scale bars: 400 μm (E).F, GWestern blot analysis of γH2AX levels in A549 cells (F) and SKMES1 cells (G) treated with cisplatin (CDDP) in the indicated dose‐dependent concentrations along with SP (5 mM). β‐Actin was used as an internal control. The experiment was performed three independent times.HQIBC analysis of γH2AX levels in A549 cells treated in the indicated dose‐dependent concentrations of cisplatin (CDDP) for around 6 h in combination with SP pre‐treatment (5 mM) for 24 h (bottom). Dots represent single cells (*n* = ~9,000) and significance (*P* < 0.0001) was calculated using two‐way ANOVA. Top, Images represent the γH2AX levels in A549 cells treated in the presence and absence of cisplatin (5 μM) along with SP pre‐treatment (5 mM) for 24 h. DAPI was used as a nuclei stain. Scale bars: 50 μm.IQIBC analysis of sum intensity levels of γH2AX foci in A549 cells (*n* = ~9,000 single cells) showing an increasing trend with the treatment of cisplatin (CDDP) in the indicated dose‐dependent concentrations in combination with SP (5 mM) with pre‐treatment condition for 24 h. Significance was calculated using two‐way ANOVA. Source data are available online for this figure.

EMT contributes to chemoresistance during treatment regimen with cisplatin, a widely used drug for lung cancer patients (Shintani *et al*, 2011; De Las Rivas *et al*, 2021). Therefore, the ability of SCFAs to alter sensitivity of cancer cells to cisplatin was measured. Cells pre‐treated with SP or SB significantly showed decreased cell growth when treated with cisplatin compared to control cells in NSCLC cell lines (Fig 4A and B, Appendix Fig S4A and B) suggesting an improved sensitivity. In addition, cell death, evaluated by cytotoxic green dye, was found more pronounced with propionate (or butanoate) in combination with cisplatin in a dose‐dependent manner (Fig 4C–E and Appendix Fig S4C) and, notably, the difference was significant even with low dose cisplatin (2.5 μM) in combination with SP (Appendix Fig S4D). The increased sensitivity of cells toward cisplatin by propionate was further confirmed by elevated γH2AX expression, a sensitive biomarker of DNA damage and chromatin stress, as observed with protein expression in NSCLC cell lines (Fig 4F and G). We also employed multi‐color quantitative image‐based cytometry (QIBC) to simultaneously analyze cell cycle, DNA replication, and DNA‐damage responses at a single‐cell level in large cell populations. Interestingly, SP treatment alone neither altered the DNA replication rate (EdU incorporation on nascent DNA) and the cell cycle phase transitions nor instigated DNA damage, as evidenced by RAD51 and 53BP1 foci levels, two critical players of the genome repair during homologous recombination and DNA end joining, respectively (Appendix Fig S4E–H). However, SP treatment alone showed dramatic S‐phase specific increase in the γH2AX levels which augmented further upon treatment with increasing doses of cisplatin (Fig 4H and I). Once again, the γ‐H2AX increasing pattern was not echoed by the incidence of RAD51 foci upon cisplatin and SP co‐treatments (Appendix Fig S4H), raising an intriguing possibility that SP treatment alters chromatin in a hitherto unrecognized manner that sensitizes cisplatin treatment.

### Gene expression profiling of propionate treated cells reveal enhanced lung epithelial features in EMT inhibition

**Figure 5:**
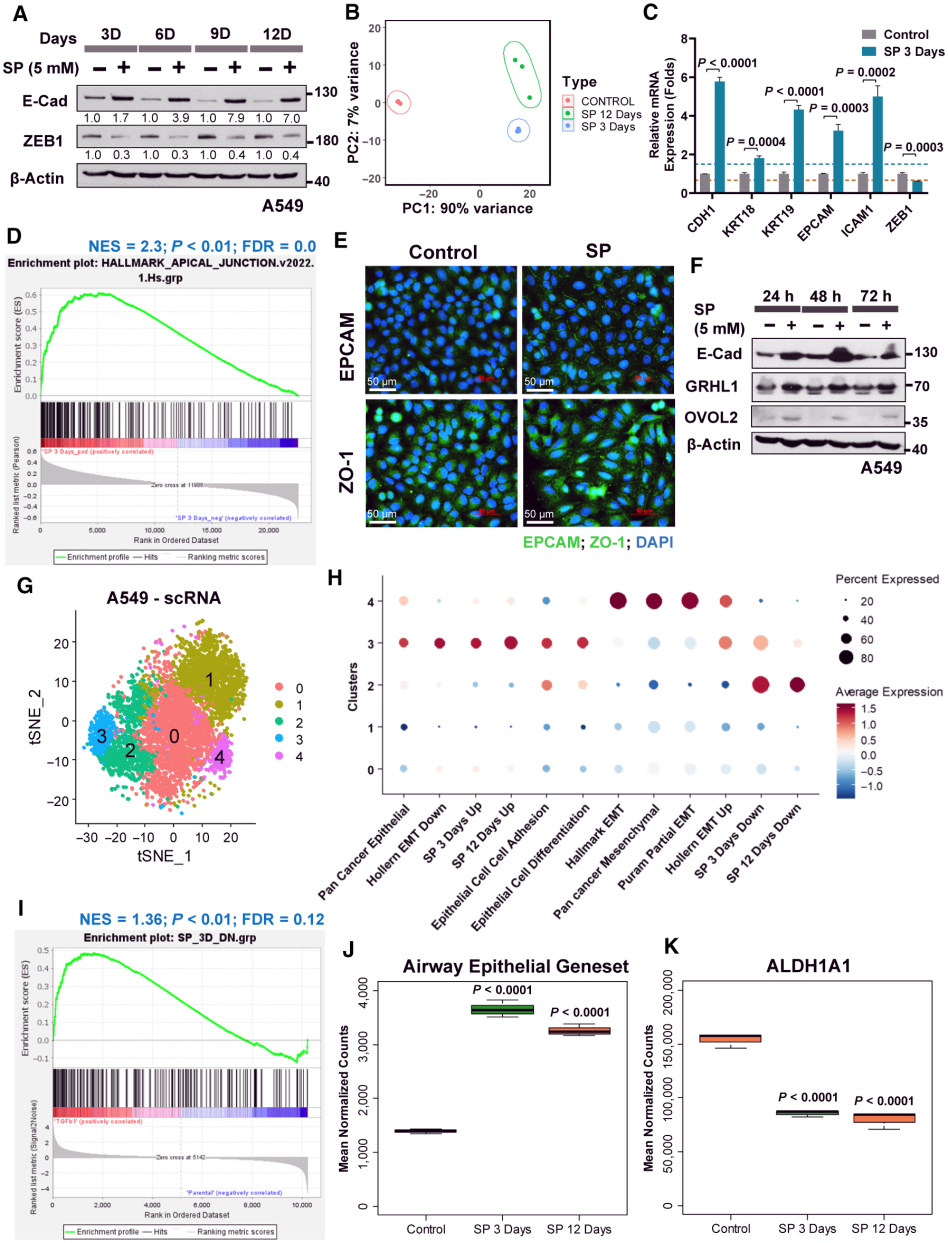
RNA‐seq expression profiling of SP treated A549 cell line reveals enriched epithelial gene expression AWestern blot analysis of E‐cadherin and ZEB1 protein expression in time series in A549 cell line treated with sodium propionate (SP) for 12 days. β‐Actin was used as an internal control. The experiment was performed three independent times. Numbers indicate the fold change compared to the control of the respective time points.BPrincipal component analysis of RNA‐seq expression profile of A549 cell line treated with SP for 3 and 12 days reveals a high variance in the gene expression profiles between the treated and the control samples (*n* = 3 per group).CReal‐time quantitative PCR analysis of epithelial genes (*CDH1*, *KRT18*, *KRT19*, *EPCAM* and *ICAM1*) and mesenchymal gene (*ZEB1*) in A549 cell line treated with sodium propionate (SP, 5 mM) for 3 days. *GAPDH* was used as an internal control. Blue and red dotted lines represent the fold change cut‐off at 1.5 and 0.67, respectively. Data points (*n* = 3) are technical replicates represented as mean ± SD of one experiment, and the experiment was performed three independent times. Significance was calculated using unpaired *t*‐test.DGene set enrichment analysis of hallmark apical junction gene‐set enrichment in a lung cancer gene expression profile GSE72094 (*N* = 442) as continuous label of SP 3 days gene‐set *z*‐score activity. Ranking of genes was based on Pearson's correlation metric in GSEA.EImmunofluorescence staining of EPCAM and ZO‐1 in A549 cell line treated with sodium propionate (SP, 5 mM) for 3 days. DAPI was used as a nuclear stain. Scale bars: 50 μm.FWestern blot analysis of GRHL1 and OVOL2 protein levels in time series in A549 cell line treated with sodium propionate (SP, 5 mM) for 3 days showed an increase in the epithelial‐specific transcription factors expression. β‐Actin was used as an internal control.GSingle cell RNA sequencing of parental A549 cell line revealed five different clusters of cells obtained from t‐SNE (t‐distributed stochastic neighbor embedding) analysis with a resolution of 0.4 and perplexity of 50 from first 25 principal components.HDot plot pattern analysis of epithelial and mesenchymal gene‐sets using AddmoduleScore analysis in Seurat showed the enrichment of SP up‐regulated genes along with the epithelial gene‐sets in cluster 3 of parental A549 cells from scRNA sequencing.IGene set enrichment analysis of SP 3 days down‐regulated gene‐set in TGFβ1‐induced HMLE cell line compared to the control obtained from GSE24202 (*n* = 3). Ranking of genes with signal2noise metric was used for GSEA.J, KBox plot visualization of the enriched airway epithelial cell‐type associated gene‐set (J) and cancer stem cell marker, ALDH1A1 (K) in SP 3 days and SP 12 days samples compared to the control. The central band inside the box represents the median value of the data (*n* = 3) obtained using the lower (bottom) and upper (top) quartile values of the box. The maximum and minimum values of the data are displayed with vertical lines (whiskers) connecting the box. Significance was calculated using un‐paired *t*‐test between the SP treated cells and the control. Source data are available online for this figure.

**Figure EV2:**
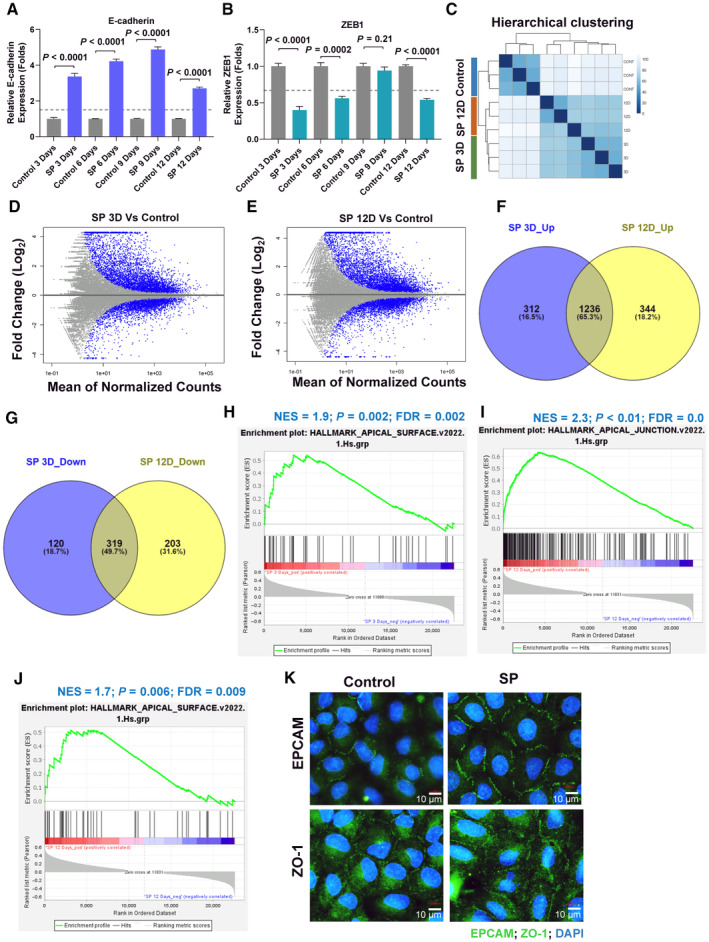
RNA‐seq expression profiling of SP treated samples from 3 days and 12 days show similar gene expression pattern with enrichment of epithelial features A, BReal‐time quantitative PCR analysis of E‐cadherin (A) and ZEB1 (B) in A549 cell line treated with sodium propionate at 5 mM in time series for 12 days. Dotted line represents the fold change cut‐off at 1.5 (A) or at 0.67 (B). Data points (*n* = 3) are technical replicates represented as mean ± SD of one experiment and the experiment was performed three independent times. Significance was calculated using un‐paired *t*‐test.CUnsupervised hierarchical clustering analysis of RNA‐seq samples of A549 cell line treated with SP for 3 days and 12 days along with control (*n* = 3 per group).D, EMA plot of differentially expressed genes identified in SP treated 3 days (D) and 12 days (E) compared to the control (*n* = 3 per group). Blue dots represent the significantly differentially expressed genes with a fold change of log_2_ (1).F, GVenn diagram representation of overlap analysis between SP 3 days and SP 12 days up‐regulated (F) or down‐regulated (G) genes.H–JGSEA of hallmark apical surface gene‐set enrichment in SP 3 days samples compared to the control (H), and enrichment of hallmark apical junction gene‐set (I) or hallmark apical surface (J) in SP 12 days samples compared to the control (*n* = 3). Ranking of genes based on Pearson's correlation metric was used for GSEA.KMagnified images of immunofluorescence staining of EPCAM and ZO‐1 in A549 cell line treated with sodium propionate (SP) at 5 mM for 3 days from Fig 5E showing the increased expression of EPCAM and ZO‐1 in the membrane region. DAPI was used as a nuclear stain. Image was re‐used from Fig 5E. Scale bars: 10 μm.

Molecular changes associated with propionate treatment *in vitro* were investigated by gene expression profiling. Increased E‐cadherin with decreased ZEB1 expression, at the translational and transcriptional levels, was consistent with 12 days long SP treatment (Figs 5A and EV2A and B). Therefore, differentially expressed genes were analyzed from RNA‐seq profiling of 3 and 12 days SP‐treated A549 cells compared to control (Figs 5B and EV2C–E). The up‐regulated and down‐regulated genes from 3 and 12 days SP profiles shared 65.3 and 49.7% genes, respectively, indicating similar gene expression maintenance over longer SP treatment (Fig EV2F and G). Overall, the results indicated reinforcement of lung epithelial features by SP treatment. The expression pattern was validated at multiple levels: (i) with high folds epithelial genes expression (*KRT18*, *KRT19*, *EPCAM*, *ICAM1* and *CDH1*) by quantitative PCR (Fig 5C), (ii) GSEA of hallmark apical junction and apical surface gene‐sets enrichment with SP gene‐sets activation pattern in lung cancer patients (Figs 5D and EV2H–J), (iii) enhanced expression of cell adhesion (EPCAM) and tight junction (ZO‐1) proteins at the cytoplasmic membrane region (Figs 5E and EV2K), and (iv) increased epithelial transcription factors GRHL1 and OVOL2 upon SP treatment (Fig 5F).

**Figure EV3:**
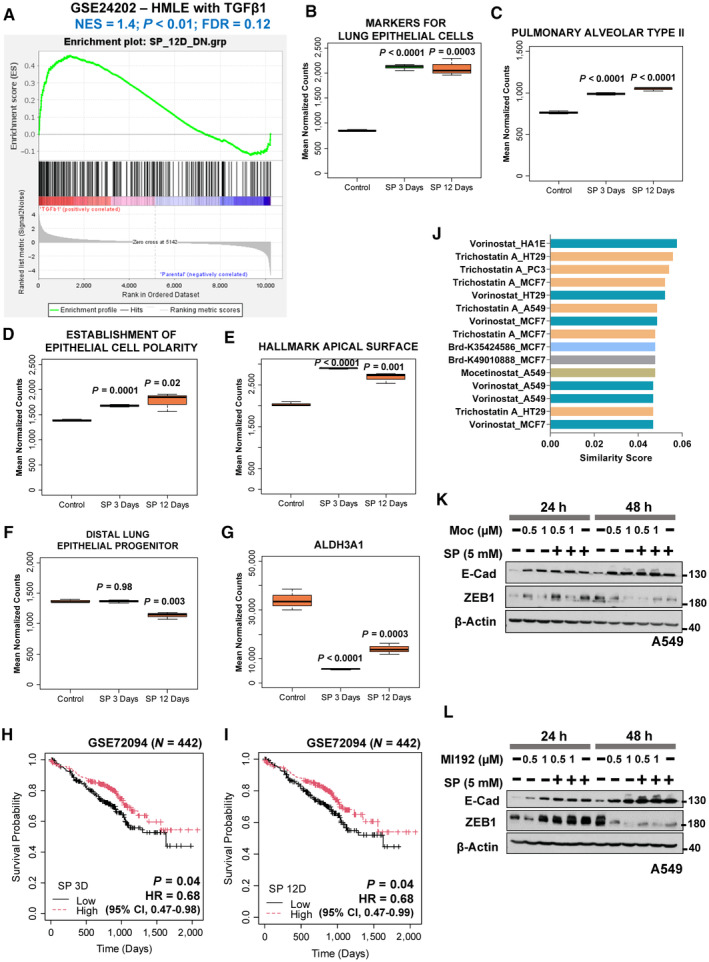
Gene expression profiling of SP treated cells showed lung specific epithelial program with epigenetic involvement AGene set enrichment analysis of SP 12 days down‐regulated gene‐set in TGFβ1‐induced HMLE cell line compared to the control obtained from GEO (GSE24202) (*n* = 3). Ranking of genes with signal2noise metric was used for GSEA.B–GBox plot visualization of the markers of lung epithelial cell‐type gene‐set (B), pulmonary alveolar type II cell‐type gene‐set (C), establishment of epithelial cell polarity gene‐set (D), hallmark apical surface gene‐set (E), distal lung epithelial progenitor genes (F), and cancer stem cell marker (ALDH3A1 (G)) in control, SP 3 days and SP 12 days RNA‐seq samples of A549 cell line. The central band inside the box represents the median value of the data (*n* = 3) obtained using the lower (bottom) and upper (top) quartile values of the box. The maximum and minimum values of the data are displayed with vertical lines (whiskers) connecting the box. Significance was calculated using un‐paired *t*‐test between the SP treated cells and the control. All the lung epithelial cell‐type associated gene‐sets were collected from PanglaoDB (B–D) and hallmark apical surface gene‐set from MSigDB (E).H, IOverall survival analysis in lung cancer patient samples (GSE72094; *N* = 442) categorized as low‐ and high‐propionate levels of SP 3 days (H) or SP 12 days (I) gene‐set *z*‐score activity based on the median showed good prognosis. HR – Hazard ratio for high propionate group was calculated using Cox proportional hazards model. *P*‐value was calculated using log‐rank method.JBar plot indicates the top ranked drug gene signatures similar to SP 12 days gene‐set based on the similarity score identified from L1000CDS^2^ search engine. Identical drugs from different treatment conditions are colored the same.K, LWestern blot analysis of E‐cadherin and ZEB1 in A549 cells treated with HDAC inhibitor, mocetinostat (K) or MI192 (L) in the indicated dose‐ and time‐dependent manner in combination with SP 5 mM. β‐Actin was used as an internal control.

Furthermore, a single‐cell RNA‐sequencing of untreated parental A549 cells was performed, and interestingly, delineated a cluster of epithelial‐like (cluster 3) and a mesenchymal‐like (cluster 4) cell features defined from cell cluster's marker genes overlap analysis (Fig 5G, Appendix Fig S5A and Appendix Table S6). While cluster 3 marker genes were significantly enriched with epithelial cell‐types including airway cells and apical junction feature, cluster 4 marker genes were enriched with mesenchymal cell‐types including fibroblasts along with EMT process and TGF‐β1 signaling (Appendix Fig S5B–J) suggesting A549 cells are hybrid‐EMT cells. With the defined clusters from scRNA‐seq, we analyzed the gene expression pattern of SP regulated gene‐sets (from RNA‐seq), and identified that epithelial‐like cell cluster (cluster 3) was found to possess SP up‐regulated gene‐sets from 3 and 12 days along with other epithelial‐associated gene‐sets, although SP down‐regulated gene‐sets were not found in cluster 4 (Fig 5H). Nevertheless, GSEA of SP down‐regulated gene‐sets from 3 or 12 days were found enriched in the genetically‐induced EMT condition with TGF‐β1 (Figs 5I and EV3A). Furthermore, the role of SP's in lung epithelial feature enhancement was confirmed with the increased expression of airway epithelial cell type gene‐sets including pulmonary alveolar type‐II cells in both SP 3 and 12 days compared to control (Figs 5J and EV3B–E). Moreover, SP significantly down‐regulated the expression of distal lung epithelial progenitor genes (Rawlins, 2008) at 12 days, and cancer stem cell markers (ALDH1A1 and ALDH3A1) expression (Figs 5K and EV3F and G) implying propionate's effect on cancer stemness related to EMT. A better prognosis in lung cancer patients was also observed for both SP 3 and 12 days gene‐sets (Fig EV3H and I), in line with the anti‐tumoral role of propionate observed in the mouse models. Altogether, expression profiling in response to SP treatment showed robust induction of lung‐specific epithelial features.

### Propionate reinforces epithelial identity via chromatin remodeling

**Figure 6:**
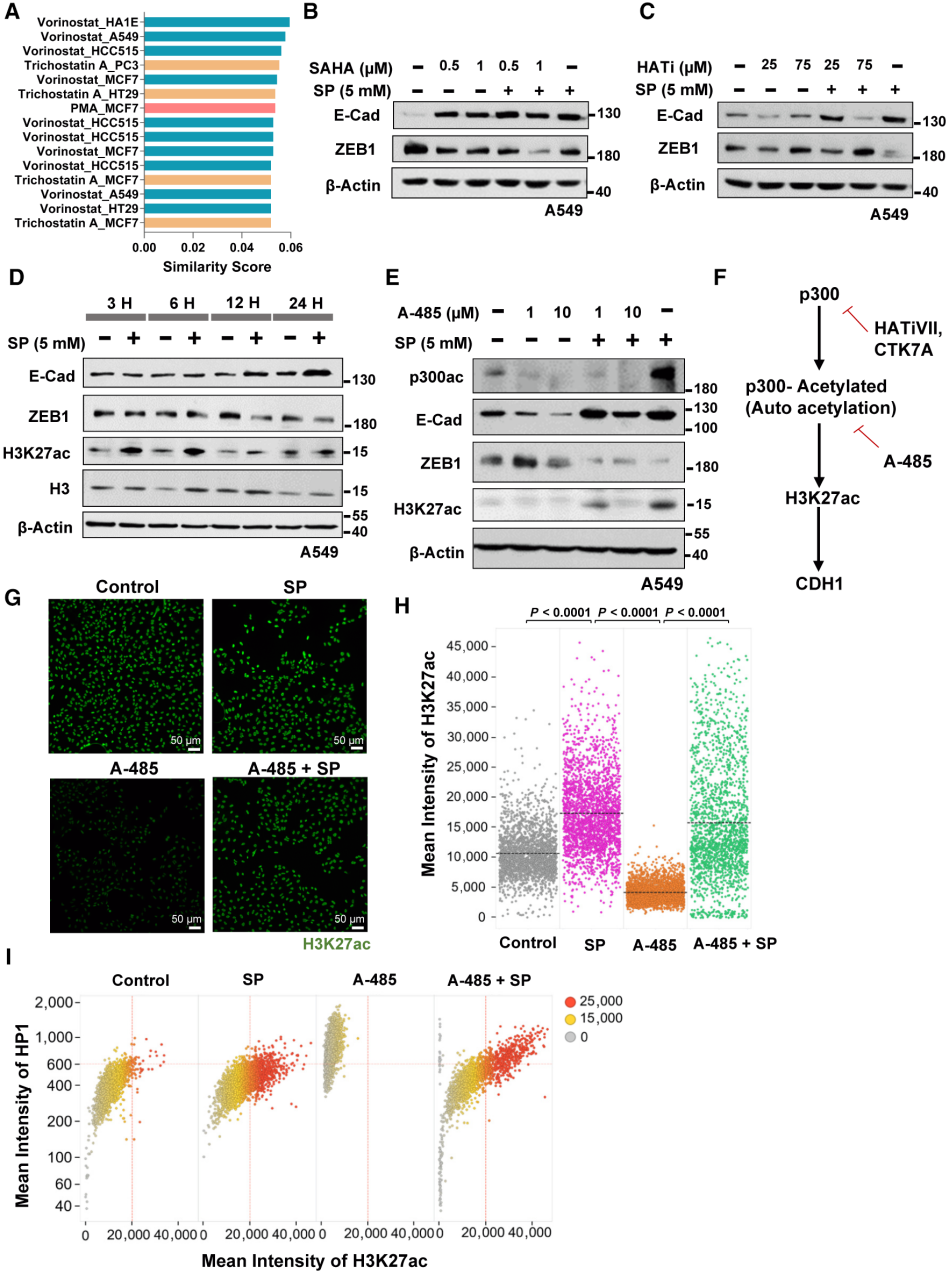
Sodium propionate modulates E‐cadherin expression through p300 signaling ABar plot indicates the top ranked drug gene signatures similar to SP 12 days gene‐set based on the similarity score identified from L1000CDS^2^. Identical drugs from different treatment conditions are colored the same.B, CWestern blot analysis of E‐cadherin and ZEB1 protein expression in A549 cells treated with vorinostat (SAHA) (B) or Histone Acetyl Transferase inhibitor VII, CTK7A (HATi) (C) in the indicated dose‐dependent concentrations in combination with sodium propionate (SP, 5 mM) for 24 h. β‐Actin was used as an internal control. The experiments were performed three independent times.DWestern blot analysis of E‐cadherin, ZEB1, H3K27ac and H3 in A549 cells treated with sodium propionate (SP, 5 mM) in time series for 24 h. β‐Actin was used as an internal control. The experiment was performed three independent times.EWestern blot analysis of p300 acetylation, E‐cadherin, ZEB1 and H3K27ac in A549 cell line treated with HAT inhibitor, A‐485, in the indicated dose‐dependent concentrations in combination with sodium propionate (SP, 5 mM) for 24 h. β‐Actin was used as an internal control. The experiment was performed three independent times.FSchematic flowchart of sodium propionate's role in the regulation of E‐cadherin expression through p300 histone acetyl transferase activity on H3K27ac mark.GImages represent the H3K27ac mark levels analyzed by QIBC in A549 cells treated with A‐485 (10 μM) in the presence and absence of SP (5 mM). DAPI was used as a nuclei stain. Scale bars: 50 μm.HQIBC analysis of mean intensity levels of H3K27ac mark in A549 cells (*n* = ~2000 single cells) treated with A‐485 (10 μM) in the presence and absence of SP (5 mM) for 24 h. Dots represent single cells and significance was calculated using One‐way ANOVA with *post‐hoc* analysis of *t*‐test between the conditions.IBiplot representation of mean intensity of HP1 to H3K27ac mark levels from QIBC analysis in A549 cells (*n* = ~2000 single cells) treated with A‐485 (10 μM) in the presence and absence of SP (5 mM) for 24 h. Color intensity values represent the mean intensity levels of the histone marks with gray representing null to red indicating high intensity. Source data are available online for this figure.

To understand the mechanism of propionate's mediation of E‐cadherin expression, a small molecule mimics search using L1000CDS^2^ search engine (Duan *et al*, 2016) identified histone deacetylases (HDAC) inhibitors in mimicking SP's gene‐set expression pattern (Figs 6A and EV3J). Therefore, the role of propionate in enforcing global chromatin‐based alterations was investigated. Although increased E‐cadherin was observed with HDAC inhibitors treatment in A549 cells (Figs 6B and EV3K and L), the effect was not additive in combination with SP. In contrast, treatment with histone acetyltransferases (HAT) inhibitor blocked the effect of SP‐mediated E‐cadherin expression (Fig 6C) indicating its expression is regulated via acetylation. Other possible propionate's mechanisms of action such as knockdown of propionate metabolic enzymes by siRNA showed no metabolic‐related effects on E‐cadherin regulation with SP treatment (Appendix Fig S6A–C) while treatment with SP showed a relative increase in PCCA and PCCB only at later time points (Appendix Fig S6D). Similarly, blocking monocarboxylate transporter (MCT) (Nakamura *et al*, 2018), modulating G protein‐coupled receptors (GPR) (Thirunavukkarasan *et al*, 2017) or peroxisome proliferator‐activated receptor (PPAR) (den Besten *et al*, 2015) signaling with inhibitors also showed to not block or reduce E‐cadherin up‐regulation by propionate (Appendix Fig S6E–I).

An in‐depth investigation was extended on the propionate's role on HAT mediated E‐cadherin regulation. Since a recent report identified SCFAs (propionate and butyrate) role in H3K27ac via p300 (Thomas & Denu, 2021), we conducted a time series experiment in NSCLC cell lines and observed high H3K27ac at 3 h with increase in E‐cadherin at 12 h (Fig 6D). Treatment with A‐485, a catalytic inhibitor of p300/CBP (Lasko *et al*, 2017) decreased E‐cadherin and H3K27ac levels in combination with SP (Fig 6E and F, and Appendix Fig S6J). Furthermore, acetylated‐p300 level was found increased in SP treated cells compared to control. This suggests that propionate induces p300‐mediated signaling with increased H3K27 acetylation. There was no effect of H3K27me3 as inferred from EZH2 inhibitors (Appendix Fig S6K and L) indicating a specificity for the H3K27ac mark in E‐cadherin regulation by SP. Further, QIBC confirmed H3K27ac increase by SP (Fig 6G and H). Interestingly, analysis of H3K27ac together with HP1 (heterochromatin marker) showed that SP‐induced H3K27 acetylation is not associated with *de novo* heterochromatin formation, and thus, might represent a unique mechanism of chromatin alteration. This notion was further supported by the fact that direct H3K27ac abrogation by p300/CBP involved accompanying heterochromatinization displayed by *de novo* formation of HP1 compartments (Fig 6I).

**Figure 7:**
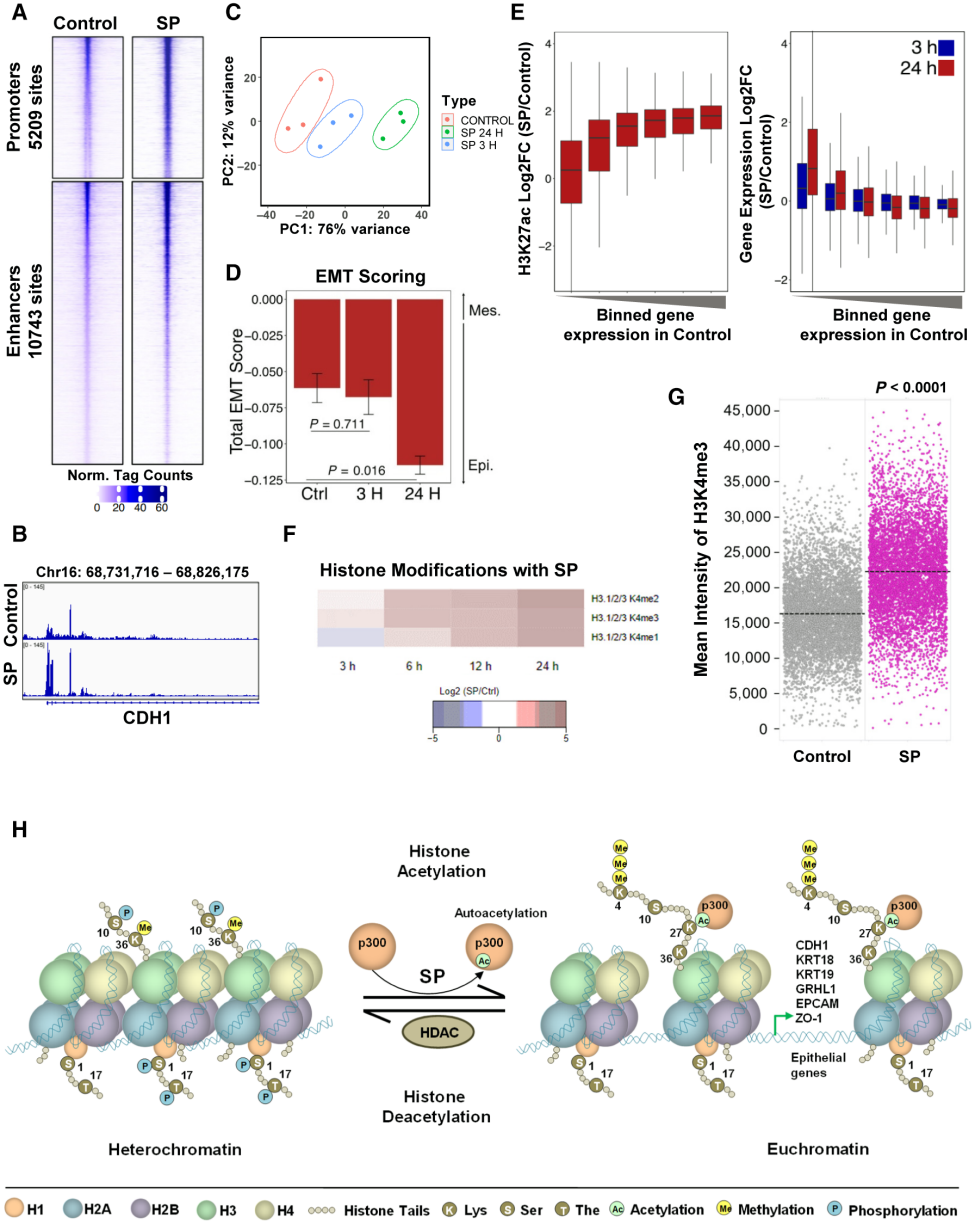
Sodium propionate induces epigenetic re‐programming toward transcriptional initiation or activation of an epithelial oriented program Heatmap visualization of ChIP‐sequencing for H3K27 acetylation (H3K27ac) mark enrichment at promoters and enhancers sites in A549 cells treated for 3 h with sodium propionate (SP, 5 mM) (*n* = 3 per group). Color intensity scale bar represents the normalized tag counts or read density at a given region.UCSC Genome browser screen shot visualization of H3K27ac peak enrichment at *CDH1* promoter region identified from ChIP‐seq for H3K27ac in A549 cells treated for 3 h with sodium propionate (SP, 5 mM). Peaks represented in the tracks are the normalized FPKM counts.Principal component analysis of RNA‐seq expression profile of A549 cell line treated with SP for 3 and 24 h (*n* = 3 per group).Bar plot represents the EMT score for control, 3 and 24 h RNA‐seq samples in biological replicates (*n* = 3) of A549 cells treated with sodium propionate. EMT score is the difference between the mesenchymal gene‐set score and epithelial gene‐set score obtained from SingScore. Data is represented as mean ± SE. *P*‐value was calculated from Welsch's two‐sided *t*‐test.Box plot visualization of H3K27ac and gene expression changes with sodium propionate treatment in A549 cells for all expressed genes sorted low‐to‐high binning of gene expression from control. Left plot represent the log_2_ fold difference in H3K27ac profile during SP treatment for 3 h while the right plot represents the log_2_ fold difference in the gene expression profile of SP for 3 h and 24 h. RNA‐seq and ChIP‐seq was performed in biological replicates (*n* = 3). Boxplot: Center line, median; box limits; upper and lower quartiles; whiskers, 1.5× interquartile range; outliers are not shown.Heatmap representation of top modulated post‐histone modifications identified by mass spectrometry in A549 cells treated with sodium propionate (SP) in time series for 24 h (*n* = 4 per group). Color intensity scale bar represent the log_2_ fold difference (FC) of SP with control (blue, FC < 0; white, FC = 0; red, FC > 0).QIBC analysis of mean intensity levels of H3K4me3 in A549 cells treated with sodium propionate (SP, 5 mM) for 24 h (*n* = ~7,000 single cells). Dots represent single cells and significance between the conditions was calculated using un‐paired *t*‐test.Schematic representation of the molecular mechanistic role of sodium propionate in epigenetic re‐programming to induce epithelial genes expression. Source data are available online for this figure.

**Figure EV4:**
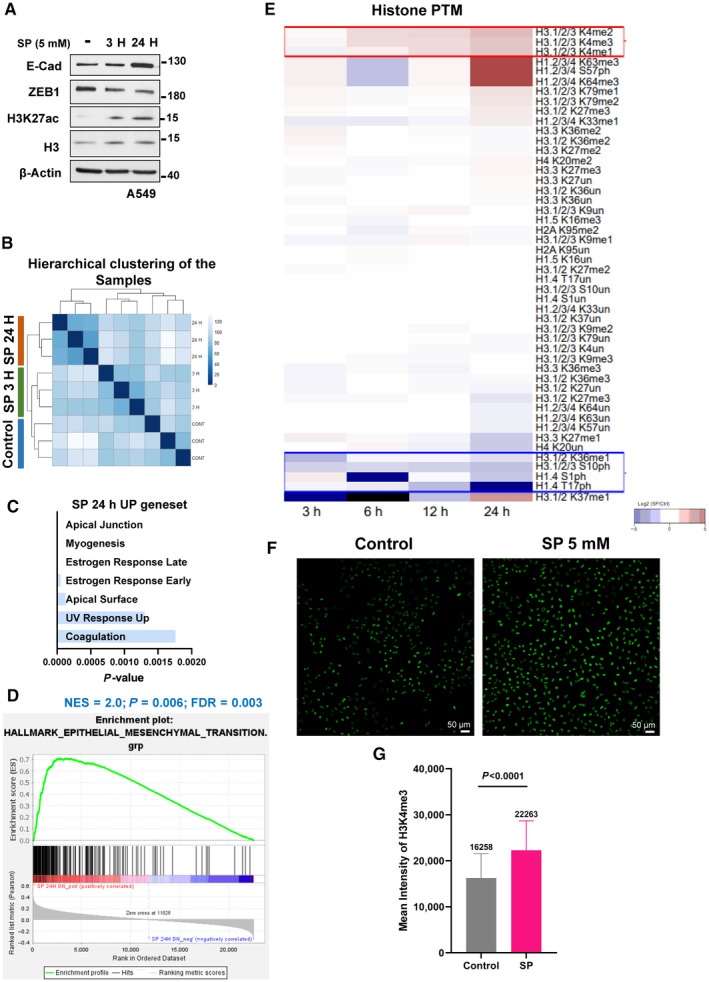
Propionate induces transcriptional histone active marks during epithelial gene expression program Western blot analysis of E‐cadherin, ZEB1, H3K27ac and H3 in A549 cells treated with sodium propionate (5 mM) for 3 and 24 h. β‐Actin was used as an internal control. The experiment was performed three independent times.Unsupervised hierarchical clustering analysis of RNA‐seq samples of A549 cell line treated with SP at 5 mM for 3 and 24 h along with control (*n* = 3 per group).Enrichr analysis of SP 24 h up‐regulated genes identified from RNA‐seq. *P*‐value was calculated using Fisher's exact test.GSEA analysis of hallmark EMT gene‐set enrichment analysis in a lung cancer gene expression profile (GSE72094, *N* = 442) as a continuous label of SP 24 h down‐regulated gene‐set *z*‐score activity. Ranking of genes was based on Pearson's correlation metric in GSEA.Heatmap representation of histone post‐translational modifications identified using mass spectrometry in A549 cells treated with sodium propionate at 5 mM in time series for 24 h (*n* = 4). Red highlighted box indicates the increased histone modifications in SP treated conditions compared to the control. Blue highlighted box indicates the decreased histone modifications in SP treated conditions compared to the control. Color intensity scale bar represent the log_2_ fold difference (FC) of SP with control (blue, FC < 0; white, FC = 0; red, FC > 0).Representative images from QIBC analysis of A549 cells treated with SP (5 mM) for 24 h and stained with H3K4me3 antibody. Scale bars: 50 μm.Bar plot of mean intensity level of H3K4me3 in A549 cells treated with SP (5 mM) for 24 h. Data points (*n* = ~7,000) are represented as mean ± SD and significance was calculated from un‐paired *t*‐test.

Indeed, a global increase in H3K27 acetylation levels in cells treated with SP for 3 h was observed using ChIP‐seq analysis along with H3K27ac peak enrichment at *CDH1* promoter site (Fig 7A and B, and EV4A). A parallel RNA‐seq for 3 h and 24 h propionate treated cells was also performed, and 24 h SP gene‐sets confirmed EMT inhibitory features (Figs 7C and EV4B–D). In addition, a time‐dependent decrease in the score with a previously published EMT gene signature (Tan *et al*, 2014) was observed from 3 to 24 h compared to the control (Fig 7D) implying SP promotes an epithelial‐specific gene expression program in part through epigenetic reprogramming. Interestingly, despite a global increase in H3K27ac, only the relatively modest SP‐mediated increase in this acetylation mark at promoters of lowly expressed genes was associated with gene activation (Fig 7E). This suggests that only largely inactive promoters are sensitive to the SP‐mediated increase in H3K27ac, thereby turning this global response of H3K27ac into a specific response promoting expression of lowly‐expressed epithelial genes.

Further investigation of other histone post‐translational modifications in A549 cells with SP treatment using mass spectrometry showed a time dependent increase in the transcriptionally active histone marks H3K4me1/2/3 while a significant repression of H3S10ph, H3K36me1, H1S1ph and H1T17ph histone marks were observed (Figs 7F and EV4E). Elevated H3K4me3 with SP was confirmed by QIBC (Figs 7G and EV4F and G) and increased H3K4me3 was found in the top SP‐induced genes (*SCUBE1*, *ESPN* and *GPR4*) including *CDH1* in a ChIP‐seq profile from Gene Expression Omnibus (GEO) (GSE35583) (Appendix Fig S7A–D). Similarly, A549 spheroid culture treatment with TGFβ/TNFα (GSE42374) showed decreased histone marks of H3K4me1/2/3 and H3K27ac for the epithelial genes (*CDH1*, *KRT19*, and *OVOL2*) with peak enrichment for *ZEB2* (Appendix Fig S7E–H).

Collectively, we discovered that propionate reduces lung cancer EMT by broad chromatin alterations encompassing H3K27ac and H3K4me1/2/3 marks accompanying transcriptional activation of epithelial gene expression shifting the cellular balance toward the epithelial state (Fig 7H).

## Discussion

Deregulated metabolism is now widely recognized as a driving hallmark of EMT, and particular focus on targeting metabolic processes that could inhibit the aggressive feature of EMT in cancer and in pulmonary fibrosis has gained clinical attention (Rout‐Pitt *et al*, 2018; Ramesh *et al*, 2020). While several inhibitors are being surpassed to clinical trials, metabolic inhibitors still face major challenges (Sun & Yang, 2020; Lemberg *et al*, 2022). In the present study, an integrative functional genomics approach has identified negative association of EMT with SCFAs propionate and butanoate in lung cancer of the NSCLC type, although the tumor gene expression profiles used in the study from the publicly available database could involve a mix of stroma and tumor cells, and the potential contribution of the tumor microenvironment remains to be investigated. SCFAs, produced by the gut microbiome, have shown therapeutic potential as dietary supplementation including in cancer treatment (Al‐Qadami *et al*, 2022). Several studies have highlighted their role in gluconeogenesis and maintenance of intestinal epithelial barrier integrity, and some suggest the existence of gut‐brain and gut‐lung axis crosstalk with SCFAs effect (Wong *et al*, 2006; Ghorbani *et al*, 2015; Dalile *et al*, 2019; Al‐Qadami *et al*, 2022). Fatty acids, in general, showed distinct roles with EMT phenomenon. For instance, elevation in the long‐chain free fatty acid palmitate has been shown to promote EMT in hepatocellular carcinoma via Wnt/β‐catenin and TGF‐β signaling (Nath *et al*, 2015). On the other hand, a decrease in the fatty acid oxidation was found during endothelial–mesenchymal transition by altering the intracellular levels of acetyl‐CoA (Xiong *et al*, 2018).

In the current study, propionate showed a standalone EMT marker genes modulation in multiple NSCLC cell lines with an increase in expression of the key epithelial gene, E‐cadherin, along with repression of ZEB1, a master regulator of EMT features including in early tumorigenesis and metastasis in NSCLC (Larsen *et al*, 2016; Brabletz *et al*, 2021). The activity of SP in E‐cadherin increase appears to be stronger at earlier time points when the cells have low cell–cell interactions with low baseline E‐cadherin, and thereby establishes a epithelial identity without impacting cell proliferation. However, the increased E‐cadherin by SP is maintained as observed with the SP treatment in longer time course experiment till 12 days. In addition, propionate could better inhibit the EMT in the partial EMT state with no or less effect in the complete mesenchymal state indicating that propionate is convincingly a better compound to act on highly‐aggressive and highly‐metastatic partial EMT state of the tumors (Pastushenko *et al*, 2018), and these results are pivotal in selection of patients for clinical trial testing with SCFAs. In fact, the effect of propionate on EMT is broader as the association was revealed by an integrative functional genomic analysis based on the pan‐cancer EMT gene signature, and further confirmed at the global level with RNA‐seq analysis of propionate treated cells at different time points. The epithelial reinforcement could also be observed in other cancer types such as in pancreatic cancer cell line. In the context of lung, propionate treatment increased the genes expression of pulmonary alveolar type‐II cell‐type implying the establishment of epithelial integrity in lungs. To further corroborate this model, parental NSCLC cells inherently retaining hybrid EMT mode with epithelial and mesenchymal cell clusters, as identified from scRNA‐seq analysis, revealed that propionate‐specific upregulated genes were predominantly enriched in the epithelial cell population cluster. All these strongly suggest propionate as a powerful epithelial enhancing factor in lung cancer cells.

Recently, it has been shown that deregulation in propionate metabolism contributes to an anaplerotic reaction in the accumulation of methylmalonic acid (MMA) mediated by ERK2 signaling resulting in breast and lung cancer with an increased EMT and metastatic potential (Gomes *et al*, 2022). However, in contrast to these findings, our study did not observe any propionate‐specific metabolic perturbations. Instead, treatment with propionate resulted in epigenetic reinforcement of the epithelial identity. This unprecedented dual and opposing effect on EMT could be a potential novel feedback loop regulating epithelial‐mesenchymal plasticity worth investigating.

EMT has been shown as an important priming factor in early steps of lung tumorigenesis as observed with the oncogenic‐mediated transformation of human bronchial cells (Sato *et al*, 2013) and with the cigarette smoke‐induced epigenetic alterations (Vaz *et al*, 2017). Our lung experimental metastasis model showed that SP could reduce the metastasis or lung colonization ability, and increase the *in vitro* chemosensitivity of cisplatin, a commonly used drug for lung cancer patients. This is in line with a report on cisplatin sensitization with propionate through an increase in the H3 acetylation levels through GPR41 signaling in liver cancer (Kobayashi *et al*, 2018). Interestingly, our detailed molecular analysis revealed that propionate induces H3K27ac via p300‐mediated signaling in a receptor‐independent manner. We postulate that propionate (as evidenced by high γH2AX levels without any DNA damage) by promoting replication stress due to changes in replication speed (Somyajit *et al*, 2017; Sedlackova *et al*, 2020) could augment cisplatin's action in inducing genomic instability causing cell death. Based on our findings, we propose that propionate could be a promising candidate for clinical investigation in NSCLC treatment. Further exploration of its *in vivo* efficacy and the underlying mechanism of action related to EMT could be crucial to fully leverage its potential to enhance clinical response to cisplatin, particularly in the context of early‐phase clinical trials where drug combinations with cisplatin‐based chemotherapy are frequently employed (Shimokawa *et al*, 2021).

The present study revealed the involvement of epigenetic reprogramming in propionate mediated epithelial gene expression. The role of SP as an HDAC inhibitor could not be ruled out as inferred from the gene‐set expression pattern matching with HDAC inhibitors gene signatures, which could also increase the E‐cadherin expression. However, treatment with HAT inhibitors (HAT inhibitor VII and A‐485) showed a convincing down‐regulation of E‐cadherin levels when treated in combination with SP. HAT inhibitor VII is a mixed mode selective inhibitor of p300/CBP and PCAF HATs, while A‐485 is a selective catalytic inhibitor of p300/CBP auto‐acetylation with no inhibitory activities on other acetyltransferases including PCAF, GCN5L2, MYST3/4, HAT1, MOF and TIP60 (Lasko *et al*, 2017). All these evidently point to the possibility that SP activates expression of epithelial genes including E‐cadherin through p300 activation. Recently, it has also been shown that propionate and butanoate metabolized to their corresponding acyl‐CoAs can activate p300 auto‐acylation in histone modification events including H3K27ac (Thomas & Denu, 2021). In future, it would be also interesting to study the dynamic role of SP in epigenetic reprogramming and EMT‐transcription factors modulation in inhibiting EMT. For instance, there could be a cross‐regulation between these entities.

Previous study showed that acetate could inhibit TGFβ1‐induced EMT features (Lyu *et al*, 2022). However, in our study, we did not find any modulation in E‐cadherin or ZEB1 levels upon sodium acetate treatment. Further, the study utilized 40 mM of sodium acetate to inhibit TGFβ1‐induced EMT features which is 40‐fold higher concentration than used in the current study. Therefore, acetate's role in inhibiting EMT in lung cancer cannot be excluded as higher concentration could have an impact in inhibiting EMT in lung cancer cells potentially through a similar mechanism of propionate and butanoate.

For the first time, propionate has been shown to have a dualistic role in inducing H3K27ac and H3K4me levels in our study. In the EMT context, demethylation of H3K4me2 by SNAIL mediated histone demethylase LSD1 (Lin *et al*, 2010), and loss of H3K27ac mark by transcriptional regulator MNT (Lavin *et al*, 2021) leads to the repression of epithelial identity genes. These support our findings on histone mark dynamicity of propionate in inducing H3K4me and H3K27ac mark levels to enhance the epithelial identity, and thereby reducing mesenchymal features. The current study also identified regulation of H3K36me1, H3S10ph, H1S1ph and H1T17ph histone marks, which would require further investigations.

Besides the genetic and epigenetic alterations, altered gut microbiome is also known to have a profound role in tumorigenesis through changes in the metabolic processes (Ge *et al*, 2021; Lythgoe *et al*, 2022). Recently, gut microbiota metabolomics of NSCLC patients undergoing immunotherapy with nivolumab showed that long‐term responders were majorly characterized with the presence of SCFA metabolites (propionic, butyric, acetic and valeric acids) with beneficial effects (Botticelli *et al*, 2020). Again, NSCLC patients responding to programmed cell death protein 1 (PD‐1) antibody therapy were found to have higher baseline levels of fecal SCFAs concentration (Zizzari *et al*, 2020). Similarly, immunotherapy for several solid cancers including lung adenocarcinoma with immune checkpoint inhibitors (nivolumab or pembrolizumab) exhibiting improved PD‐1 inhibitor efficacy showed high baseline fecal concentrations of SCFAs mainly propionic, butyric, valeric and acetic acid with longer progression‐free survival (Nomura *et al*, 2020). Therefore, propionate could be administered as a combinatorial drug along with immunotherapy for NSCLC patients for improved therapeutic efficacy (Shields *et al*, 2021).

Diet is intimately connected with the microbial community (diversity and abundance) in humans especially with the gut microbiota, and dietary changes could contribute to the microbial profiles during diseased conditions. Epidemiological data shows that high‐fiber diets are related to a lower cancer incidence and the anti‐cancer effect is significantly due to the formation of SCFAs from high‐fiber diet (Mirzaei *et al*, 2021). It has been shown that mice fed with probiotic supplementation increased the SCFA producing bacteria (propionate and butyrate) in the gut with the inhibition of lung metastasis of melanoma cells (Chen *et al*, 2021) implicating diet‐microbiome‐SCFA link. Similarly, evidences showed that physical activity such as exercise can alter the composition of the gut microbiota, and eventually increase the SCFA production as an additional substrate for metabolism (Song & Chan, 2019). While propionate and butanoate are produced in the gut with the highest levels in the large intestine (30–150 mM), there is no consensus report on the range of SCFAs concentration in the lungs or about its substrate source. SCFAs have been detected in the sputum from 0.1 to 5 mM concentration possibly indicating the availability of SCFAs in the lungs and suggesting an existence of a gut‐lung axis connection (Ghorbani *et al*, 2015). In line with this, our data on the propionate's enhancement of lung‐specific epithelial features and the response of oral administration of propionate opposing lung tumorigenesis in mice suggest the possibility of propionate production from lung‐resident microbiome, which is known to contribute to cancer formation (Jin *et al*, 2019). Therefore, the present data call for future investigations addressing the role of SCFAs‐producing bacteria in protecting from a more aggressive (EMT‐driven) type of NSCLC.

Despite some anti‐metabolic drugs, such as metformin, have shown anti‐EMT effects in preclinical studies of lung cancer (Li *et al*, 2014), their clinical benefits are limited (Skinner *et al*, 2021) and there are currently no drugs approved for reducing *in vivo* EMT (Chen *et al*, 2020). Since propionate is a biotic compound and a safe food ingredient with therapeutic ability (Rangan & Mondino, 2022), the beneficial effects reported here make propionate a promising avenue for further clinical exploration as a treatment option for NSCLC patients. Alternative strategies like consuming fiber‐enriched diet and physical activities resulting in high SCFA content in the system could offer a realistic approach in cancer prevention and treatment.

In summary, the present study identified the therapeutic potential of propionate in inhibiting EMT and its associated features in lung cancer. Propionate mediates epigenetic reprogramming by increasing transcriptionally active chromatin marks to reinforce epithelial gene expression pattern. This ability of facilitating lung‐specific epithelial identity may have potential clinical benefits in cancer and in other conditions in which EMT is determinant like in fibrotic disorders.

## Materials and Methods

### Cell culture

Lung cancer cell lines were purchased from ATCC and KPL cell line was derived from lung tumorigenic mice intubated with adeno‐associated virus (AAV‐KPL) virus. A549 and KPL cell lines were cultured in DMEM high glucose medium (Gibco). SKMES1, CALU‐1, H1299 and NCI‐H520 cell lines were cultured in RPMI‐1640 medium (Gibco), and NCI‐H23 was cultured in RPMI‐1640 with 1 mM sodium pyruvate (Sigma). All media were supplemented with 2 mM of L‐glutamine (Gibco), 100 U/ml of Penicillin–Streptomycin (Gibco), and 10% Fetal bovine serum (Gibco). All cell lines were authenticated by STR profiling and tested regularly for mycoplasma contamination. Cell lines were maintained at 37°C with 5% CO_2_ level in a humidified incubator.

### Drug treatment

For drug treatment studies, around 1.5 × 10^5^ cells were seeded for the treatment in a 6‐well cell culture dish. After 24 h of seeding, cells were treated with the appropriate inhibitors in the presence or absence of sodium propionate (Sigma, P1880). Inhibitors used in the study were: Recombinant Human TGF‐β1 protein (R&D Systems, 240‐B‐002), SAHA (Sigma, SML0061), HAT inhibitor VII, CTK7A (Sigma, 382115), A‐485 (MedChemExpress, HY‐107455), AZD3965 (MedChemExpress, HY‐12750), Syrosingopine (Sigma, SML1908), AR420626 (Sigma, SML1339), mocetinostat (MedChemExpress, HY‐12164), MI192 (Sigma, SML1451), GW9662 (Sigma, M6191), Troglitazone (Sigma, T2573), GSK126 (MedChemExpress, HY‐13470), and GSK343 (MedChemExpress, HY‐13500). Sodium butyrate (303410), sodium acetate (S2889), propionic acid‐1‐^13^C (282448), aniline (242284), N‐(3‐Dimethylaminopropyl)‐N′‐ethylcarbodiimide hydrochloride (8009070001), succinic acid (14079) and 2‐mercaptoethanol (63689) were purchased from Sigma. Matrigel matrix (356230) was from corning.

### EDTA detachment

For experiments involving EDTA‐mediated detachment, cells were harvested by washing twice with 1× PBS without Ca^2+^/Mg^2+^ (Lonza), incubated with 5 ml of 0.5 mM EDTA (Lonza) at 37°C for 2 min to detach the cells, mixed well to obtain single cell suspension with equal volume of ice‐cold 1× PBS containing Ca^2+^/Mg^2+^ (Sigma), and centrifuged at 800 rpm for 3 min at 4°C. Cells were then mixed with 5 ml of warm media and incubated at 37°C for 1 h with frequent agitation to re‐express E‐cadherin. After 1 h of incubation, cells were then washed twice with 1× PBS containing Ca^2+^/Mg^2+^ and centrifuged at 800 rpm for 4 min to proceed for the downstream experimental purposes.

### Lung experimental metastasis model

NSG mice strain was purchased from Jackson Laboratory for the experimental lung metastasis study. Around 1 × 10^6^ cells of A549‐pFUL2G or SKMES1‐pFUL2G were seeded in a 10‐cm dish and after 24 h of seeding, cells were treated with sodium propionate (5 mM) for 3 days. The cells were then harvested with EDTA detachment method. Around 500,000 cells were resuspended in 100 μl of 1× PBS containing Ca^2+^/Mg^2+^ and injected in the tail vein of female NSG mice of 10 weeks. Lung colonization was examined using *in vivo* imaging system (IVIS, Perkin Elmer) in the anesthetized mice by intraperitoneal injection of 3 mg/ml of D‐luciferin (Cayman Chemicals, 14682) as substrate for luciferase enzyme produced by pFUL2G cells in the lungs, and bioluminescence signal was recorded as radiance (p/s/cm^2^/sr) and reported the overall bioluminescence signal as total flux (p/s). Sample size was chosen based on the previous similar experiments (Siddiqui *et al*, 2021).

### CRISPR/Cas9‐mediated lung tumorigenesis in mice and SP administration in drinking water

Ultra‐purified recombinant adeno‐associated virus (AAV‐KPL) was produced and obtained from VectorBuilder Inc., USA from the AAV:ITR‐U6‐sgRNA(Kras)‐U6‐sgRNA(p53)‐U6‐sgRNA(Lkb1)‐pEFS‐Rluc‐2A‐Cre‐shortPA‐KrasG12D_HDRdonor‐ITR vector (addgene, #60224). Adult male and female in‐house bred C57BL/6 strain constitutively expressing Cas9 (B6J.129(B6N)‐*Gt(ROSA)26Sor* ^tm1(CAG‐cas9*,^ ^*‐EGFP)Fezh*^/J) mice were housed under standard conditions and grouped normalizing for gender and age (7–14 weeks). Pre‐administration of sodium chloride (SC, 150 mM) or sodium propionate (SP, 150 mM) in mice drinking water was started 1 week before lung delivery of the virus and continued until the end of the study. Drinking water was changed twice a week with freshly prepared compounds. AAV‐KPL virus was oropharyngeally delivered to mice resulting in a B/6.*Kras* ^*G12D/G12D*^ *p53* ^*Δ/Δ*^ *Lkb1* ^*Δ/Δ*^ genotype (referred to as KPL mice). For oropharyngeal virus delivery, mice were anesthetized using isoflurane, and 3 × 10^11^ viral units per mouse were administered dissolved in 25 μl of 0.9% sodium chloride. Mice were sacrificed at humane endpoint scoring breathing patterns and body weight loss. Mice were euthanized by cervical dislocation to collect lungs and lymph nodes for H&E staining, and mice survival was recorded. Presence of lung tumors and lymph node metastases was evaluated by a trained pathologist at the Odense University hospital (KEO) in a blind fashion. Animal protocols were approved by the Danish Animal Welfare Authority (approval 2020‐15‐0201‐00607, CRISPR‐Cas9 model) and by the Institutional Animal Care and Use Committee of the Regierung von Unterfranken (NSG model).

### Flow cytometry

A549 cells treated with SP (5 mM) for 3 days in 10‐cm dish were detached using EDTA detachment protocol and around 5 × 10^5^ cells were washed once with 3 ml of blocking buffer (2% BSA (Roth, 8076.4) in 1× PBS containing Ca^2+^/Mg^2+^), and centrifuged at 800 rpm for 3 min. Cells were then stained with 2.5 μl of PE anti‐human E‐cadherin (50 μg/ml; Biolegend, 324106) or matched concentration of PE Mouse IgG1, κ Isotype ctrl (FC) (200 μg/ml; Biolegend, 400113) as per manufacturer's protocol (Biolegend) for 1 h in dark at room temperature with frequent agitation. Cells were then washed once with blocking buffer and resuspended in 500 μl FACS resuspension buffer (2% FBS with 5 mM EDTA in 1× PBS) containing 5 μl of DAPI (20 μg/ml), and were immediately analyzed using CytoFLEX (Beckman Coulter) and the analysis was performed using FlowJo software v10.6.

### Immunofluorescence

Around 1 × 10^5^ cells were seeded on a glass coverslip for 3 days SP treatment in a 12‐well cell culture plate. After 3 days SP treatment, cells were washed with 1× PBS followed by the addition of ice‐cold methanol (Sigma) and incubated the coverslips for 20 min at room temperature. Cells were then blocked with blocking buffer (3% BSA in 1× PBS) at room temperature for 1 h. Cells were incubated overnight at 4°C with the primary antibodies. Primary antibodies with the dilutions used in the study were: E‐cadherin (1:250, mouse, Cell Signaling, 14472), EPCAM (1:100, rabbit, Invitrogen, PA5‐29634) and ZO‐1 (1:150, goat, abcam, ab190085) prepared in the blocking buffer. Next day, coverslips were washed with 1× PBS and incubated with the corresponding fluorochrome‐conjugated secondary antibodies for 1 h in dark at room temperature. Secondary antibodies with the dilutions used in the study were: anti‐mouse Alexa Fluor 488 conjugate (1:250, ThermoFisher Scientific, A28175), anti‐rabbit Alexa Fluor 488 conjugate (1:200, Cell Signaling, 4412S) and anti‐goat Alexa Fluor 488 conjugate (1:200, abcam, ab150129) prepared in the blocking buffer. After secondary antibody incubation, cells were washed with 1× PBS, and the coverslips were mounted on a glass slide using FluoroSheild with DAPI containing mounting medium (VWR, F6057). Cells were then visualized using Leica DM5500B fluorescence microscope or with Nikon Widefield Ti‐2, and the images were acquired either using Leica Application Suita‐X software or with NIS‐Elements Viewer 5.21.

### Immunohistochemistry

IHC analysis was performed in all cases as follows: 5 mm‐thick serial paraffin sections from paraffin blocks were processed using an automated platform (Ventana BenchMark Ultra, ROCHE) with a primary antibody against EPCAM (polyclonal, 1:1,000 dilution, Thermo Fisher Scientific, PA5‐29634). Immunoreactivity was evaluated by H‐score, calculated as follows: H‐score = ΣPi (i + 1), where i represents the intensity of staining (0–3+) and Pi stands for the percentage of stained tumor cells (0–100%). IHC were scored by trained pathologists in a blind fashion.

### Western blot

Cells were harvested for protein using Pierce RIPA lysis buffer (Thermo Scientific) containing 1× Halt Protease & Phosphatase inhibitor cocktail (Thermo Scientific) and estimated the proteins using Pierce BCA Protein Assay Kit as per manufacturer's protocol (ThermoFisher Scientific). 15–30 μg of proteins were resolved in 8% SDS–PAGE separating gel and transferred the proteins from the resolved gel to a PVDF membrane (Thermo Scientific). Membranes were blocked with the blocking buffer (5% non‐fat dried milk powder or 3% BSA prepared in 1× TBS‐T) for 1 h at room temperature, and then incubated overnight at 4°C with the primary antibodies. Primary antibodies with the dilutions used in the study were: E‐cadherin (1:5,000, mouse, Cell Signaling, 14472), ZEB1 (1:2,000, rabbit, Sigma, HPA027524), γH2AX (1:2,000, rabbit, Cell Signaling, 9718S), GRHL1 (1:1,000, rabbit, abcam, ab111582), OVOL2 (1:1,000, mouse, abcam, ab169469), H3K27ac (1:3,000, rabbit, abcam, ab177178), H3K27me3 (1:3,000, mouse, abcam, ab6002), H3 (1:10,000, rabbit, Cell Signaling, 9715S), Acetyl‐CBP‐Lys1535/p300‐Lys1499 (1:1,000, rabbit, Cell Signaling, 4771S), TUBA4A (1:10,000, mouse, Sigma, T6199), ZEB2 (1:1,000, rabbit, abcam, ab138222), TWIST1 (1:500, mouse, abcam, ab50887), SNAIL (1:1,000, rabbit, abcam, ab216347), SLUG (1:1,000, rabbit, abcam, ab27568), NNMT (1:2,000, mouse, abcam, ab119758), PCCA (1:1,000, rabbit, abcam, ab187686), PCCB (1:1,000, rabbit, Sigma, HPA036940) and β‐Actin HRP conjugated (1:10,000, Cell Signaling, 12262). Next day, the membranes were washed with 1× TBS‐T and incubated the membrane blots in secondary antibody dilution (Southern Biotech). Secondary antibodies conjugated with HRP with the dilutions used in the study were: Goat Anti‐mouse IgG1‐HRP (1:10,000, Southern Biotech, 1071‐05), Goat Anti‐rabbit IgG‐HRP (1:10,000, Southern Biotech, 4030‐05), Goat Anti‐mouse IgG2b‐HRP (1:10,000, Southern Biotech, 1091‐05) and Goat Anti‐mouse IgG3‐HRP (1:10,000, Southern Biotech, 1101‐05). Protein bands were detected using Pierce ECL western blotting solution (Thermo Scientific) with the development of X‐ray films (Thermo Scientific). Bands were quantified using ImageJ quantification tool by using β‐Actin to normalize the target protein levels, and the relative expression was indicated as fold change of the target protein in comparison to the control of the respective time points.

### Cell proliferation assay and dose response curve analysis

Approximately 1,000 cells per well were seeded in low density (5–10% confluence) in a 96‐well plate in triplicates, and after 24 h, cells were treated with SP at 5 mM. For dose response curve analysis with cisplatin, 1,000 cells/well were seeded and pre‐treated with SCFAs (SP or SB) for 48 h followed by cisplatin (Tocris, 2251) treatment in a dose dependent manner for 72 h. Plates were then incubated at 37°C in IncuCyte S3 (Sartorius) and real‐time live‐cell proliferation analysis was performed at regular intervals of 4 h for 12 days with phase contrast image acquisition mode using Incucyte S3 software. To quantify cell death, Cytotox Green (Sartorius) was mixed in the media and the image acquisition mode was set to green channel. Proliferation was plotted as percent confluence over time. Dose responsive curve was generated using a non‐linear fit of log(inhibitor) vs normalized response using GraphPad with a two‐way ANOVA analysis to obtain the significance between the conditions.

### Migration assay

A549 cells were seeded in 96‐well plate of around 3,000 cells per well and treated with SP alone or pre‐treated with SP (5 mM) for 48 h followed by TGF‐β1 (2 ng/ml) for 24 h. Once the cells reached confluency, scratch was made using Incucyte 96‐well Woundmaker Tool (Essen BioScience), washed the cells with 1× PBS to remove the debris, and incubated the plates at 37°C in IncuCyte S3. Relative wound density was measured using the integrated quantification module for scratch wound at regular intervals of 4 h for 3 days in Incucyte S3.

### Spheroid assay

Around 2,500 cells/well were seeded in a 96‐well ultra‐low attachment plate and centrifuged the cells at 300 rpm for 10 min at 20°C. The plate was then incubated at 37°C for 3 days until the spheroids attained a desired size of 200–500 μm in diameter. Then, the spheroids were treated with sodium propionate (5 mM) or with TGF‐β1 (2 ng/ml). The plate was then pre‐cooled on ice followed by the addition of matrigel matrix to the spheroids, centrifuged at 300 rpm for 5 min at 4°C and allowed the matrigel to polymerize at 37°C incubator for 30 min. The spheroids were then monitored and measured the brightfield area of the spheroids using real‐time spheroid invasion assay at regular intervals of 4 h for 5 days in Incucyte S3.

### ECM cell adhesion assay

ECM cell adhesion assay was performed as per manufacturer's instructions (Merck). Briefly, 70,000 cells per well were seeded in the wells of the strips coated with seven different ECM proteins. Cells were allowed to attach to the surface for 1 h at 37°C incubator, and then gently washed twice with the provided assay buffer. After washing, 100 μl of the cell stain solution was added to each well, and allowed to incubate for 5 min followed by cell stain removal by washing the wells thrice with deionized water. Wells were then air dried, added with 100 μl of extraction buffer, and incubated on a rotating shaker until the stain was completely solubilized. The absorbance was then measured at 540 nm in a microplate reader.

### Quantitative image‐based cytometry (QIBC)

A549 cells of around 2.5 × 10^5^ cells per well were seeded on a round glass coverslip in a 6‐well plate. After 24 h of seeding, cells were pre‐treated with SP at 5 mM for 24 h followed by the treatment with cisplatin in a dose dependent concentration for around 6 h. Cells were then mixed with 10 μM of EdU in each well and the plates were incubated for last 20 min before fixation. Cells were fixed with 4% formaldehyde stabilized with 0.5–1.5% methanol (VWR, 9713) for 12 min at room temperature, washed with ice‐cold 1× PBS, and further incubated with 1× PBS containing 0.2% Triton X‐100 (Sigma, X100) for 5 min. In case of assessing chromatin‐based proteins, cells were pre‐extracted for the chromatin‐bound proteins by washing the treated cells with ice‐cold 1× PBS followed by incubating the cells with ice‐cold 1× PBS containing 0.2% Triton X‐100 for 90 s. Cells were then washed again with ice‐cold 1× PBS and fixed the cells with 4% formaldehyde for 12 min at room temperature. After fixation, Click‐iT Edu reaction was performed using A647‐Azide (ThermoFisher Scientific, A10277) through a copper‐catalyzed click chemistry for 30 min in dark and washed once with 1× PBS containing 0.1% Tween20 (Sigma, P1379), and thrice with 1× PBS. Coverslips were then incubated with primary antibodies for 1 h in dark at room temperature and washed with 1× PBS containing 0.1% Tween20. Primary antibodies with the dilutions used were: H3K27ac (1:500 rabbit, abcam, ab177178), H3K4me3 (1:1,000, rabbit, abcam, ab8580), HP1 (1:500, mouse, Santa Cruz, SC515341), H3S10ph (1:2,000, mouse, abcam, ab14955), γH2AX (1:2,000, mouse, Biolegend, 613402), RAD51 (1:1,000, mouse, abcam, ab213), and 53BP1 (1:2,000, mouse, Millipore, Mab3802). Next, cells were incubated with the secondary antibodies containing DAPI (0.5 μg/ml) as nuclear counterstain for 30 min in dark at room temperature. Secondary antibody conjugates of Alexa Fluor 488 or 568 for mouse (A‐11029 or A‐11031) and rabbit (A‐11034 or A‐11036) from ThermoFisher Scientific in 1:2,000 dilutions were used. Coverslips were then washed once with 1× PBS containing 0.1% Tween20, and thrice with 1× PBS. Coverslips were rinsed with MilliQ water, air dried and mounted onto the glass slide using Mowiol 4–88 mounting medium. QIBC was performed as previously described (Somyajit *et al*, 2017). Briefly, images were acquired with a ScanR inverted microscope high‐content screening station (Olympus) equipped with wide‐field optics, air objective, fast excitation and emission filter‐wheel devices for DAPI, FITC, Cy3, and Cy5 wavelengths, an MT20 illumination system, and a digital monochrome Hamamatsu ORCA‐Flash 4.0LT CCD camera. Images were acquired in an automated fashion with the ScanR acquisition software (Olympus, 3.2.1). 49–81 images were acquired containing at least 5,000 cells per condition. Acquisition times for the different channels were adjusted for non‐saturated conditions in 12‐bit dynamic range, and identical settings were applied to all the samples within one experiment. Images were processed and analyzed with ScanR analysis software. First, a dynamic background correction was applied to all images. The DAPI signal was then used for the generation of an intensity‐threshold‐based mask to identify individual nuclei as main objects. This mask was then applied to analyze pixel intensities in different channels for each individual nucleus. For analysis of sub‐nuclear foci, additional masks were generated by segmentation of the respective images into individual spots with intensity‐based or spot‐detector modules included in the software. Each focus was defined as a sub‐object, and this mask was used to quantify pixel intensities in foci. After this segmentation of objects and sub‐objects, the desired parameters for the different nuclei or foci were quantified, with single parameters (mean and total intensities, area, foci count, and foci intensities) as well as calculated parameters (sum of foci intensity per nucleus). These values were then exported and analyzed with TIBCO Spotfire Software, version 11.1, to quantify absolute, median, and average values in cell populations and to generate all color‐coded scatter plots. Within one experiment, similar cell numbers were compared for the different conditions (at least 4,000–5,000 cells), and for visualization low x‐axis jittering was applied (random displacement of objects along the x axis) to make overlapping markers visible.

### siRNA transfection

Reverse transfection of propionate metabolism‐specific siRNAs was performed using Lipofectamine RNAiMAX transfection reagent as per manufacturer's protocol (ThermoFisher Scientific). Briefly, transfection complex was prepared by mixing 50 nM of SMARTPool ON‐TARGETplus Human siRNAs (horizon, PerkinElmer) with 3 μl of Lipofectamine RNAiMAX transfection reagent (ThermoFisher Scientific) in 200 μl Opti‐MEM (ThermoFisher Scientific) and incubated the complex for 15 min at room temperature. 200 μl of transfection complex was then mixed with 800 μl of A549 cells (3 × 10^5^ cells) while cell seeding in a 24‐well plate. After 48 h of siRNA transfection, cells were treated with SP (5 mM) for 24 h, and the cells were harvested for western blot analysis of EMT markers.

### RNA isolation, cDNA synthesis, and real‐time PCR

A549 cells were harvested using 700 μl of QIAzol reagent (Qiagen). Mouse lung tumor tissue was collected in RNA*later*™ solution (AM7020, Invitrogen) and stored at −20°C. On the day of RNA isolation, tissues were blotted away of excess RNA*later*™ solution and homogenized the tissue to fine pieces in QIAzol reagent using 1.5 ml pestle (Fisher Scientific). Total RNA was then isolated as per the manufacturer's protocol using miRNeasy kit (Qiagen) and eluted the RNA in 40 μl of nuclease‐free water (VWR). 500 ng of isolated RNA was converted to cDNA using Tetro cDNA synthesis kit (meridian BioScience) with random hexamers using the routine protocol. Real‐Time PCR was performed using 2× Taqman Universal master mix II, no UNG buffer (Applied Biosystems). Briefly, 5 μl of synthesized cDNA was mixed with 1× Universal Taqman master mix buffer along with 1× Taqman probes and the reaction was set in Roche 96‐well system with pre‐incubation at 95°C for 10 min followed by 40 cycles of 95°C for 15 s and 60°C for 1 min in a FAM acquisition mode. Real‐time qPCR analysis was performed with Ct values obtained from LightCycler 96 SW 1.1 (Roche) and determined the folds as test over control using the ΔΔCt method.

### RNA‐sequencing

Total RNA was isolated from A549 cells treated with SP for 3 h, 24 h, 3 days and 12 days. Similarly, total RNA was isolated from lung tumor tissues of KPL mice administered with sodium chloride or sodium propionate. 1 μg of total RNA was diluted in 25 μl of nuclease‐free water. Sequencing libraries were constructed using NEBNext Ultra RNA Library Prep Kit for Illumina according to the manufacturer's protocol (NEB) and paired‐end sequencing was performed with NovaSeq 6000 platform (Illumina).

RNA‐Seq analysis was performed by subjecting the Fastq files for the QC analysis using FastQC followed by aligning the reads to the reference genome GRCh38 release 105 with the respective human gene annotation file using STAR aligner v2.7.9a. The reference genome of GRCm39 release 108 was used for the mouse genome alignment. The aligned files were then used for counting the raw reads using the Featurecounts function in Rsubread v2.10.5 package in R 4.2.1. Raw count reads were then used for the differential gene expression analysis with DESeq2 v1.36.0 package in R with an adjusted *P*‐value < 0.0001 and fold change of 2. Mean normalized values from DESeq2 were used for the expression analysis of gene‐set between conditions.

### Single cell RNA‐sequencing

A549 cells for single cell RNA sequencing (scRNA‐seq) was performed using 10× Genomics guidelines at the Institute of Human Genetics, Friedrich‐Alexander‐University of Erlangen‐Nürnberg, Erlangen, Germany. Around 6,000 cells were read with approximately 25,000 reads per cell. Briefly, the filtered_feature_bc_matrix data containing barcodes, features and matrix obtained from cellranger‐4.0.0 pipeline were further analyzed by Seurat v4.2.1 package in R. Quality control followed by pre‐processing of the file was performed with the filtering of the data by removing cells with less than 500 genes, UMIs with < 2,500 and > 45,000 total number of molecules, more than 10% mitochondrial genes and > 5% largest gene. Log_10_ genes per UMI was set at > 0.85 for filtering. Counts were then normalized for the library size and identified 2,000 variable genes to perform PCA. Variations including mitochondrial genes, cell cycle genes, and genes & UMI counts per cell were all regressed out for the downstream analysis. First 25 principal components from PCA of the data were selected to determine the cell clusters using Lovain algorithm with a resolution of 0.4 and visualized the cell clusters using tSNE dimensionality reduction method. Marker genes were then identified for each cluster. AddModuleScore function in Seurat was applied to identify the gene‐set activity metrics in each cell clusters for epithelial, mesenchymal and SP regulated gene‐sets.

### Gene enrichment analysis

Gene set enrichment analysis (GSEA) was performed for the patient samples categorized as low and high based on the median of the *z*‐score activation for SP‐regulated genes with the gene ranks calculated using signal2noise metric. For the association of SP‐regulated gene‐sets with the hallmark adherens junction or hallmark adherens surface, continuous label of SP gene‐set *z*‐score activation in the expression profile using the gene ranks calculated using Pearson ranked gene metric was employed. Significance was set to nominal *P*‐value < 0.05 and FDR < 0.25. Gene enrichment analysis using representational overlap analysis was carried out using Enrichr web tool for SP‐regulated genes identified from RNA‐Seq analysis or for the cell cluster marker genes identified from scRNA‐seq of A549 cell lines. L1000CDS^2^ search engine was used to identify the small molecule mimics of SP‐regulated gene signature identified from RNA‐Seq.

### ChIP‐sequencing

H3K27ac ChIP‐seq was performed on SP treated A549 cells as described previously (Siersbæk *et al*, 2020). In brief, cells were double crosslinked in 2 mM DSG and 1% FA before harvesting of the cells by scraping from the culture dish. Cell pellets were washed and sonicated until fragment sizes ranged between 200 and 500 bp. Sonicated chromatin was then immunoprecipitated overnight using Dynabeads protein A (Dynabeads, 10002D) coated with the antibody of interest. Next day, the beads were washed six times in cold RIPA buffer, and the DNA was decrosslinked and purified using standard phenol‐chloroform purification. Purified DNA was submitted to NGS library preparation using NEBNext Ultra II DNA Library Prep Kit (New England BioLabs, E765) and sequenced with NovaSeq 6000 (Illumina) platform to reach approximately 25 million paired‐end reads per sample. Paired‐end reads were aligned to the human genome using Hisat2 v2.1.0. Duplicated reads from PCR amplification were removed using samtools before peaks were called with the default setting in MACS2. Only peaks called within all replicates were considered as consensus peaks. Homer was used to visualize heatmaps and for counting normalized tag counts within promoter regions.

### SingScore

RNA‐seq normalized counts from DESeq2 were ranked for each condition and SingScore was used to calculate gene‐set enrichment scores as described previously (Foroutan *et al*, 2018). The calculated EMT score represents the difference between the mesenchymal and the epithelial gene‐sets obtained from previous report (Tan *et al*, 2014).

### Histone extraction and digestion

Histones were extracted from frozen cell pellets by acid extraction. Briefly, nuclei were isolated using nuclear isolation buffer (15 mM Tris–HCl pH 7.5, 60 mM KCl, 11 mM CaCl_2_, 5 mM NaCl, 5 mM MgCl_2_, 250 mM sucrose, 1 mM dithiothreitol, 10 mM sodium butyrate and 0.1% Igepal) supplemented with protease (cOmplete™ Protease Inhibitor Cocktail, Roche) and phosphatase (PhosSTOP, Roche) inhibitors. Nuclei were pelleted by centrifugation (1,000 *g* – 5 min) and washed twice with nucleus isolation buffer without Igepal. Histones were extracted by resuspending the pellet in 0.2 N H_2_SO_4_ for 1 h, and the supernatant was collected after centrifugation (20,000 *g* – 5 min). Histones were precipitated overnight after adding trichloroacetic acid to a final concentration of 20%. Histones were pelleted by centrifugation (20,000 *g* – 15 min) and washed once with 0.1% HCl in acetone and then twice with pure acetone. After the last wash, histones were air‐dried and resuspended in H_2_O. All steps were performed at 4°C. Purity of histones was evaluated by SDS–PAGE and protein concentration was determined using Qubit™ Protein Assay Kit (Invitrogen). Histones were digested using the Propionylation‐PIC method). For each reaction, 5 μg of histones were diluted in H_2_O to a total volume of 9 μl. pH was adjusted with 0.4 μl of 5% NaOH and buffered with 1 μl of 1 M EPPS. Before propionylation, cysteine residues were reduced and alkylated with 12.5 mM dithiothreitol and 25 mM iodoacetamide, respectively. Propionylation reaction was performed by adding 1.5 μl of 1% propionic anhydride in acetonitrile for 2 min at RT and was quenched with 1.5 μl hydroxylamine 80 mM. Histones were digested overnight at 37°C using 0.1 μg of trypsin. For derivatization, peptides were incubated at 37°C for one additional hour with 4.5 μl of 1% phenylisocyanate in acetonitrile. Histone peptides were desalted using homemade C18‐stage‐tips.

### LC–MS/MS analysis

Stage‐tip desalted histone peptides were resuspended in HPLC solvent A (0.1% formic acid in H_2_O). LC–MS/MS analysis was performed with ~500 ng peptides using a nano‐flow HPLC system (EASY‐nLC 1000, ThermoScientific) coupled with an Orbitrap mass spectrometer (Exploris480, ThermoScientific). Peptides were loaded onto a ~ 4 cm long, 100 μm ID precolumn packed with 5 μm C18 particles and separated onto a 18 cm long, 75 μm ID analytical column packed with 3 μm C18 particles. Peptides were separated at a flow rate of 250 nl/min by a linear gradient from 2% solvent B (0.1% formic acid in 95% acetonitrile) to 45% solvent B over 40 min followed by a ramp to 100% solvent B in 3 min and stabilization at 100% solvent B during 7 min (total run time: 50 min). Full mass range spectra were acquired at a resolution of 120,000 (at *m/z* 200) with a scan range of *m/z* 300–2000, and the 15 most intense precursors were selected for MS/MS. Fragmentation was performed by high energy collisional dissociation (HCD) with 30% normalized collision energy. MS/MS spectra were acquired at a resolution of 30,000 (at *m/z* 200).

### Epiproteomic data analysis

Thermo .raw files were processed with Proteome Discoverer 2.5 (ThermoScientific) using MASCOT and Percolator with label‐free quantification node. Spectra were searched with MASCOT against a human histone database (Swiss‐Prot reviewed, downloaded from www.uniprot.org), using ArgC as digestion enzyme with 1 missed cleavage accepted. Full MS and MS/MS tolerances were set to 5 ppm and 0.05 Da, respectively. Propionylation of lysines and alkylation of cysteines with iodoacetamide were mentioned as static modifications. Several dynamic modifications were considered for the search: propionylation of protein N‐terminus, derivatization of peptide N‐terminus by phenylisocyanate, acetylation of protein N‐terminus, methylation/dimethylation/trimethylation of lysines and phosphorylation of serines and threonines. The identity of each peak was verified by hand using retention times and MS/MS spectra. The area under the curve (AUC) was calculated by the software for each specific proteoform and was normalized to the global amount of peptides with the same primary sequence. To identify changes in histone PTM abundances, log_2_ fold‐change values of SP‐treated cells compared to control were calculated for each point.

### Detection of intracellular propionate by LC/MS

Quantification of intracellular propionate levels was carried out essentially as described (Chan *et al*, 2017). Briefly, around 1.5 × 10^5^ cells of A549 cells were seeded in triplicates and treated with sodium propionate for 72 h before cell pellet collection and metabolite extraction. Cell pellets were resuspended in 400 μl extraction solvent consisting of acetonitrile (ACN) and water (1:1 v/v) containing 50 μM [^13^C_1_]‐propionic acid and shook at 900 rpm and 4°C for 10 min. Samples were centrifuged at 21,000 *g* at 4°C for 15 min, and the supernatant was harvested (100 μl). Supernatants were mixed with aniline (final concentration 10 mM) and EDC (final concentration 5 mM) for derivatization of short‐chain fatty acids at 4°C for 120 min. The derivatization reaction was quenched by adding succinic acid (final concentration of 18.35 mM) and 2‐mercaptoethanol (final concentration of 4.58 mM) and incubated the samples at 4°C for 120 min.

LC/MS analysis was performed using an Agilent 1290 LC coupled to a 6530b qTOF MS (Agilent). Water and HPLC‐grade isopropanol, both containing 0.1% formic acid were the mobile phases A and B, respectively. Chromatographic separation was carried out with a Zorbax RRHD Eclipse Plus C18 column (2.1 mm × 150 mm, i.d., 1.8 μm from Agilent maintained at 40°C) at a flow rate of 0.35 ml/min using the following gradient: 15% B (0–2 min), 15–33% B (2–6 min), 33–34% B (6–7.5 min), 34–36% B (7.5–12 min), 100% B (12–13 min) and 15% B (13–15 min). Autosampler was kept at 4°C. Mass spectrometry was operated in positive ion electrospray ionization mode. Data acquisition and processing were performed using Agilent MassHunter Workstation Software ‐ LC/MS Data Acquisition (B.09.00) and Agilent MassHunter Workstation Software ‐ Profinder (ver. 10.0). Intracellular levels of propionate were reported as the ratio between the endogenous ^12^C‐propionate and the ^13^C‐labeled internal standard.

### Gene‐sets source

Metabolic process associated gene‐sets, hallmark gene‐sets, and EMT associated gene‐sets were collected from MSigDb v6.2. In total 335 metabolic process associated gene‐sets were collected (135 of KEGG and 200 of REACTOME gene‐sets). Metabolic process associated gene‐sets collectively represent diverse metabolic processes representing carbohydrates, steroids, amino acids, vitamins, lipids, fatty acids, catabolic and anabolic, enzymatic activities including coenzymes or co‐factors, hormones, nucleotide, biopolymer or macromolecular, alcohol, amine, drug, organic, inorganic and acyl chain related metabolisms. It also includes EMT associated gene‐sets as a positive control for the integrative functional genomic analysis.

### Metabolic process activation analysis

Metabolic process activity score was calculated as described previously (Ramesh & Ganesan, 2016). Fold expression values (log_2_) for each gene in the tumor samples was calculated relative to the median expression value across the tumor samples as reference. Next, mean and standard deviation of the fold expression in the whole gene expression profile for each tumor sample was calculated. Similarly, mean was calculated from the fold expression value for the metabolic gene‐sets for each tumor samples by extracting the fold expression values for all genes in the gene‐set. The metabolic process activity score (*z*‐score) was calculated by subtracting the mean fold expression value of the whole gene expression from the mean fold expression value of the metabolic gene‐set and divided the obtained value with the standard deviation of the whole fold expression value. Finally, the derived activity score was normalized by multiplying the obtained score with the square root of the number of genes in the gene‐set to obtain a normalized *z*‐score activity score for the metabolic process. Metabolic process activity scores were then associated with EMT activation score by Pearson's correlation method in R. A meta‐correlation was performed using metacor.DSL function in R wherein the number of samples in each dataset was included for the analysis and significantly associated gene‐sets were identified with meta‐*r* > 0.3 and meta‐*r* < −0.3 as positively and negatively associated, respectively, with meta‐*P*‐value < 0.05. Network visualization of the associated pathways was carried out using VisANT software. Heatmap representation of the metabolic process activity levels was carried out using R.

### Survival analysis

Lung cancer gene expression profiles were obtained from GEO as normalized values and for TCGA profiles from cbioportal platform as mRNA *z*‐score values. SP gene‐set score was calculated similar to the *z*‐score activation with the difference of up‐regulated and down‐regulated activity scores in the patient samples. Samples were then categorized as SP‐low and SP‐high based on the median of the SP gene‐set activity score, and generated survival curve based on Kaplan–Meier estimate analysis. Significant difference between the two categorized samples was estimated using log‐rank test in R software.

### Statistical analysis

All statistical analysis was performed using GraphPad Prism9 software or using R v4.2.1 software. Presence of lung tumors and lymph node metastases was evaluated in a blinded fashion. Similarly, IHC scores were evaluated in a blinded manner. Mice were randomized based on the gender and age for the CRISPR/Cas9 mediated lung tumorigenesis. Outlier in the group for the lung experimental metastasis model was assessed using Grubbs' test with the alpha set at 0.05. Significance was calculated using the unpaired *t*‐test between the groups.

## Author contributions

**Vignesh Ramesh:** Conceptualization; data curation; software; formal analysis; validation; investigation; visualization; methodology; writing – original draft; writing – review and editing. **Paradesi Naidu Gollavilli:** Data curation; formal analysis; validation; investigation; visualization; methodology. **Luisa Pinna:** Data curation; formal analysis; validation; investigation; visualization; methodology. **Mohammad Aarif Siddiqui:** Data curation; formal analysis; validation; investigation; visualization; methodology. **Adriana Martinez Turtos:** Data curation; formal analysis; validation; investigation; visualization; methodology. **Francesca Napoli:** Data curation; formal analysis; validation; investigation; visualization; methodology. **Yasmin Antonelli:** Data curation; formal analysis; validation; investigation; visualization; methodology. **Aldo Leal‐Egaña:** Data curation; formal analysis; validation; investigation; visualization; methodology. **Jesper Foged Havelund:** Data curation; formal analysis; validation; investigation; visualization; methodology. **Simon Toftholm Jakobsen:** Data curation; formal analysis; validation; investigation; visualization; methodology. **Elisa Le Boiteux:** Data curation; formal analysis; validation; investigation; visualization; methodology. **Marco Volante:** Data curation; formal analysis; validation; investigation; visualization; methodology. **Nils Joakim Færgeman:** Resources; validation; visualization; methodology. **Ole N Jensen:** Resources; validation; visualization; methodology. **Rasmus Siersbæk:** Resources; validation; investigation; visualization; methodology. **Kumar Somyajit:** Resources; data curation; formal analysis; validation; investigation; visualization; methodology. **Paolo Ceppi:** Conceptualization; resources; data curation; supervision; funding acquisition; validation; investigation; visualization; writing – original draft; project administration; writing – review and editing.

## Disclosure statement and competing interests

The authors declare that they have no conflict of interest.

## Supporting information



Appendix

Expanded View Figures PDF

PDF+

Source Data for Figure 1

Source Data for Figure 2

Source Data for Figure 3

Source Data for Figure 4

Source Data for Figure 5

Source Data for Figure 6

Source Data for Figure 7

## Data Availability

The datasets produced in this study are available in the following databases:
RNA‐Seq data: Gene Expression Omnibus GSE224740 (https://www.ncbi.nlm.nih.gov/geo/query/acc.cgi?acc=GSE224740) ChIP‐Seq data: Gene Expression Omnibus GSE224739 (https://www.ncbi.nlm.nih.gov/geo/query/acc.cgi?acc=GSE224739)
