# Supplementary material for: Propionate reinforces epithelial identity and reduces aggressiveness of lung carcinoma
Source: EMBO Mol Med. 2023 Sep 28;15(12):e17836. doi: 10.15252/emmm.202317836 (PMC10701619; doi:10.15252/emmm.202317836)
Supplement: Supplementary file 1 — Appendix [file EMMM-15-e17836-s007.pdf]

Propionate reinforces epithelial identity and reduces aggressiveness of lung carcinoma

Ramesh et al

Appendix - Table of Contents

Appendix Items	Title	Page No.
Appendix Table S1	List of gene expression profiles with sample numbers used for the integrative functional genomic analysis.	1
Appendix Table S2	List of 77 genes in pan-cancer EMT gene signature for mesenchymal and epithelial states obtained from Mak et al., 2016 used for the integrative functional genomic analysis.	2
Appendix Table S3	List of metabolic process gene-sets of KEGG and REACTOME collected from MSigDB for the integrative functional genomic analysis	3
Appendix Table S4	List of meta-correlation values with meta-pvalues of KEGG gene sets associated with pan-cancer EMT gene signature	8
Appendix Table S5	List of meta-correlation values with meta-pvalues of REACTOME gene sets associated with pan-cancer EMT gene signature	11
Appendix Table S6	List of marker genes in each cell type cluster identified from single cell RNA sequencing of parental A549 cells	15
Appendix Figure S1	Integrative genomic analysis identified negative association between propionate or butanoate with EMT in lung cancer gene expression profiles.	16
Appendix Figure S2	In vitro treatment effect of SCFAs, propionate or butanoate, in EMT marker gene expression	18
Appendix Figure S3	Effect of sodium propionate on lung tumorigenesis in KPL virus intubated Cas9-C57BL/6 mice administered orally with sodium propionate in drinking water.	19
Appendix Figure S4	SCFAs, propionate or butanoate, sensitizes the cells to cisplatin treatment in NSCLC cell lines.	21
Appendix Figure S5	Single cell RNA-seq of untreated parental A549 cell line shows cell clusters of epithelial and mesenchymal cell type enrichment.	23
Appendix Figure S6	Identification of possible mechanistic role of propionate’s action in E-cadherin’s increased expression.	25
Appendix Figure S7	Validation of histone marks H3K4me1/2/3 and H3K27ac in epithelial gene expression program by SP.	27

Appendix Table S1. List of gene expression profiles with sample numbers used for the integrative functional genomic analysis.

Profiles	No. of samples
GSE8894	138
GSE30219	293
GSE31210	226
GSE37745	196
GSE50081	181
GSE72094	442
**Total**	**1476**

Appendix Table S2. List of 77 genes in pan-cancer EMT gene signature for mesenchymal and epithelial states obtained from Mak *et al.,* 2016 used for the integrative functional genomic

Mesenchymal		Epithelial
ADAM12	VCAN	AP1G1
ADAMTS12	VIM	ATP8B1
ADAMTS2	ZEB2	CDH1
AEBP1		CDS1
ANGPTL2		CGN
ANTXR1		CLDN4
AXL		CNOT1
BNC2		CTNND1
CALD1		DYNC1LI2
CDH2		ERBB3
CMTM3		ESRP1
CNRIP1		ESRP2
COL10A1		F11R
COL1A1		GALNT3
COL1A2		GPR56
COL3A1		GRHL2
COL5A1		HOOK1
COL5A2		IRF6
COL6A1		MAP7
COL6A2		MARVELD2
COL6A3		MARVELD3
COL8A1		MYO5B
DACT1		OCLN
EMP3		PRSS8
FAP		SPINT1
FBN1		
FN1		
FSTL1		
GPC6		
GYPC		
HTRA1		
INHBA		
ITGA11		
LOXL2		
LRRC15		
MMP2		
MSRB3		
NAP1L3		
NID2		
OLFML2B		
PCOLCE		
PDGFRB		
PMP22		
POSTN		
SPARC		
SPOCK1		
SULF1		
SYT11		
THBS2		

Appendix Table S3. List of metabolic process gene-sets of KEGG and REACTOME collected from MSigDB for the integrative functional genomic analysis

KEGG_GLYCOLYSIS_GLUCONEOGENESIS
KEGG_CITRATE_CYCLE_TCA_CYCLE
KEGG_PENTOSE_PHOSPHATE_PATHWAY
KEGG_PENTOSE_AND_GLUCURONATE_INTERCONVERSIONS
KEGG_FRUCTOSE_AND_MANNOSE_METABOLISM
KEGG_GALACTOSE_METABOLISM
KEGG_ASCORBATE_AND_ALDARATE_METABOLISM
KEGG_FATTY_ACID_METABOLISM
KEGG_STEROID_BIOSYNTHESIS
KEGG_PRIMARY_BILE_ACID_BIOSYNTHESIS
KEGG_STEROID_HORMONE_BIOSYNTHESIS
KEGG_OXIDATIVE_PHOSPHORYLATION
KEGG_PURINE_METABOLISM
KEGG_PYRIMIDINE_METABOLISM
KEGG_ALANINE_ASPARTATE_AND_GLUTAMATE_METABOLISM
KEGG_GLYCINE_SERINE_AND_THREONINE_METABOLISM
KEGG_CYSTEINE_AND_METHIONINE_METABOLISM
KEGG_VALINE_LEUCINE_AND_ISOLEUCINE_DEGRADATION
KEGG_VALINE_LEUCINE_AND_ISOLEUCINE_BIOSYNTHESIS
KEGG_LYSINE_DEGRADATION
KEGG_ARGININE_AND_PROLINE_METABOLISM
KEGG_HISTIDINE_METABOLISM
KEGG_TYROSINE_METABOLISM
KEGG_PHENYLALANINE_METABOLISM
KEGG_TRYPTOPHAN_METABOLISM
KEGG_BETA_ALANINE_METABOLISM
KEGG_TAURINE_AND_HYPOTAURINE_METABOLISM
KEGG_SELENOAMINO_ACID_METABOLISM
KEGG_GLUTATHIONE_METABOLISM
KEGG_STARCH_AND_SUCROSE_METABOLISM
KEGG_N_GLYCAN_BIOSYNTHESIS
KEGG_OTHER_GLYCAN_DEGRADATION
KEGG_O_GLYCAN_BIOSYNTHESIS
KEGG_AMINO_SUGAR_AND_NUCLEOTIDE_SUGAR_METABOLISM
KEGG_GLYCOSAMINOGLYCAN_DEGRADATION
KEGG_GLYCOSAMINOGLYCAN_BIOSYNTHESIS_CHONDROITIN_SULFATE
KEGG_GLYCOSAMINOGLYCAN_BIOSYNTHESIS_KERATAN_SULFATE
KEGG_GLYCOSAMINOGLYCAN_BIOSYNTHESIS_HEPARAN_SULFATE
KEGG_GLYCEROLIPID_METABOLISM
KEGG_INOSITOL_PHOSPHATE_METABOLISM
KEGG_GLYCOSYLPHOSPHATIDYLINOSITOL_GPI_ANCHOR_BIOSYNTHESIS
KEGG_GLYCEROPHOSPHOLIPID_METABOLISM
KEGG_ETHER_LIPID_METABOLISM
KEGG_ARACHIDONIC_ACID_METABOLISM
KEGG_LINOLEIC_ACID_METABOLISM
KEGG_ALPHA_LINOLENIC_ACID_METABOLISM
KEGG_SPHINGOLIPID_METABOLISM
KEGG_GLYCOSPHINGOLIPID_BIOSYNTHESIS_LACTO_AND_NEOLACTO_SERIES
KEGG_GLYCOSPHINGOLIPID_BIOSYNTHESIS_GLOBO_SERIES
KEGG_GLYCOSPHINGOLIPID_BIOSYNTHESIS_GANGLIO_SERIES
KEGG_PYRUVATE_METABOLISM
KEGG_GLYOXYLATE_AND_DICARBOXYLATE_METABOLISM
KEGG_PROPANOATE_METABOLISM
KEGG_BUTANOATE_METABOLISM
KEGG_ONE_CARBON_POOL_BY_FOLATE
KEGG_RIBOFLAVIN_METABOLISM
KEGG_NICOTINATE_AND_NICOTINAMIDE_METABOLISM
KEGG_PANTOTHENATE_AND_COA_BIOSYNTHESIS
KEGG_FOLATE_BIOSYNTHESIS
KEGG_RETINOL_METABOLISM
KEGG_TERPENOID_BACKBONE_BIOSYNTHESIS
KEGG_LIMONENE_AND_PINENE_DEGRADATION
KEGG_NITROGEN_METABOLISM
KEGG_SULFUR_METABOLISM
KEGG_AMINOACYL_TRNA_BIOSYNTHESIS
KEGG_METABOLISM_OF_XENOBIOTICS_BY_CYTOCHROME_P450
KEGG_DRUG_METABOLISM_CYTOCHROME_P450
KEGG_DRUG_METABOLISM_OTHER_ENZYMES
KEGG_BIOSYNTHESIS_OF_UNSATURATED_FATTY_ACIDS
KEGG_ABC_TRANSPORTERS
KEGG_RIBOSOME
KEGG_RNA_DEGRADATION
KEGG_RNA_POLYMERASE
KEGG_BASAL_TRANSCRIPTION_FACTORS

KEGG_DNA_REPLICATION
KEGG_SPLICEOSOME
KEGG_PROTEASOME
KEGG_PROTEIN_EXPORT
KEGG_PPAR_SIGNALING_PATHWAY
KEGG_BASE_EXCISION_REPAIR
KEGG_NUCLEOTIDE_EXCISION_REPAIR
KEGG_MISMATCH_REPAIR
KEGG_HOMOLOGOUS_RECOMBINATION
KEGG_NON_HOMOLOGOUS_END_JOINING
KEGG_MAPK_SIGNALING_PATHWAY
KEGG_ERBB_SIGNALING_PATHWAY
KEGG_CALCIUM_SIGNALING_PATHWAY
KEGG_CYTOKINE_CYTOKINE_RECEPTOR_INTERACTION
KEGG_CHEMOKINE_SIGNALING_PATHWAY
KEGG_PHOSPHATIDYLINOSITOL_SIGNALING_SYSTEM
KEGG_NEUROACTIVE_LIGAND_RECEPTOR_INTERACTION
KEGG_CELL_CYCLE
KEGG_OOCYTE_MEIOSIS
KEGG_P53_SIGNALING_PATHWAY
KEGG_UBIQUITIN_MEDIATED_PROTEOLYSIS
KEGG_SNARE_INTERACTIONS_IN_VESICULAR_TRANSPORT
KEGG_REGULATION_OF_AUTOPHAGY
KEGG_LYSOSOME
KEGG_ENDOCYTOSIS
KEGG_PEROXISOME
KEGG_MTOR_SIGNALING_PATHWAY
KEGG_APOPTOSIS
KEGG_CARDIAC_MUSCLE_CONTRACTION
KEGG_VASCULAR_SMOOTH_MUSCLE_CONTRACTION
KEGG_WNT_SIGNALING_PATHWAY
KEGG_DORSO_VENTRAL_AXIS_FORMATION
KEGG_NOTCH_SIGNALING_PATHWAY
KEGG_HEDGEHOG_SIGNALING_PATHWAY
KEGG_TGF_BETA_SIGNALING_PATHWAY
KEGG_AXON_GUIDANCE
KEGG_VEGF_SIGNALING_PATHWAY
KEGG_FOCAL_ADHESION
KEGG_ECM_RECEPTOR_INTERACTION
KEGG_CELL_ADHESION_MOLECULES_CAMS
KEGG_ADHERENS_JUNCTION
KEGG_TIGHT_JUNCTION
KEGG_GAP_JUNCTION
KEGG_COMPLEMENT_AND_COAGULATION_CASCADES
KEGG_ANTIGEN_PROCESSING_AND_PRESENTATION
KEGG_RENIN_ANGIOTENSIN_SYSTEM
KEGG_TOLL_LIKE_RECEPTOR_SIGNALING_PATHWAY
KEGG_NOD_LIKE_RECEPTOR_SIGNALING_PATHWAY
KEGG_RIG_I_LIKE_RECEPTOR_SIGNALING_PATHWAY
KEGG_CYTOSOLIC_DNA_SENSING_PATHWAY
KEGG_JAK_STAT_SIGNALING_PATHWAY
KEGG_HEMATOPOIETIC_CELL_LINEAGE
KEGG_NATURAL_KILLER_CELL_MEDIATED_CYTOTOXICITY
KEGG_T_CELL_RECEPTOR_SIGNALING_PATHWAY
KEGG_B_CELL_RECEPTOR_SIGNALING_PATHWAY
KEGG_FC_EPSILON_RI_SIGNALING_PATHWAY
KEGG_FC_GAMMA_R_MEDIATED_PHAGOCYTOSIS
KEGG_LEUKOCYTE_TRANSENDOTHELIAL_MIGRATION
KEGG_INSULIN_SIGNALING_PATHWAY
KEGG_GNRH_SIGNALING_PATHWAY
KEGG_NEUROTROPHIN_SIGNALING_PATHWAY
REACTOME_GLYCOGEN_BREAKDOWN_GLYCOGENOLYSIS
REACTOME_TRANSLATION
REACTOME_PYRIMIDINE_CATABOLISM
REACTOME_RNA_POL_III_TRANSCRIPTION_INITIATION_FROM_TYPE_2_PROMOTER
REACTOME_PYRUVATE_METABOLISM_AND_CITRIC_ACID_TCA_CYCLE
REACTOME_PTM_GAMMA_CARBOXYLATION_HYPUSINE_FORMATION_AND_ARYLSULFATASE_ACTIVATION
REACTOME_RNA_POL_I_TRANSCRIPTION_TERMINATION
REACTOME_ACTIVATION_OF_THE_PRE_REPLICATIVE_COMPLEX
REACTOME_PROCESSING_OF_INTRONLESS_PRE_MRNAS
REACTOME_GAP_JUNCTION_DEGRADATION
REACTOME_BILE_ACID_AND_BILE_SALT_METABOLISM
REACTOME_SYNTHESIS_OF_BILE_ACIDS_AND_BILE_SALTS_VIA_7ALPHA_HYDROXYCHOLESTEROL
REACTOME_RECYCLING_OF_BILE_ACIDS_AND_SALTS
REACTOME_METABOLISM_OF_NON_CODING_RNA
REACTOME_SYNTHESIS_OF_BILE_ACIDS_AND_BILE_SALTS_VIA_24_HYDROXYCHOLESTEROL
REACTOME_SYNTHESIS_OF_BILE_ACIDS_AND_BILE_SALTS

REACTOME_METABOLISM_OF_STEROID_HORMONES_AND_VITAMINS_A_AND_D
REACTOME_ANDROGEN_BIOSYNTHESIS
REACTOME_COPI_MEDIATED_TRANSPORT
REACTOME_TCA_CYCLE_AND_RESPIRATORY_ELECTRON_TRANSPORT
REACTOME_GROWTH_HORMONE_RECEPTOR_SIGNALING
REACTOME_CELL_CELL_COMMUNICATION
REACTOME_ABCA_TRANSPORTERS_IN_LIPID_HOMEOSTASIS
REACTOME_ENDOSOMAL_VACUOLAR_PATHWAY
REACTOME_TETRAHYDROBIOPTERIN_BH4_SYNTHESIS_RECYCLING_SALVAGE_AND_REGULATION
REACTOME_ACTIVATED_AMPK_STIMULATES_FATTY_ACID_OXIDATION_IN_MUSCLE
REACTOME_VITAMIN_B5_PANTOTHENATE_METABOLISM
REACTOME_METABOLISM_OF_VITAMINS_AND_COFACTORS
REACTOME_O_LINKED_GLYCOSYLATION_OF_MUCINS
REACTOME_SULFUR_AMINO_ACID_METABOLISM
REACTOME_SPHINGOLIPID_DE_NOVO_BIOSYNTHESIS
REACTOME_TERMINATION_OF_O_GLYCAN_BIOSYNTHESIS
REACTOME_GLYCOSPHINGOLIPID_METABOLISM
REACTOME_PPARA_ACTIVATES_GENE_EXPRESSION
REACTOME_TRIGLYCERIDE_BIOSYNTHESIS
REACTOME_ACYL_CHAIN_REMODELLING_OF_PI
REACTOME_TGF_BETA_RECEPTOR_SIGNALING_IN_EMT_EPITHELIAL_TO_MESENCHYMAL_TRANSITION
REACTOME_DOWNREGULATION_OF_TGF_BETA_RECEPTOR_SIGNALING
REACTOME_ACYL_CHAIN_REMODELLING_OF_PC
REACTOME_TGF_BETA_RECEPTOR_SIGNALING_ACTIVATES_SMADS
REACTOME_PHOSPHOLIPID_METABOLISM
REACTOME_CS_DS_DEGRADATION
REACTOME_SYNTHESIS_OF_PA
REACTOME_OXYGEN_DEPENDENT_PROLINE_HYDROXYLATION_OF_HYPOXIA_INDUCIBLE_FACTOR_ALPHA
REACTOME_SYNTHESIS_OF_PE
REACTOME_CHONDROITIN_SULFATE_BIOSYNTHESIS
REACTOME_HYALURONAN_UPTAKE_AND_DEGRADATION
REACTOME_HYALURONAN_METABOLISM
REACTOME_KERATAN_SULFATE_BIOSYNTHESIS
REACTOME_ALPHA_LINOLENIC_ACID_ALA_METABOLISM
REACTOME_PI_METABOLISM
REACTOME_CHONDROITIN_SULFATE_DERMATAN_SULFATE_METABOLISM
REACTOME_SYNTHESIS_OF_PC
REACTOME_HS_GAG_BIOSYNTHESIS
REACTOME_KERATAN_SULFATE_KERATIN_METABOLISM
REACTOME_KERATAN_SULFATE_DEGRADATION
REACTOME_HEPARAN_SULFATE_HEPARIN_HS_GAG_METABOLISM
REACTOME_GLYCOSAMINOGLYCAN_METABOLISM
REACTOME_ACYL_CHAIN_REMODELLING_OF_PG
REACTOME_ACYL_CHAIN_REMODELLING_OF_PE
REACTOME_ACYL_CHAIN_REMODELLING_OF_PS
REACTOME_GLYCEROPHOSPHOLIPID_BIOSYNTHESIS
REACTOME_PLATELET_ADHESION_TO_EXPOSED_COLLAGEN
REACTOME_REGULATION_OF_PYRUVATE_DEHYDROGENASE_PDH_COMPLEX
REACTOME_METABOLISM_OF_AMINO_ACIDS_AND_DERIVATIVES
REACTOME_RNA_POL_I_TRANSCRIPTION
REACTOME_FATTY_ACYL_COA_BIOSYNTHESIS
REACTOME_INTEGRIN_CELL_SURFACE_INTERACTIONS
REACTOME_REGULATION_OF_ORNITHINE_DECARBOXYLASE_ODC
REACTOME_CYTOCHROME_P450_ARRANGED_BY_SUBSTRATE_TYPE
REACTOME_BASE_FREE_SUGAR_PHOSPHATE_REMOVAL_VIA_THE_SINGLE_NUCLEOTIDE_REPLACEMENT_PATHWAY
REACTOME_HDL_MEDIATED_LIPID_TRANSPORT
REACTOME_RNA_POL_II_TRANSCRIPTION
REACTOME_RNA_POL_III_TRANSCRIPTION
REACTOME_AMINO_ACID_TRANSPORT_ACROSS_THE_PLASMA_MEMBRANE
REACTOME_ENDOGENOUS_STEROLS
REACTOME_GLYCOLYSIS
REACTOME_MITOCHONDRIAL_FATTY_ACID_BETA_OXIDATION
REACTOME_METABOLISM_OF_POLYAMINES
REACTOME_INTEGRATION_OF_ENERGY_METABOLISM
REACTOME_GLUCONEOGENESIS
REACTOME_MITOCHONDRIAL_TRNA_AMINOACYLATION
REACTOME_CYTOSOLIC_TRNA_AMINOACYLATION
REACTOME_ADENYLATE_CYCLASE_ACTIVATING_PATHWAY
REACTOME_ADENYLATE_CYCLASE_INHIBITORY_PATHWAY
REACTOME_PEPTIDE_HORMONE_BIOSYNTHESIS
REACTOME_ACETYLCHOLINE_BINDING_AND_DOWNSTREAM_EVENTS
REACTOME_ABC_FAMILY_PROTEINS_MEDIATED_TRANSPORT
REACTOME_TRNA_AMINOACYLATION
REACTOME_STEROID_HORMONES
REACTOME_AMINE_DERIVED_HORMONES
REACTOME_INSULIN_SYNTHESIS_AND_PROCESSING
REACTOME_GLUCAGON_SIGNALING_IN_METABOLIC_REGULATION

REACTOME_MRNA_PROCESSING
REACTOME_PEROXISOMAL_LIPID_METABOLISM
REACTOME_METABOLISM_OF_NUCLEOTIDES
REACTOME_AMINE_LIGAND_BINDING_RECEPTORS
REACTOME_METABOLISM_OF_PROTEINS
REACTOME_SEROTONIN_RECEPTORS
REACTOME_MRNA_SPLICING
REACTOME_MRNA_SPLICING_MINOR_PATHWAY
REACTOME_3_UTR_MEDIATED_TRANSLATIONAL_REGULATION
REACTOME_PURINE_RIBONUCLEOSIDE_MONOPHOSPHATE_BIOSYNTHESIS
REACTOME_CITRIC_ACID_CYCLE_TCA_CYCLE
REACTOME_REGULATION_OF_INSULIN_SECRETION_BY_GLUCAGON_LIKE_PEPTIDE1
REACTOME_REGULATION_OF_INSULIN_SECRETION
REACTOME_INHIBITION_OF_INSULIN_SECRETION_BY_ADRENALINE_NORADRENALINE
REACTOME_NUCLEOTIDE_LIKE_PURINERGIC_RECEPTORS
REACTOME_ACTIVATION_OF_CHAPERONES_BY_ATF6_ALPHA
REACTOME_EICOSANOID_LIGAND_BINDING_RECEPTORS
REACTOME_UNFOLDED_PROTEIN_RESPONSE
REACTOME_GLUCAGON_TYPE_LIGAND_RECEPTORS
REACTOME_REGULATION_OF_INSULIN_SECRETION_BY_ACETYLCHOLINE
REACTOME_SYNTHESIS_SECRETION_AND_DEACYLATION_OF_GHRELIN
REACTOME_PURINE_SALVAGE
REACTOME_LYSOSOME_VESICLE_BIOGENESIS
REACTOME_TRANSPORT_OF_GLUCOSE_AND_OTHER_SUGARS_BILE_SALTS_AND_ORGANIC_ACIDS_METAL_IONS_AND_AMINE_COMPOUNDS
REACTOME_SPHINGOLIPID_METABOLISM
REACTOME_CELL_CELL_JUNCTION_ORGANIZATION
REACTOME_TRANSPORT_OF_INORGANIC_CATIONS_ANIONS_AND_AMINO_ACIDS_OLIGOPEPTIDES
REACTOME_GOLGI_ASSOCIATED_VESICLE_BIOGENESIS
REACTOME_AMINO_ACID_AND_OLIGOPEPTIDE_SLC_TRANSPORTERS
REACTOME_BRANCHED_CHAIN_AMINO_ACID_CATABOLISM
REACTOME_SYNTHESIS_OF_DNA
REACTOME_DEADENYLATION_OF_MRNA
REACTOME_MRNA_DECAY_BY_5_TO_3_EXORIBONUCLEASE
REACTOME_ZINC_TRANSPORTERS
REACTOME_METAL_ION_SLC_TRANSPORTERS
REACTOME_AUTODEGRADATION_OF_THE_E3_UBIQUITIN_LIGASE_COP1
REACTOME_METABOLISM_OF_MRNA
REACTOME_MRNA_DECAY_BY_3_TO_5_EXORIBONUCLEASE
REACTOME_BILE_SALT_AND_ORGANIC_ANION_SLC_TRANSPORTERS
REACTOME_DEADENYLATION_DEPENDENT_MRNA_DECAY
REACTOME_AMINE_COMPOUND_SLC_TRANSPORTERS
REACTOME_PYRUVATE_METABOLISM
REACTOME_PURINE_CATABOLISM
REACTOME_GLUCOSE_TRANSPORT
REACTOME_METABOLISM_OF_RNA
REACTOME_MITOTIC_G1_G1_S_PHASES
REACTOME_MYOGENESIS
REACTOME_PHOSPHOLIPASE_C_MEDIATED_CASCADE
REACTOME_ACTIVATION_OF_KAINATE_RECEPTORS_UPON_GLUTAMATE_BINDING
REACTOME_SYNTHESIS_AND_INTERCONVERSION_OF_NUCLEOTIDE_DI_AND_TRIPHOSPHATES
REACTOME_RNA_POL_I_RNA_POL_III_AND_MITOCHONDRIAL_TRANSCRIPTION
REACTOME_DNA_REPAIR
REACTOME_EFFECTS_OF_PIP2_HYDROLYSIS
REACTOME_METABOLISM_OF_LIPIDS_AND_LIPOPROTEINS
REACTOME_FATTY_ACID_TRIACYLGLYCEROL_AND_KETONE_BODY_METABOLISM
REACTOME_TRANSPORT_OF_VITAMINS_NUCLEOSIDES_AND_RELATED_MOLECULES
REACTOME_HIGHLY_CALCIUM_PERMEABLE_POSTSYNAPTIC_NICOTINIC_ACETYLCHOLINE_RECEPTORS
REACTOME_ORGANIC_CATION_ANION_ZWITTERION_TRANSPORT
REACTOME_SYNTHESIS_OF_SUBSTRATES_IN_N_GLYCAN_BIOSYTHESIS
REACTOME_RESPIRATORY_ELECTRON_TRANSPORT
REACTOME_ASPARAGINE_N_LINKED_GLYCOSYLATION
REACTOME_BIOSYNTHESIS_OF_THE_N_GLYCAN_PRECURSOR_DOLICHOL_LIPID_LINKED_OLIGOSACCHARIDE_LLO_AND_TRANSFER_TO_A_NASCENT_PROTEIN
REACTOME_AMINO_ACID_SYNTHESIS_AND_INTERCONVERSION_TRANSAMINATION
REACTOME_NITRIC_OXIDE_STIMULATES_GUANYLATE_CYCLASE
REACTOME_N_GLYCAN_TRIMMING_IN_THE_ER_AND_CALNEXIN_CALRETICULIN_CYCLE
REACTOME_PLATELET_SENSITIZATION_BY_LDL
REACTOME_AQUAPORIN_MEDIATED_TRANSPORT
REACTOME_PLATELET_CALCIUM_HOMEOSTASIS
REACTOME_INCRETIN_SYNTHESIS_SECRETION_AND_INACTIVATION
REACTOME_TRANSPORT_OF_ORGANIC_ANIONS
REACTOME_REGULATION_OF_WATER_BALANCE_BY_RENAL_AQUAPORINS
REACTOME_TRANSPORT_TO_THE_GOLGI_AND_SUBSEQUENT_MODIFICATION
REACTOME_IRON_UPTAKE_AND_TRANSPORT
REACTOME_N_GLYCAN_ANTENNAE_ELONGATION
REACTOME_ION_TRANSPORT_BY_P_TYPE_ATPASES
REACTOME_ADVANCED_GLYCOSYLATION_ENDPRODUCT_RECEPTOR_SIGNALING
REACTOME_N_GLYCAN_ANTENNAE_ELONGATION_IN_THE_MEDIAL_TRANS_GOLGI

REACTOME_ION_CHANNEL_TRANSPORT
REACTOME_ETHANOL_OXIDATION
REACTOME_SYNTHESIS_OF_VERY_LONG_CHAIN_FATTY_ACYL_COAS
REACTOME_DNA_REPLICATION
REACTOME_E2F_MEDIATED_REGULATION_OF_DNA_REPLICATION
REACTOME_METABOLISM_OF_CARBOHYDRATES
REACTOME_HORMONE_SENSITIVE_LIPASE_HSL_MEDIATED_TRIACYLGLYCEROL_HYDROLYSIS
REACTOME_PURINE_METABOLISM
REACTOME_LIPID_DIGESTION_MOBILIZATION_AND_TRANSPORT
REACTOME_TRANSPORT_OF_RIBONUCLEOPROTEINS_INTO_THE_HOST_NUCLEUS
REACTOME_RESPIRATORY_ELECTRON_TRANSPORT_ATP_SYNTHESIS_BY_CHEMIOSMOTIC_COUPLING_AND_HEAT_PRODUCTION_BY_UNCOUPLING_PROTEINS
REACTOME_FORMATION_OF_ATP_BY_CHEMIOSMOTIC_COUPLING
REACTOME_GLUCURONIDATION
REACTOME_REGULATION_OF_GLUCOKINASE_BY_GLUCOKINASE_REGULATORY_PROTEIN
REACTOME_LIPOPROTEIN_METABOLISM
REACTOME_CHYLOMICRON_MEDIATED_LIPID_TRANSPORT
REACTOME_SIGNALING_BY_TGF_BETA_RECEPTOR_COMPLEX
REACTOME_GLUTATHIONE_CONJUGATION
REACTOME_GLUCOSE_METABOLISM
REACTOME_VOLTAGE_GATED_POTASSIUM_CHANNELS
REACTOME_POTASSIUM_CHANNELS
REACTOME_NUCLEOTIDE_BINDING_DOMAIN_LEUCINE_RICH_REPEAT_CONTAINING_RECEPTOR_NLR_SIGNALING_PATHWAYS
REACTOME_AMYLOIDS
REACTOME_TELOMERE_MAINTENANCE
REACTOME_TRYPTOPHAN_CATABOLISM
REACTOME_CHOLESTEROL_BIOSYNTHESIS
REACTOME_METABOLISM_OF_PORPHYRINS
REACTOME_DIGESTION_OF_DIETARY_CARBOHYDRATE
REACTOME_SYNTHESIS_OF_GLYCOSYLPHOSPHATIDYLINOSITOL_GPI
REACTOME_PYRIMIDINE_METABOLISM

Appendix Table S4. List of meta-correlation values with meta-pvalues of KEGG gene sets associated with pan-cancer EMT gene signature

ID	Meta_Correlation	Meta_pval
KEGG_ECM_RECEPTOR_INTERACTION	0.82	1E-104
KEGG_FOCAL_ADHESION	0.75	2E-105
KEGG_GLYCOSAMINOGLYCAN_BIOSYNTHESIS_CHONDROITIN_SULFATE	0.71	2E-86
KEGG_CYTOKINE_CYTOKINE_RECEPTOR_INTERACTION	0.57	5E-96
KEGG_CHEMOKINE_SIGNALING_PATHWAY	0.54	1E-88
KEGG_FC_GAMMA_R_MEDIATED_PHAGOCYTOSIS	0.54	7E-116
KEGG_TGF_BETA_SIGNALING_PATHWAY	0.49	9E-18
KEGG_GAP_JUNCTION	0.49	2E-81
KEGG_TOLL_LIKE_RECEPTOR_SIGNALING_PATHWAY	0.48	2E-87
KEGG_LEUKOCYTE_TRANSENDOTHELIAL_MIGRATION	0.48	3E-90
KEGG_HEMATOPOIETIC_CELL_LINEAGE	0.47	2E-32
KEGG_NOD_LIKE_RECEPTOR_SIGNALING_PATHWAY	0.47	9E-83
KEGG_NATURAL_KILLER_CELL_MEDIATED_CYTOTOXICITY	0.44	2E-46
KEGG_B_CELL_RECEPTOR_SIGNALING_PATHWAY	0.44	1E-72
KEGG_JAK_STAT_SIGNALING_PATHWAY	0.44	4E-35
KEGG_CELL_ADHESION_MOLECULES_CAMS	0.43	1E-43
KEGG_NEUROACTIVE_LIGAND_RECEPTOR_INTERACTION	0.40	9E-33
KEGG_AXON_GUIDANCE	0.39	1E-41
KEGG_CALCIUM_SIGNALING_PATHWAY	0.39	2E-11
KEGG_T_CELL_RECEPTOR_SIGNALING_PATHWAY	0.37	7E-51
KEGG_CYTOSOLIC_DNA_SENSING_PATHWAY	0.33	1E-20
KEGG_GLYCOSAMINOGLYCAN_BIOSYNTHESIS_HEPARAN_SULFATE	0.32	2E-37
KEGG_GLYCOSPHINGOLIPID_BIOSYNTHESIS_GLOBO_SERIES	0.32	8E-37
KEGG_MAPK_SIGNALING_PATHWAY	0.30	7E-27
KEGG_ANTIGEN_PROCESSING_AND_PRESENTATION	0.30	1E-13
KEGG_COMPLEMENT_AND_COAGULATION_CASCADES	0.29	3E-15
KEGG_NICOTINATE_AND_NICOTINAMIDE_METABOLISM	0.29	4E-30
KEGG_GLYCOSPHINGOLIPID_BIOSYNTHESIS_GANGLIO_SERIES	0.28	1E-07
KEGG_VASCULAR_SMOOTH_MUSCLE_CONTRACTION	0.28	4E-05
KEGG_APOPTOSIS	0.27	1E-21
KEGG_GLYCOSAMINOGLYCAN_BIOSYNTHESIS_KERATAN_SULFATE	0.26	6E-06
KEGG_P53_SIGNALING_PATHWAY	0.26	2E-07
KEGG_HEDGEHOG_SIGNALING_PATHWAY	0.25	4E-07
KEGG_RENIN_ANGIOTENSIN_SYSTEM	0.25	4E-05
KEGG_GLYCOSAMINOGLYCAN_DEGRADATION	0.22	4E-05
KEGG_WNT_SIGNALING_PATHWAY	0.21	3E-14
KEGG_NEUROTROPHIN_SIGNALING_PATHWAY	0.20	3E-09
KEGG_FC_EPSILON_RI_SIGNALING_PATHWAY	0.20	1E-07
KEGG_VEGF_SIGNALING_PATHWAY	0.18	9E-10
KEGG_RIG_I_LIKE_RECEPTOR_SIGNALING_PATHWAY	0.16	2E-10
KEGG_MTOR_SIGNALING_PATHWAY	0.15	1E-03
KEGG_DORSO_VENTRAL_AXIS_FORMATION	0.15	3E-04
KEGG_PANTOTHENATE_AND_COA_BIOSYNTHESIS	0.15	6E-04
KEGG_LYSOSOME	0.14	1E-04
KEGG_ADHERENS_JUNCTION	0.10	1E-04
KEGG_GALACTOSE_METABOLISM	0.10	4E-03
KEGG_ENDOCYTOSIS	0.08	4E-02
KEGG_CELL_CYCLE	0.06	2E-01
KEGG_OOCYTE_MEIOSIS	0.04	2E-01
KEGG_OTHER_GLYCAN_DEGRADATION	0.04	2E-01
KEGG_AMINO_SUGAR_AND_NUCLEOTIDE_SUGAR_METABOLISM	0.03	2E-01
KEGG_NOTCH_SIGNALING_PATHWAY	0.03	3E-01
KEGG_DRUG_METABOLISM_OTHER_ENZYMES	0.03	3E-01
KEGG_PPAR_SIGNALING_PATHWAY	0.01	4E-01
KEGG_N_GLYCAN_BIOSYNTHESIS	0.01	4E-01
KEGG_RIBOFLAVIN_METABOLISM	0.01	4E-01
KEGG_PROTEASOME	0.01	4E-01
KEGG_PHOSPHATIDYLINOSITOL_SIGNALING_SYSTEM	0.00	5E-01

KEGG_PENTOSE_AND_GLUCURONATE_INTERCONVERSIONS	-0.02	3E-01
KEGG_ERBB_SIGNALING_PATHWAY	-0.02	2E-01
KEGG_PROTEIN_EXPORT	-0.03	2E-01
KEGG_ASCORBATE_AND_ALDARATE_METABOLISM	-0.03	2E-01
KEGG_DNA_REPLICATION	-0.04	3E-01
KEGG_SNARE_INTERACTIONS_IN_VESICULAR_TRANSPORT	-0.04	2E-01
KEGG_STARCH_AND_SUCROSE_METABOLISM	-0.04	5E-02
KEGG_TRYPTOPHAN_METABOLISM	-0.05	9E-02
KEGG_STEROID_HORMONE_BIOSYNTHESIS	-0.05	7E-02
KEGG_PRIMARY_BILE_ACID_BIOSYNTHESIS	-0.05	5E-02
KEGG_REGULATION_OF_AUTOPHAGY	-0.05	2E-01
KEGG_GNRH_SIGNALING_PATHWAY	-0.06	7E-02
KEGG_SULFUR_METABOLISM	-0.06	1E-01
KEGG_ETHER_LIPID_METABOLISM	-0.07	2E-01
KEGG_FRUCTOSE_AND_MANNOSE_METABOLISM	-0.07	3E-02
KEGG_BASAL_TRANSCRIPTION_FACTORS	-0.07	1E-01
KEGG_SPHINGOLIPID_METABOLISM	-0.07	2E-02
KEGG_HOMOLOGOUS_RECOMBINATION	-0.08	1E-01
KEGG_UBIQUITIN_MEDIATED_PROTEOLYSIS	-0.08	1E-02
KEGG_PURINE_METABOLISM	-0.08	5E-02
KEGG_RIBOSOME	-0.09	2E-02
KEGG_INSULIN_SIGNALING_PATHWAY	-0.09	3E-02
KEGG_RETINOL_METABOLISM	-0.09	4E-04
KEGG_MISMATCH_REPAIR	-0.10	1E-02
KEGG_DRUG_METABOLISM_CYTOCHROME_P450	-0.10	4E-04
KEGG_PYRIMIDINE_METABOLISM	-0.11	8E-03
KEGG_GLYCEROLIPID_METABOLISM	-0.12	9E-04
KEGG_METABOLISM_OF_XENOBIOTICS_BY_CYTOCHROME_P450	-0.12	1E-04
KEGG_O_GLYCAN_BIOSYNTHESIS	-0.13	6E-07
KEGG_PENTOSE_PHOSPHATE_PATHWAY	-0.13	2E-03
KEGG_ARACHIDONIC_ACID_METABOLISM	-0.13	3E-02
KEGG_ONE_CARBON_POOL_BY_FOLATE	-0.14	3E-02
KEGG_BIOSYNTHESIS_OF_UNSATURATED_FATTY_ACIDS	-0.14	2E-08
KEGG_PHENYLALANINE_METABOLISM	-0.14	5E-08
KEGG_FOLATE_BIOSYNTHESIS	-0.15	6E-05
KEGG_GLYCOSPHINGOLIPID_BIOSYNTHESIS_LACTO_AND_NEOLACTO_SERIES	-0.15	4E-06
KEGG_GLYCOLYSIS_GLUCONEOGENESIS	-0.15	8E-04
KEGG_VALINE_LEUCINE_AND_ISOLEUCINE_BIOSYNTHESIS	-0.15	1E-05
KEGG_CARDIAC_MUSCLE_CONTRACTION	-0.16	5E-03
KEGG_INOSITOL_PHOSPHATE_METABOLISM	-0.16	9E-03
KEGG_GLYOXYLATE_AND_DICARBOXYLATE_METABOLISM	-0.16	5E-05
KEGG_TAURINE_AND_HYPOTAURINE_METABOLISM	-0.16	1E-03
KEGG_NUCLEOTIDE_EXCISION_REPAIR	-0.17	5E-09
KEGG_GLYCINE_SERINE_AND_THREONINE_METABOLISM	-0.18	2E-06
KEGG_NITROGEN_METABOLISM	-0.18	8E-03
KEGG_ABC_TRANSPORTERS	-0.19	2E-04
KEGG_BETA_ALANINE_METABOLISM	-0.19	4E-04
KEGG_BASE_EXCISION_REPAIR	-0.20	3E-04
KEGG_ALANINE_ASPARTATE_AND_GLUTAMATE_METABOLISM	-0.20	2E-05
KEGG_CYSTEINE_AND_METHIONINE_METABOLISM	-0.21	5E-05
KEGG_NON_HOMOLOGOUS_END_JOINING	-0.21	8E-06
KEGG_RNA_DEGRADATION	-0.22	2E-08
KEGG_GLUTATHIONE_METABOLISM	-0.23	9E-07
KEGG_AMINOACYL_TRNA_BIOSYNTHESIS	-0.23	8E-14
KEGG_ALPHA_LINOLENIC_ACID_METABOLISM	-0.23	1E-05
KEGG_TIGHT_JUNCTION	-0.23	2E-07
KEGG_LINOLEIC_ACID_METABOLISM	-0.26	7E-16
KEGG_ARGININE_AND_PROLINE_METABOLISM	-0.26	9E-10
KEGG_SPLICEOSOME	-0.27	8E-09
KEGG_LYSINE_DEGRADATION	-0.28	1E-17
KEGG_TYROSINE_METABOLISM	-0.28	1E-18
KEGG_OXIDATIVE_PHOSPHORYLATION	-0.29	8E-21

KEGG_RNA_POLYMERASE	-0.29	2E-31
KEGG_CITRATE_CYCLE_TCA_CYCLE	-0.31	5E-19
KEGG_STEROID_BIOSYNTHESIS	-0.33	2E-17
KEGG_HISTIDINE_METABOLISM	-0.35	4E-17
KEGG_GLYCEROPHOSPHOLIPID_METABOLISM	-0.37	2E-17
KEGG_LIMONENE_AND_PINENE_DEGRADATION	-0.37	1E-07
KEGG_PYRUVATE_METABOLISM	-0.39	4E-18
KEGG_SELENOAMINO_ACID_METABOLISM	-0.43	1E-12
KEGG_TERPENOID_BACKBONE_BIOSYNTHESIS	-0.44	1E-57
KEGG_BUTANOATE_METABOLISM	-0.46	3E-22
KEGG_FATTY_ACID_METABOLISM	-0.46	2E-21
KEGG_PROPANOATE_METABOLISM	-0.46	1E-16
KEGG_VALINE_LEUCINE_AND_ISOLEUCINE_DEGRADATION	-0.47	2E-14
KEGG_GLYCOSYLPHOSPHATIDYLINOSITOL_GPI_ANCHOR_BIOSYNTHESIS	-0.47	3E-29
KEGG_PEROXISOME	-0.52	2E-14

Appendix Table S5. List of meta-correlation values with meta-pvalues of REACTOME gene sets associated with pan-cancer EMT gene signature

ID	Meta_correlation	Meta_pval
REACTOME_CHONDROITIN_SULFATE_BIOSYNTHESIS	0.83	1E-94
REACTOME_PLATELET_ADHESION_TO_EXPOSED_COLLAGEN	0.76	5E-91
REACTOME_CHONDROITIN_SULFATE_DERMATAN_SULFATE_METABOLISM	0.70	6E-94
REACTOME_GLYCOSAMINOGLYCAN_METABOLISM	0.70	8E-96
REACTOME_INTEGRIN_CELL_SURFACE_INTERACTIONS	0.67	1E-79
REACTOME_CS_DS_DEGRADATION	0.64	5E-38
REACTOME_KERATAN_SULFATE_BIOSYNTHESIS	0.64	3E-56
REACTOME_KERATAN_SULFATE_DEGRADATION	0.64	8E-166
REACTOME_KERATAN_SULFATE_KERATIN_METABOLISM	0.63	1E-39
REACTOME_MYOGENESIS	0.53	5E-22
REACTOME_HEPARAN_SULFATE_HEPARIN_HS_GAG_METABOLISM	0.51	4E-79
REACTOME_EICOSANOID_LIGAND_BINDING_RECEPTORS	0.49	4E-29
REACTOME_NITRIC_OXIDE_STIMULATES_GUANYLATE_CYCLASE	0.46	2E-09
REACTOME_GAP_JUNCTION_DEGRADATION	0.44	2E-18
REACTOME_METABOLISM_OF_CARBOHYDRATES	0.40	4E-18
REACTOME_INHIBITION_OF_INSULIN_SECRETION_BY_ADRENALINE_NORADRENALINE	0.38	3E-08
REACTOME_HDL_MEDIATED_LIPID_TRANSPORT	0.37	1E-44
REACTOME_NUCLEOTIDE_BINDING_DOMAIN_LEUCINE_RICH_REPEAT_CONTAINING_RECEPTOR_NLR_SIGNALING_PATHWAYS	0.37	7E-49
REACTOME_NUCLEOTIDE_LIKE_PURINERGIC_RECEPTORS	0.35	1E-43
REACTOME_TRANSPORT_TO_THE_GOLGI_AND_SUBSEQUENT_MODIFICATION	0.35	2E-26
REACTOME_GROWTH_HORMONE_RECEPTOR_SIGNALING	0.34	1E-26
REACTOME_HS_GAG_BIOSYNTHESIS	0.33	4E-40
REACTOME_GLUCAGON_SIGNALING_IN_METABOLIC_REGULATION	0.33	1E-04
REACTOME_ACTIVATION_OF_KAINATE_RECEPTORS_UPON_GLUTAMATE_BINDING	0.28	4E-05
REACTOME_HYALURONAN_METABOLISM	0.27	1E-18
REACTOME_SEROTONIN_RECEPTORS	0.27	5E-11
REACTOME_PEPTIDE_HORMONE_BIOSYNTHESIS	0.26	3E-10
REACTOME_ENDOGENOUS_STEROLS	0.26	3E-17
REACTOME_POTASSIUM_CHANNELS	0.25	1E-03
REACTOME_ADENYLATE_CYCLASE_INHIBITORY_PATHWAY	0.25	2E-05
REACTOME_LIPOPROTEIN_METABOLISM	0.24	1E-16
REACTOME_CELL_CELL_COMMUNICATION	0.24	2E-09
REACTOME_ENDOSOMAL_VACUOLAR_PATHWAY	0.24	4E-09
REACTOME_TRYPTOPHAN_CATABOLISM	0.23	6E-20
REACTOME_UNFOLDED_PROTEIN_RESPONSE	0.23	2E-15
REACTOME_N_GLYCAN_ANTENNAE_ELONGATION_IN_THE_MEDIAL_TRANS_GOLGI	0.23	3E-07
REACTOME_LIPID_DIGESTION_MOBILIZATION_AND_TRANSPORT	0.23	7E-06
REACTOME_SYNTHESIS_SECRETION_AND_DEACYLATION_OF_GHRELIN	0.23	5E-08
REACTOME_REGULATION_OF_INSULIN_SECRETION_BY_GLUCAGON_LIKE_PEPTIDE1	0.22	5E-04
REACTOME_REGULATION_OF_INSULIN_SECRETION	0.22	2E-03
REACTOME_AMINO_ACID_TRANSPORT_ACROSS_THE_PLASMA_MEMBRANE	0.21	2E-07
REACTOME_GLUCAGON_TYPE_LIGAND_RECEPTORS	0.20	2E-03
REACTOME_AMINO_ACID_AND_OLIGOPEPTIDE_SLC_TRANSPORTERS	0.19	4E-04
REACTOME_HYALURONAN_UPTAKE_AND_DEGRADATION	0.19	9E-14
REACTOME_PYRIMIDINE_METABOLISM	0.19	7E-05
REACTOME_DOWNREGULATION_OF_TGF_BETA_RECEPTOR_SIGNALING	0.18	3E-10
REACTOME_ADVANCED_GLYCOSYLATION_ENDPRODUCT_RECEPTOR_SIGNALING	0.17	7E-04
REACTOME_INCRETIN_SYNTHESIS_SECRETION_AND_INACTIVATION	0.17	1E-03
REACTOME_BILE_SALT_AND_ORGANIC_ANION_SLC_TRANSPORTERS	0.16	7E-03
REACTOME_TGF_BETA_RECEPTOR_SIGNALING_ACTIVATES_SMADS	0.16	1E-08
REACTOME_PURINE_SALVAGE	0.16	5E-10
REACTOME_N_GLYCAN_ANTENNAE_ELONGATION	0.14	2E-02
REACTOME_PYRIMIDINE_CATABOLISM	0.13	5E-04
REACTOME_STEROID_HORMONES	0.13	2E-04
REACTOME_ADENYLATE_CYCLASE_ACTIVATING_PATHWAY	0.12	2E-02
REACTOME_INTEGRATION_OF_ENERGY_METABOLISM	0.12	5E-02
REACTOME_HORMONE_SENSITIVE_LIPASE_HSL_MEDIATED_TRIACYLGLYCEROL_HYDROLYSIS	0.11	5E-02

REACTOME_AMINE_LIGAND_BINDING_RECEPTORS	0.11	2E-04
REACTOME_TRANSPORT_OF_ORGANIC_ANIONS	0.11	8E-03
REACTOME_ASPARAGINE_N_LINKED_GLYCOSYLATION	0.11	6E-05
REACTOME_OXYGEN_DEPENDENT_PROLINE_HYDROXYLATION_OF_HYPOXIA_INDUCIBLE_FACTOR_ALPHA	0.10	3E-03
REACTOME_REGULATION_OF_WATER_BALANCE_BY_RENAL_AQUAPORINS	0.10	6E-02
REACTOME_ACETYLCHOLINE_BINDING_AND_DOWNSTREAM_EVENTS	0.10	8E-02
REACTOME_HIGHLY_CALCIUM_PERMEABLE_POSTSYNAPTIC_NICOTINIC_ACETYLCHOLINE_RECEPTORS	0.10	8E-02
REACTOME_PLATELET_SENSITIZATION_BY_LDL	0.09	4E-04
REACTOME_TRANSPORT_OF_INORGANIC_CATIONS_ANIONS_AND_AMINO_ACIDS_OLIGOPEPTIDES	0.08	4E-02
REACTOME_AMYLOIDS	0.07	3E-03
REACTOME_AQUAPORIN_MEDIATED_TRANSPORT	0.06	1E-01
REACTOME_CHYLOMICRON_MEDIATED_LIPID_TRANSPORT	0.06	9E-03
REACTOME_PTM_GAMMA_CARBOXYLATION_HYPUSINE_FORMATION_AND_ARYLSULFATASE_ACTIVATION	0.06	6E-02
REACTOME_PYRUVATE_METABOLISM	0.06	6E-02
REACTOME_INSULIN_SYNTHESIS_AND_PROCESSING	0.06	2E-01
REACTOME_VOLTAGE_GATED_POTASSIUM_CHANNELS	0.05	2E-01
REACTOME_PHOSPHOLIPASE_C_MEDIATED_CASCADE	0.05	2E-01
REACTOME_PLATELET_CALCIUM_HOMEOSTASIS	0.05	1E-01
REACTOME_TRANSPORT_OF_GLUCOSE_AND_OTHER_SUGARS_BILE_SALTS_AND_ORGANIC_ACIDS_METAL_IONS_AND_AMINE_COMPOUNDS	0.05	2E-01
REACTOME_ACTIVATION_OF_CHAPERONES_BY_ATF6_ALPHA	0.05	8E-02
REACTOME_REGULATION_OF_INSULIN_SECRETION_BY_ACETYLCHOLINE	0.04	1E-01
REACTOME_IRON_UPTAKE_AND_TRANSPORT	0.04	1E-01
REACTOME_GLYCOLYSIS	0.04	2E-01
REACTOME_GLYCOSPHINGOLIPID_METABOLISM	0.03	2E-01
REACTOME_SYNTHESIS_OF_SUBSTRATES_IN_N_GLYCAN_BIOSYTHESIS	0.03	2E-01
REACTOME_METABOLISM_OF_NUCLEOTIDES	0.02	3E-01
REACTOME_METABOLISM_OF_STEROID_HORMONES_AND_VITAMINS_A_AND_D	0.02	4E-01
REACTOME_E2F_MEDIATED_REGULATION_OF_DNA_REPLICATION	0.02	4E-01
REACTOME_GLUCURONIDATION	0.02	3E-01
REACTOME_PI_METABOLISM	0.01	4E-01
REACTOME_METAL_ION_SLC_TRANSPORTERS	0.01	4E-01
REACTOME_DNA_REPLICATION	0.01	4E-01
REACTOME_MITOTIC_G1_G1_S_PHASES	0.01	4E-01
REACTOME_SIGNALING_BY_TGF_BETA_RECEPTOR_COMPLEX	0.01	4E-01
REACTOME_GLUCOSE_TRANSPORT	0.00	5E-01
REACTOME_TRANSPORT_OF_VITAMINS_NUCLEOSIDES_AND_RELATED_MOLECULES	0.00	5E-01
REACTOME_SYNTHESIS_OF_DNA	0.00	5E-01
REACTOME_ACTIVATION_OF_THE_PRE_REPLICATIVE_COMPLEX	-0.01	4E-01
REACTOME_EFFECTS_OF_PIP2_HYDROLYSIS	-0.02	4E-01
REACTOME_AUTODEGRADATION_OF_THE_E3_UBIQUITIN_LIGASE_COP1	-0.02	3E-01
REACTOME_PURINE_METABOLISM	-0.02	2E-01
REACTOME_CYTOCHROME_P450_ARRANGED_BY_SUBSTRATE_TYPE	-0.03	1E-01
REACTOME_PURINE_CATABOLISM	-0.03	3E-01
REACTOME_N_GLYCAN_TRIMMING_IN_THE_ER_AND_CALNEXIN_CALRETICULIN_CYCLE	-0.04	2E-01
REACTOME_SPHINGOLIPID_METABOLISM	-0.05	2E-01
REACTOME_COPI_MEDIATED_TRANSPORT	-0.05	3E-02
REACTOME_TELOMERE_MAINTENANCE	-0.05	1E-01
REACTOME_RNA_POL_I_TRANSCRIPTION	-0.06	4E-02
REACTOME_ACYL_CHAIN_REMODELLING_OF_PI	-0.07	1E-01
REACTOME_GLUCOSE_METABOLISM	-0.08	1E-03
REACTOME_REGULATION_OF_ORNITHINE_DECARBOXYLASE_ODC	-0.08	1E-02
REACTOME_CYTOSOLIC_TRNA_AMINOACYLATION	-0.08	2E-02
REACTOME_METABOLISM_OF_PORPHYRINS	-0.08	5E-03
REACTOME_LYSOSOME_VESICLE_BIOGENESIS	-0.08	2E-02
REACTOME_MRNA_DECAY_BY_5_TO_3_EXORIBONUCLEASE	-0.10	3E-03
REACTOME_TRANSLATION	-0.10	1E-02
REACTOME_GLYCOGEN_BREAKDOWN_GLYCOGENOLYSIS	-0.10	7E-03
REACTOME_RNA_POL_I_RNA_POL_III_AND_MITOCHONDRIAL_TRANSCRIPTION	-0.10	4E-03

REACTOME_GLUCONEOGENESIS	-0.10	3E-04
REACTOME_SYNTHESIS_OF_VERY_LONG_CHAIN_FATTY_ACYL_COAS	-0.10	7E-02
REACTOME_PPARA_ACTIVATES_GENE_EXPRESSION	-0.10	3E-02
REACTOME_PURINE_RIBONUCLEOSIDE_MONOPHOSPHATE_BIOSYNTHESIS	-0.10	2E-02
REACTOME_METABOLISM_OF_POLYAMINES	-0.10	8E-04
REACTOME_SYNTHESIS_AND_INTERCONVERSION_OF_NUCLEOTIDE_DI_AND_TRIPHOSPHATES	-0.11	6E-03
REACTOME_TERMINATION_OF_O_GLYCAN_BIOSYNTHESIS	-0.11	1E-03
REACTOME_AMINE_DERIVED_HORMONES	-0.11	1E-02
REACTOME_BIOSYNTHESIS_OF_THE_N_GLYCAN_PRECURSOR_DOLICHOL_LIPID_LINKED_OLIGOSACCHARIDE_LLO_AND_TRANSFER_TO_A_NASCENT_PROTEIN	-0.12	1E-03
REACTOME_O_LINKED_GLYCOSYLATION_OF_MUCINS	-0.13	8E-07
REACTOME_SYNTHESIS_OF_BILE_ACIDS_AND_BILE_SALTS_VIA_24_HYDROXYCHOLESTEROL	-0.13	4E-05
REACTOME_ZINC_TRANSPORTERS	-0.13	3E-02
REACTOME_SPHINGOLIPID_DE_NOVO_BIOSYNTHESIS	-0.13	2E-03
REACTOME_3_UTR_MEDIATED_TRANSLATIONAL_REGULATION	-0.13	1E-03
REACTOME_SYNTHESIS_OF_BILE_ACIDS_AND_BILE_SALTS	-0.13	2E-07
REACTOME_METABOLISM_OF_PROTEINS	-0.13	6E-05
REACTOME_ACYL_CHAIN_REMODELLING_OF_PC	-0.13	1E-02
REACTOME_ANDROGEN_BIOSYNTHESIS	-0.14	5E-03
REACTOME_REGULATION_OF_GLUCOKINASE_BY_GLUCOKINASE_REGULATORY_PROTEIN	-0.14	3E-03
REACTOME_TRANSPORT_OF_RIBONUCLEOPROTEINS_INTO_THE_HOST_NUCLEUS	-0.14	2E-03
REACTOME_ALPHA_LINOLENIC_ACID_ALA_METABOLISM	-0.15	3E-06
REACTOME_AMINO_ACID_SYNTHESIS_AND_INTERCONVERSION_TRANSAMINATION	-0.15	6E-04
REACTOME_TETRAHYDROBIOPTERIN_BH4_SYNTHESIS_RECYCLING_SALVAGE_AND_REGULATION	-0.16	1E-09
REACTOME_ACYL_CHAIN_REMODELLING_OF_PE	-0.16	1E-03
REACTOME_AMINE_COMPOUND_SLC_TRANSPORTERS	-0.16	4E-05
REACTOME_RECYCLING_OF_BILE_ACIDS_AND_SALTS	-0.16	2E-05
REACTOME_METABOLISM_OF_VITAMINS_AND_COFACTORS	-0.16	3E-07
REACTOME_ABCA_TRANSPORTERS_IN_LIPID_HOMEOSTASIS	-0.16	9E-03
REACTOME_METABOLISM_OF_MRNA	-0.17	2E-04
REACTOME_BILE_ACID_AND_BILE_SALT_METABOLISM	-0.17	2E-09
REACTOME_CELL_CELL_JUNCTION_ORGANIZATION	-0.18	1E-03
REACTOME_MRNA_DECAY_BY_3_TO_5_EXORIBONUCLEASE	-0.18	3E-06
REACTOME_ACYL_CHAIN_REMODELLING_OF_PG	-0.18	4E-03
REACTOME_METABOLISM_OF_RNA	-0.18	5E-05
REACTOME_ACYL_CHAIN_REMODELLING_OF_PS	-0.18	9E-04
REACTOME_ORGANIC_CATION_ANION_ZWITTERION_TRANSPORT	-0.18	7E-05
REACTOME_METABOLISM_OF_NON_CODING_RNA	-0.18	8E-05
REACTOME_DNA_REPAIR	-0.19	4E-04
REACTOME_SYNTHESIS_OF_PA	-0.19	4E-06
REACTOME_DIGESTION_OF_DIETARY_CARBOHYDRATE	-0.20	2E-04
REACTOME_DEADENYLATION_DEPENDENT_MRNA_DECAY	-0.22	5E-11
REACTOME_PROCESSING_OF_INTRONLESS_PRE_MRNAS	-0.22	2E-07
REACTOME_REGULATION_OF_PYRUVATE_DEHYDROGENASE_PDH_COMPLEX	-0.22	4E-18
REACTOME_FORMATION_OF_ATP_BY_CHEMIOSMOTIC_COUPLING	-0.23	3E-06
REACTOME_TGF_BETA_RECEPTOR_SIGNALING_IN_EMT_EPITHELIAL_TO_MESENCHYMAL_TRANSITION	-0.23	1E-06
REACTOME_RNA_POL_I_TRANSCRIPTION_TERMINATION	-0.23	3E-19
REACTOME_FATTY_ACYL_COA_BIOSYNTHESIS	-0.23	8E-05
REACTOME_ION_CHANNEL_TRANSPORT	-0.24	2E-09
REACTOME_TRNA_AMINOACYLATION	-0.24	3E-13
REACTOME_PHOSPHOLIPID_METABOLISM	-0.24	8E-09
REACTOME_MRNA_SPLICING_MINOR_PATHWAY	-0.24	1E-08
REACTOME_METABOLISM_OF_AMINO_ACIDS_AND_DERIVATIVES	-0.24	8E-17
REACTOME_GOLGI_ASSOCIATED_VESICLE_BIOGENESIS	-0.25	2E-15
REACTOME_SYNTHESIS_OF_BILE_ACIDS_AND_BILE_SALTS_VIA_7ALPHA_HYDROXYCHOLESTEROL	-0.25	1E-22
REACTOME_PYRUVATE_METABOLISM_AND_CITRIC_ACID_TCA_CYCLE	-0.25	3E-23
REACTOME_RNA_POL_III_TRANSCRIPTION	-0.26	1E-13
REACTOME_DEADENYLATION_OF_MRNA	-0.26	3E-24
REACTOME_RNA_POL_II_TRANSCRIPTION	-0.27	3E-10

REACTOME_MRNA_PROCESSING	-0.27	3E-09
REACTOME_ETHANOL_OXIDATION	-0.27	9E-25
REACTOME_ION_TRANSPORT_BY_P_TYPE_ATPASES	-0.28	4E-12
REACTOME_GLUTATHIONE_CONJUGATION	-0.28	2E-11
REACTOME_RNA_POL_III_TRANSCRIPTION_INITIATION_FROM_TYPE_2_PROMOTER	-0.28	2E-29
REACTOME_MRNA_SPLICING	-0.29	8E-10
REACTOME_ABC_FAMILY_PROTEINS_MEDIATED_TRANSPORT	-0.29	2E-06
REACTOME_METABOLISM_OF_LIPIDS_AND_LIPOPROTEINS	-0.30	2E-12
REACTOME_BASE_FREE_SUGAR_PHOSPHATE_REMOVAL_VIA_THE_SINGLE_NUCLEOTIDE_REPLACEMENT_PATHWAY	-0.30	8E-09
REACTOME_SULFUR_AMINO_ACID_METABOLISM	-0.31	3E-13
REACTOME_RESPIRATORY_ELECTRON_TRANSPORT_ATP_SYNTHESIS_BY_CHEMIOSMOTIC_COUPLING_AND_HEAT_PRODUCTION_BY_UNCOUPLING_PROTEINS	-0.32	2E-17
REACTOME_TCA_CYCLE_AND_RESPIRATORY_ELECTRON_TRANSPORT	-0.32	6E-23
REACTOME_TRIGLYCERIDE_BIOSYNTHESIS	-0.32	8E-29
REACTOME_RESPIRATORY_ELECTRON_TRANSPORT	-0.33	4E-22
REACTOME_CITRIC_ACID_CYCLE_TCA_CYCLE	-0.33	3E-23
REACTOME_PEROXISOMAL_LIPID_METABOLISM	-0.36	3E-28
REACTOME_FATTY_ACID_TRIACYLGLYCEROL_AND_KETONE_BODY_METABOLISM	-0.36	2E-15
REACTOME_MITOCHONDRIAL_TRNA_AMINOACYLATION	-0.37	8E-48
REACTOME_SYNTHESIS_OF_PC	-0.38	5E-22
REACTOME_GLYCEROPHOSPHOLIPID_BIOSYNTHESIS	-0.38	2E-13
REACTOME_VITAMIN_B5_PANTOTHENATE_METABOLISM	-0.38	2E-26
REACTOME_SYNTHESIS_OF_PE	-0.38	2E-09
REACTOME_CHOLESTEROL_BIOSYNTHESIS	-0.40	2E-39
REACTOME_SYNTHESIS_OF_GLYCOSYLPHOSPHATIDYLINOSITOL_GPI	-0.46	2E-20
REACTOME_ACTIVATED_AMPK_STIMULATES_FATTY_ACID_OXIDATION_IN_MUSCLE	-0.47	1E-27
REACTOME_BRANCHED_CHAIN_AMINO_ACID_CATABOLISM	-0.48	8E-14
REACTOME_MITOCHONDRIAL_FATTY_ACID_BETA_OXIDATION	-0.50	6E-18

Appendix Table S6. List of marker genes in each cell type cluster identified from single cell RNA sequencing of parental A549 cells

Cluster_0	Cluster_1	Cluster_2		Cluster_3		Cluster_4		
FXYD2	HMOX1	AGR2	SPDEF	CEACAM6	GACAT2	PMEPA1	INPP4B	PGRMC2
CD24	AKAP12	SLC12A2	TST	KRT19	CALB2	CCDC80	LBH	ARHGEF18
AXL	ID2	FN1	PTGS2	AGR2	NET1	TGFBI	EPHB2	ACSL4
KRT19	RSPO3	MUC5AC	IFITM2	PRSS3	CDC42EP3	IGFBP7	MARCKSL1	SEMA3C
MMP7	CTSB	AKR1C1	FOS	MUC5AC	GALNT5	SERPINE1	JAG1	MAP7
EEF1A2	TESC	CPLX2	PLS1	ITGB4	LMO7	TAGLN	CDK6	MFGE8
S100A3	KYNU	CEACAM6	COL5A2	FGFBP1	SLCO1B3	GLIPR1	ANKLE2	NUAK1
	GDF15	PDLIM5	CLDN2	CAVIN3	ITGA2	SOX4	HMGA2	EEA1
	EFHD2	MUC5B	TSPAN13	CRIP2	JUP	COL4A2	FGF2	GRB10
	MFF	LCN2	KLF13	CAPG	PCED1B	JUNB	IGF1R	DCBLD1
	NAMPT	ANXA13	STEAP1	LCN2	EPS8	PCED1B	SKIL	MOB3B
	CYP24A1	SYT1	LRP10	SOX4	ADD3	PDLIM7	IL11	FST
	GPC1	RAP1GAP	JUP	CNTN1	GAL3ST1	PGM2L1	SPOCK1	SCN9A
	PAQR5	EPHX1	PLA2G4A	VAMP8	KRT15	PLEK2	PHLDB2	DBN1
	TNNT1	MMP7	HLA-DMB	SYT1	MDK	CITED4	C15orf48	
	EPAS1	CYP1B1	SMIM14	PLEK2		TGM2	PALLD	
	CCND3	MTUS1	BCAS1	RHOD		COL5A1	IL32	
	SRGN	CCPG1	RNASE4	ZNF185		ETS2	ANTXR1	
	CEBPB	SLC9A3R2		ITPRID2		SLC26A2	CDC42EP3	
	KCNMA1	EPS8		ITGA6		CDH2	RAI14	
	HSPA2	CFH		MUC5B		GLS	CADM1	
	AGFG1	SLPI		SERPINB1		NCOR2	LAMC2	
	ATIC	TM4SF20		ETHE1		NRP2	GALNT10	
	SFRP1	SCP2		TMBIM4		NNMT	THBS1	
	RHOQ	ARHGAP18		UPK1B		RAB3B	CEP170	
	DARS	CAMK2N1		ANXA13		FOXP1	JUN	
	NPC1	BAMBI		MARCKSL1		KCNMA1	EPB41L2	
	PMP22	AQP3		ALDH2		FRMD6	GREM1	
	MAP3K20	CNTN1		IGFL2-AS1		COL4A1	ITGA2	
	ADM	INSL4		MMP7		MT1X	TANC2	
	HILPDA	RDH10		PLAUR		TGFB1I1	LYPD1	
	STAT1	TPD52L1		LAMB3		DUSP1	TNFAIP8	
	IGFBP2	S100P		NT5E		PODXL	PACS1	
	SLC16A3	FGL1		AP1S3		SPDL1	CCN2	

Appendix Figure S1

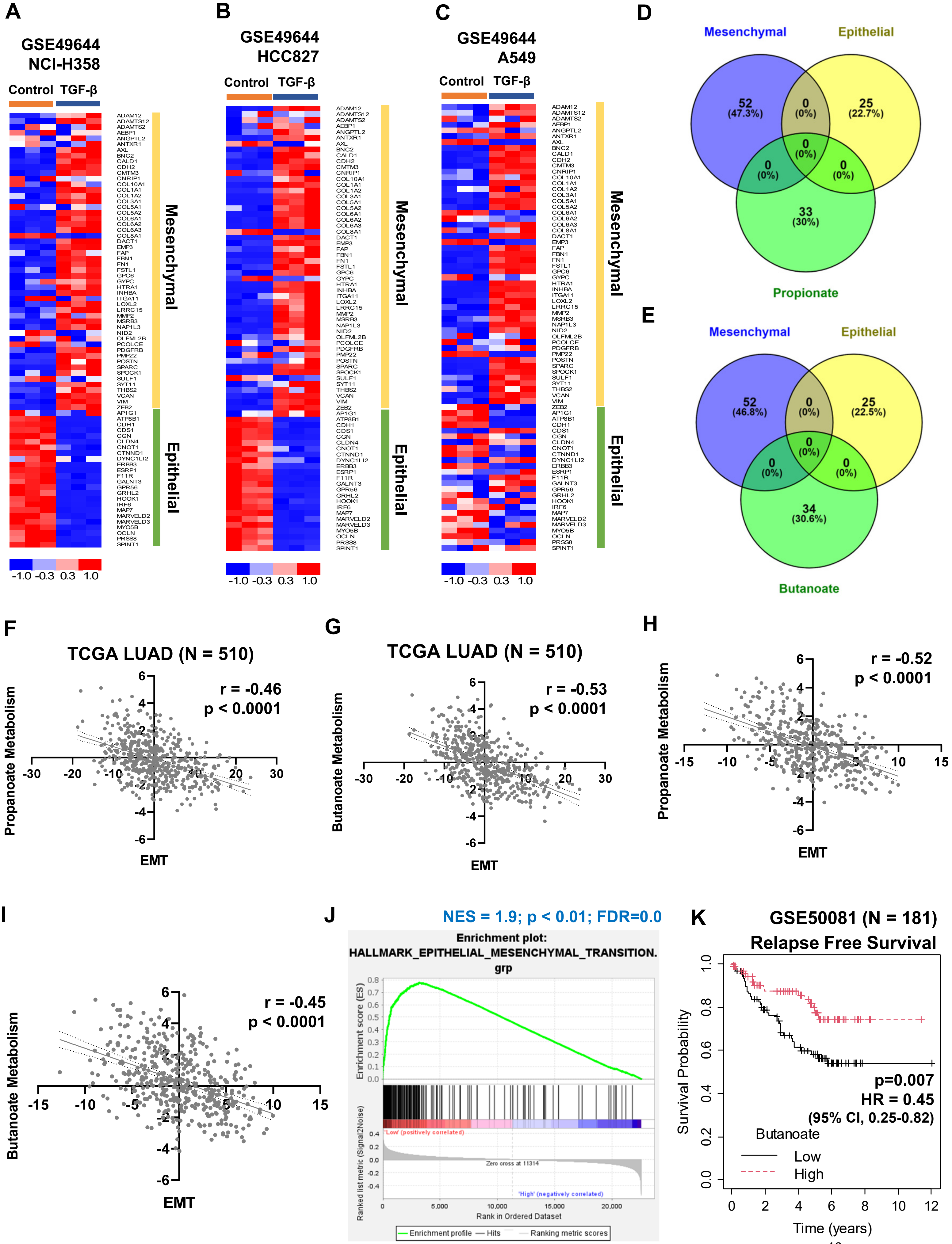

Appendix Figure S1. Integrative genomic analysis identified negative association between propionate or butanoate with EMT in lung cancer gene expression profiles.

A-C. Heatmap represents the gene expression pattern of pan-cancer EMT gene signature showing an increase in the mesenchymal genes with a decrease in the epithelial genes in the TGF-β1 treated NSCLC cells (n=3). Gene expression profile of NSCLC cell lines (NCI-H358 (A), HCC827 (B) and A549 (C)) treated with TGF-β1 for 3 weeks were obtained from GEO (GSE49644).

D, E. Venn diagram representation of gene content overlap between EMT gene signature (mesenchymal and epithelial genes) and propionate (D) or butanoate (E) gene sets.

F, G. Correlation plot of short-chain fatty acids (propanoate (F) or butanoate (G)) with EMT gene signature in lung adenocarcinoma patients (N=510) from The Cancer Genome Atlas (TCGA).

H, I. Correlation plot of activation scores of short-chain fatty acids (propanoate (H) and butanoate (I)) with a different source of EMT gene signature (Jechlinger EMT gene signature) obtained from MSigDB in lung cancer patient samples obtained from GEO (GSE72094, N=442).

J. Gene-set enrichment analysis of hallmark EMT gene-sets with the lung cancer patient samples (GSE72094; N=442) categorized as low and high based on the butanoate gene-set activation levels showed EMT enrichment in low butanoate patient samples. Ranking of genes with signal2noise metric was used for GSEA.

K. Relapse free survival analysis in lung cancer patient samples (GSE50081 (N=181)) categorized as low- and high-butanoate levels based on the median showed good prognosis for butanoate gene set. HR – Hazard ratio for high butanoate group was calculated using Cox proportional hazards model. p-value was calculated using log-rank method.

Appendix Figure S2

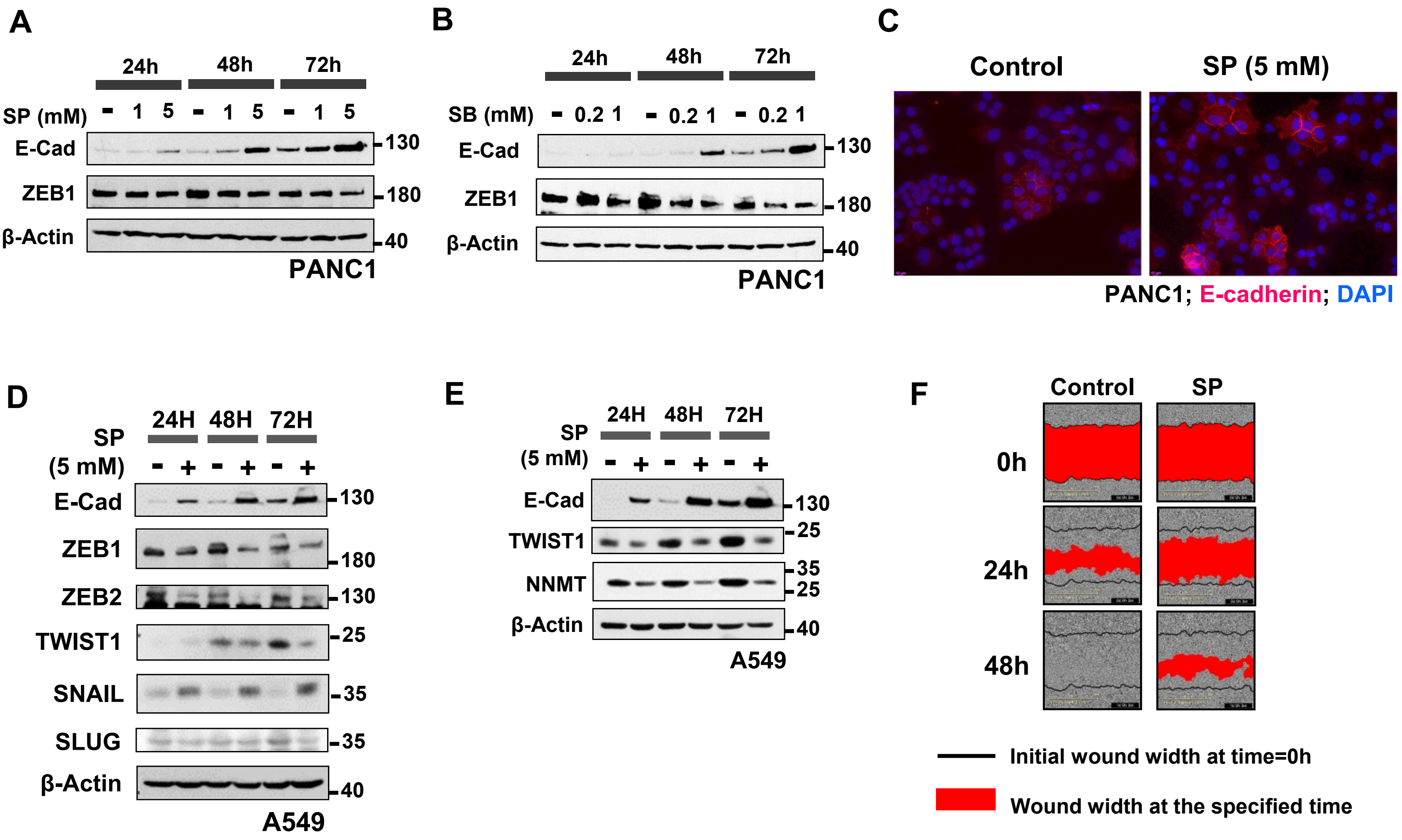

Appendix Figure S2. In vitro treatment effect of SCFAs, propionate or butanoate, in EMT marker gene expression

A, B. Western blot analysis of E-cadherin and ZEB1 in PANC1 cells treated with sodium propionate (A) or sodium butanoate (B) in the indicated dose- and time-dependent manner. β-Actin was used as an internal control. The experiments were performed three independent times.

C. Immunofluorescence staining of E-cadherin in PANC1 cell line treated with sodium propionate (5 mM) for 3 days. DAPI was used as a nuclear stain. Scale bars: 10 μm.

D. Western blot analysis of E-cadherin and EMT-associated transcription factors (ZEB1, ZEB2, TWIST1, SNAIL and SLUG) in A549 cells treated with sodium propionate (5 mM) in the indicated time-dependent manner. β-Actin was used as an internal control. The experiment was performed two independent times.

E. Western blot analysis of E-cadherin, TWIST1 and NNMT protein levels in A549 cells treated with sodium propionate (SP, 5 mM) in the indicated time-dependent manner. β-Actin was used as an internal control. The experiment was performed three independent times.

F. Images represent the wound width (highlighted by red region) at the indicated time points in A549 cells treated with sodium propionate (SP) for 3 days for migration assay.

Appendix Figure S3

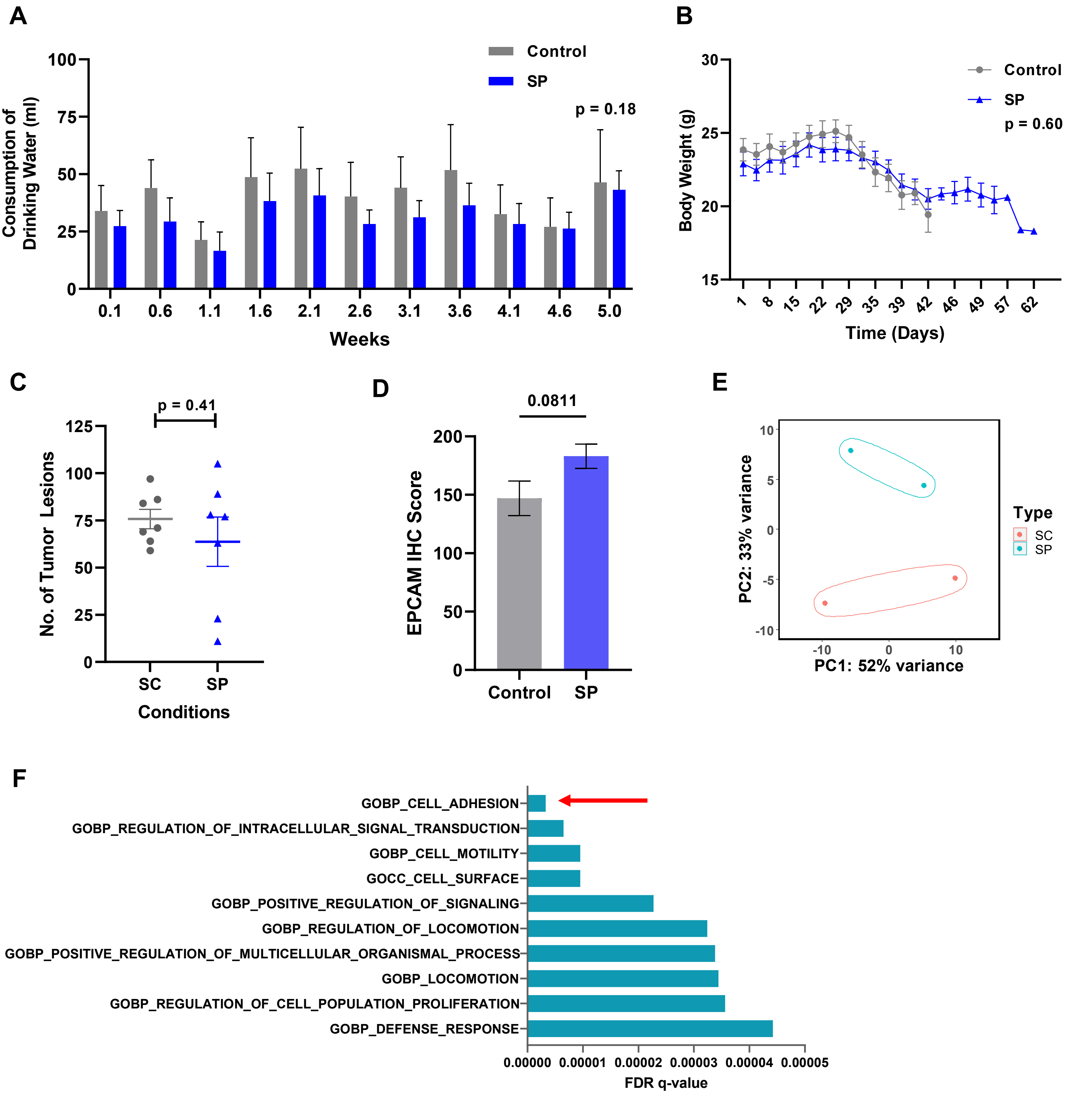

Appendix Figure S3. Effect of sodium propionate on lung tumorigenesis in KPL virus intubated Cas9-C57BL/6 mice administered orally with sodium propionate in drinking water.

A. Bar plot of consumption of drinking water containing sodium propionate (150 mM) or sodium chloride (150 mM) by KPL mice over the course of 5 weeks. Sodium propionate (150 mM) or sodium chloride (150 mM) was freshly prepared in drinking water by changing the water twice a week for mice. Data points (n=6 cages per group) are represented as mean ± SEM and significance was calculated using Two-way ANOVA.

B. Line plot of body weight measurements over the course of study of Cas9-C57BL/6 mice with lung tumorigenesis induced with AAV-KPL virus. Data points (n=10) are represented as mean ± SD. Sodium propionate (150 mM) or sodium chloride (150 mM) was administered orally in drinking water.

C. Comparison of number of tumor lesions from the H&E staining of lungs of Cas9-C57BL/6 mice with lung tumorigenesis induced with AAV-KPL virus and orally administered with sodium chloride (SC) or sodium propionate (SP). Data points (n=7 per group) represent mean ± SEM. Significance was calculated using unpaired t-test.

D. Immunohistochemistry staining of EPCAM in lung tumor tissues of KPL-mouse administered with SP. Data points (n=5 per group) are represented as mean ± SEM and significance was calculated using unpaired t-test.

E. Principal component analysis of RNA-seq expression profile (n=2 per group) of lung tumor tissue samples from Cas9-C57BL/6 mice intubated with AAV-KPL virus and administered with sodium propionate (SP) in drinking water.

F. Gene-set ontology of differentially expressed genes from RNA-seq expression profile of lung tumor tissue samples from Cas9-C57BL/6 mice intubated with AAV-KPL virus and administered with sodium propionate (SP) in drinking water. Red arrow indicates the top significantly enriched process.

Appendix Figure S4

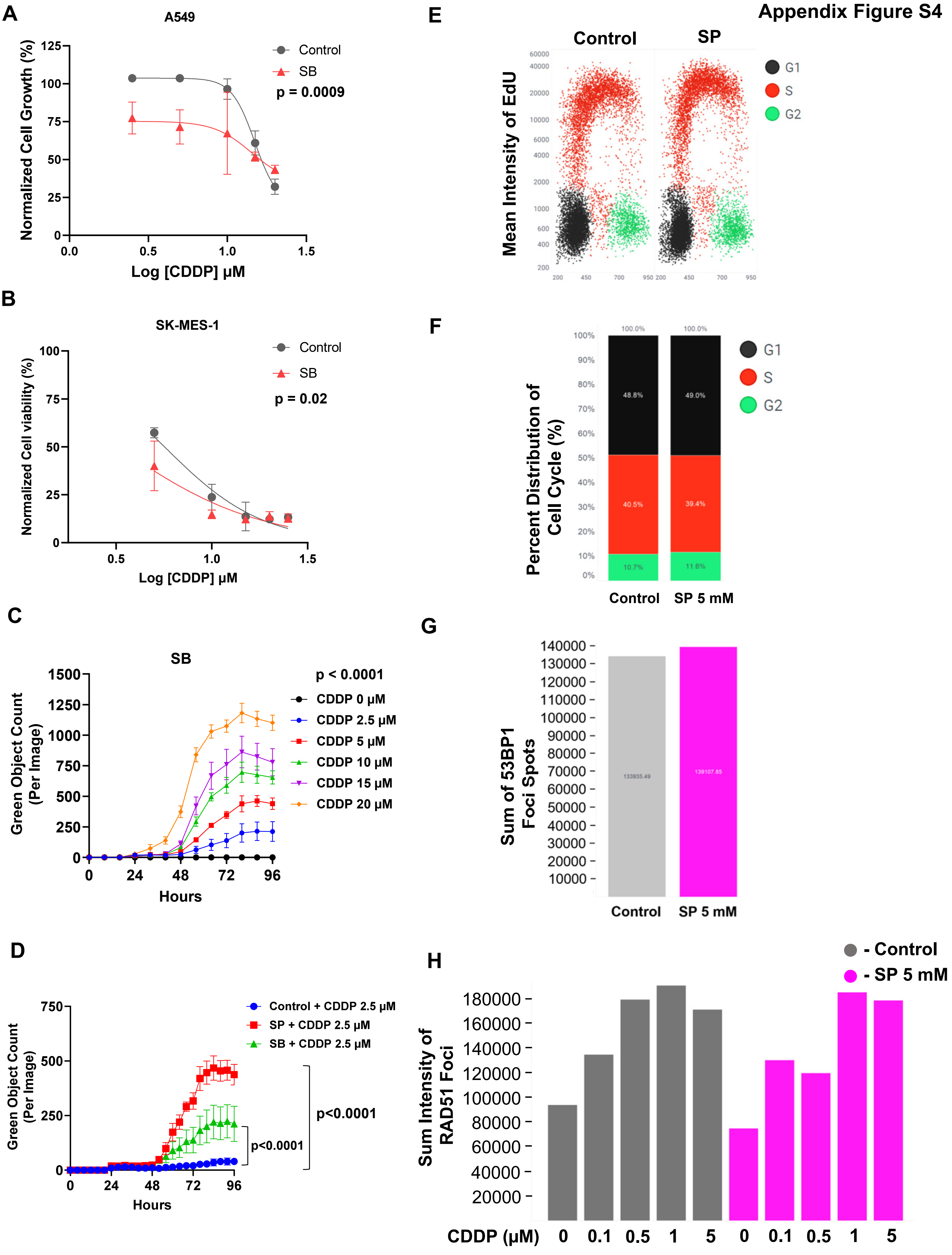

Appendix Figure S4. SCFAs, propionate or butanoate, sensitizes the cells to cisplatin treatment in NSCLC cell lines.

A, B. Dose responsive curve of cisplatin treatment in combination with sodium butanoate (SB, 1 mM) in A549 (A) or SKMES1 (B) cell lines with pre-treatment condition for 48 hours. Data points (n=3) are represented as mean ± SD with p-value calculated from Two-way ANOVA analysis. The experiment was performed three independent times.

C. Quantification of dead cells as green object count using Cytotox Green in A549 cells treated with dose-dependent cisplatin in combination with 1 mM SB with pre-treatment condition for 48 hours. Data points (n=3) are represented as mean ± SD of one experiment and the experiment was performed three independent times. p-value was calculated from Two-way ANOVA analysis.

D. Line plot comparison of the green object count for the cells treated with cisplatin (2.5 μM) in combination with SP (5 mM) or SB (1 mM). Data points (n=3) are represented as mean ± SD of one experiment and the experiment was performed three independent times. Significance was calculated from Two-way ANOVA followed by multiple comparison between conditions using Tukey's multiple comparison test.

E, F. QIBC analysis of cell cycle phases segregated based on Edu staining in A549 cells (n=~9000 single cells) treated with sodium propionate (5 mM) for 24 hours (E) and stacked bar plot represents the percent distribution levels of cell cycle phases (G1, S and G2) in A549 cell lines treated with sodium propionate (5 mM) for 24 hours (F).

G. QIBC analysis of sum intensity levels of 53BP1 foci spots in A549 cells (n=~9000 single cells) treated with SP (5 mM) plotted as bar plot indicating no changes in the levels between the conditions.

H. QIBC analysis of sum intensity levels of RAD51 foci in A549 cells (n=~9000 single cells) plotted as bar plot with the treatment of cisplatin (CDDP) in the indicated dose-dependent concentrations in combination with SP (5 mM).

Appendix Figure S5

A

Cluster	No. of Cells
0	2065
1	1523
2	946
3	363
4	306

B

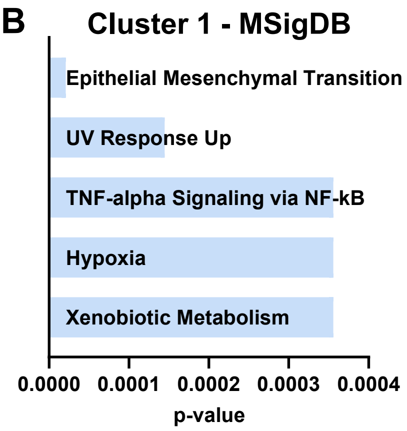

C

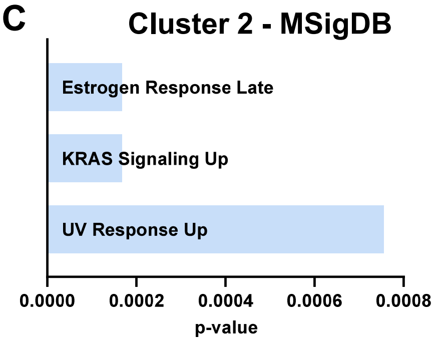

D

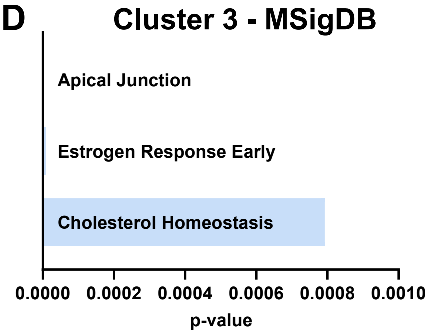

E

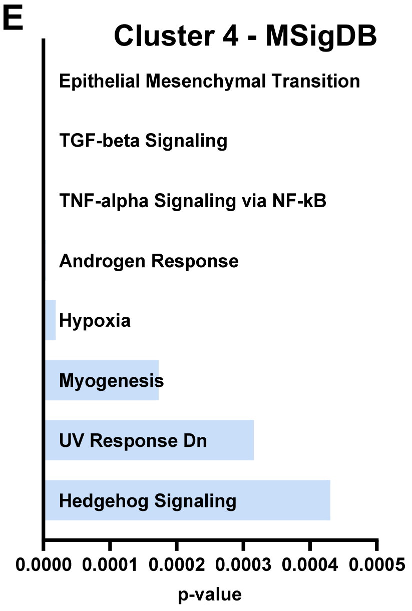

F

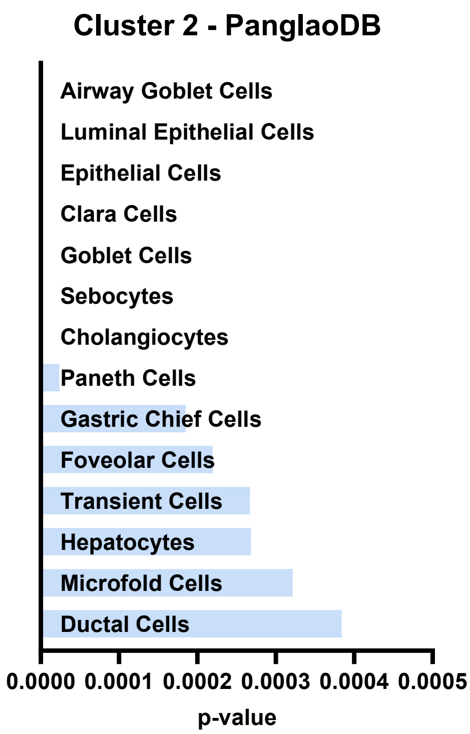

G

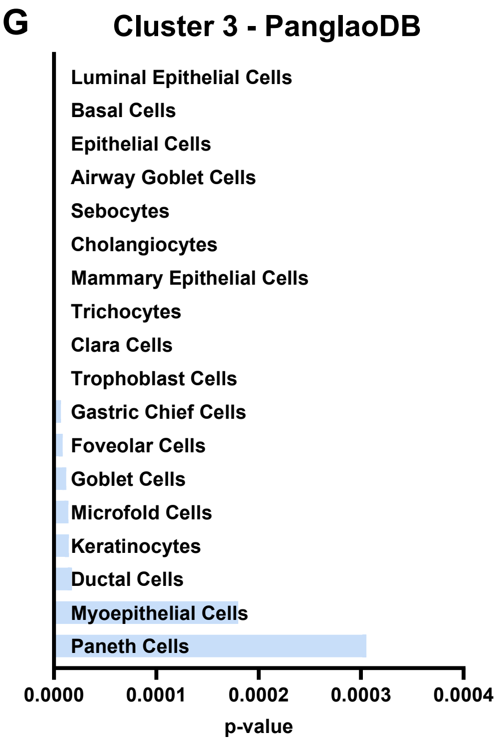

H

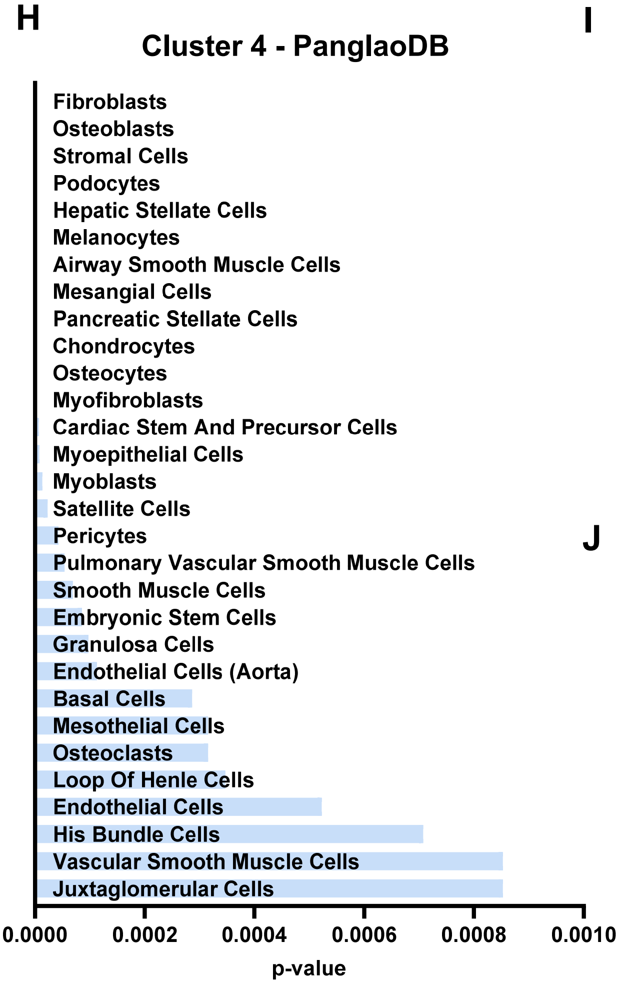

I

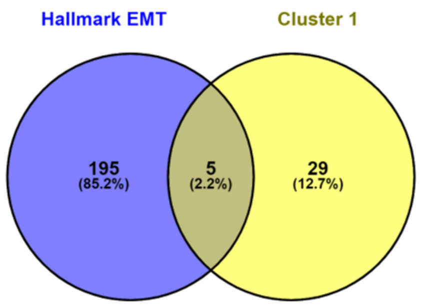

5 common genes in "Cluster 1" and "Hallmark EMT":

ID2
GPC1
SFRP1
PMP22
IGFBP2

J

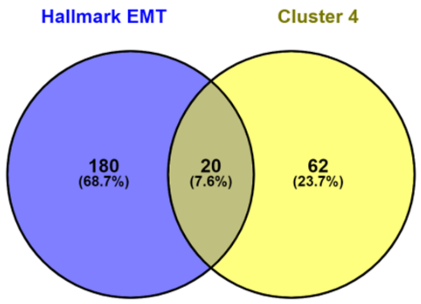

20 common genes in "Cluster 4" and "Hallmark EMT":

PMEPA1	COL4A1
TGFBI	FGF2
SERPINE1	SPOCK1
TAGLN	IL32
GLIPR1	CADM1
COL4A2	LAMC2
TGM2	THBS1
COL5A1	JUN
CDH2	GREM1
NNMT	ITGA2

Appendix Figure S5. Single cell RNA-seq of untreated parental A549 cell line shows cell clusters of epithelial and mesenchymal cell type enrichment.

A. Table list the number of cells identified in each cluster of parental A549 cells sequenced by single cell RNA sequencing.

B-H. Enrichr based gene enrichment analysis of marker genes of cell clusters 1 to 4 identified from scRNA-seq of A549 cells for MSigDB gene-sets collection (B-E), and PanglaoDB (F-H) gene-sets collection. Significant enrichment was considered with p-value < 0.001 computed using Fisher's exact test.

I, J. Venn diagram representation of overlap analysis between hallmark EMT gene signature and cluster 1 marker genes (I) or cluster 4 marker genes (J). Overlapping genes are listed below the respective venn diagrams.

Appendix Figure S6

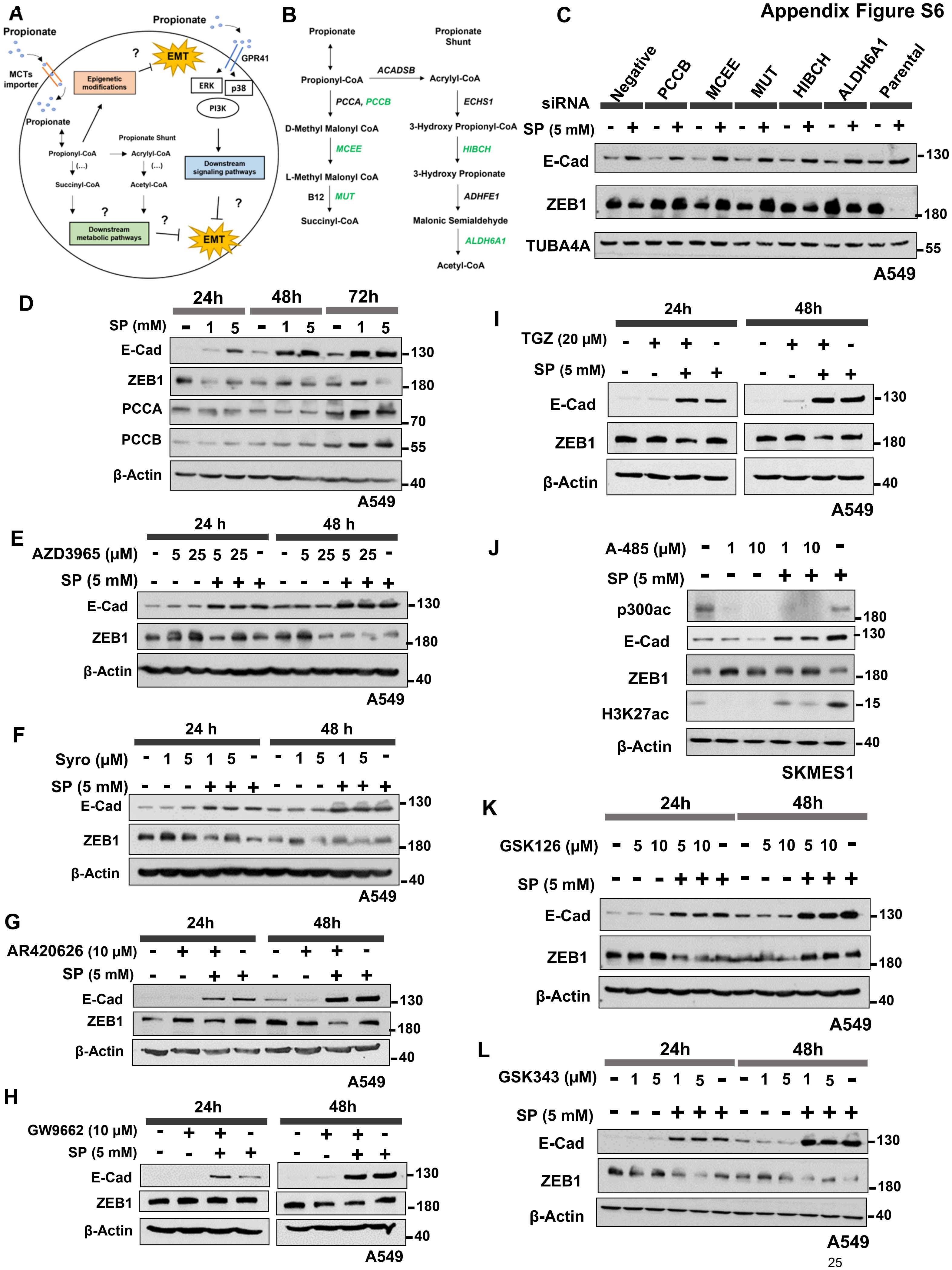

Appendix Figure S6. Identification of possible mechanistic role of propionate's action in E-cadherin's increased expression.

A. Schematic representation of possible modes of propionate's mechanism of action in the cell involving MCT transporters, epigenetic mechanism, metabolic pathway, and GPR41 signaling.

B. Schematic representation of propionate metabolic pathway with the enzymes involved in the metabolic conversions. Green highlighted enzymatic genes were used for siRNA-mediated knockdown study.

C. Western blot analysis of E-cadherin and ZEB1 in A549 cells knock down with propionate-specific metabolic genes (*PCCB*, *MCEE*, *MUT*, *HIBCH* and *ALDH6A1*) for 48 hours followed by treatment with sodium propionate for 24 hours. Non-targeting siRNA was used as a control. TUBA4A was used as an internal control.

D. Western blot analysis of E-cadherin, ZEB1 and propionate metabolic genes (PCCA and PCCB) in A549 cell line treated with sodium propionate (SP) in the indicated dose- and time-dependent manner. β-Actin was used as an internal control.

E, F. Western blot analysis of E-cadherin and ZEB1 in A549 cells treated with MCT transport inhibitors, AZD3965 (E) and Syrosingopine (F), in the indicated dose-dependent concentrations in combination with sodium propionate (SP, 5 mM) for the indicated time points. β-Actin was used as an internal control.

G. Western blot analysis of E-cadherin and ZEB1 in A549 cells treated with GPR41 modulator, AR420626 (10 µM) in combination with sodium propionate (SP, 5 mM) for the indicated time points. β-Actin was used as an internal control.

H, I. Western blot analysis of E-cadherin and ZEB1 in A549 cell line treated with PPARγ inhibitor, GW9662 (H) and PPARγ activator, Troglitazone (I), in the indicated concentration in combination with sodium propionate (SP, 5 mM) for the indicated time points. β-Actin was used as an internal control.

J. Western blot analysis of p300 acetylation, E-cadherin, ZEB1 and H3K27ac in SKMES1 cells treated with A-485 (10 µM) in combination with sodium propionate (SP, 5 mM) for 24 hours. β-Actin was used as an internal control.

K, L. Western blot analysis of E-cadherin and ZEB1 in A549 cells treated with H3K27me3 inhibitors, GSK126 (K) and GSK343 (L), in the indicated dose-dependent concentrations in combination with sodium propionate (SP, 5 mM) for the indicated time points. β-Actin was used as an internal control.

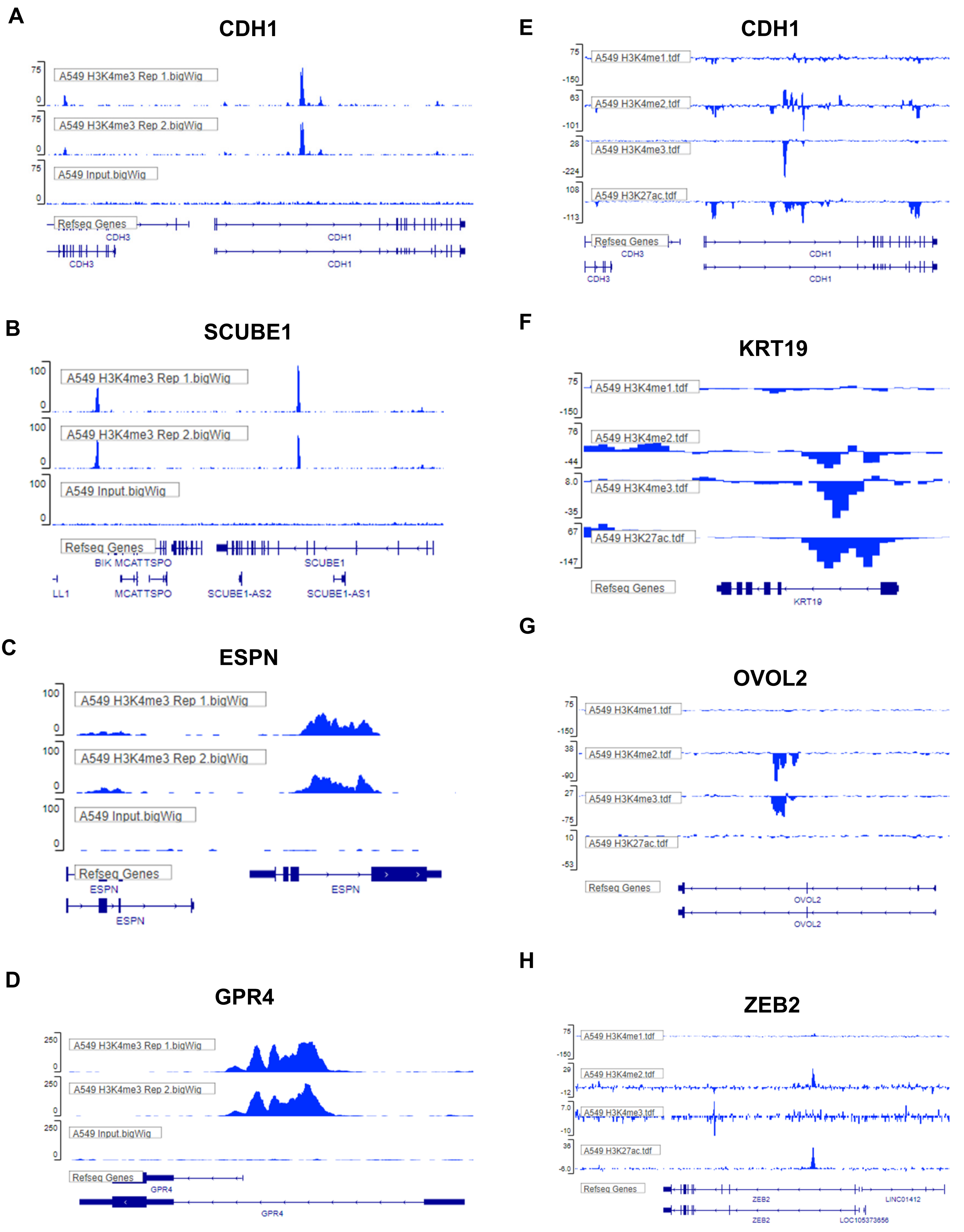
A
CDH1
A549 H3K4me3 Rep 1.bigWig
A549 H3K4me3 Rep 2.bigWig
A549 Input.bigWig
Refseq Genes
CDH3
CDH1
B
SCUBE1
A549 H3K4me3 Rep 1.bigWig
A549 H3K4me3 Rep 2.bigWig
A549 Input.bigWig
Refseq Genes
BIK MCATTSPO
SCUBE1
LL1
MCATTSPO
SCUBE1-AS2
SCUBE1-AS1
C
ESPN
A549 H3K4me3 Rep 1.bigWig
A549 H3K4me3 Rep 2.bigWig
A549 Input.bigWig
Refseq Genes
ESPN
D
GPR4
A549 H3K4me3 Rep 1.bigWig
A549 H3K4me3 Rep 2.bigWig
A549 Input.bigWig
Refseq Genes
GPR4
E
CDH1
A549 H3K4me1.tdf
A549 H3K4me2.tdf
A549 H3K4me3.tdf
A549 H3K27ac.tdf
Refseq Genes
CDH3
CDH1
F
KRT19
A549 H3K4me1.tdf
A549 H3K4me2.tdf
A549 H3K4me3.tdf
A549 H3K27ac.tdf
Refseq Genes
KRT19
G
OVOL2
A549 H3K4me1.tdf
A549 H3K4me2.tdf
A549 H3K4me3.tdf
A549 H3K27ac.tdf
Refseq Genes
OVOL2
H
ZEB2
A549 H3K4me1.tdf
A549 H3K4me2.tdf
A549 H3K4me3.tdf
A549 H3K27ac.tdf
Refseq Genes
ZEB2
LINC01412
LOC105373656

Appendix Figure S7. Validation of histone marks H3K4me1/2/3 and H3K27ac in epithelial gene expression program by SP.

A-D. Integrative genomic viewer screen shot visualization of H3K4me3 marks in A549 cells for the peak enrichment at *CDH1* (A), *SCUBE1* (B), *ESPN* (C) and *GPR4* (D) locus. ChIP-seq profile for H3K4me3 marks in A549 cells was obtained from GEO (GSE35583).

E-H. Integrative genomic viewer screen shot visualization of H3K4me1, H3K4me2, H3K4me3 and H3K27ac in A549 cells for the peak enrichment at *CDH1* (E), *KRT19* (F), *OVOL2* (G), and *ZEB2* (H) locus. ChIP-seq profile of A549 spheroid culture treated with TGFβ or TNFα was obtained from GEO (GSE42374).
