# Supplementary material for: Propionate reinforces epithelial identity and reduces aggressiveness of lung carcinoma
Source: EMBO Mol Med. 2023 Sep 28;15(12):e17836. doi: 10.15252/emmm.202317836 (PMC10701619; doi:10.15252/emmm.202317836)
Supplement: Supplementary file 2 — Expanded View Figures PDF [file EMMM-15-e17836-s002.pdf]

Expanded View Figures

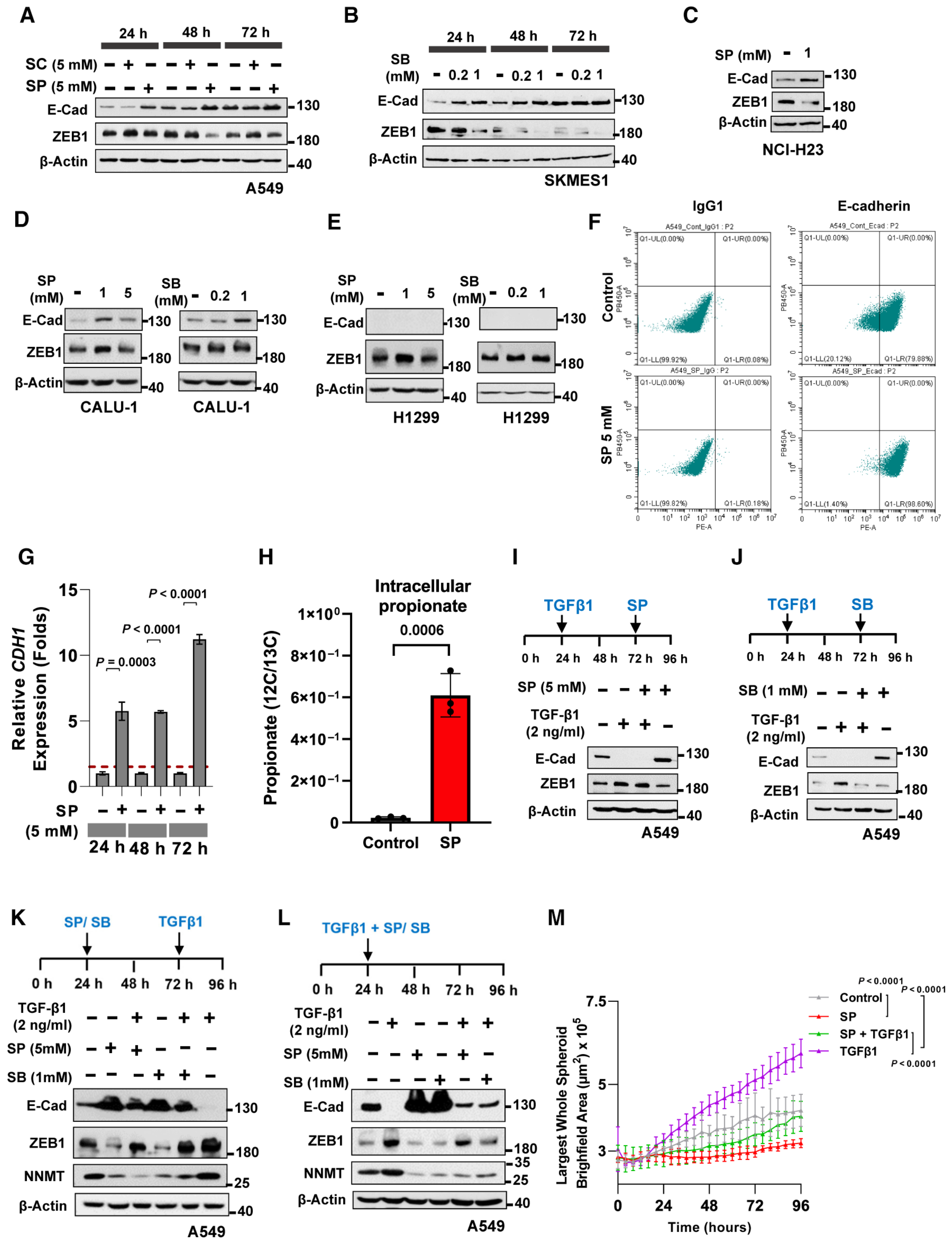

Figure EV1.

◀ **Figure EV1. *In vitro* treatment effect of SCFAs, propionate or butanoate, in EMT marker gene expression.**

A Western blot analysis of E-cadherin and ZEB1 in A549 cell line treated with indicated equimolar concentration of sodium chloride (SC) or sodium propionate (SP) in a time-dependent manner. β-Actin was used as an internal control.

B Western blot analysis of E-cadherin and ZEB1 in SKMES1 cells treated with sodium butanoate (SB) in the indicated dose- and time-dependent manner. β-Actin was used as an internal control. The experiment was performed three independent times.

C Western blot analysis of E-cadherin and ZEB1 in NCI-H23 cell line treated with 1 mM of sodium propionate for 72 h. β-Actin was used as an internal control. The experiment was performed three independent times.

D, E Western blot analysis of E-cadherin and ZEB1 in CALU-1 (D) and in H1299 (E) cell lines treated with sodium propionate (SP) or sodium butanoate (SB) in the indicated dose-dependent manner for 48 h. β-Actin was used as an internal control. The experiment was performed three independent times.

F Flow cytometry analysis of membrane-associated E-cadherin (PE-conjugated E-cadherin) in A549 cells treated with sodium propionate at 5 mM concentration for 48 h. Mean fluorescence intensity of PE-E-cadherin in SP treated cells and controls were 62,858 and 21,999, respectively. PE-conjugated IgG1 was used as a control for flow cytometry. The experiment was performed three independent times.

G Real-time quantitative PCR analysis of epithelial genes *CDH1* in A549 cell line treated with sodium propionate (SP) at 5 mM for 3 days. *GAPDH* was used as an internal control. Red dotted line represents the fold change cut-off at 1.5. Data points (n = 3) are technical replicates represented as mean ± SD of one experiment, and the experiment was performed three independent times. Significance was calculated using unpaired t-test.

H Detection of intracellular levels of propionate in A549 cell line treated with SP (5 mM) for 3 days (n = 3). 13C propionate was used as an internal standard. Data is represented as mean ± SD of technical replicates and significance was calculated using unpaired t-test.

I, J Western blot analysis of E-cadherin and ZEB1 in A549 cells treated with TGF-β1 (2 ng/ml) for 48 h followed by treatment with 5 mM SP (I) or 1 mM SB (J) for 24 h. β-Actin was used as an internal control. The experiments were repeated three independent times.

K Western blot analysis of E-cadherin, ZEB1 and NNMT protein levels in A549 cells pre-treated with sodium propionate (SP, 5 mM) or sodium butanoate (SB, 1 mM) for 48 h followed by TGF-β1 (2 ng/ml) for 24 h. β-Actin was used as an internal control. The experiment was performed three independent times.

L Western blot analysis of E-cadherin, ZEB1 and NNMT protein levels in A549 cells co-treated with sodium propionate (SP, 5 mM) or sodium butanoate (SB, 1 mM) in combination with TGF-β1 (2 ng/ml) for 72 h. β-Actin was used as an internal control. The experiment was performed three independent times.

M Line Plot depicts the quantification of spheroid brightfield area of A549 spheroids treated with sodium propionate (SP, 5 mM) and TGF-β1 (2 ng/ml). Data points (n = 4) are technical replicates represented as mean ± SD and the experiment was performed three independent times. P-value was calculated from two-way ANOVA followed by multiple comparison between conditions using Tukey's multiple comparison test.

 © 2023 The Authors

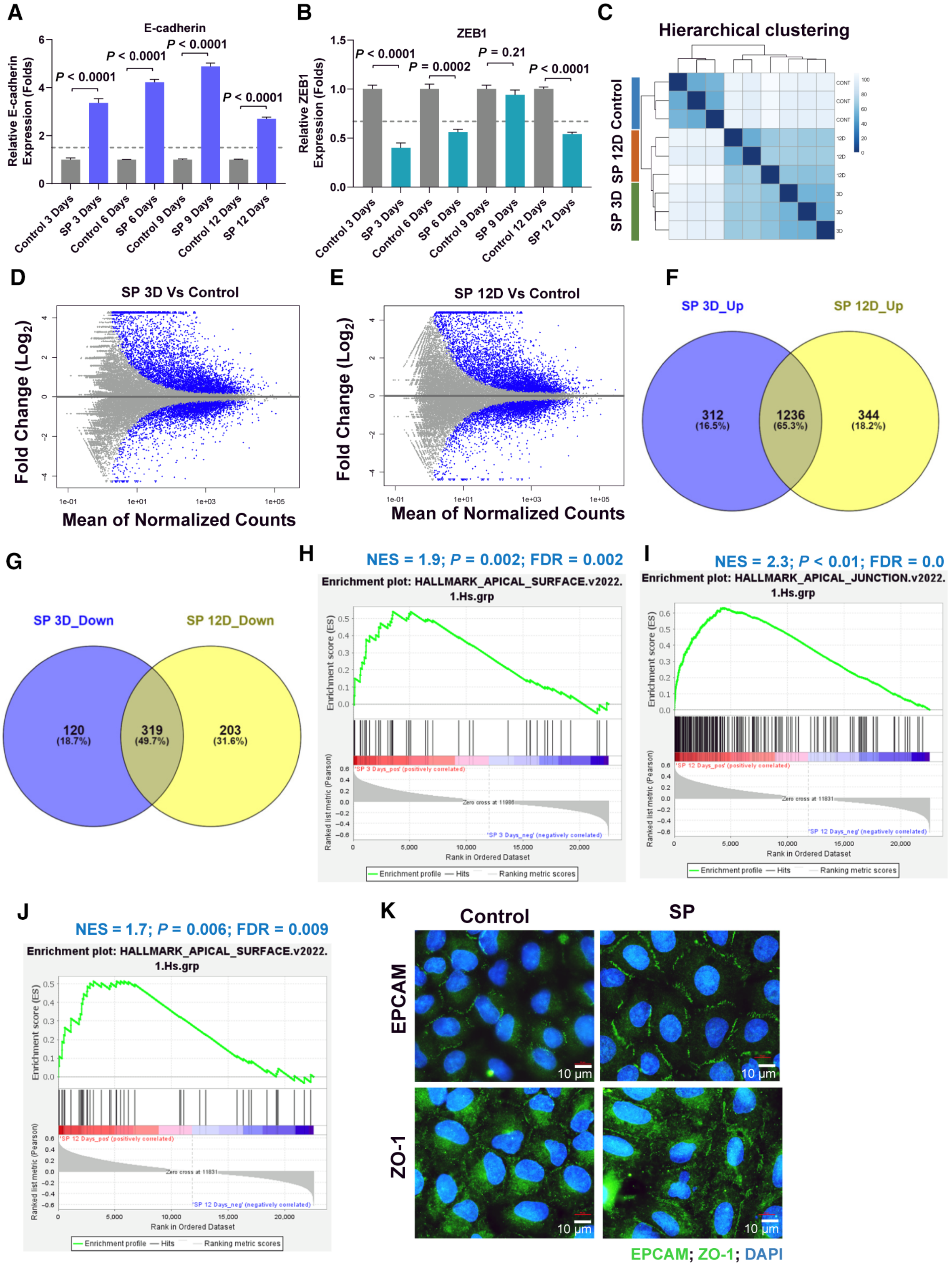
A
E-cadherin
Relative E-cadherin Expression (Folds)
P < 0.0001
P < 0.0001
P < 0.0001
P < 0.0001
P < 0.0001
Control 3 Days
SP 3 Days
Control 6 Days
SP 6 Days
Control 9 Days
SP 9 Days
Control 12 Days
SP 12 Days
B
ZEB1
Relative ZEB1 Expression (Folds)
P < 0.0001
P = 0.0002
P = 0.21
P < 0.0001
C
Hierarchical clustering
SP 3D SP 12D Control
D
SP 3D Vs Control
Fold Change (Log2)
Mean of Normalized Counts
E
SP 12D Vs Control
Fold Change (Log2)
Mean of Normalized Counts
F
SP 3D_Up
SP 12D_Up
312 (16.5%)
1236 (65.3%)
344 (18.2%)
G
SP 3D_Down
SP 12D_Down
120 (18.7%)
319 (49.7%)
203 (31.6%)
H
NES = 1.9; P = 0.002; FDR = 0.002
Enrichment plot: HALLMARK_APICAL_SURFACE.v2022.1.Hs.grp
I
NES = 2.3; P < 0.01; FDR = 0.0
Enrichment plot: HALLMARK_APICAL_JUNCTION.v2022.1.Hs.grp
J
NES = 1.7; P = 0.006; FDR = 0.009
Enrichment plot: HALLMARK_APICAL_SURFACE.v2022.1.Hs.grp
K
Control
SP
EPCAM
ZO-1
10 µm
EPCAM; ZO-1; DAPI

Figure EV2.

Figure EV2. RNA-seq expression profiling of SP treated samples from 3 days and 12 days show similar gene expression pattern with enrichment of epithelial features.

A, B Real-time quantitative PCR analysis of E-cadherin (A) and ZEB1 (B) in A549 cell line treated with sodium propionate at 5 mM in time series for 12 days. Dotted line represents the fold change cut-off at 1.5 (A) or at 0.67 (B). Data points (n = 3) are technical replicates represented as mean ± SD of one experiment and the experiment was performed three independent times. Significance was calculated using un-paired t-test.

C Unsupervised hierarchical clustering analysis of RNA-seq samples of A549 cell line treated with SP for 3 days and 12 days along with control (n = 3 per group).

D, E MA plot of differentially expressed genes identified in SP treated 3 days (D) and 12 days (E) compared to the control (n = 3 per group). Blue dots represent the significantly differentially expressed genes with a fold change of $\log_2$ (1).

F, G Venn diagram representation of overlap analysis between SP 3 days and SP 12 days up-regulated (F) or down-regulated (G) genes.

H–J GSEA of hallmark apical surface gene-set enrichment in SP 3 days samples compared to the control (H), and enrichment of hallmark apical junction gene-set (I) or hallmark apical surface (J) in SP 12 days samples compared to the control (n = 3). Ranking of genes based on Pearson's correlation metric was used for GSEA.

K Magnified images of immunofluorescence staining of EPCAM and ZO-1 in A549 cell line treated with sodium propionate (SP) at 5 mM for 3 days from Fig 5E showing the increased expression of EPCAM and ZO-1 in the membrane region. DAPI was used as a nuclear stain. Image was re-used from Fig 5E. Scale bars: 10 μm.

 © 2023 The Authors

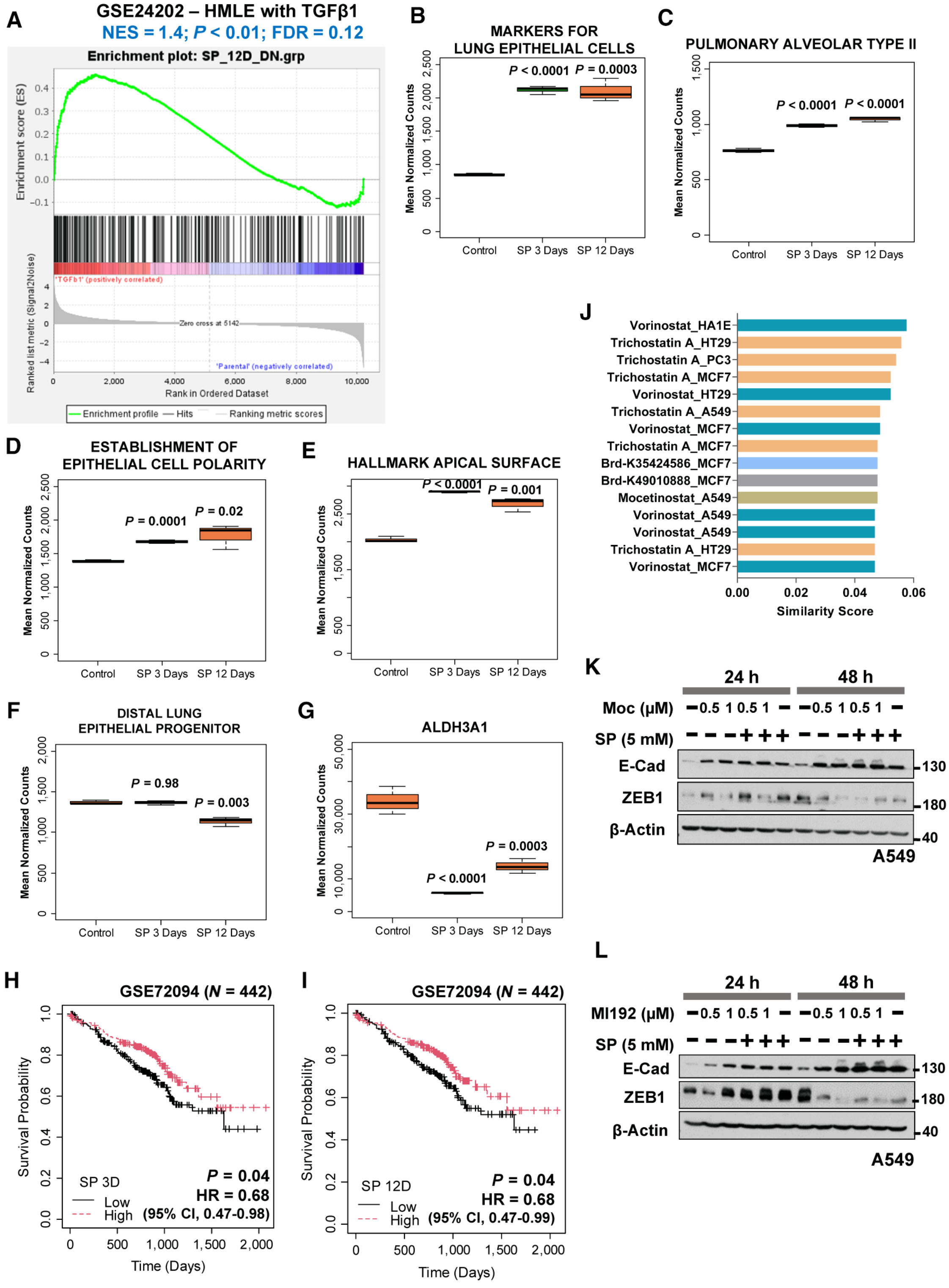
A
GSE24202 – HMLE with TGFβ1
NES = 1.4; P < 0.01; FDR = 0.12
Enrichment plot: SP_12D_DN.grp
B
MARKERS FOR LUNG EPITHELIAL CELLS
P < 0.0001 P = 0.0003
C
PULMONARY ALVEOLAR TYPE II
P < 0.0001 P < 0.0001
D
ESTABLISHMENT OF EPITHELIAL CELL POLARITY
P = 0.0001 P = 0.02
E
HALLMARK APICAL SURFACE
P < 0.0001 P = 0.001
F
DISTAL LUNG EPITHELIAL PROGENITOR
P = 0.98 P = 0.003
G
ALDH3A1
P < 0.0001 P = 0.0003
Mean Normalized Counts
Control SP 3 Days SP 12 Days
H
GSE72094 (N = 442)
SP 3D
P = 0.04
HR = 0.68
(95% CI, 0.47-0.98)
I
GSE72094 (N = 442)
SP 12D
P = 0.04
HR = 0.68
(95% CI, 0.47-0.99)
Survival Probability
Time (Days)
Low
High
J
Vorinostat_HA1E
Trichostatin A_HT29
Trichostatin A_PC3
Trichostatin A_MCF7
Vorinostat_HT29
Trichostatin A_A549
Vorinostat_MCF7
Trichostatin A_MCF7
Brd-K35424586_MCF7
Brd-K49010888_MCF7
Mocetinostat_A549
Vorinostat_A549
Vorinostat_A549
Trichostatin A_HT29
Vorinostat_MCF7
Similarity Score
K
24 h 48 h
Moc (μM)
SP (5 mM)
E-Cad 130
ZEB1 180
β-Actin 40
A549
L
24 h 48 h
MI192 (μM)
SP (5 mM)
E-Cad 130
ZEB1 180
β-Actin 40
A549

Figure EV3.

◀

Figure EV3. Gene expression profiling of SP treated cells showed lung specific epithelial program with epigenetic involvement.

A Gene set enrichment analysis of SP 12 days down-regulated gene-set in TGFβ1-induced HMLE cell line compared to the control obtained from GEO (GSE24202) (n = 3). Ranking of genes with signal2noise metric was used for GSEA.

B–G Box plot visualization of the markers of lung epithelial cell-type gene-set (B), pulmonary alveolar type II cell-type gene-set (C), establishment of epithelial cell polarity gene-set (D), hallmark apical surface gene-set (E), distal lung epithelial progenitor genes (F), and cancer stem cell marker (ALDH3A1 (G)) in control, SP 3 days and SP 12 days RNA-seq samples of A549 cell line. The central band inside the box represents the median value of the data (n = 3) obtained using the lower (bottom) and upper (top) quartile values of the box. The maximum and minimum values of the data are displayed with vertical lines (whiskers) connecting the box. Significance was calculated using un-paired t-test between the SP treated cells and the control. All the lung epithelial cell-type associated gene-sets were collected from PanglaoDB (B–D) and hallmark apical surface gene-set from MSigDB (E).

H, I Overall survival analysis in lung cancer patient samples (GSE72094; N = 442) categorized as low- and high-propionate levels of SP 3 days (H) or SP 12 days (I) gene-set z-score activity based on the median showed good prognosis. HR – Hazard ratio for high propionate group was calculated using Cox proportional hazards model. P-value was calculated using log-rank method.

J Bar plot indicates the top ranked drug gene signatures similar to SP 12 days gene-set based on the similarity score identified from L1000CDS^2 search engine. Identical drugs from different treatment conditions are colored the same.

K, L Western blot analysis of E-cadherin and ZEB1 in A549 cells treated with HDAC inhibitor, mocetinostat (K) or MI192 (L) in the indicated dose- and time-dependent manner in combination with SP 5 mM. β-Actin was used as an internal control.

 © 2023 The Authors

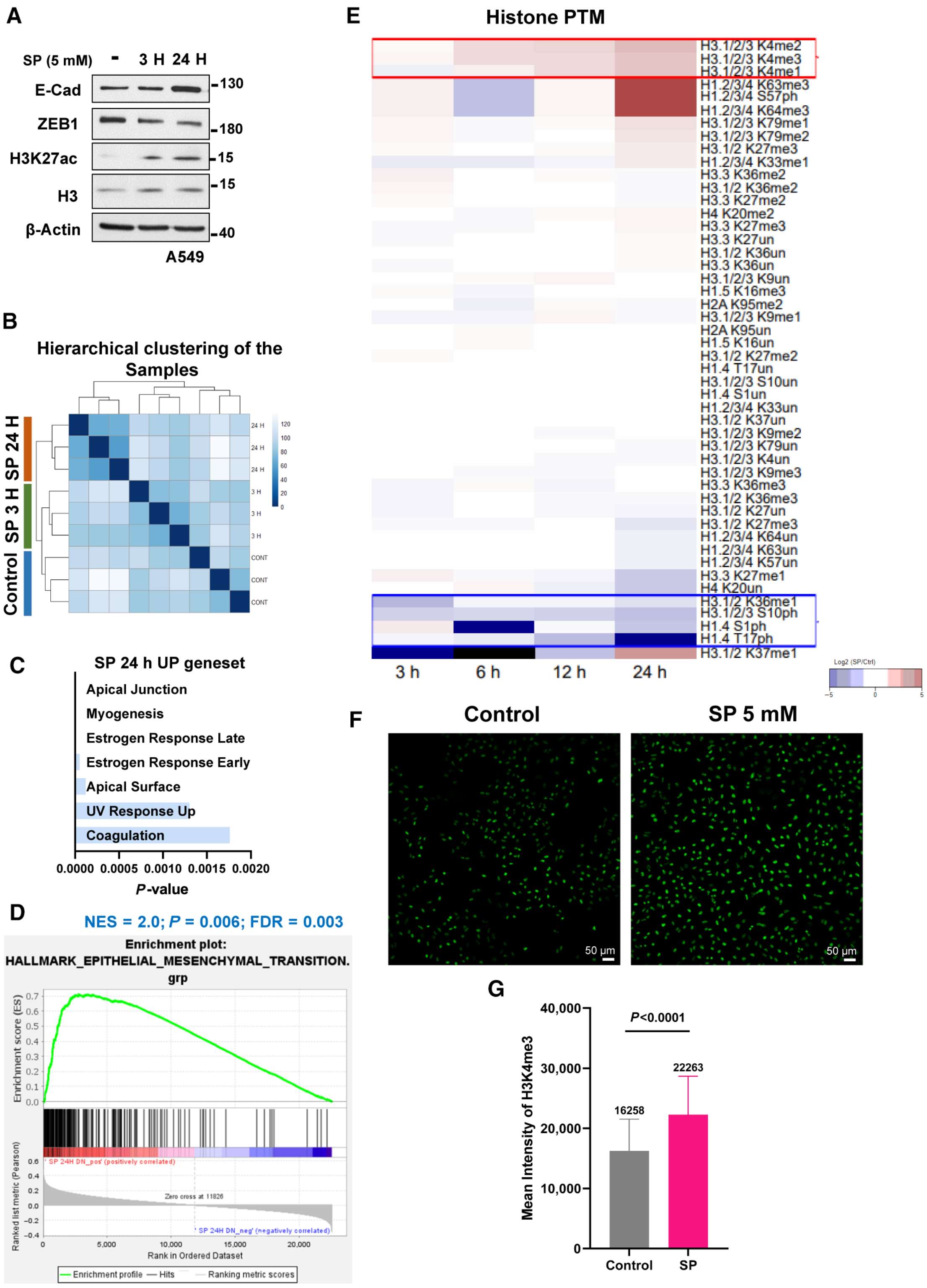
A
SP (5 mM) - 3 H 24 H
E-Cad
ZEB1
H3K27ac
H3
β-Actin
130
180
15
15
40
A549
B
Hierarchical clustering of the Samples
Control SP 3 H SP 24 H
C
SP 24 h UP geneset
Apical Junction
Myogenesis
Estrogen Response Late
Estrogen Response Early
Apical Surface
UV Response Up
Coagulation
0.0000 0.0005 0.0010 0.0015 0.0020
P-value
D
NES = 2.0; P = 0.006; FDR = 0.003
Enrichment plot: HALLMARK_EPITHELIAL_MESENCHYMAL_TRANSITION.grp
Enrichment score (ES)
Ranked list metric (Pearson)
Rank in Ordered Dataset
Enrichment profile — Hits Ranking metric scores
E
Histone PTM
H3.1/2/3 K4me2
H3.1/2/3 K4me3
H3.1/2/3 K4me1
H1.2/3/4 K63me3
H1.2/3/4 S57ph
H1.2/3/4 K64me3
H3.1/2/3 K79me1
H3.1/2/3 K79me2
H3.1/2 K27me3
H1.2/3/4 K33me1
H3.3 K36me2
H3.1/2 K36me2
H3.3 K27me2
H4 K20me2
H3.3 K27me3
H3.3 K27un
H3.1/2 K36un
H3.3 K36un
H3.1/2/3 K9un
H1.5 K16me3
H2A K95me2
H3.1/2/3 K9me1
H2A K95un
H1.5 K16un
H3.1/2 K27me2
H1.4 T17un
H3.1/2/3 S10un
H1.4 S1un
H1.2/3/4 K33un
H3.1/2 K37un
H3.1/2/3 K9me2
H3.1/2/3 K79un
H3.1/2/3 K4un
H3.1/2/3 K9me3
H3.3 K36me3
H3.1/2 K36me3
H3.1/2 K27un
H3.1/2 K27me3
H1.2/3/4 K64un
H1.2/3/4 K63un
H1.2/3/4 K57un
H3.3 K27me1
H4 K20un
H3.1/2 K36me1
H3.1/2/3 S10ph
H1.4 S1ph
H1.4 T17ph
H3.1/2 K37me1
3 h 6 h 12 h 24 h
Log2 (SP/Ctrl)
F
Control
SP 5 mM
50 μm
50 μm
G
Mean Intensity of H3K4me3
40,000
30,000
20,000
10,000
0
P<0.0001
16258
22263
Control SP

Figure EV4.

◀

Figure EV4. Propionate induces transcriptional histone active marks during epithelial gene expression program.

A Western blot analysis of E-cadherin, ZEB1, H3K27ac and H3 in A549 cells treated with sodium propionate (5 mM) for 3 and 24 h. β-Actin was used as an internal control. The experiment was performed three independent times.
B Unsupervised hierarchical clustering analysis of RNA-seq samples of A549 cell line treated with SP at 5 mM for 3 and 24 h along with control (n = 3 per group).
C Enrichr analysis of SP 24 h up-regulated genes identified from RNA-seq. P-value was calculated using Fisher's exact test.
D GSEA analysis of hallmark EMT gene-set enrichment analysis in a lung cancer gene expression profile (GSE72094, N = 442) as a continuous label of SP 24 h down-regulated gene-set z-score activity. Ranking of genes was based on Pearson's correlation metric in GSEA.
E Heatmap representation of histone post-translational modifications identified using mass spectrometry in A549 cells treated with sodium propionate at 5 mM in time series for 24 h (n = 4). Red highlighted box indicates the increased histone modifications in SP treated conditions compared to the control. Blue highlighted box indicates the decreased histone modifications in SP treated conditions compared to the control. Color intensity scale bar represent the $\log_2$ fold difference (FC) of SP with control (blue, FC $<$ 0; white, FC = 0; red, FC $>$ 0).
F Representative images from QIBC analysis of A549 cells treated with SP (5 mM) for 24 h and stained with H3K4me3 antibody. Scale bars: 50 μm.
G Bar plot of mean intensity level of H3K4me3 in A549 cells treated with SP (5 mM) for 24 h. Data points (n = ~7,000) are represented as mean ± SD and significance was calculated from un-paired t-test.

 © 2023 The Authors
