# Supplementary figures and images for: Propionate reinforces epithelial identity and reduces aggressiveness of lung carcinoma
Source: EMBO Mol Med. 2023 Sep 28;15(12):e17836. doi: 10.15252/emmm.202317836 (PMC10701619; doi:10.15252/emmm.202317836)

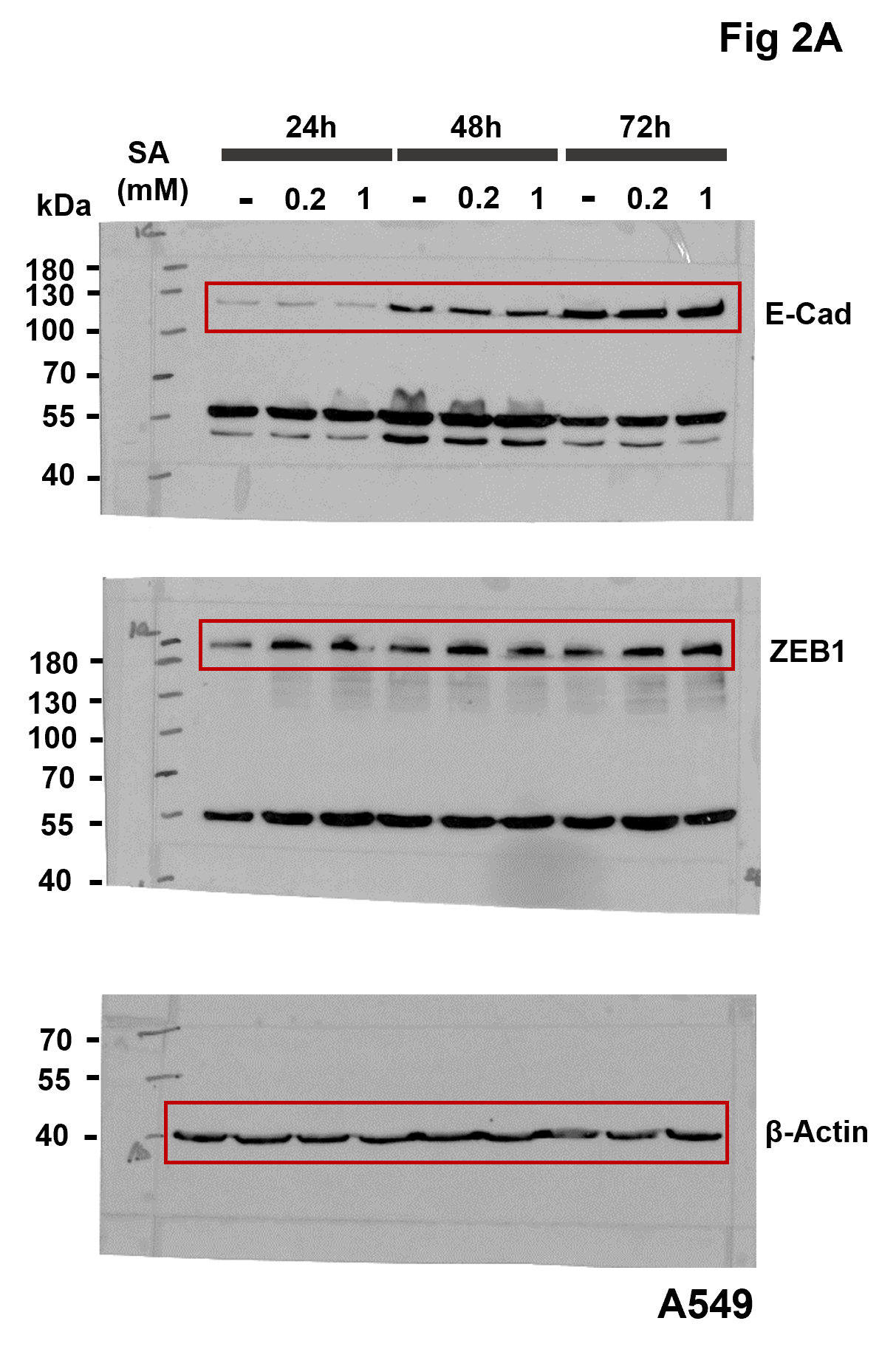

Supplement: Supplementary file 5 — Source Data for Figure 2 [file EMMM-15-e17836-s004.zip › Figure_2/Fig_2A/Fig_2A.tif]

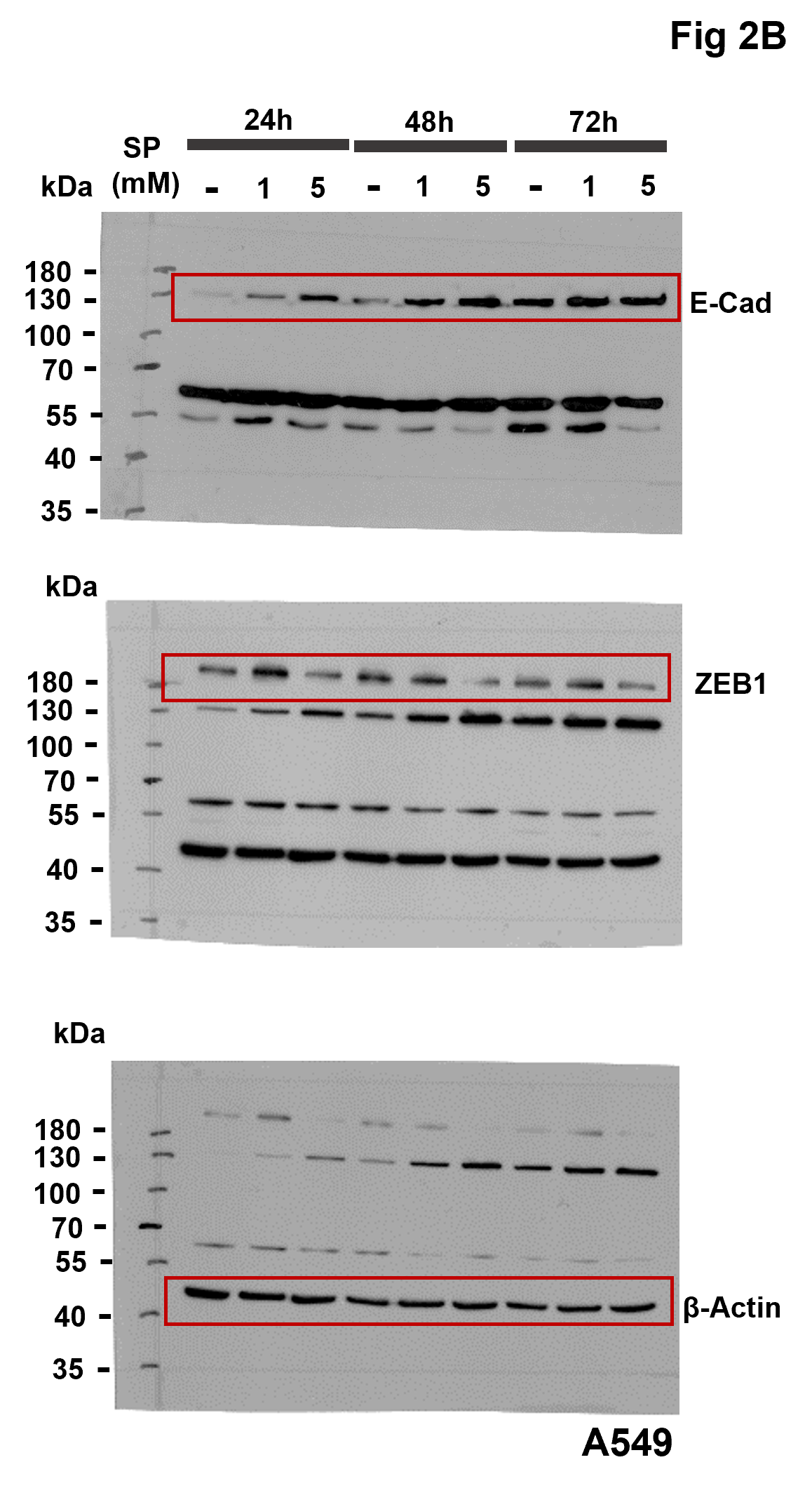

Supplement: Supplementary file 5 — Source Data for Figure 2 [file EMMM-15-e17836-s004.zip › Figure_2/Fig_2B/Fig_2B.tif]

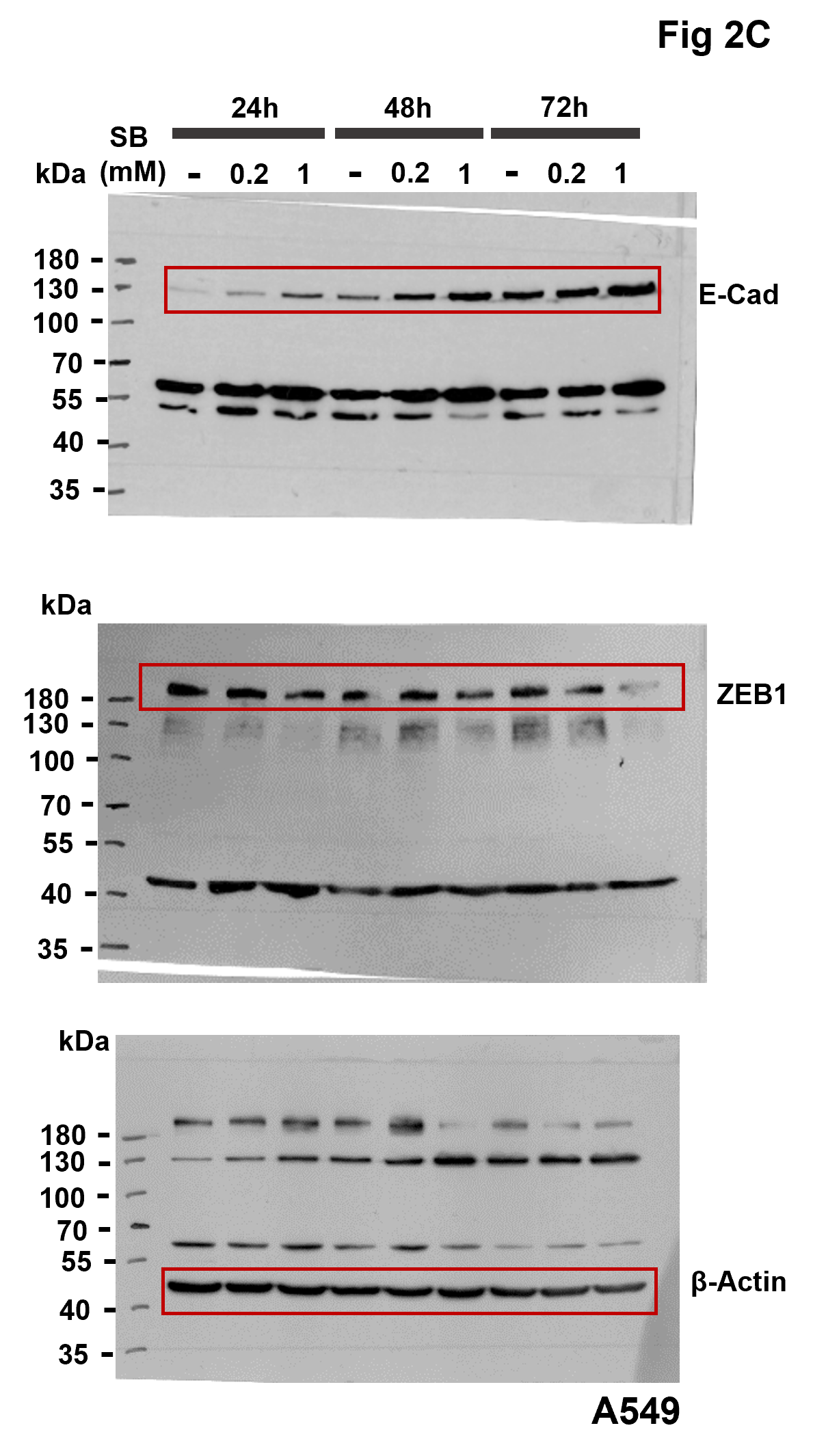

Supplement: Supplementary file 5 — Source Data for Figure 2 [file EMMM-15-e17836-s004.zip › Figure_2/Fig_2C/Fig_2C.tif]

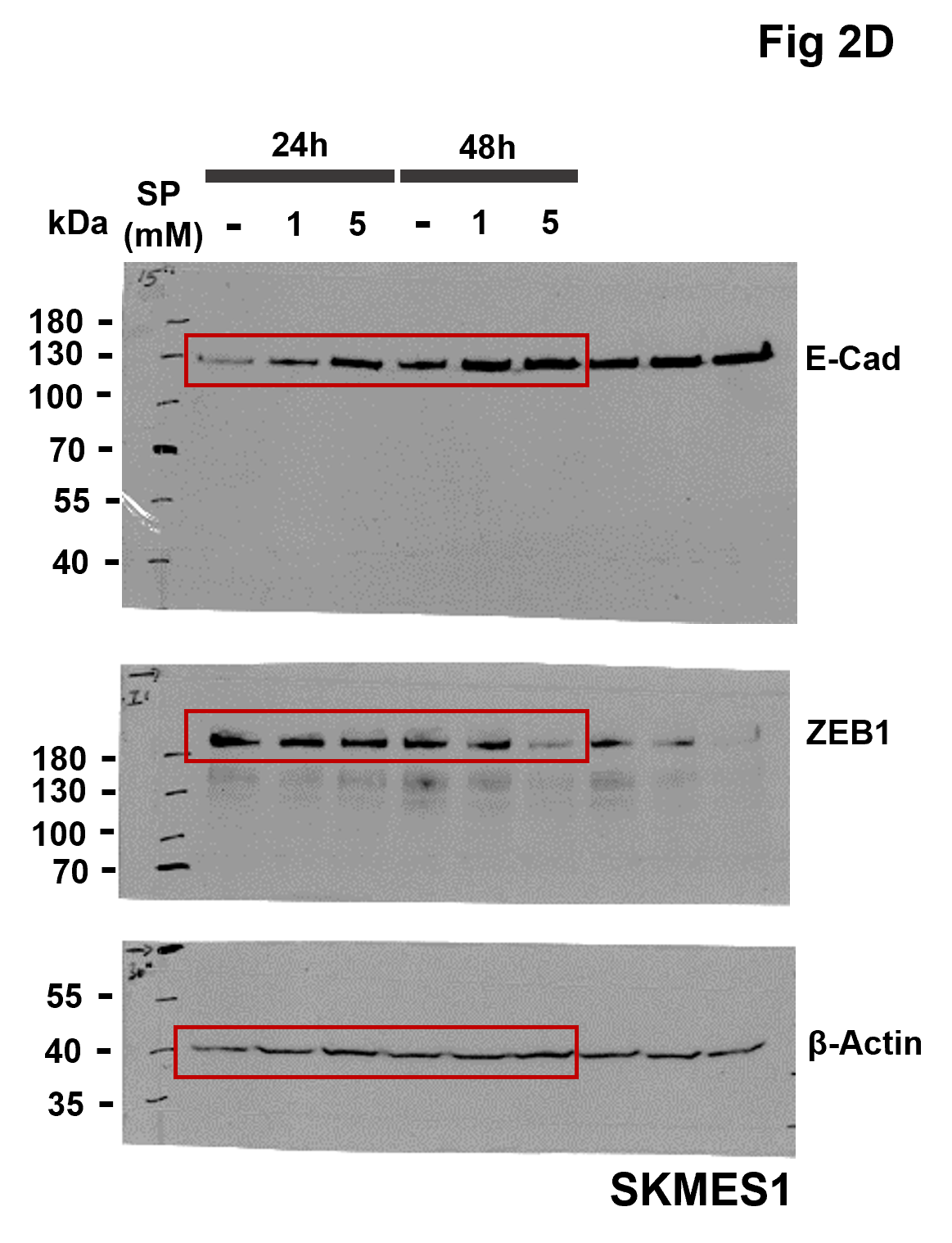

Supplement: Supplementary file 5 — Source Data for Figure 2 [file EMMM-15-e17836-s004.zip › Figure_2/Fig_2D/Fig_2D.tif]

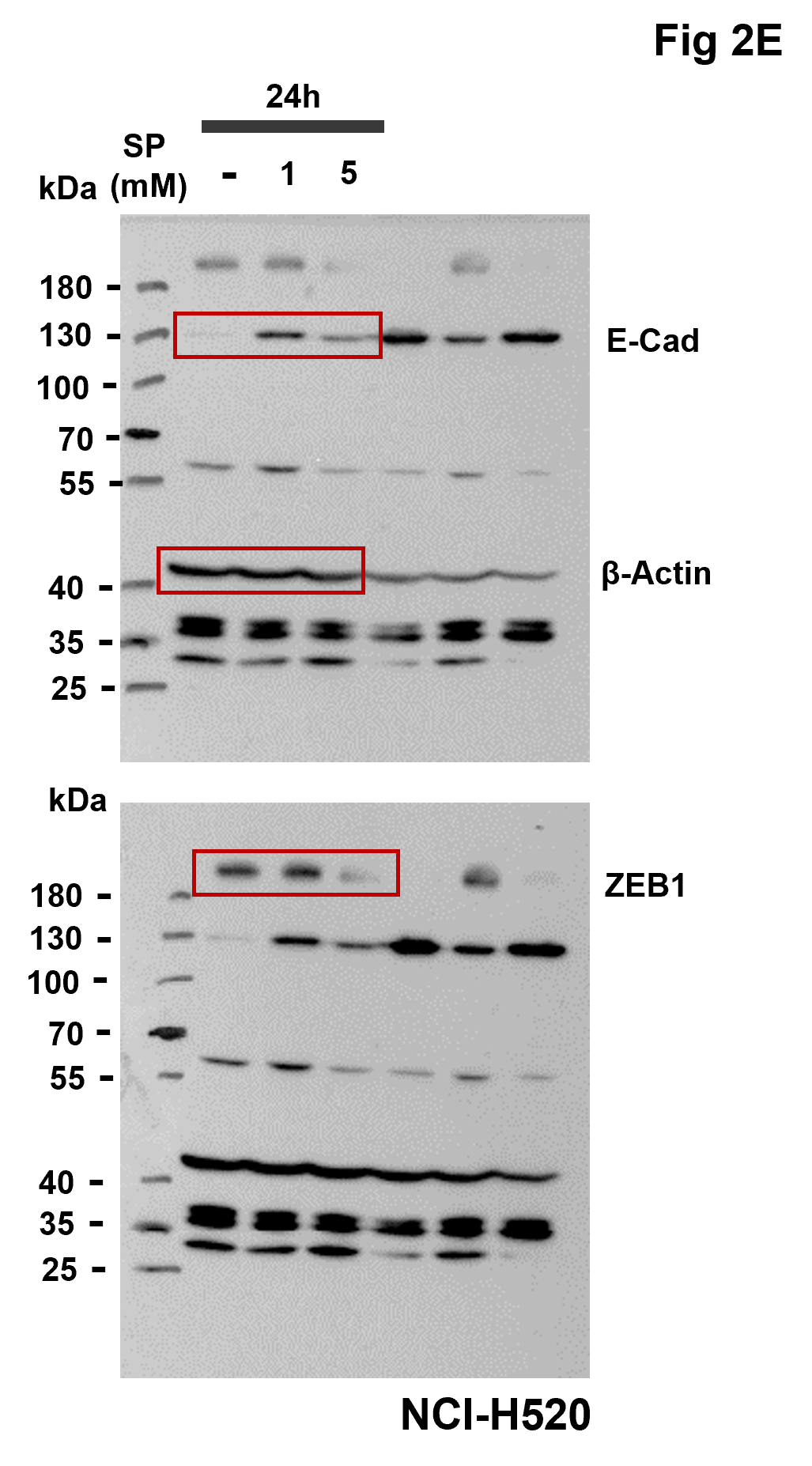

Supplement: Supplementary file 5 — Source Data for Figure 2 [file EMMM-15-e17836-s004.zip › Figure_2/Fig_2E/Fig_2E.tif]

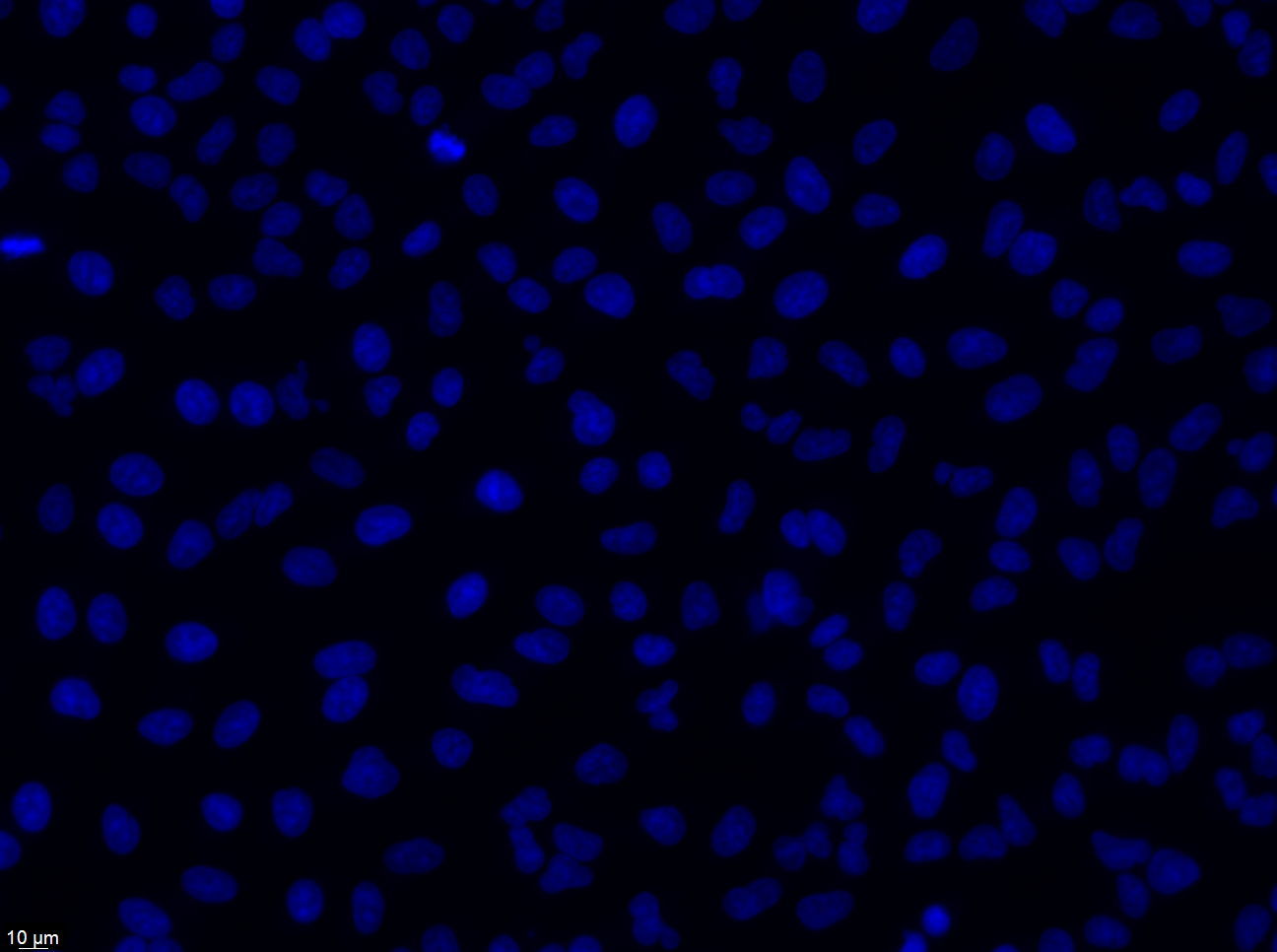

Supplement: Supplementary file 5 — Source Data for Figure 2 [file EMMM-15-e17836-s004.zip › Figure_2/Fig_2F/A549/Control/A549_Control_C_II_3_ch00_DAPI.tif]

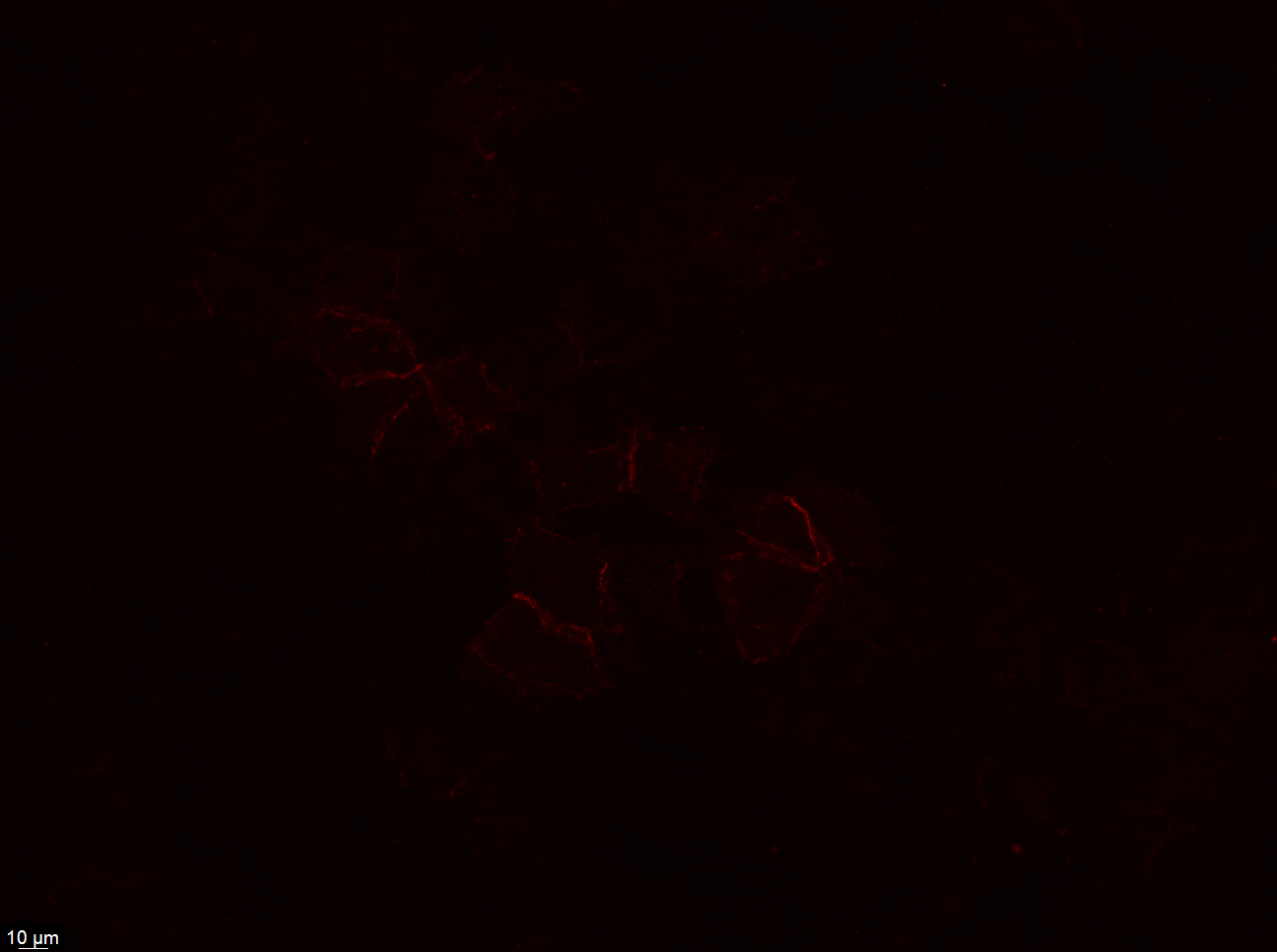

Supplement: Supplementary file 5 — Source Data for Figure 2 [file EMMM-15-e17836-s004.zip › Figure_2/Fig_2F/A549/Control/A549_Control_C_II_3_ch02_ECAD.tif]

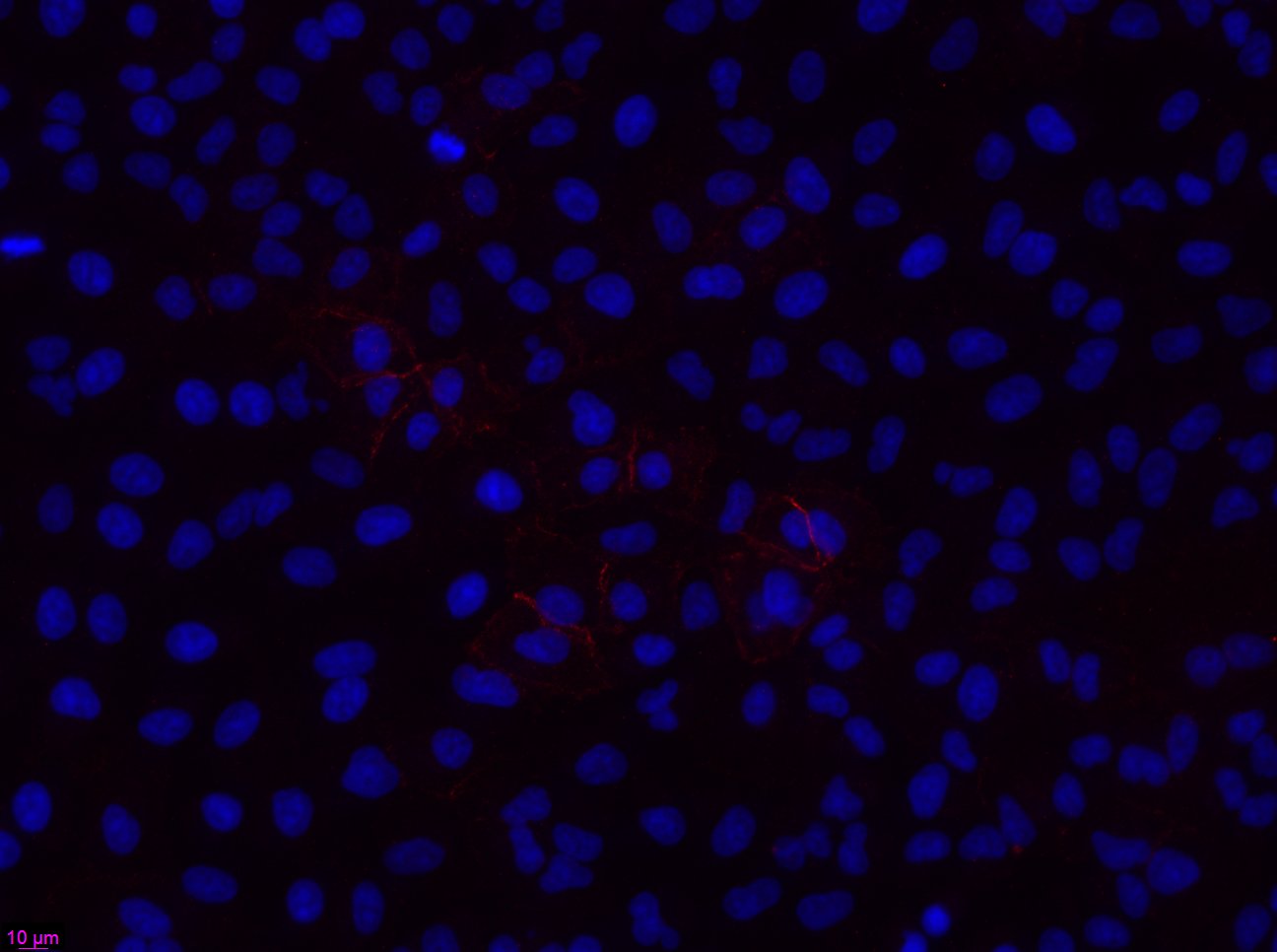

Supplement: Supplementary file 5 — Source Data for Figure 2 [file EMMM-15-e17836-s004.zip › Figure_2/Fig_2F/A549/Control/A549_Control_C_II_3_ECAD.jpg]

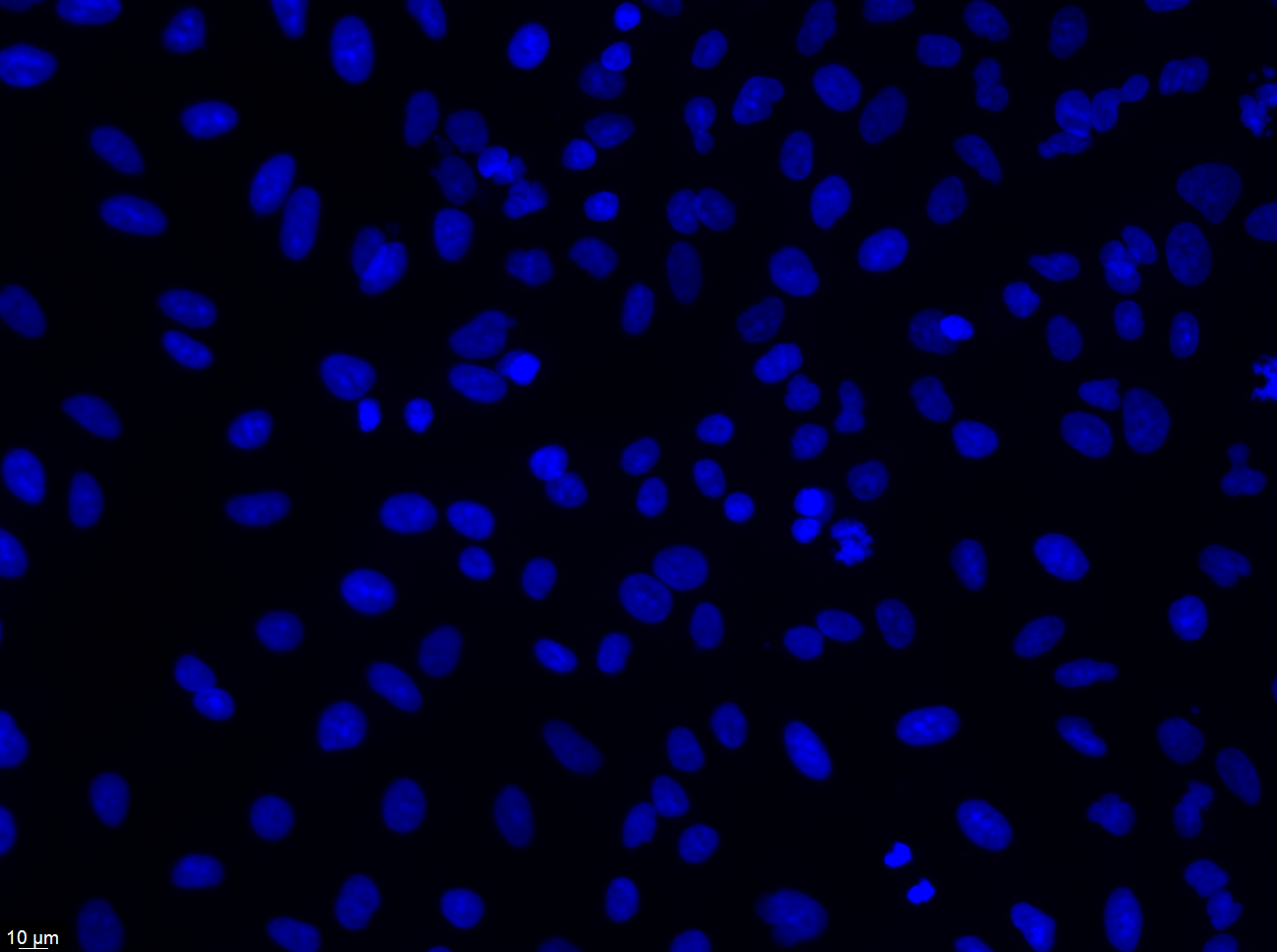

Supplement: Supplementary file 5 — Source Data for Figure 2 [file EMMM-15-e17836-s004.zip › Figure_2/Fig_2F/A549/SP/A549_SP_II_1_ch00_DAPI.tif]

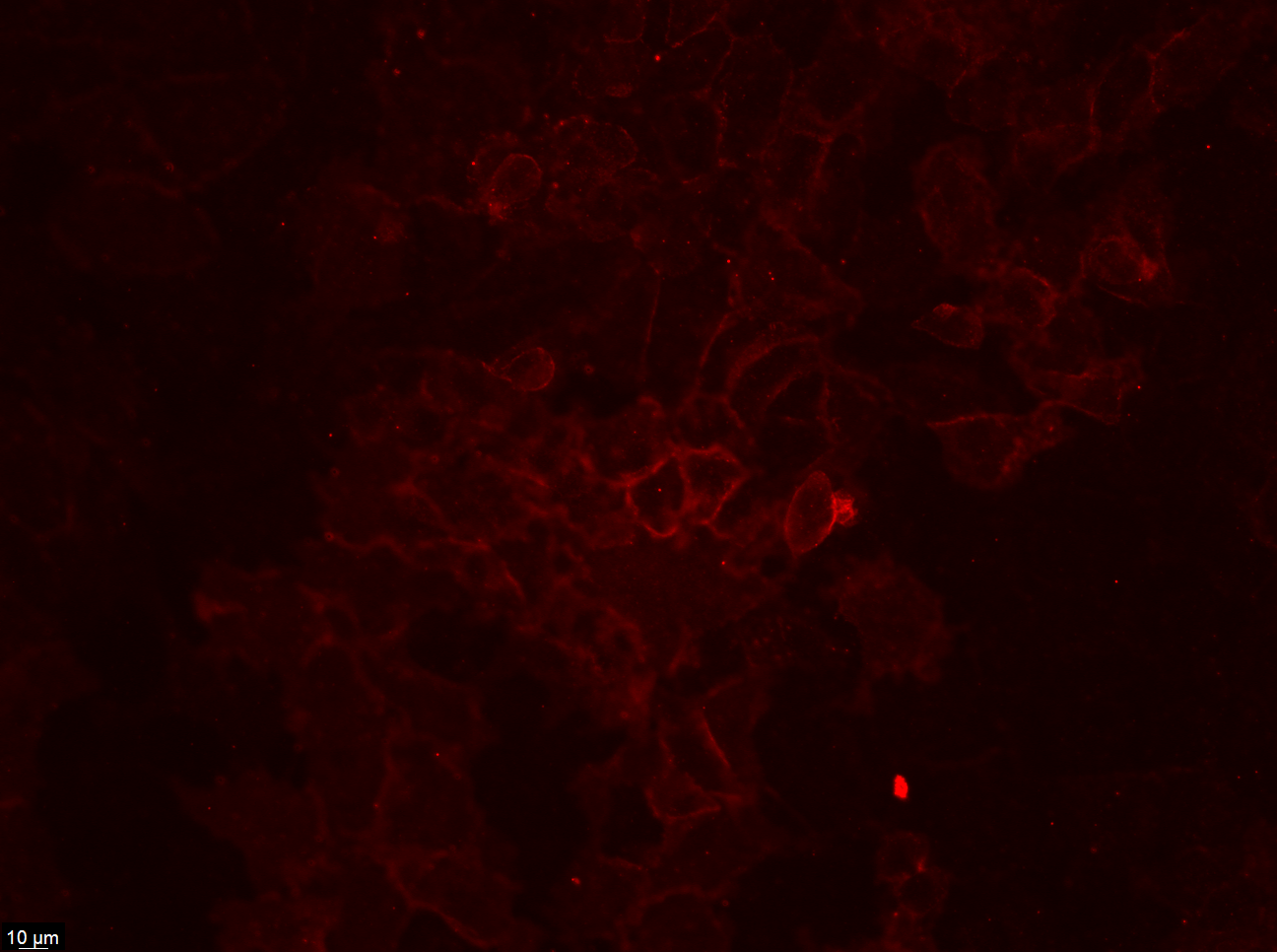

Supplement: Supplementary file 5 — Source Data for Figure 2 [file EMMM-15-e17836-s004.zip › Figure_2/Fig_2F/A549/SP/A549_SP_II_1_ch02_ECAD.tif]

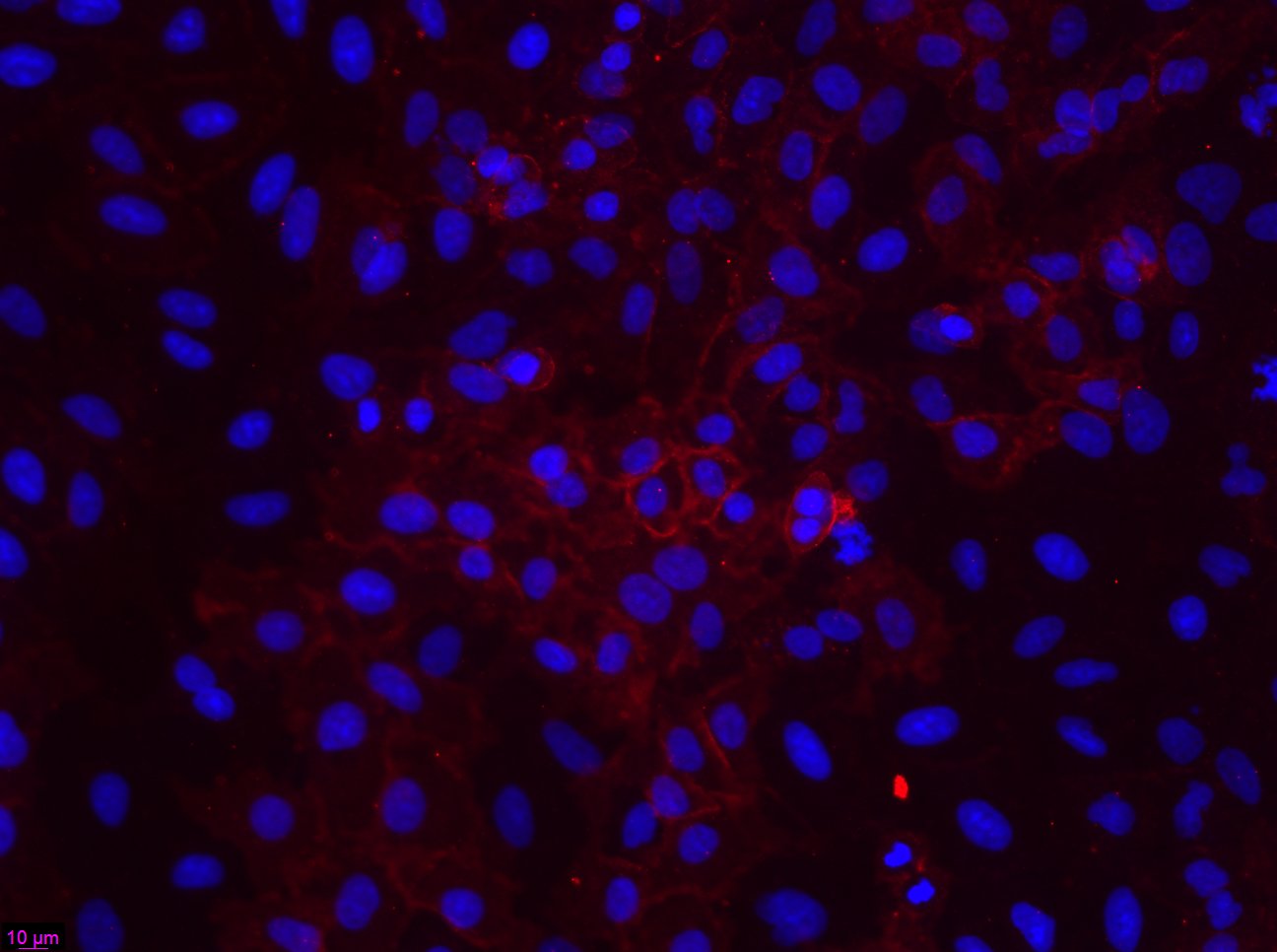

Supplement: Supplementary file 5 — Source Data for Figure 2 [file EMMM-15-e17836-s004.zip › Figure_2/Fig_2F/A549/SP/A549_SP_II_1_ECAD.jpg]

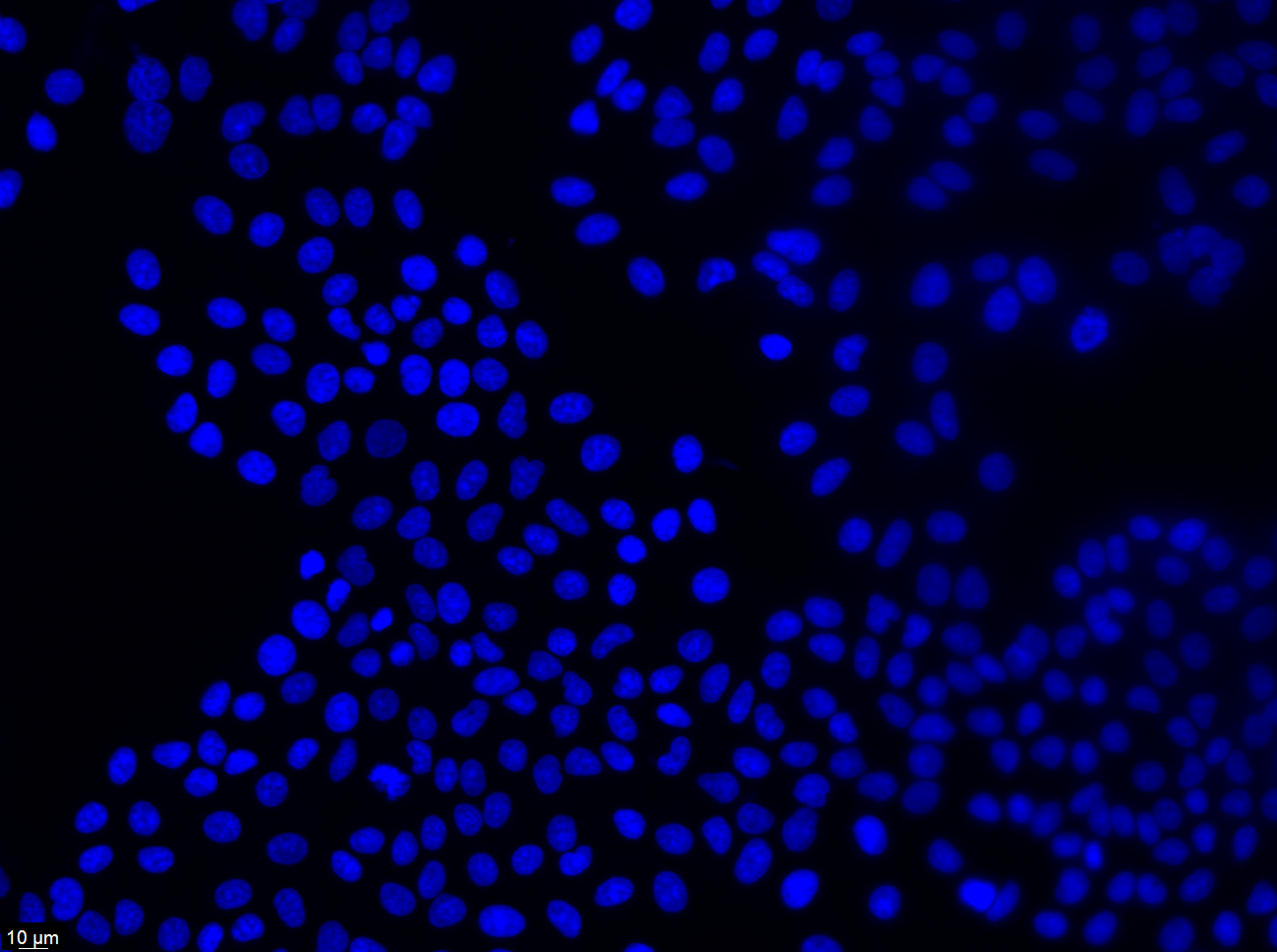

Supplement: Supplementary file 5 — Source Data for Figure 2 [file EMMM-15-e17836-s004.zip › Figure_2/Fig_2F/SKMES1/Control/SKMES1_Control_C_3_ch00_DAPI.tif]

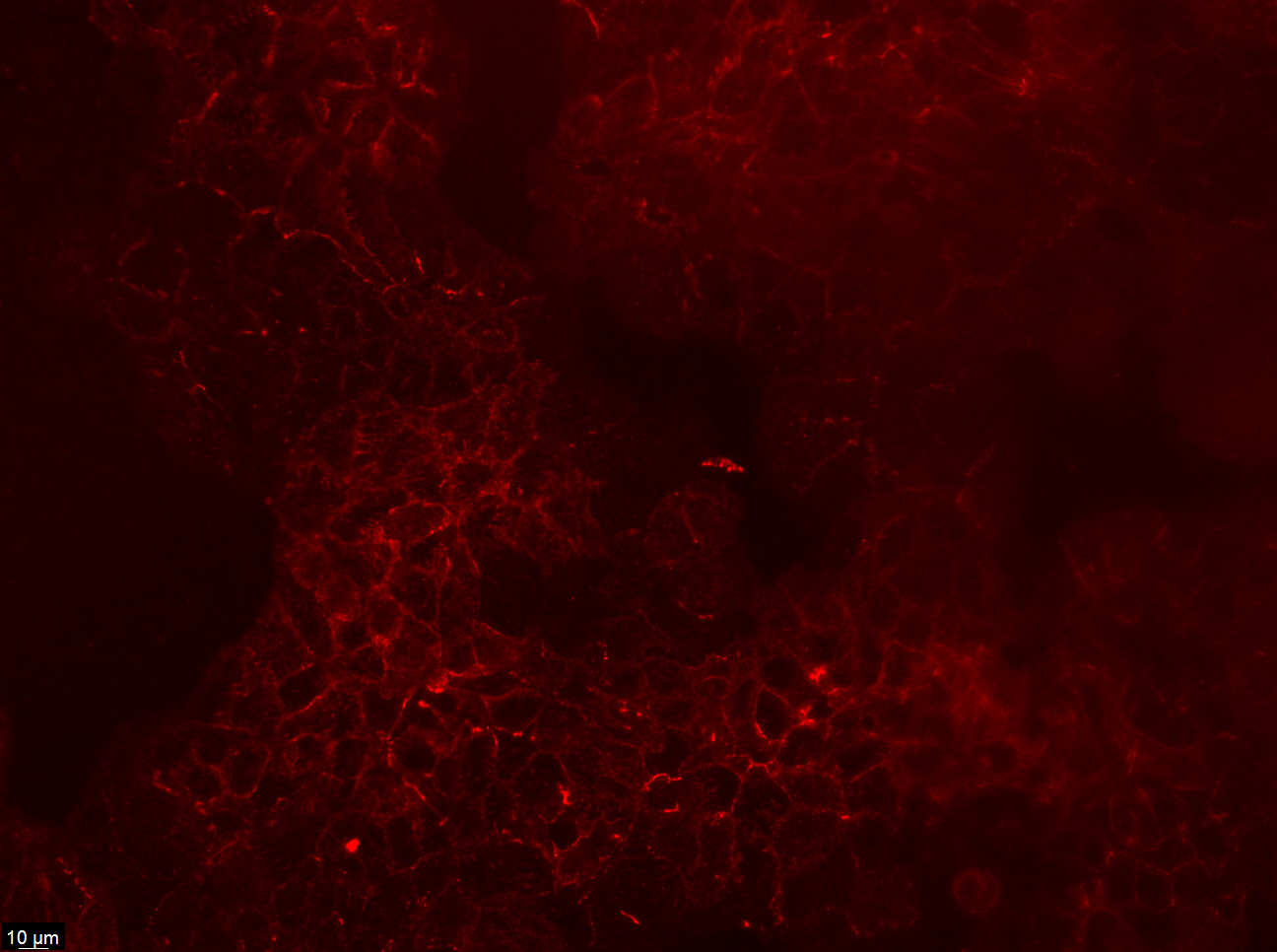

Supplement: Supplementary file 5 — Source Data for Figure 2 [file EMMM-15-e17836-s004.zip › Figure_2/Fig_2F/SKMES1/Control/SKMES1_Control_C_3_ch02_ECAD.tif]

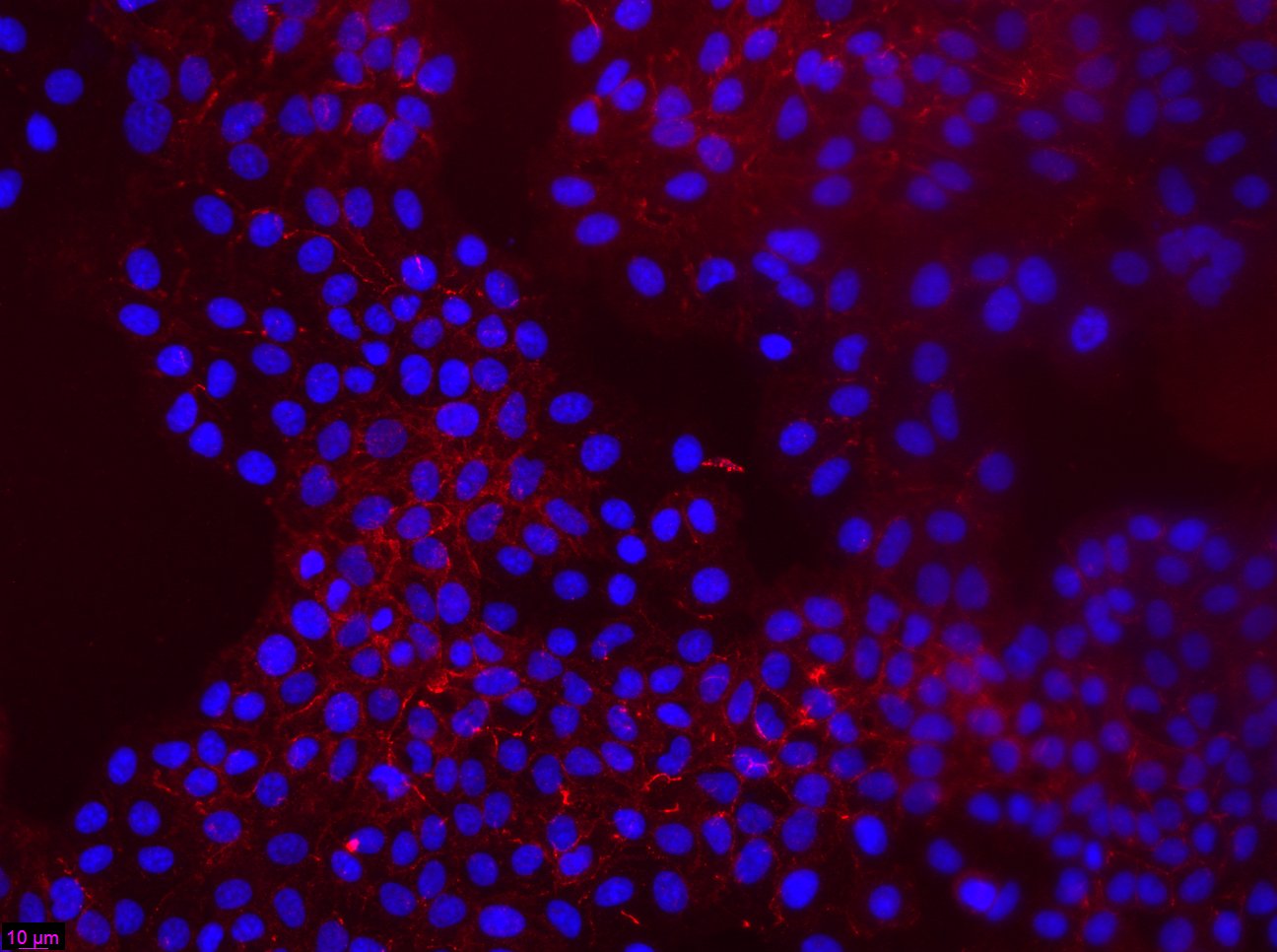

Supplement: Supplementary file 5 — Source Data for Figure 2 [file EMMM-15-e17836-s004.zip › Figure_2/Fig_2F/SKMES1/Control/SKMES1_Control_C_3_ECAD.jpg]

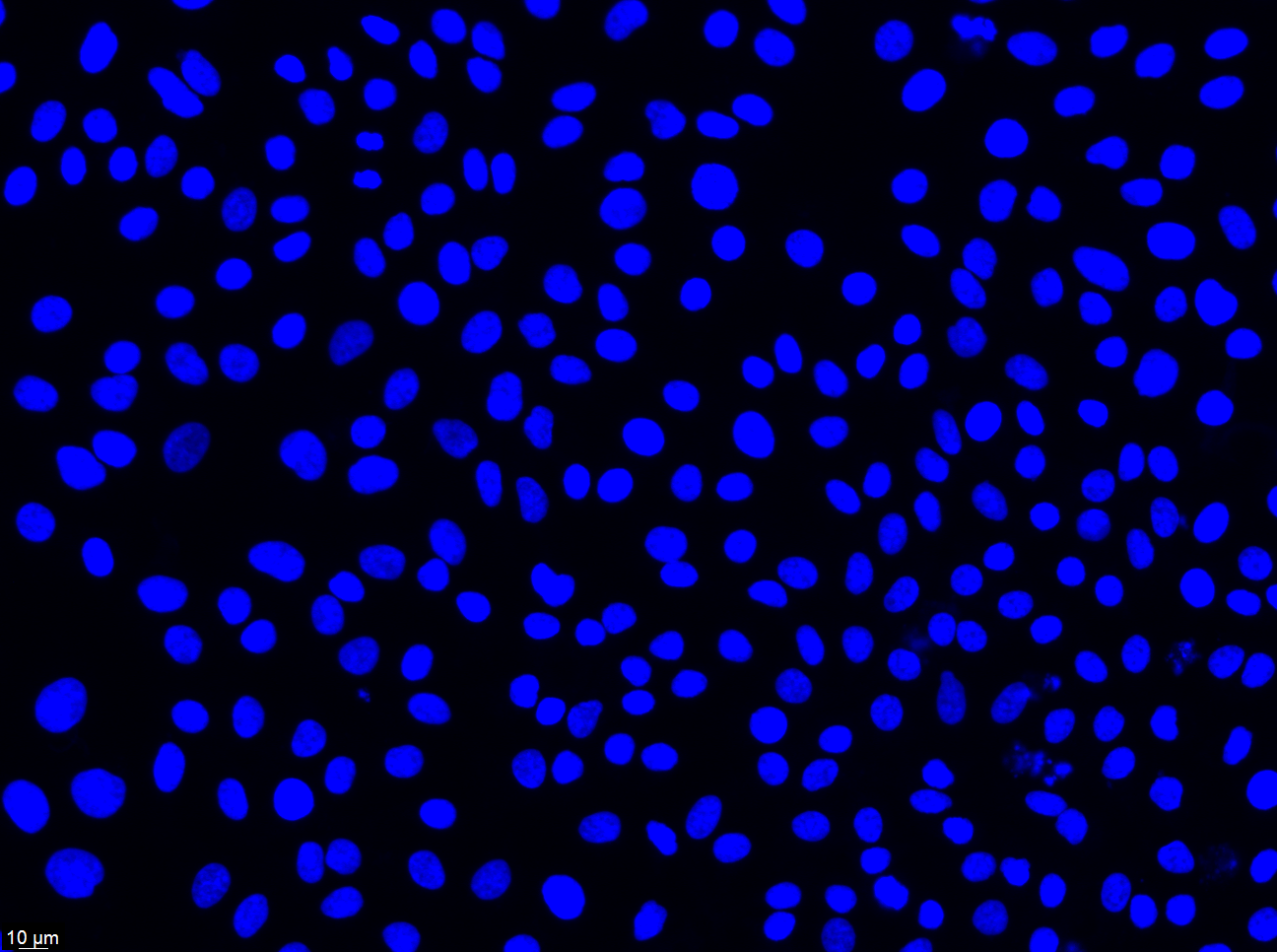

Supplement: Supplementary file 5 — Source Data for Figure 2 [file EMMM-15-e17836-s004.zip › Figure_2/Fig_2F/SKMES1/SP/SKMES1_SP_3_ch00_DAPI.tif]

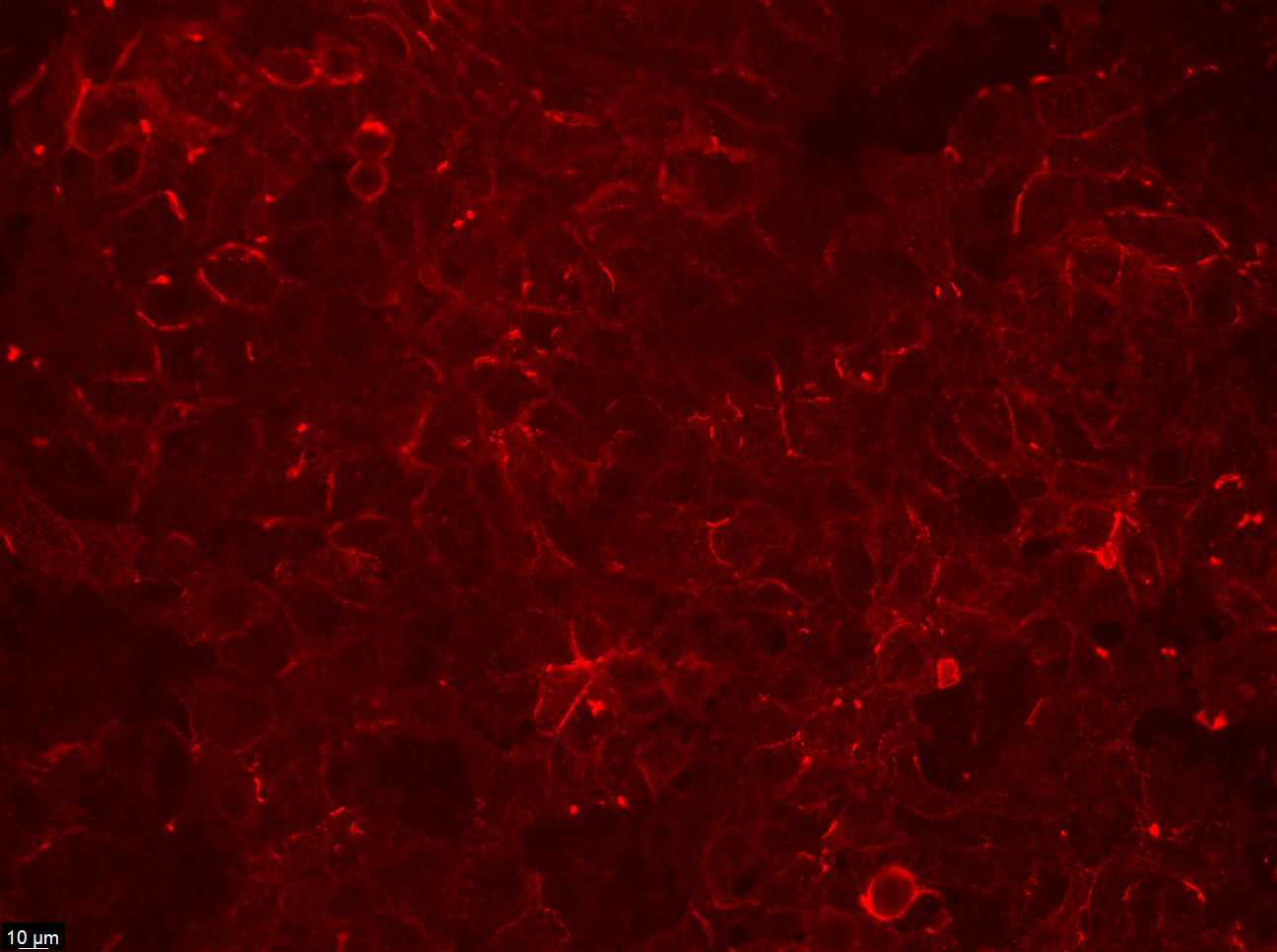

Supplement: Supplementary file 5 — Source Data for Figure 2 [file EMMM-15-e17836-s004.zip › Figure_2/Fig_2F/SKMES1/SP/SKMES1_SP_3_ch02_ECAD.tif]

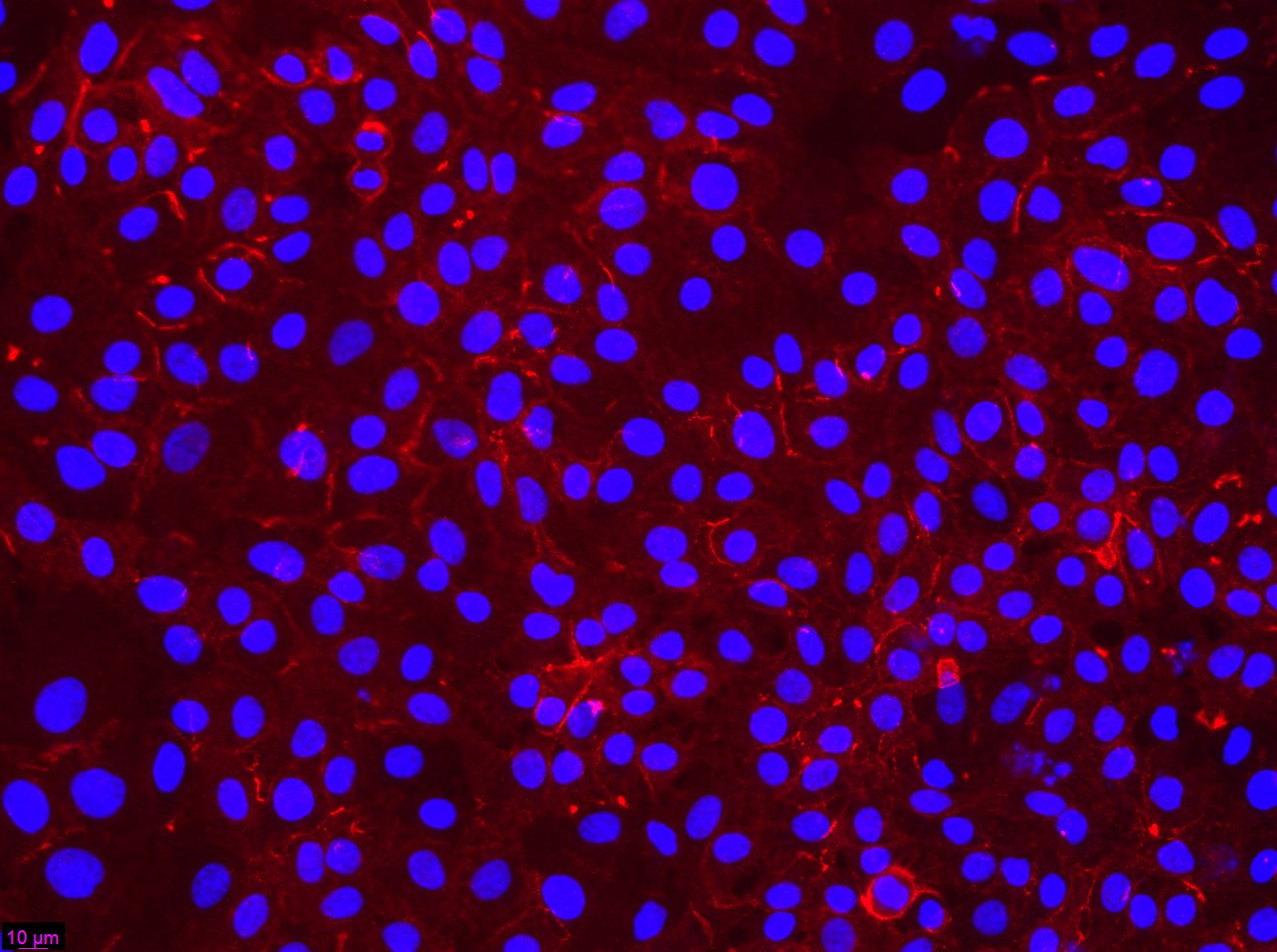

Supplement: Supplementary file 5 — Source Data for Figure 2 [file EMMM-15-e17836-s004.zip › Figure_2/Fig_2F/SKMES1/SP/SKMES1_SP_SP_3_ECAD.jpg]

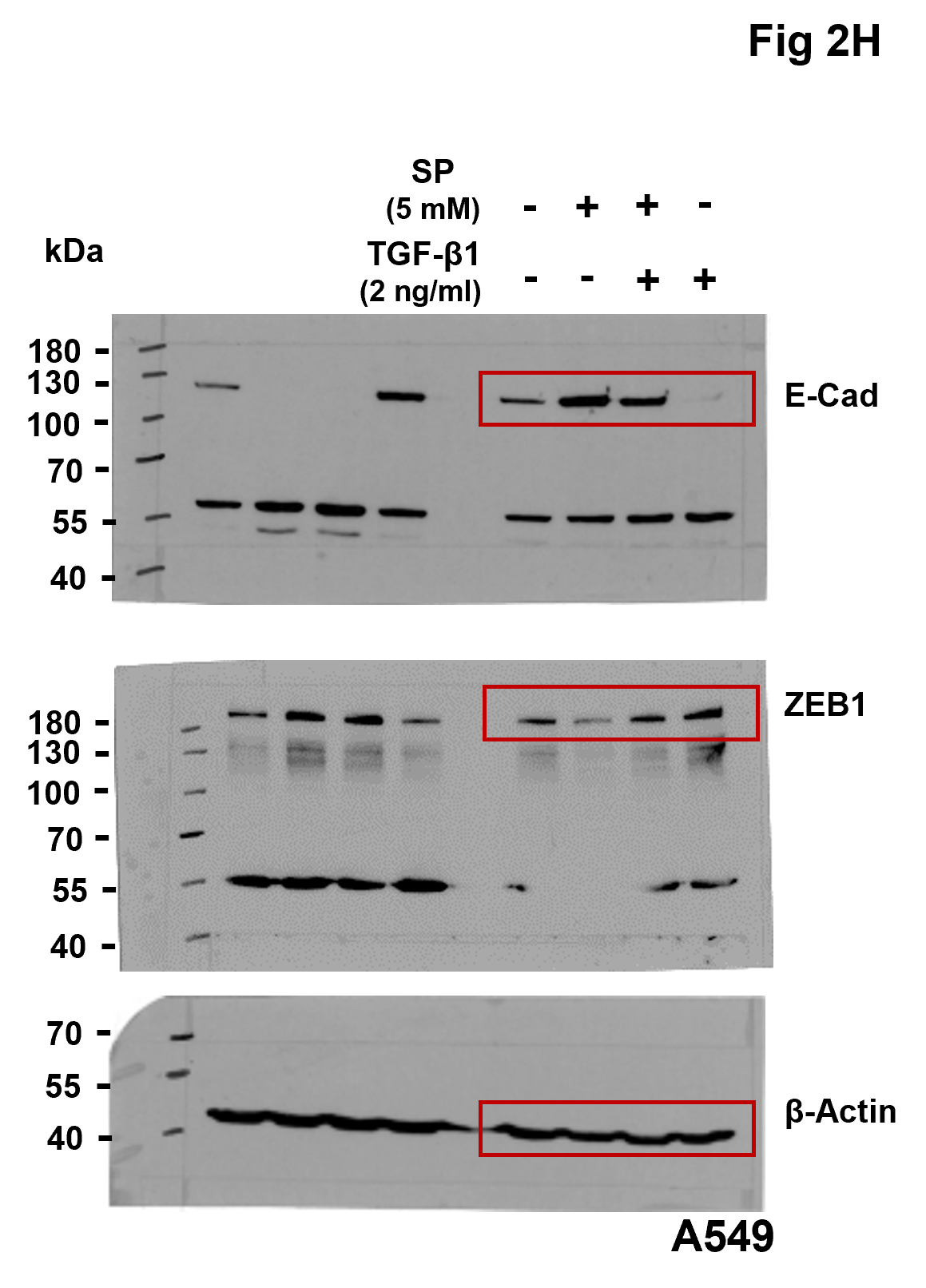

Supplement: Supplementary file 5 — Source Data for Figure 2 [file EMMM-15-e17836-s004.zip › Figure_2/Fig_2H/Fig_2H.tif]

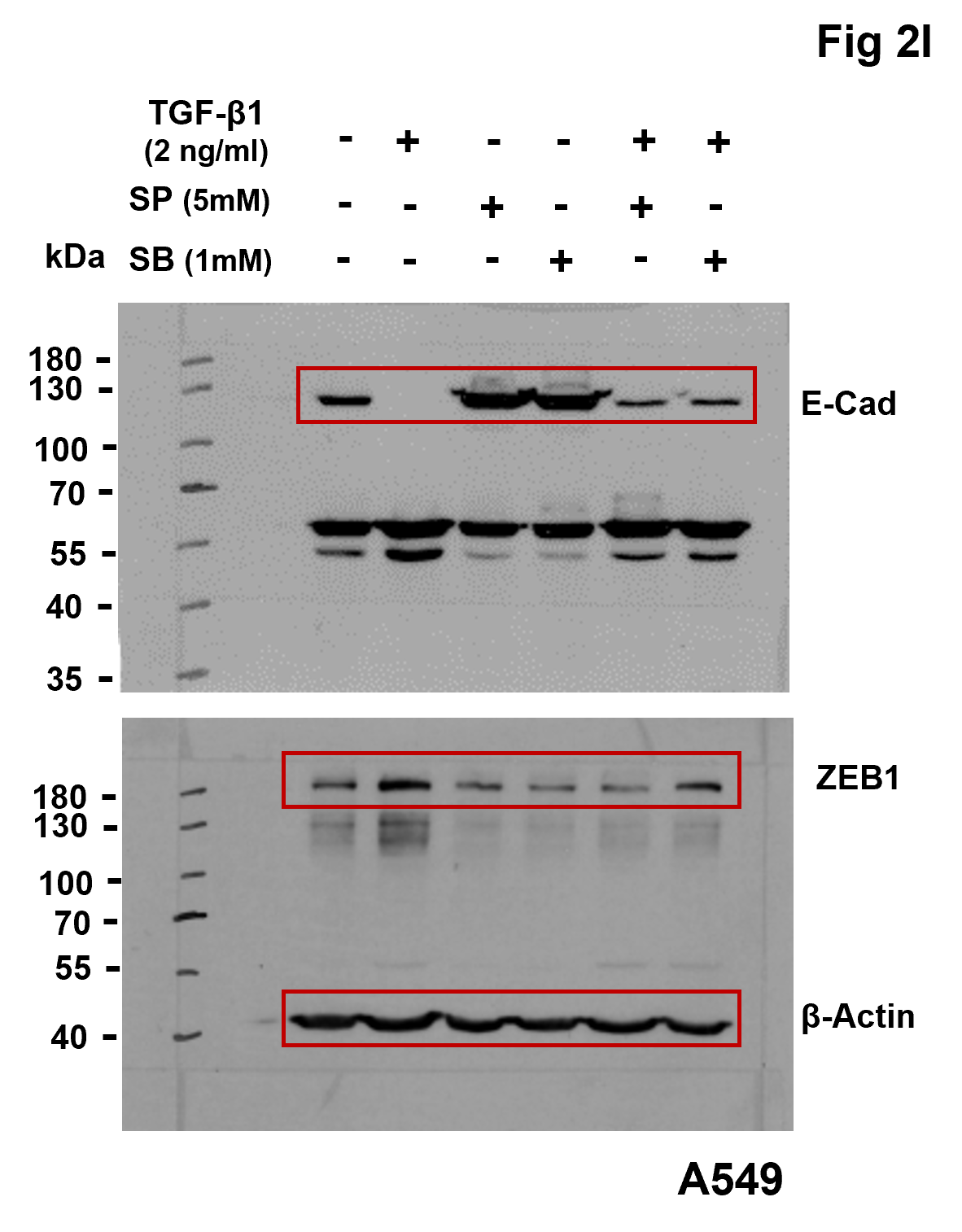

Supplement: Supplementary file 5 — Source Data for Figure 2 [file EMMM-15-e17836-s004.zip › Figure_2/Fig_2I/Fig_2I.tif]

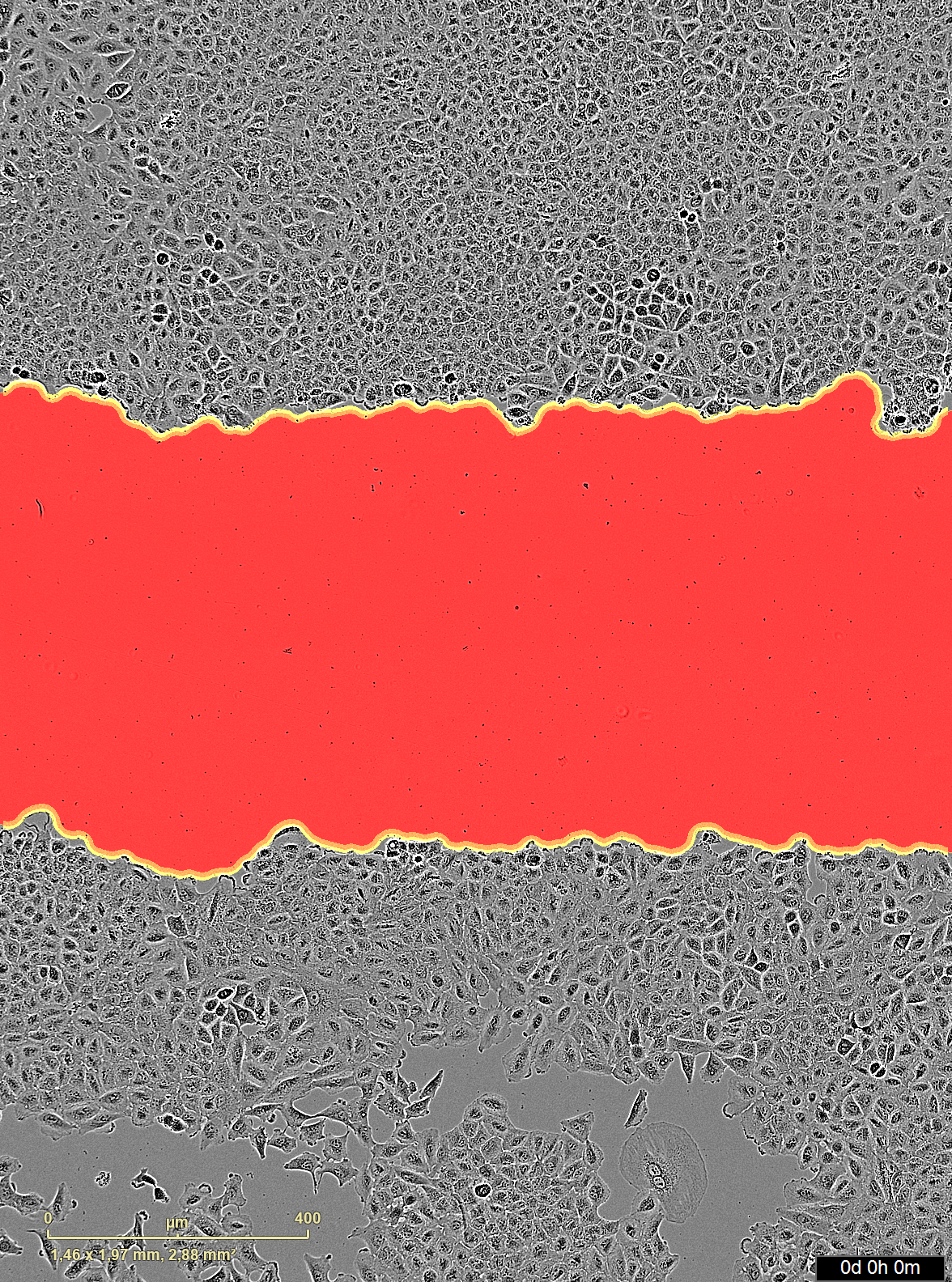

Supplement: Supplementary file 5 — Source Data for Figure 2 [file EMMM-15-e17836-s004.zip › Figure_2/Fig_2J/Images/A549_Control_0h.tif]

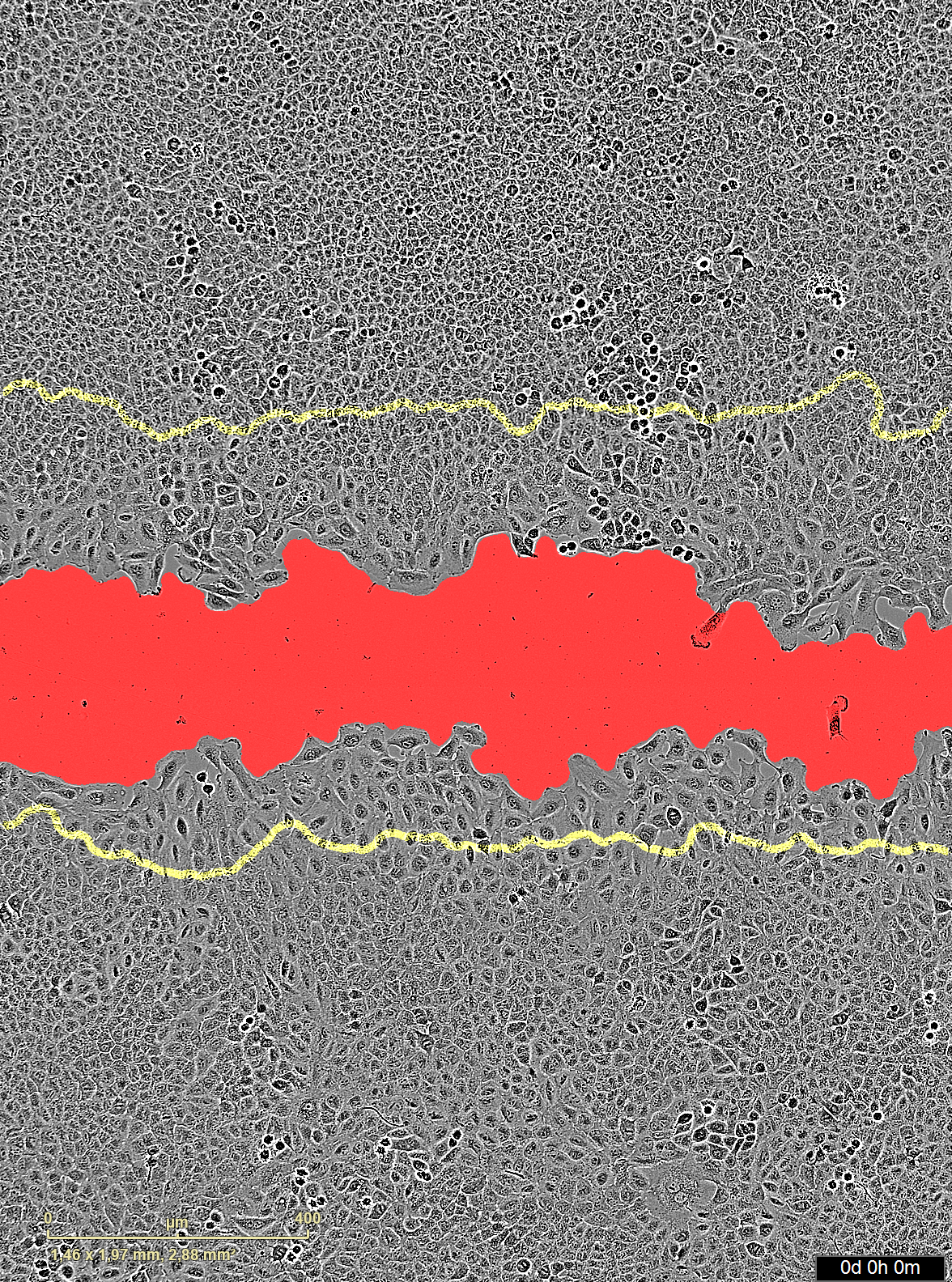

Supplement: Supplementary file 5 — Source Data for Figure 2 [file EMMM-15-e17836-s004.zip › Figure_2/Fig_2J/Images/A549_Control_24h.tif]

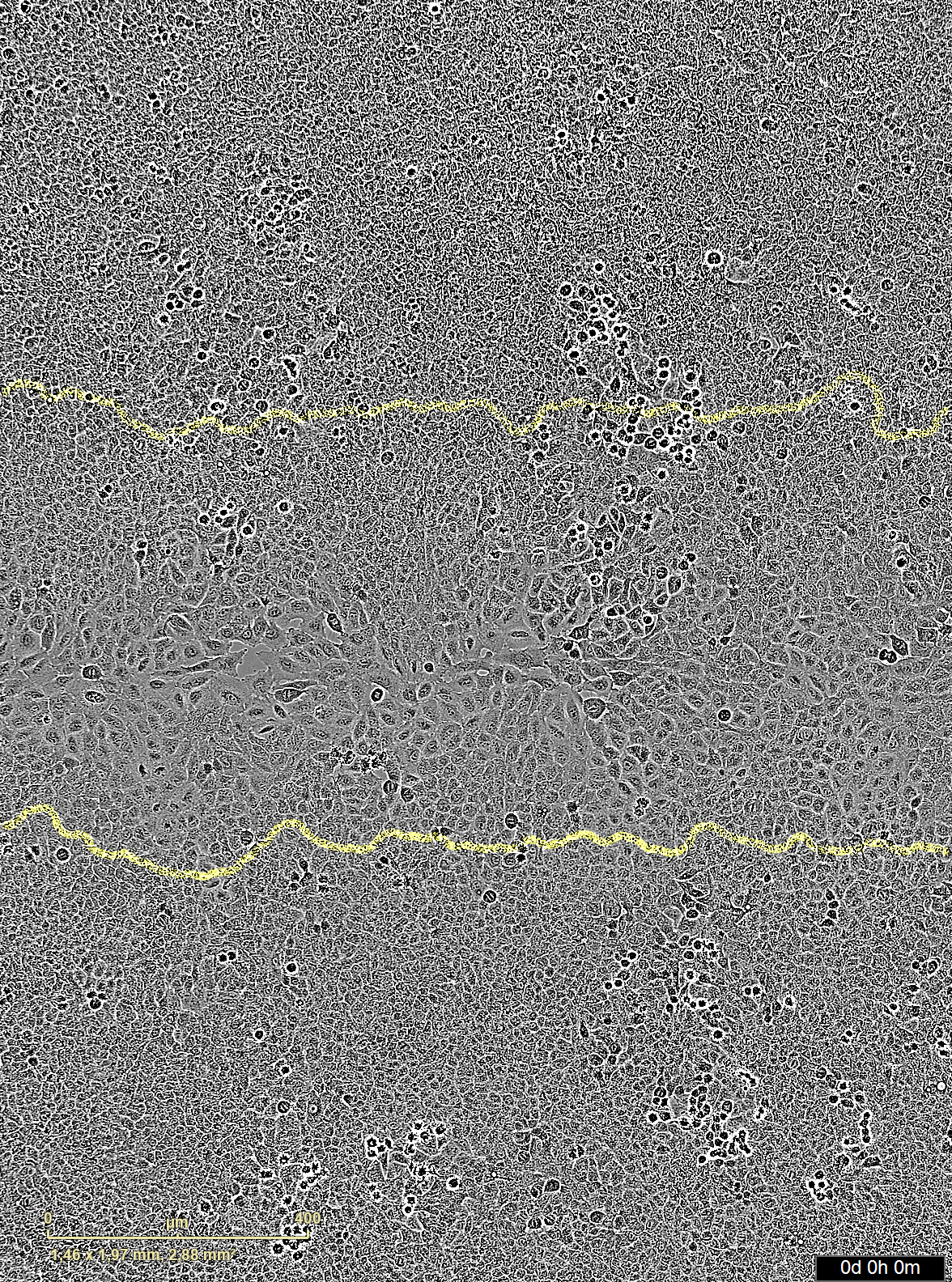

Supplement: Supplementary file 5 — Source Data for Figure 2 [file EMMM-15-e17836-s004.zip › Figure_2/Fig_2J/Images/A549_Control_48h.tif]

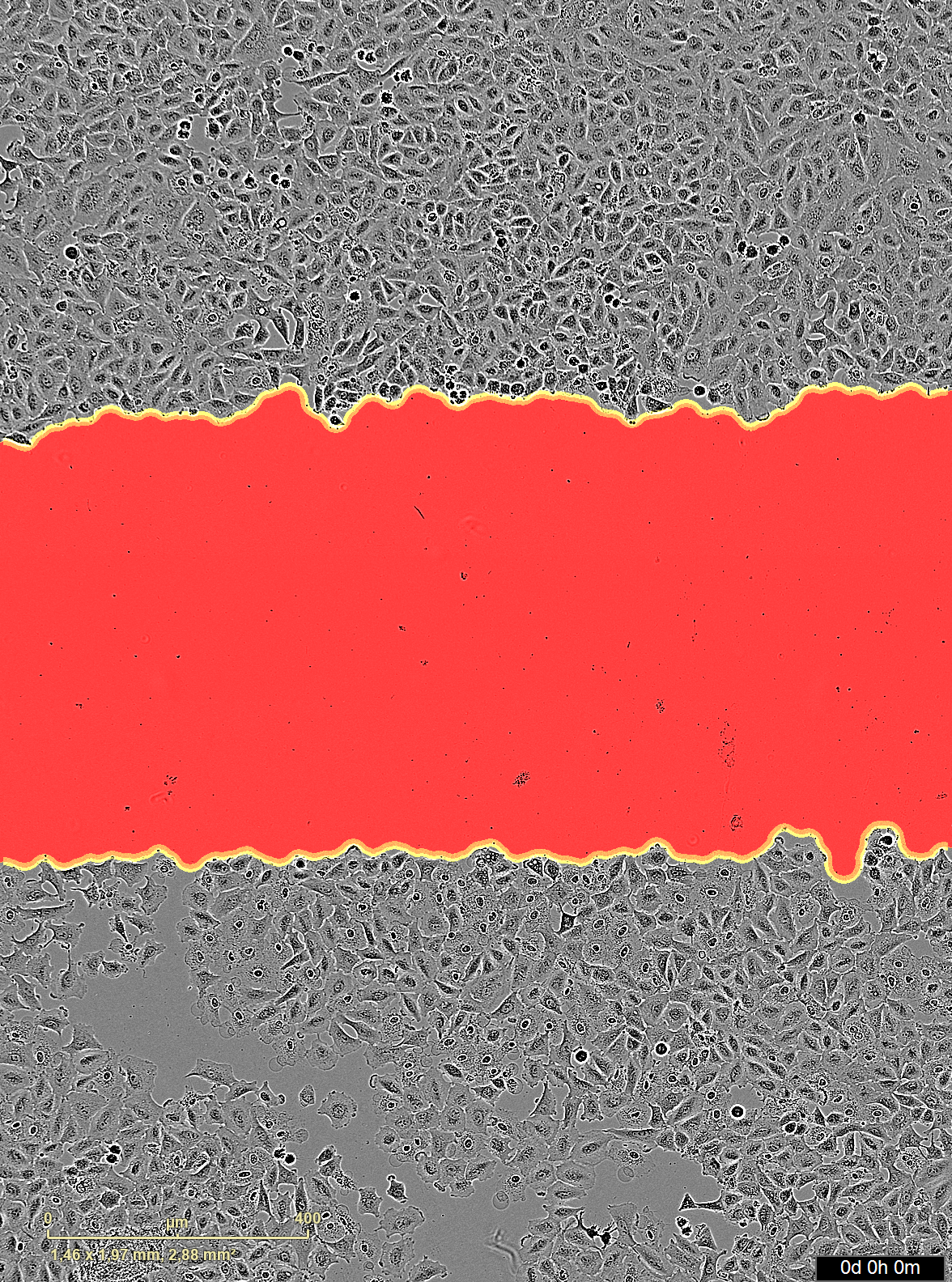

Supplement: Supplementary file 5 — Source Data for Figure 2 [file EMMM-15-e17836-s004.zip › Figure_2/Fig_2J/Images/A549_SP_0h.tif]

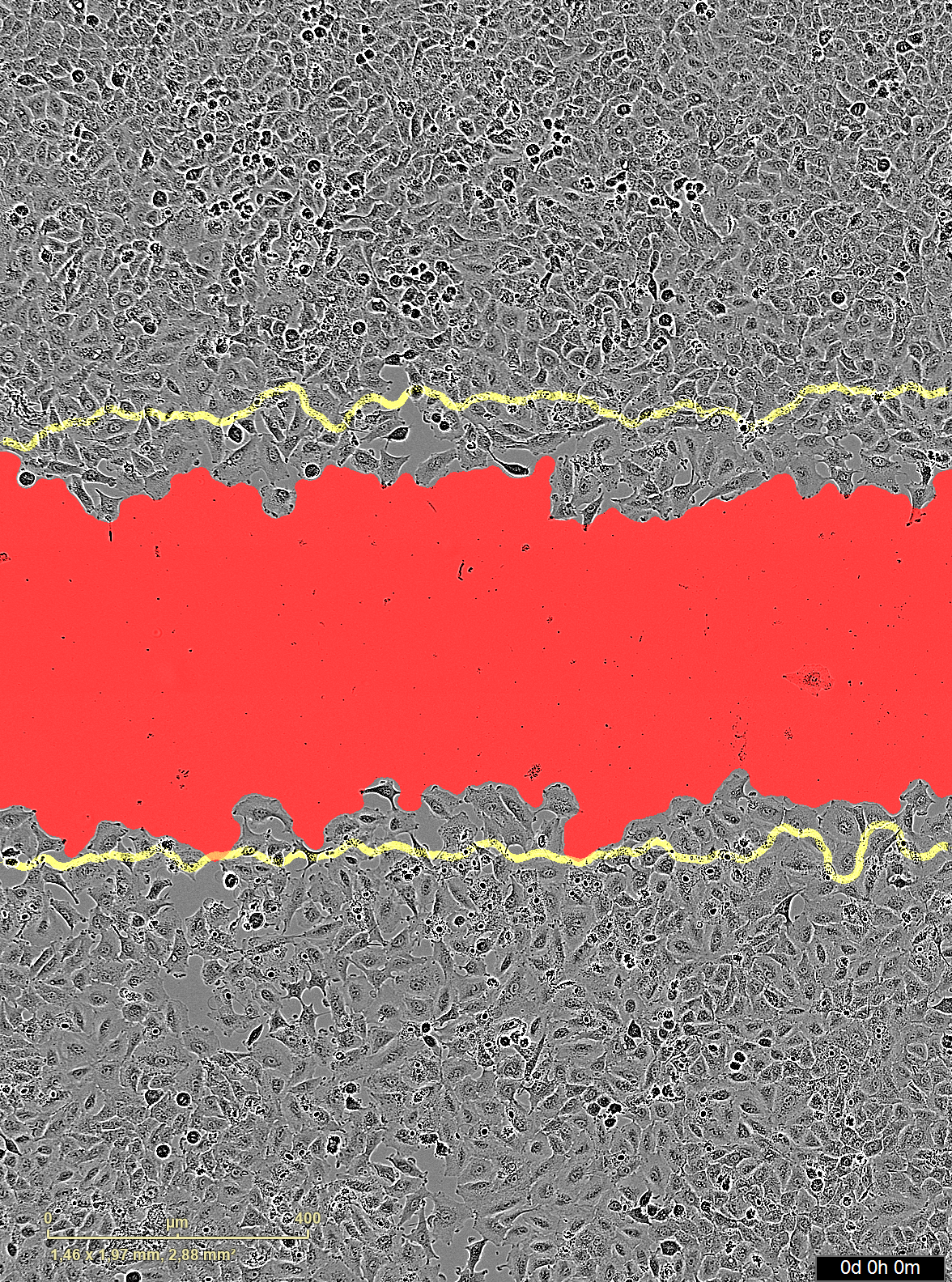

Supplement: Supplementary file 5 — Source Data for Figure 2 [file EMMM-15-e17836-s004.zip › Figure_2/Fig_2J/Images/A549_SP_24h.tif]

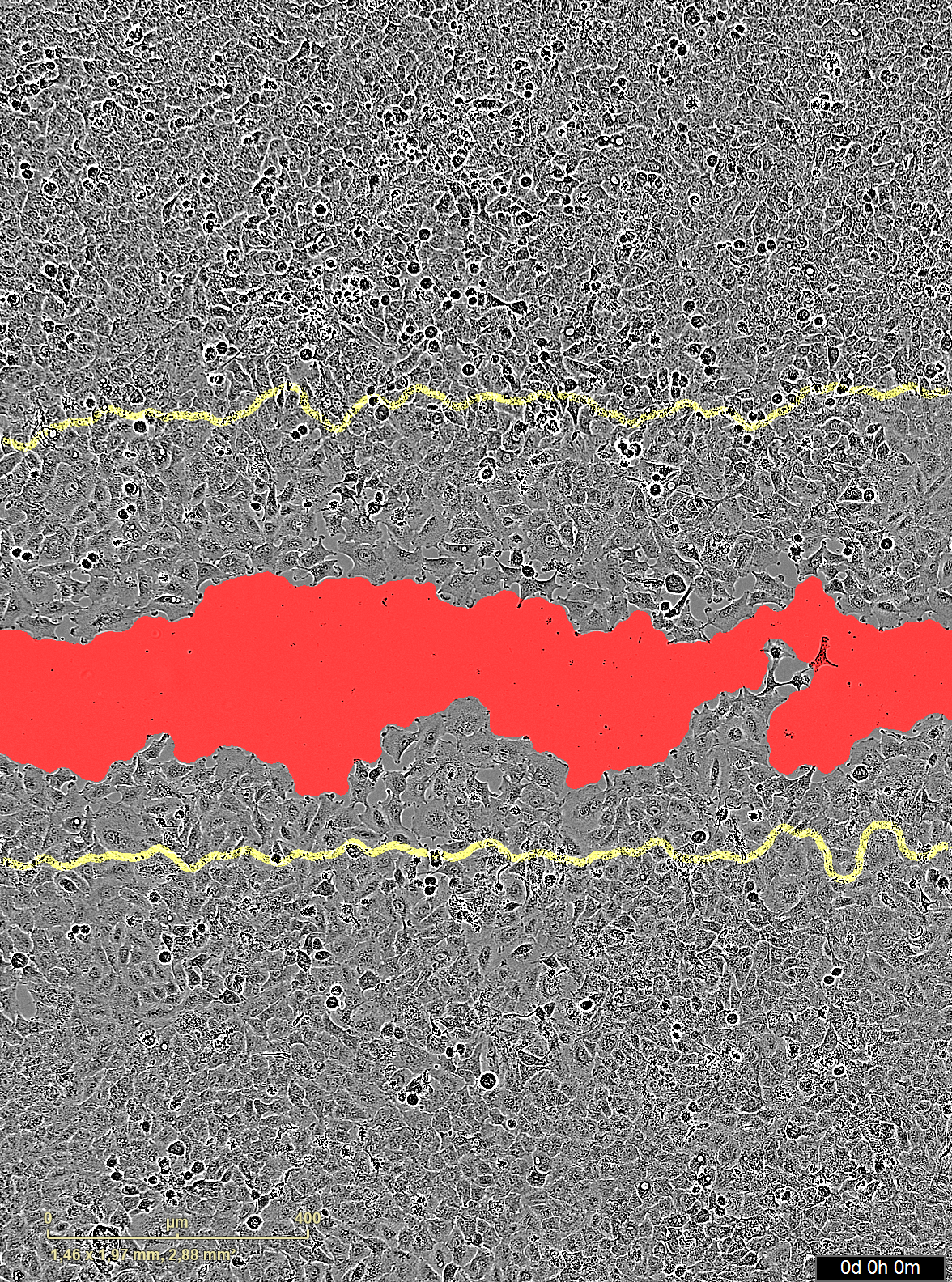

Supplement: Supplementary file 5 — Source Data for Figure 2 [file EMMM-15-e17836-s004.zip › Figure_2/Fig_2J/Images/A549_SP_48h.tif]

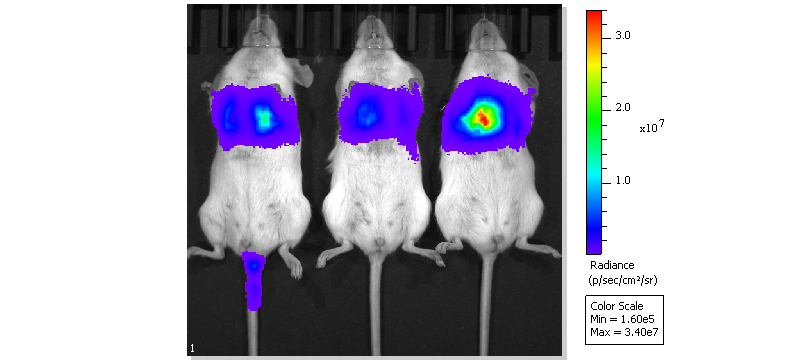

Supplement: Supplementary file 6 — Source Data for Figure 3 [file EMMM-15-e17836-s006.zip › Figure_3/Fig_3B/A549_PFUL2G_Control_799_800_801_5sec.png]

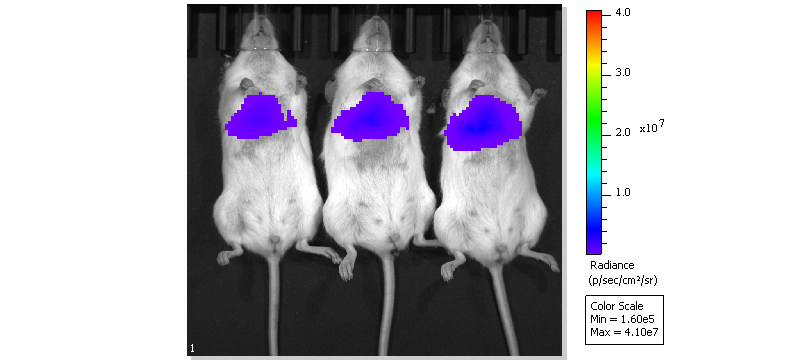

Supplement: Supplementary file 6 — Source Data for Figure 3 [file EMMM-15-e17836-s006.zip › Figure_3/Fig_3B/A549_PFUL2G_SP_790_791_792_5sec.png]

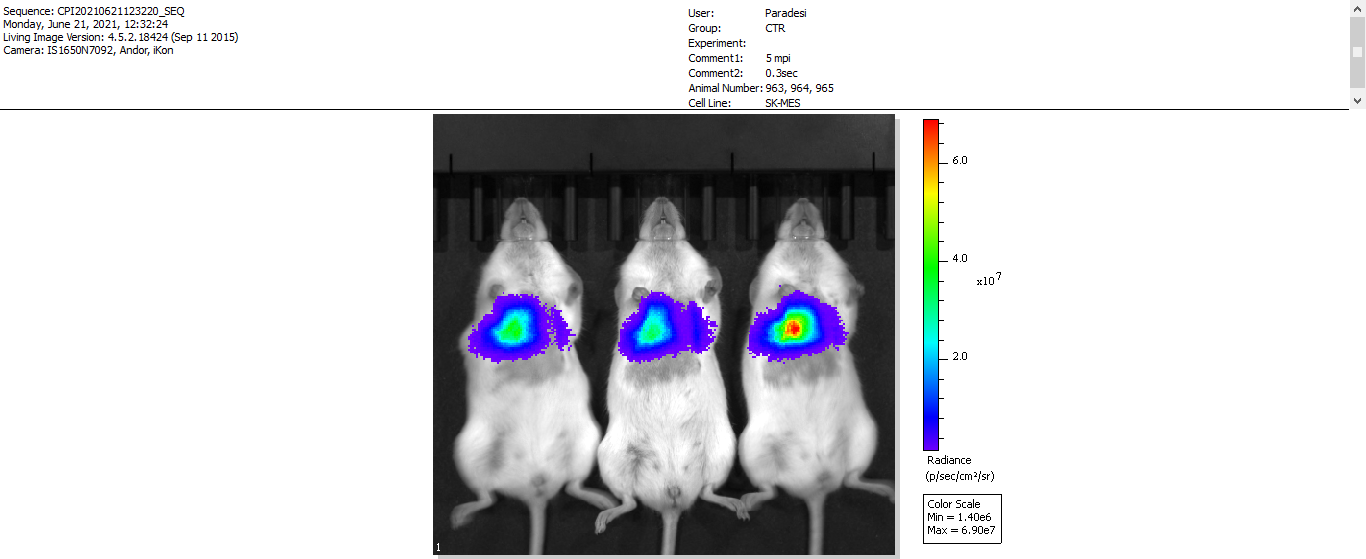

Supplement: Supplementary file 6 — Source Data for Figure 3 [file EMMM-15-e17836-s006.zip › Figure_3/Fig_3E/SKMES1_PFUL2G_Control_2963_2964_2965_5mpi.png]

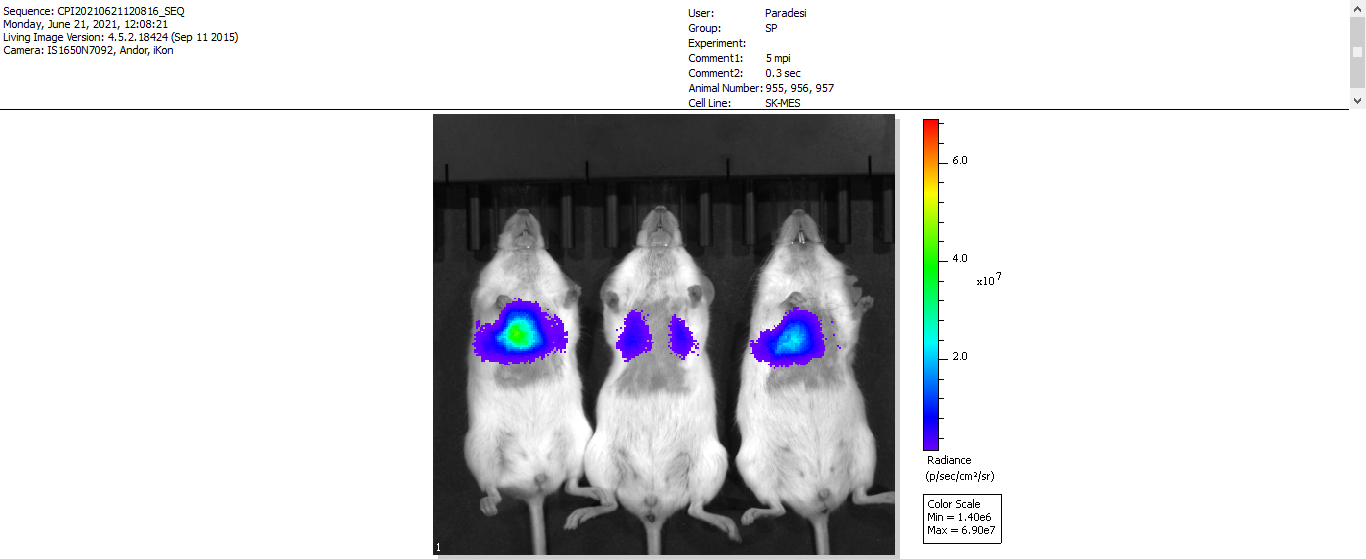

Supplement: Supplementary file 6 — Source Data for Figure 3 [file EMMM-15-e17836-s006.zip › Figure_3/Fig_3E/SKMES1_PFUL2G_SP_2955_2956_2957_5mpi.png]

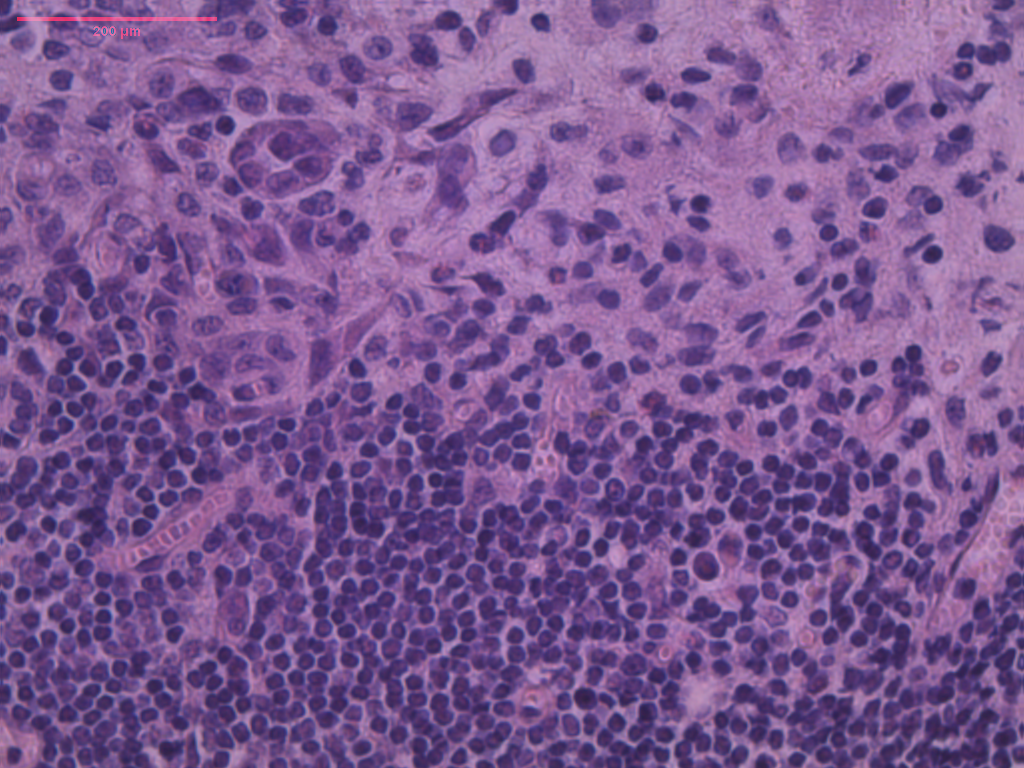

Supplement: Supplementary file 6 — Source Data for Figure 3 [file EMMM-15-e17836-s006.zip › Figure_3/Fig_3I/LN_Control_MAS_53_40X.tif]

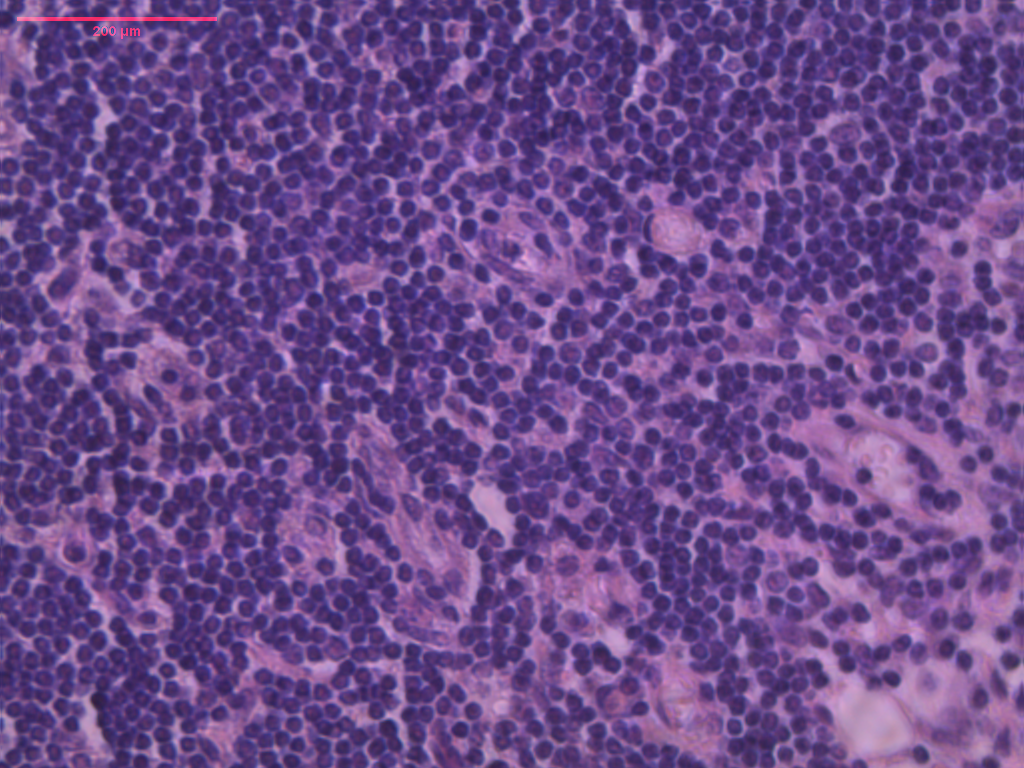

Supplement: Supplementary file 6 — Source Data for Figure 3 [file EMMM-15-e17836-s006.zip › Figure_3/Fig_3I/LN_SP_MAS_66_40X.tif]

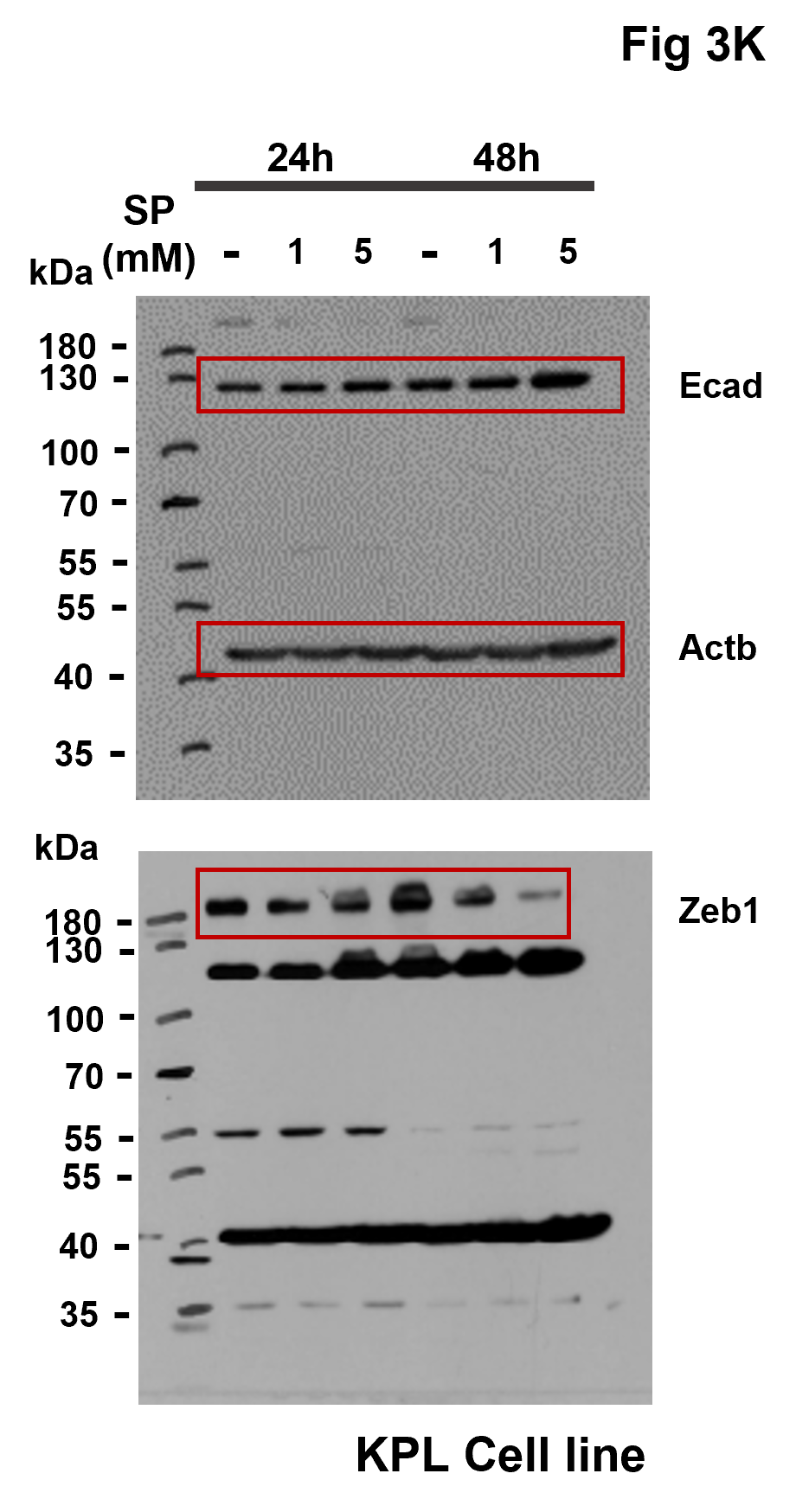

Supplement: Supplementary file 6 — Source Data for Figure 3 [file EMMM-15-e17836-s006.zip › Figure_3/Fig_3K/Fig_3K.tif]

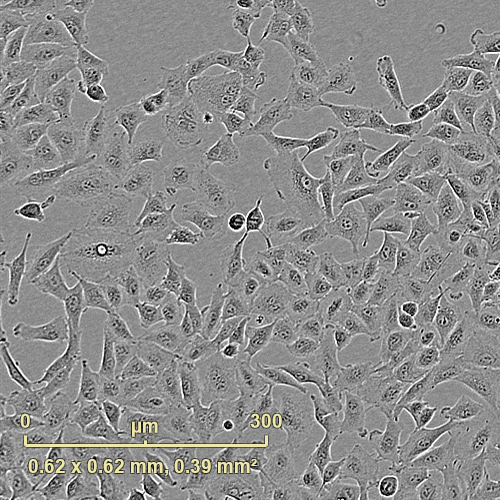

Supplement: Supplementary file 7 — Source Data for Figure 4 [file EMMM-15-e17836-s009.zip › Figure_4/Fig_4A/A549_Control_10uM.tif]

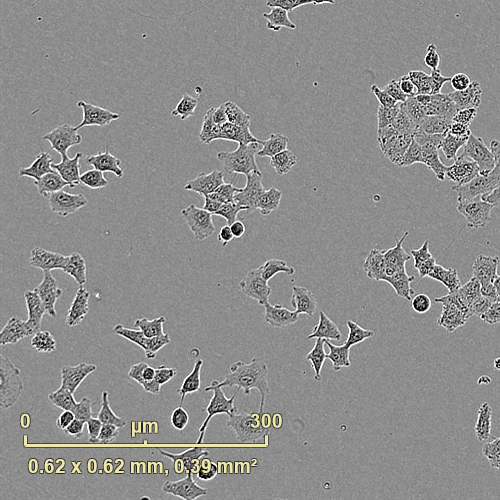

Supplement: Supplementary file 7 — Source Data for Figure 4 [file EMMM-15-e17836-s009.zip › Figure_4/Fig_4A/A549_SP_10uM.tif]

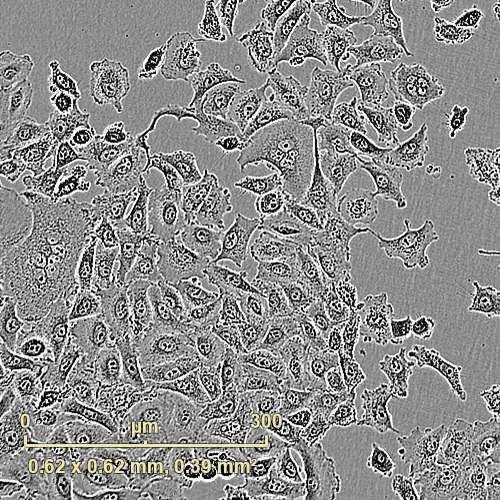

Supplement: Supplementary file 7 — Source Data for Figure 4 [file EMMM-15-e17836-s009.zip › Figure_4/Fig_4B/SKMES1_Control_5uM.tif]

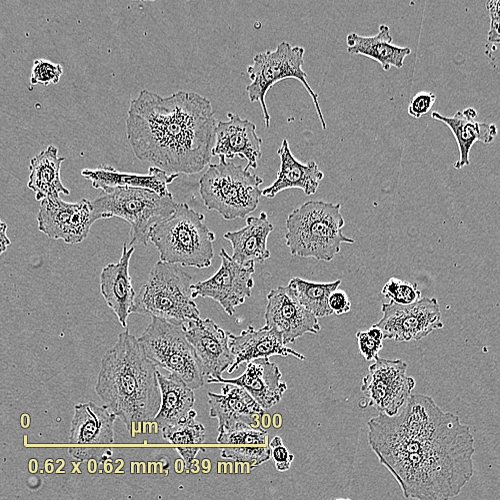

Supplement: Supplementary file 7 — Source Data for Figure 4 [file EMMM-15-e17836-s009.zip › Figure_4/Fig_4B/SKMES1_SP_5uM.tif]

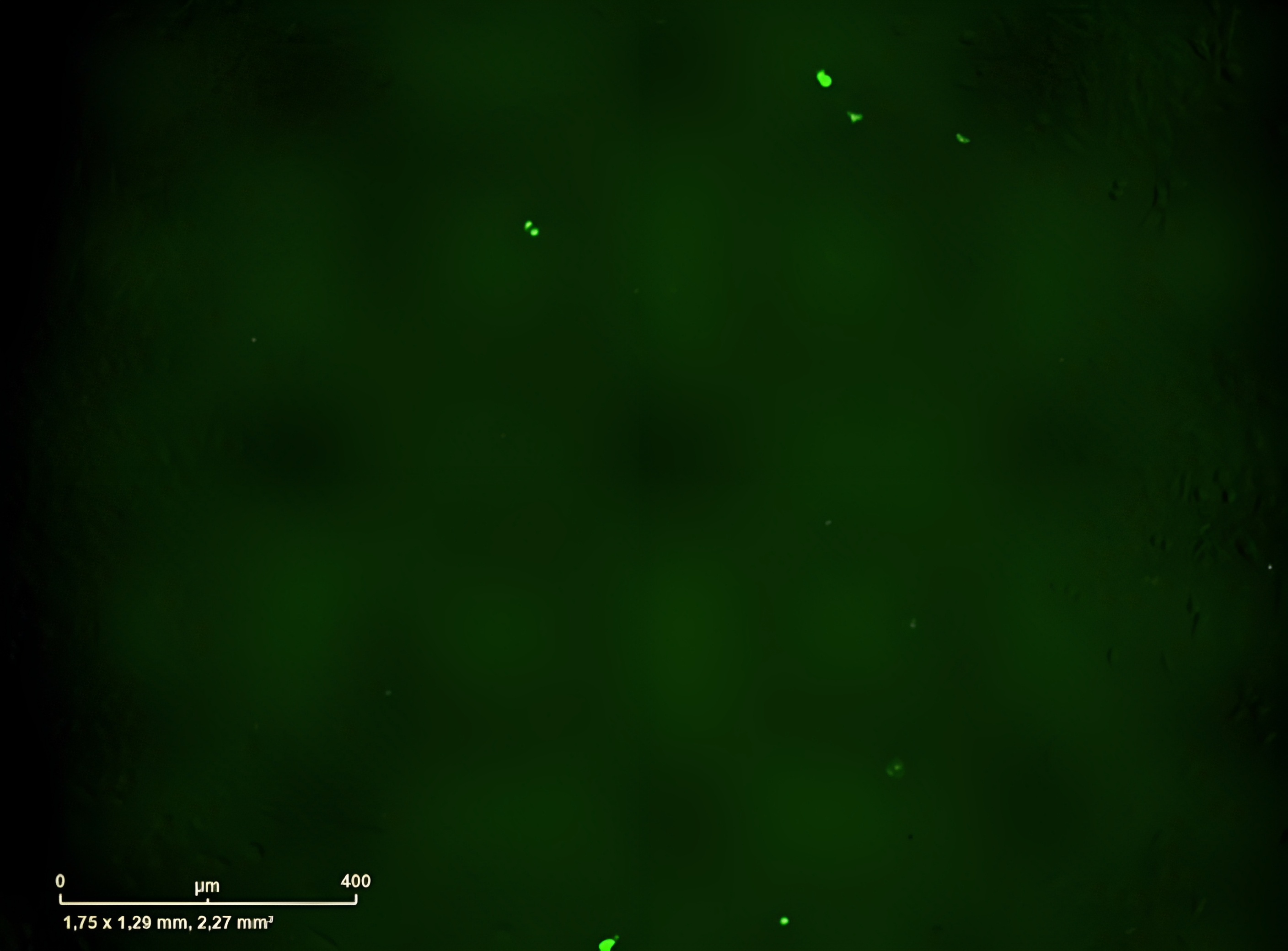

Supplement: Supplementary file 7 — Source Data for Figure 4 [file EMMM-15-e17836-s009.zip › Figure_4/Fig_4E/A549_Control_CDDP_0uM.jpg]

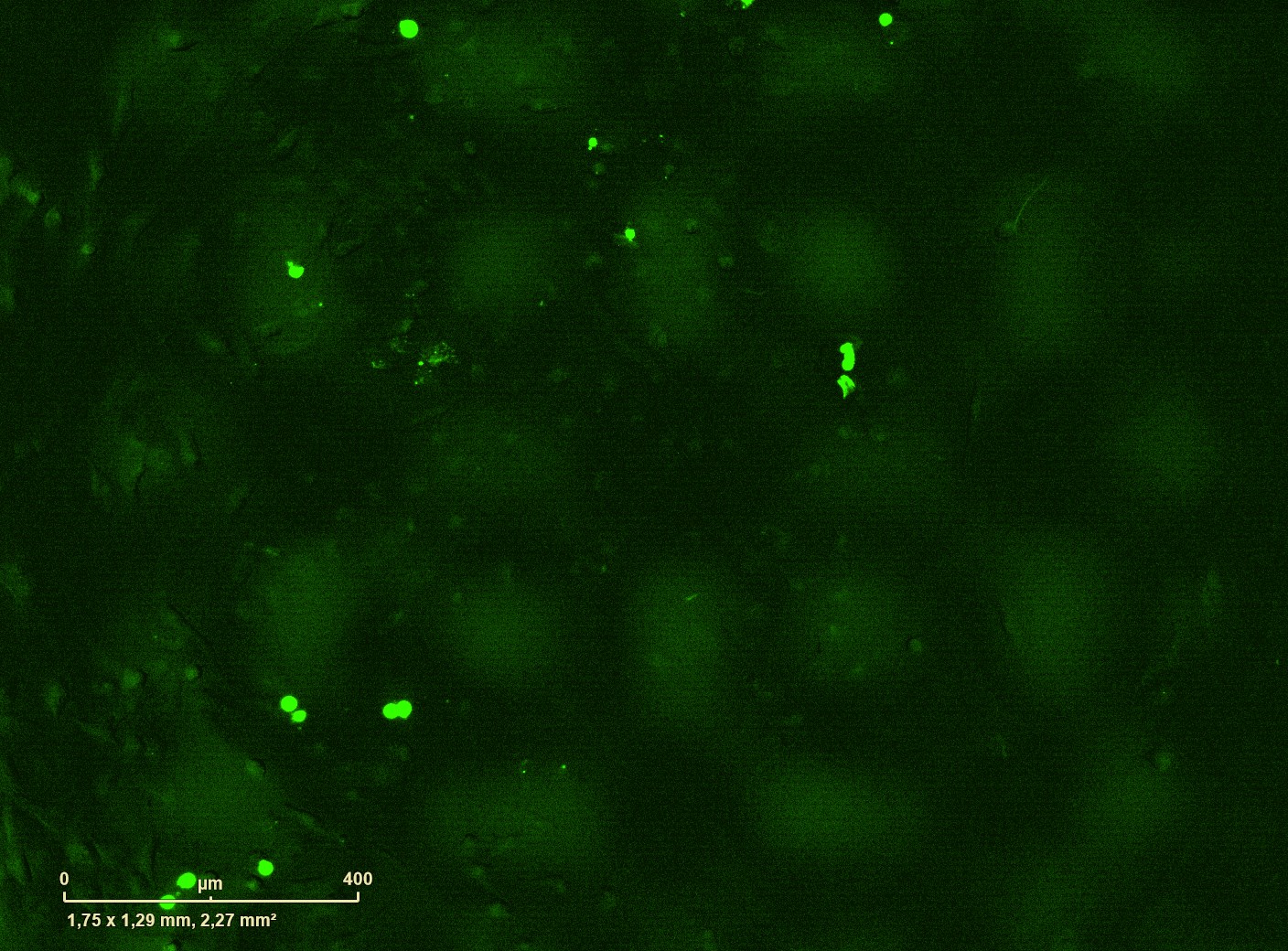

Supplement: Supplementary file 7 — Source Data for Figure 4 [file EMMM-15-e17836-s009.zip › Figure_4/Fig_4E/A549_Control_CDDP_5uM.jpg]

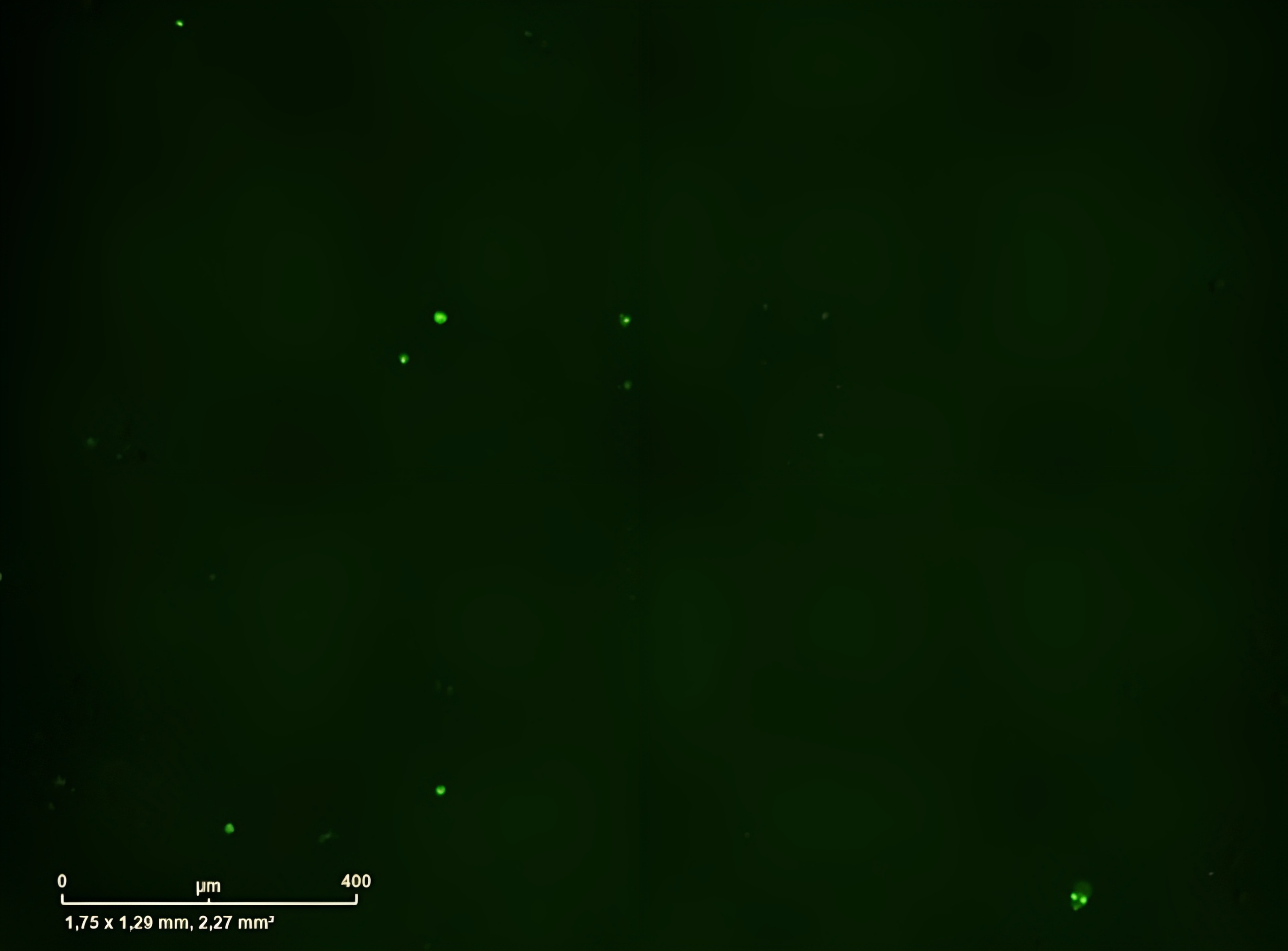

Supplement: Supplementary file 7 — Source Data for Figure 4 [file EMMM-15-e17836-s009.zip › Figure_4/Fig_4E/A549_SP_CDDP_0uM.jpg]

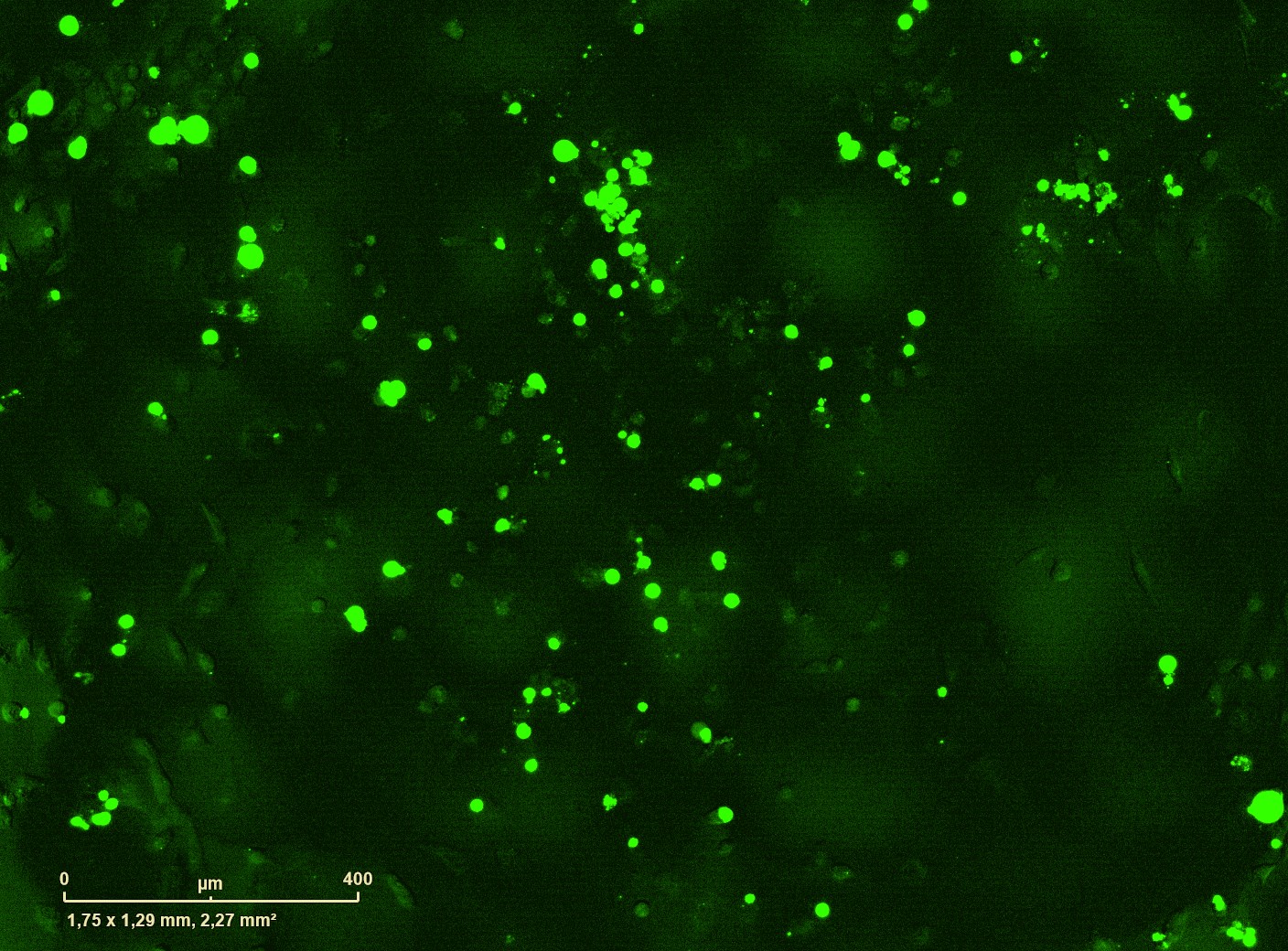

Supplement: Supplementary file 7 — Source Data for Figure 4 [file EMMM-15-e17836-s009.zip › Figure_4/Fig_4E/A549_SP_CDDP_5uM.jpg]

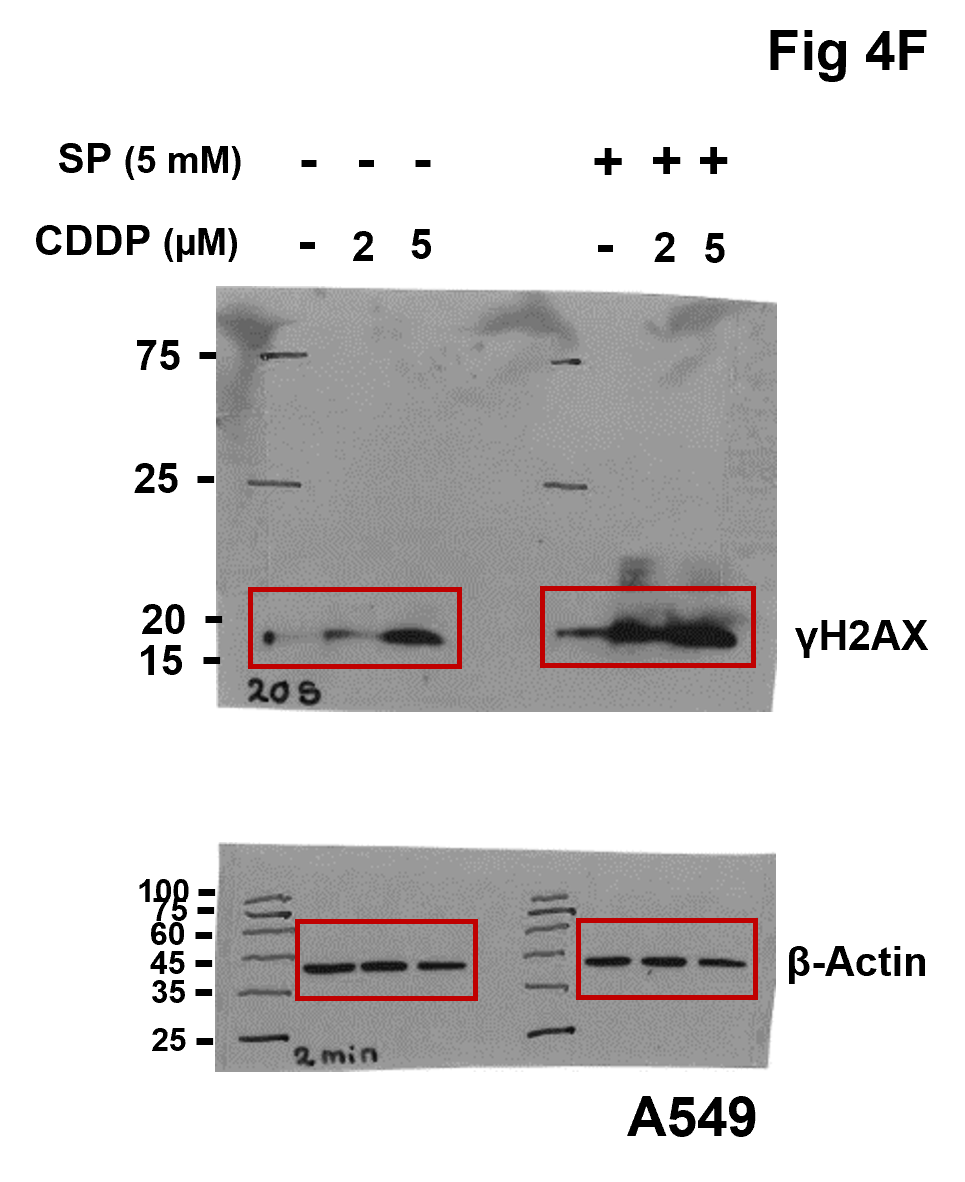

Supplement: Supplementary file 7 — Source Data for Figure 4 [file EMMM-15-e17836-s009.zip › Figure_4/Fig_4F/Fig_4F.tif]

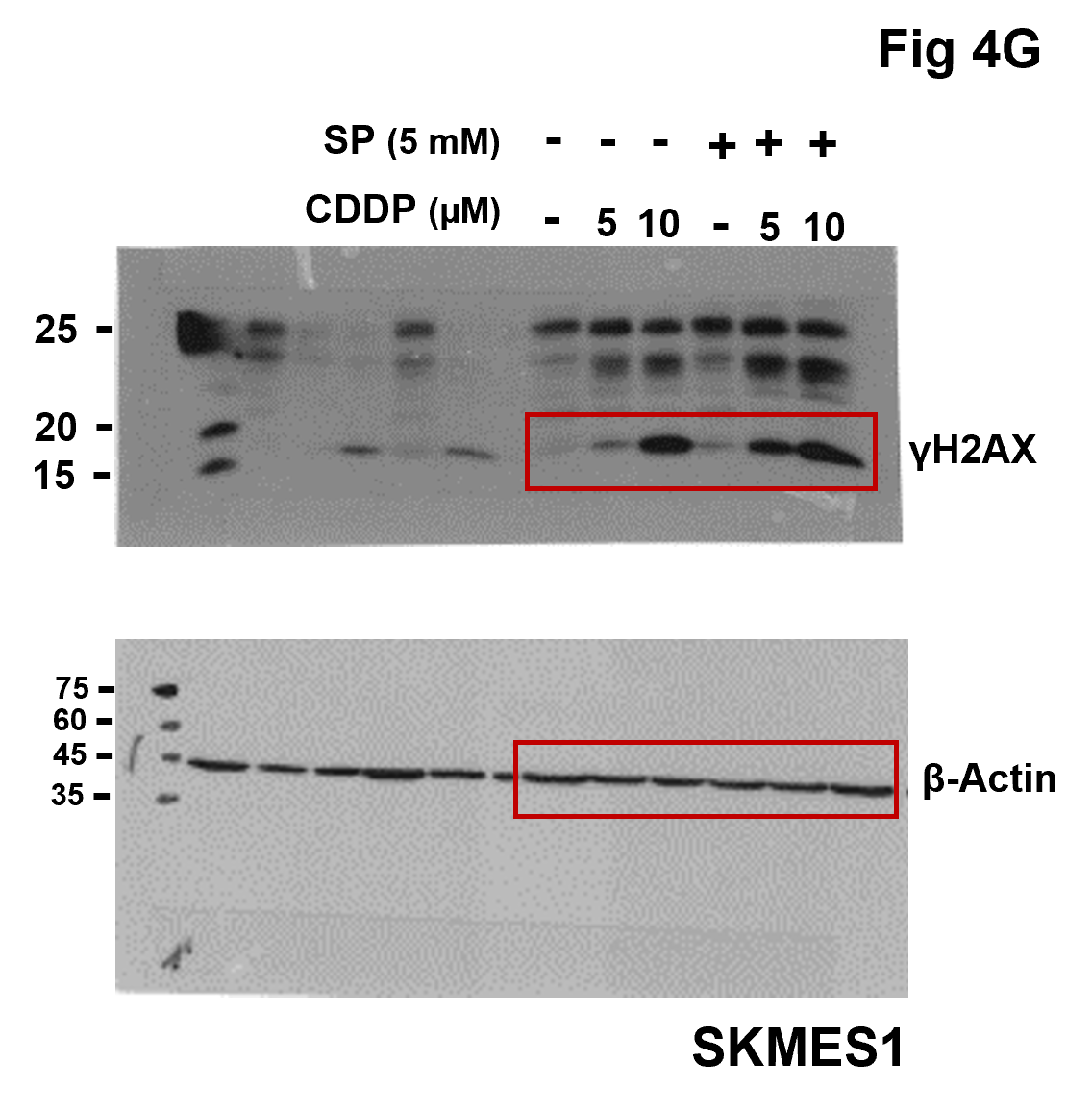

Supplement: Supplementary file 7 — Source Data for Figure 4 [file EMMM-15-e17836-s009.zip › Figure_4/Fig_4G/Fig_4G.tif]

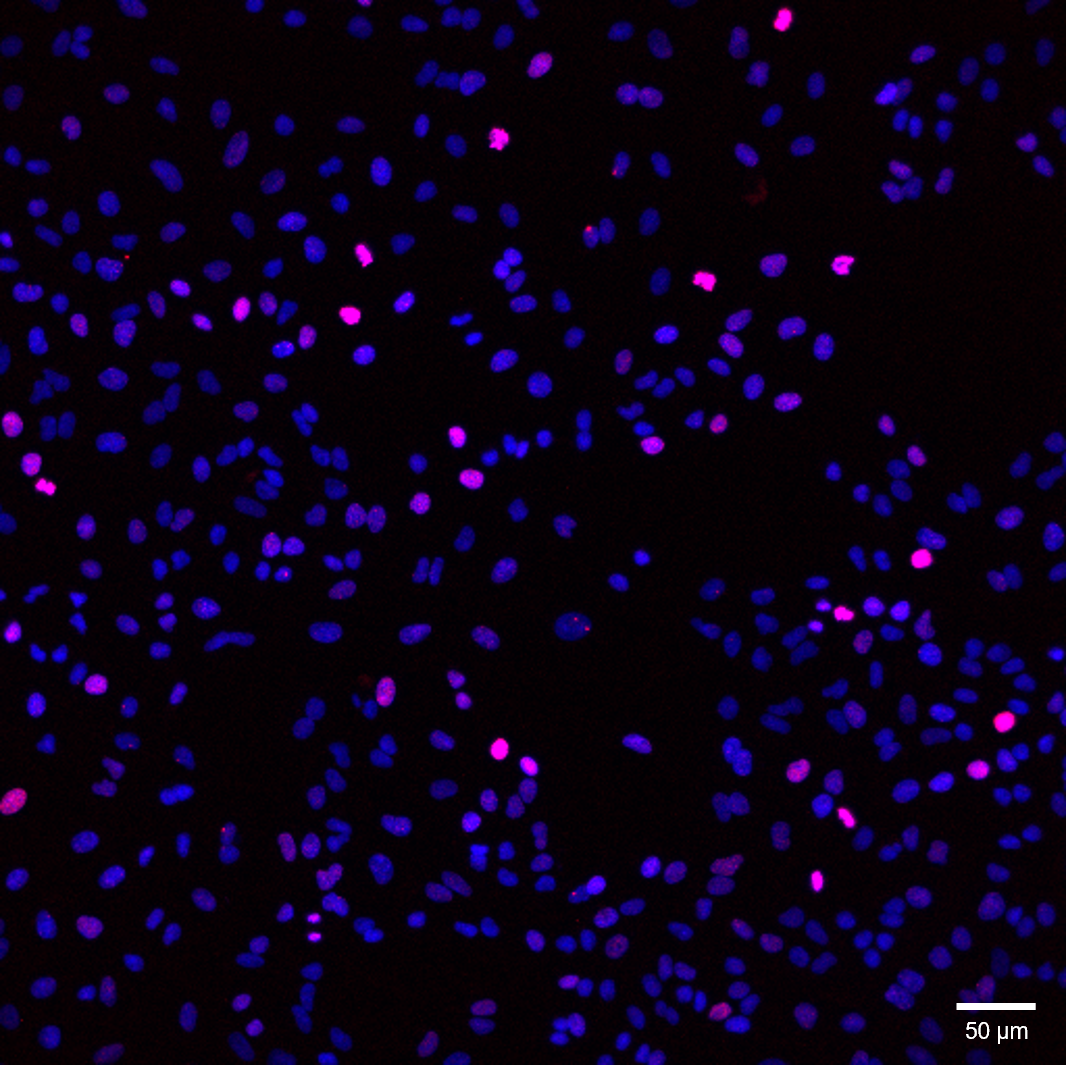

Supplement: Supplementary file 7 — Source Data for Figure 4 [file EMMM-15-e17836-s009.zip › Figure_4/Fig_4H/A549_Control_CDDP_0uM.tif]

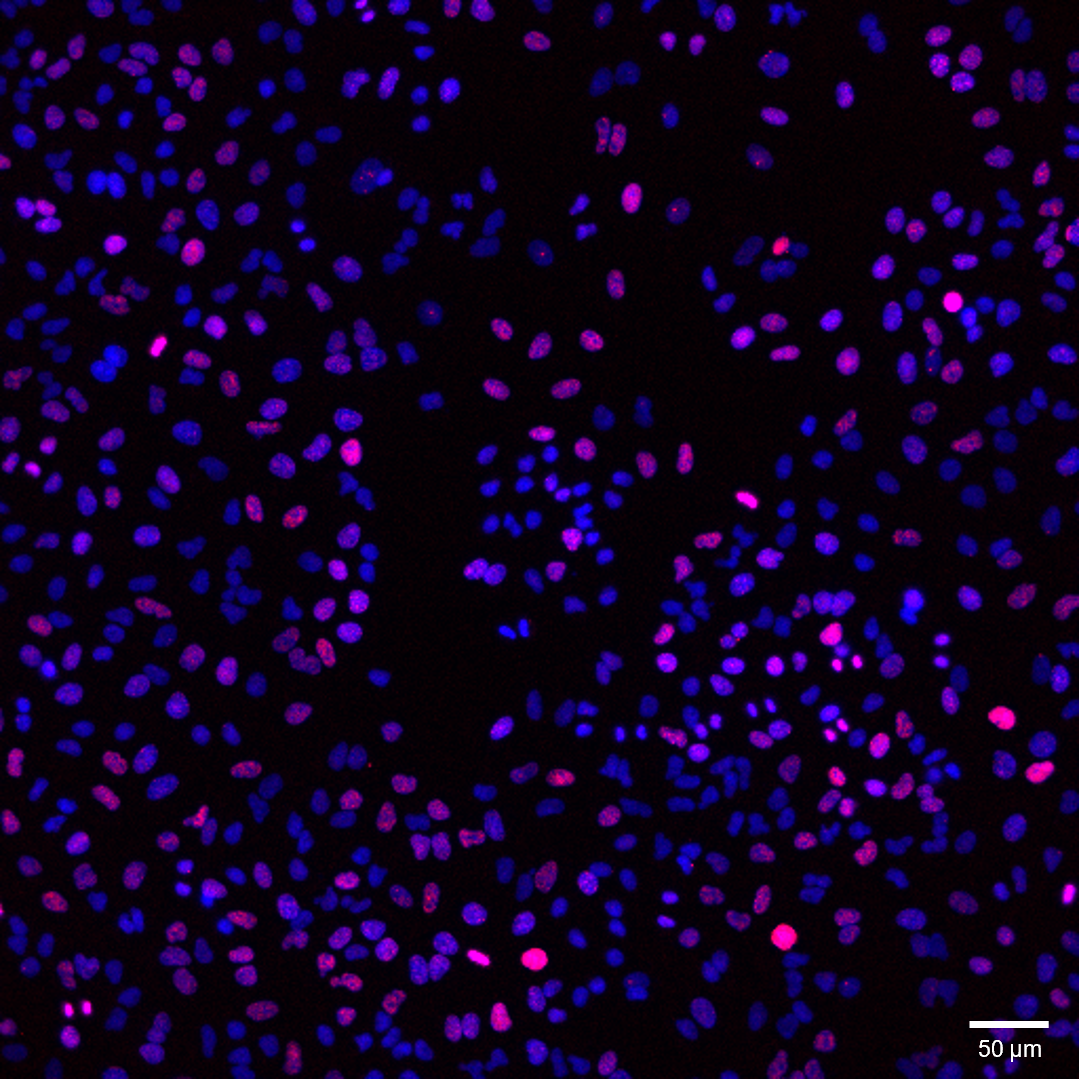

Supplement: Supplementary file 7 — Source Data for Figure 4 [file EMMM-15-e17836-s009.zip › Figure_4/Fig_4H/A549_Control_CDDP_5uM.tif]

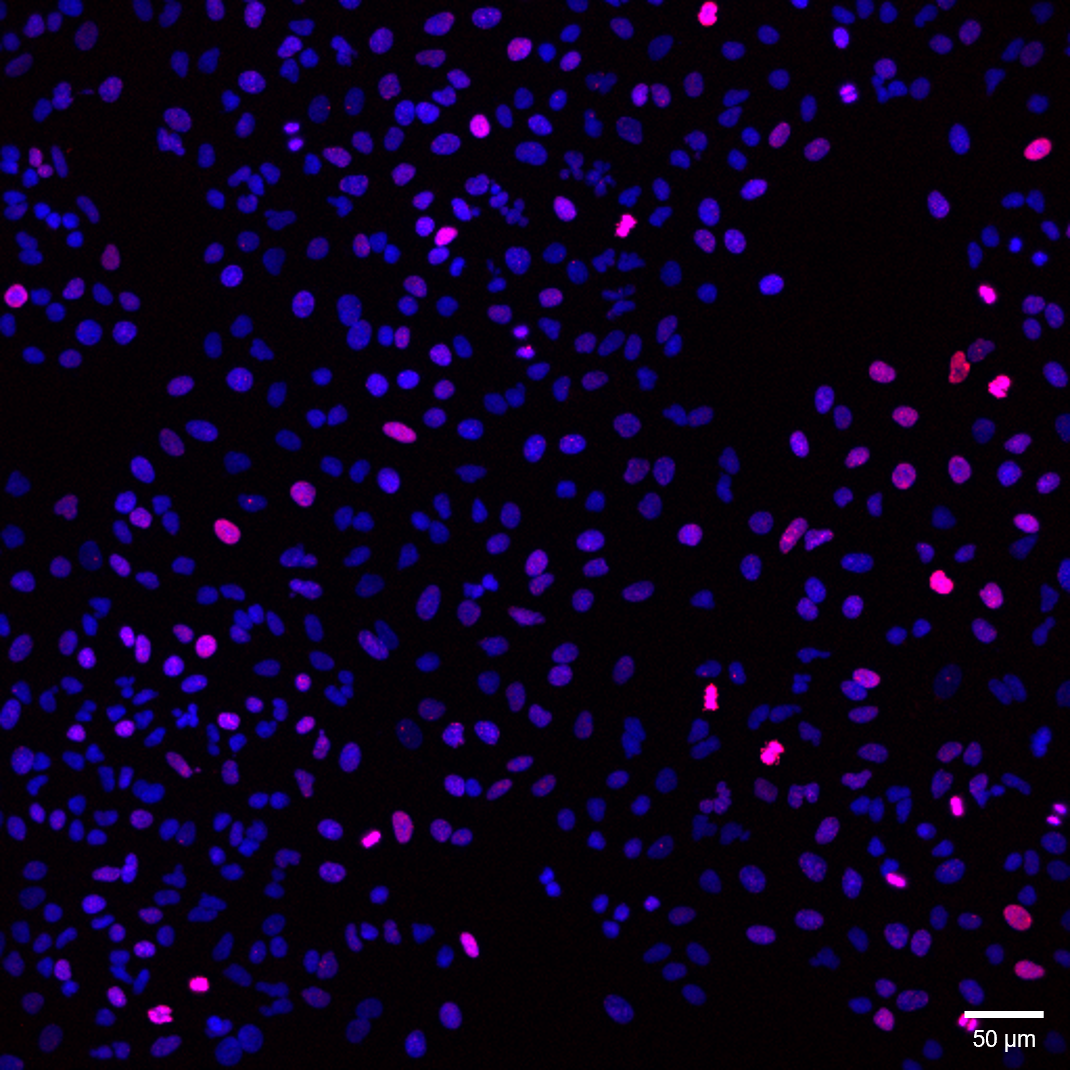

Supplement: Supplementary file 7 — Source Data for Figure 4 [file EMMM-15-e17836-s009.zip › Figure_4/Fig_4H/A549_SP_CDDP_0uM.tif]

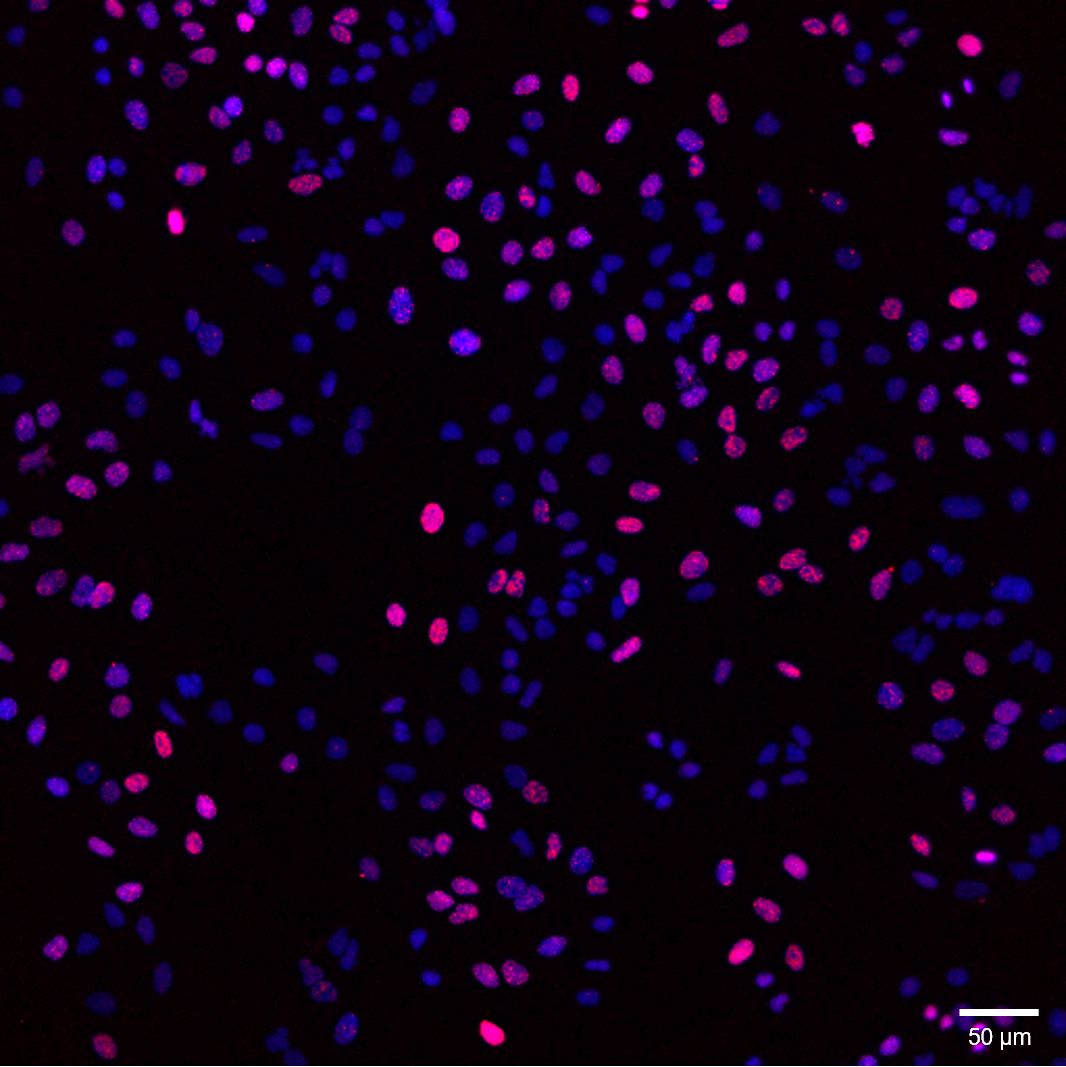

Supplement: Supplementary file 7 — Source Data for Figure 4 [file EMMM-15-e17836-s009.zip › Figure_4/Fig_4H/A549_SP_CDDP_5uM.tif]

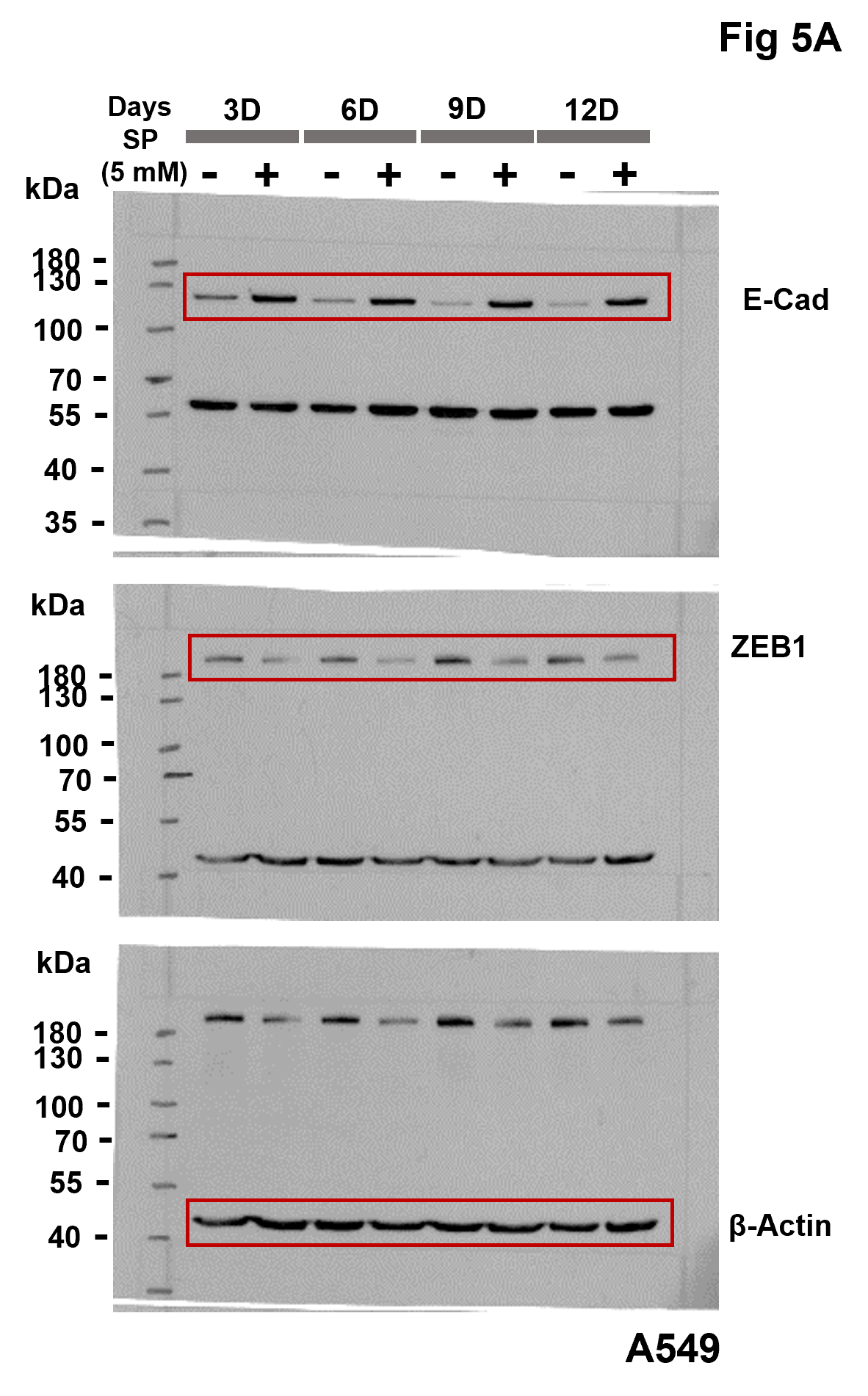

Supplement: Supplementary file 8 — Source Data for Figure 5 [file EMMM-15-e17836-s011.zip › Figure_5/Fig_5A/Fig_5A.tif]

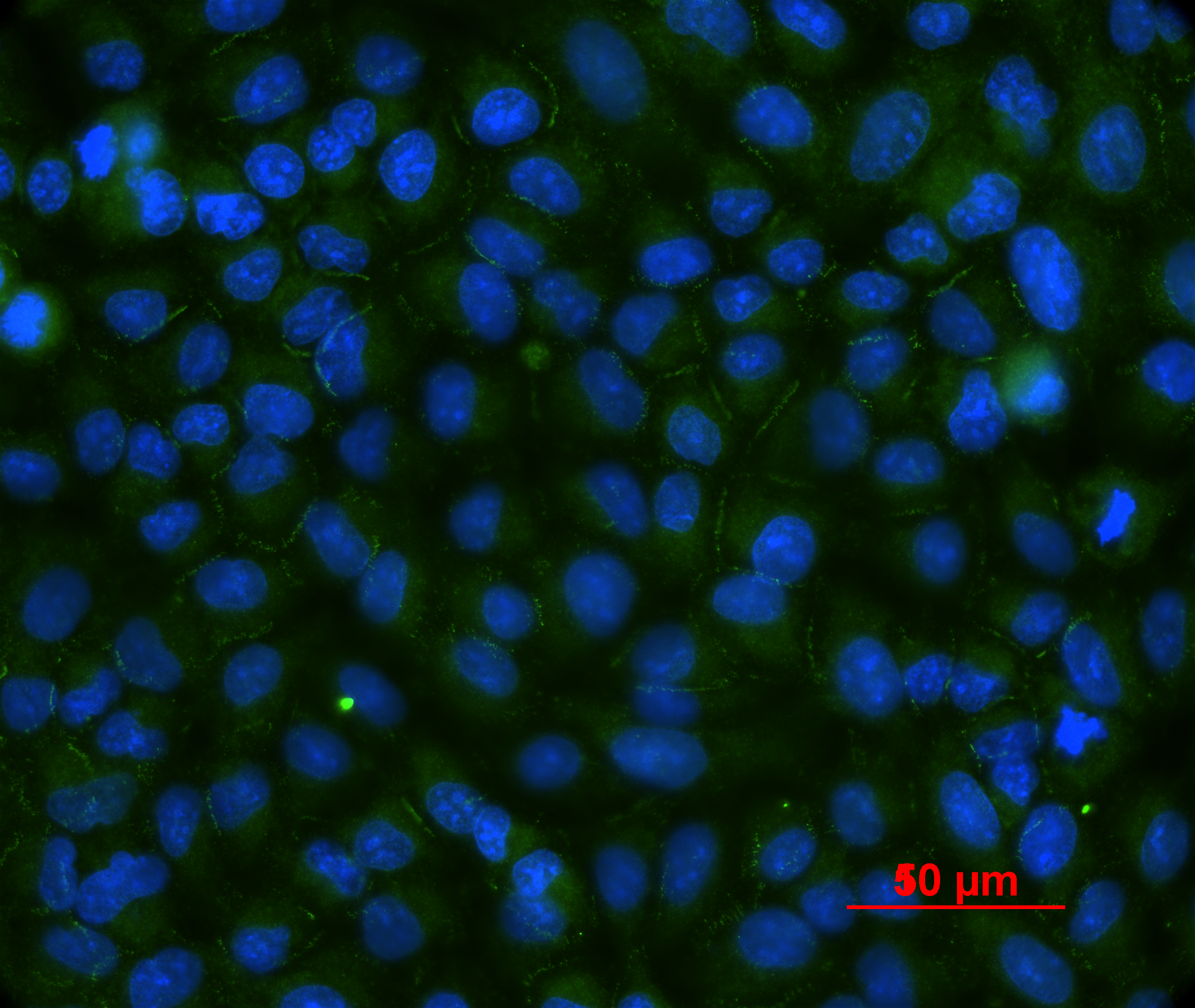

Supplement: Supplementary file 8 — Source Data for Figure 5 [file EMMM-15-e17836-s011.zip › Figure_5/Fig_5E/A549_Control_EPCAM_4_RGB.tif]

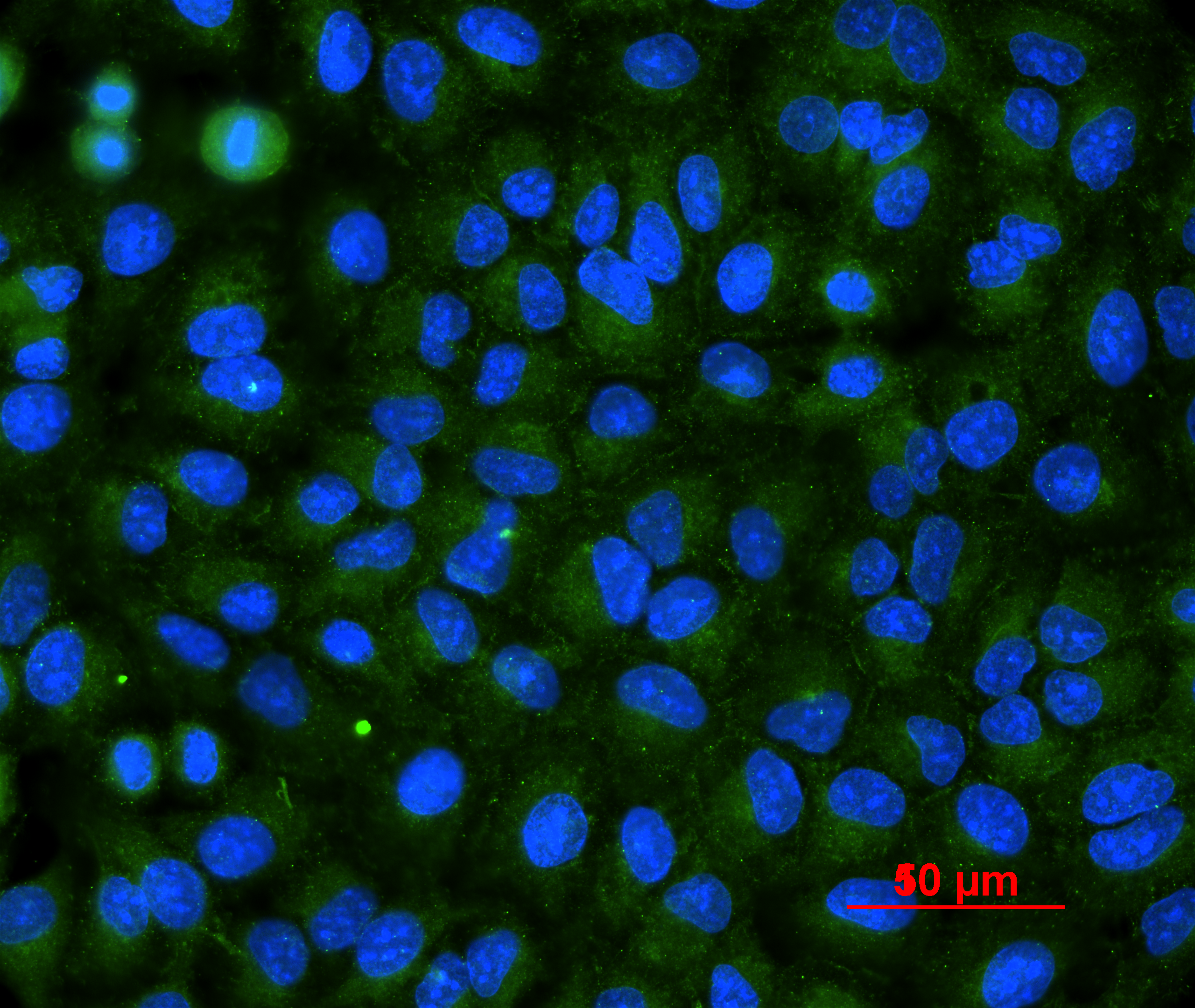

Supplement: Supplementary file 8 — Source Data for Figure 5 [file EMMM-15-e17836-s011.zip › Figure_5/Fig_5E/A549_Control_ZO1_9_RGB.tif]

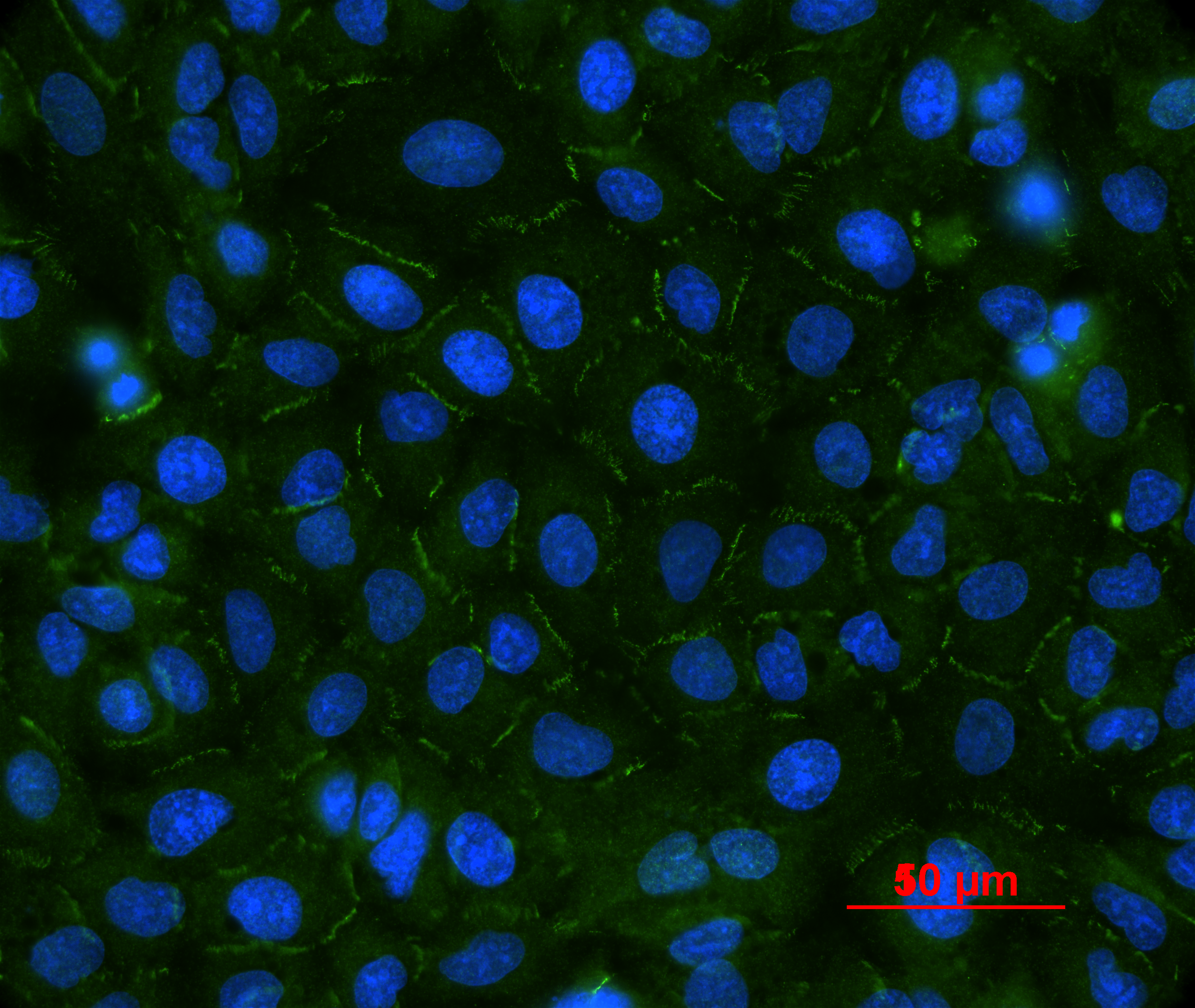

Supplement: Supplementary file 8 — Source Data for Figure 5 [file EMMM-15-e17836-s011.zip › Figure_5/Fig_5E/A549_SP_EPCAM_7_RGB.tif]

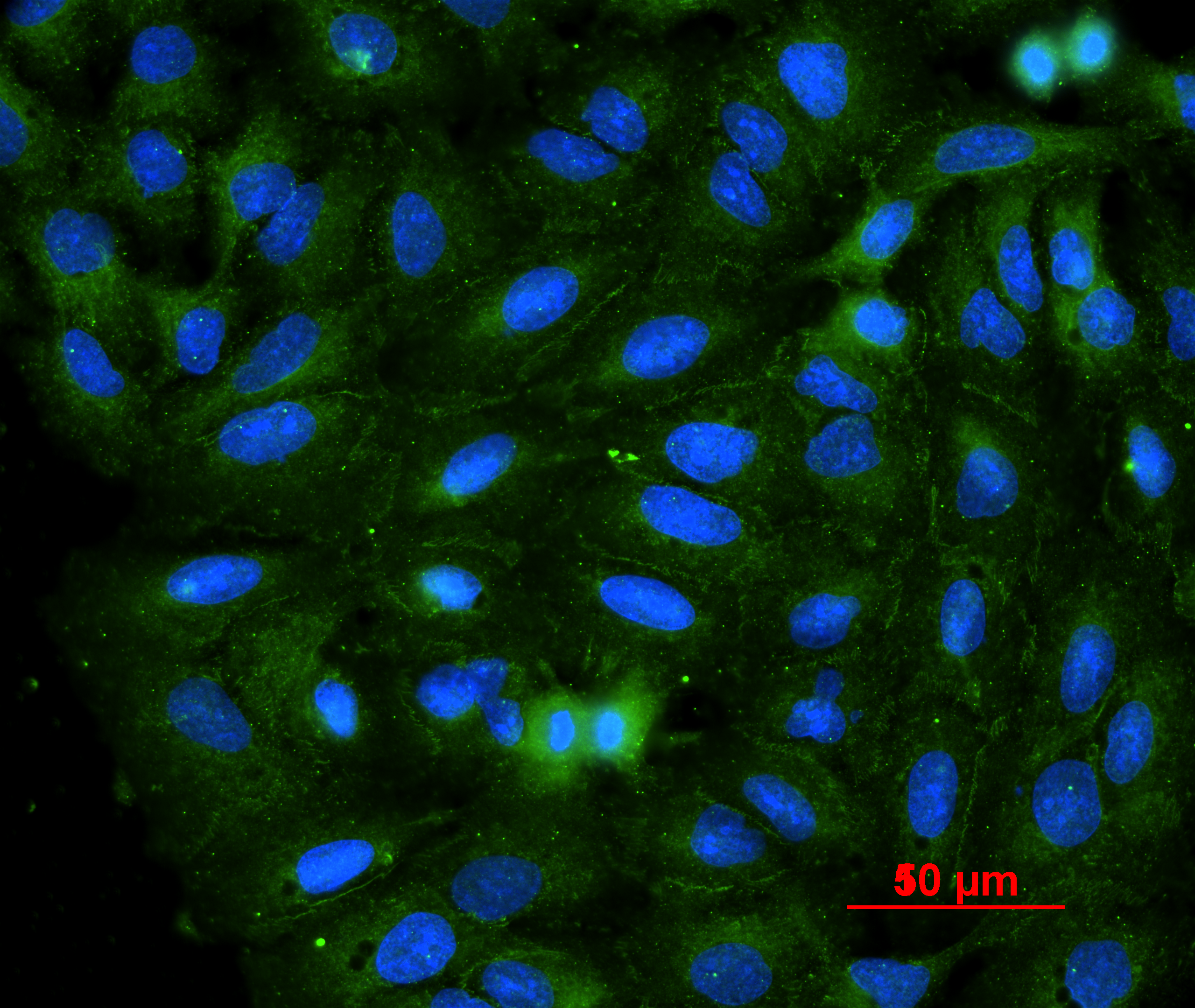

Supplement: Supplementary file 8 — Source Data for Figure 5 [file EMMM-15-e17836-s011.zip › Figure_5/Fig_5E/A549_SP_ZO1_6_RGB.tif]

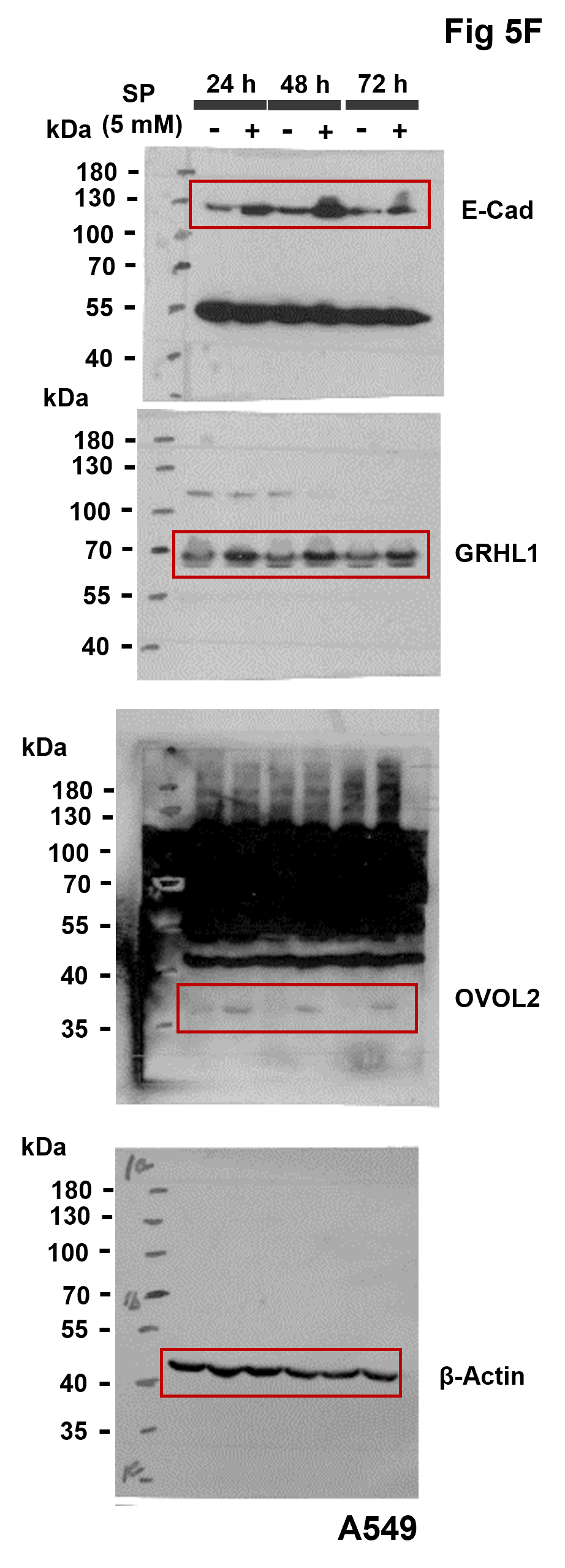

Supplement: Supplementary file 8 — Source Data for Figure 5 [file EMMM-15-e17836-s011.zip › Figure_5/Fig_5F/Fig_5F.tif]

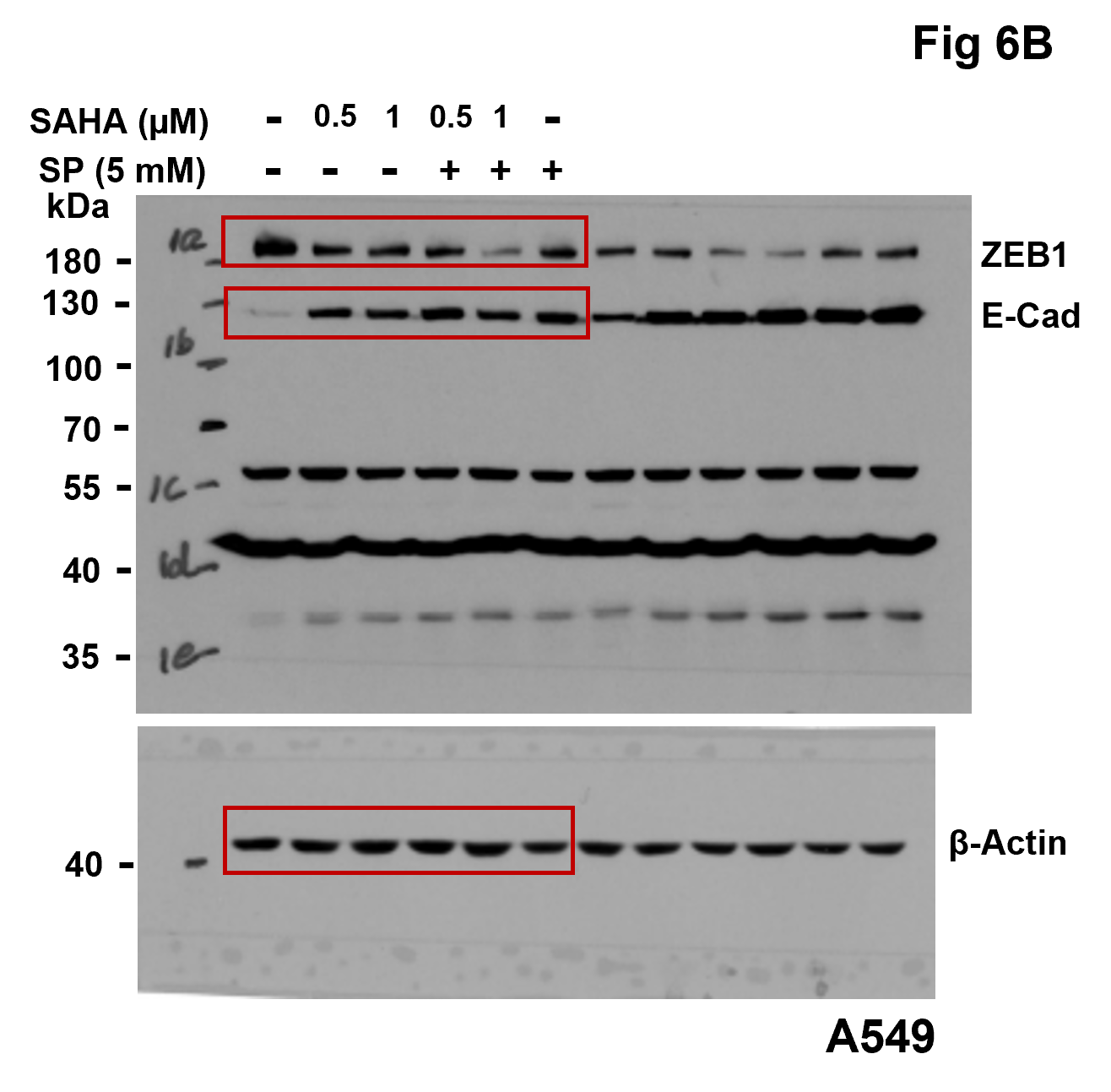

Supplement: Supplementary file 9 — Source Data for Figure 6 [file EMMM-15-e17836-s003.zip › Figure_6/Fig_6B/Fig_6B.tif]

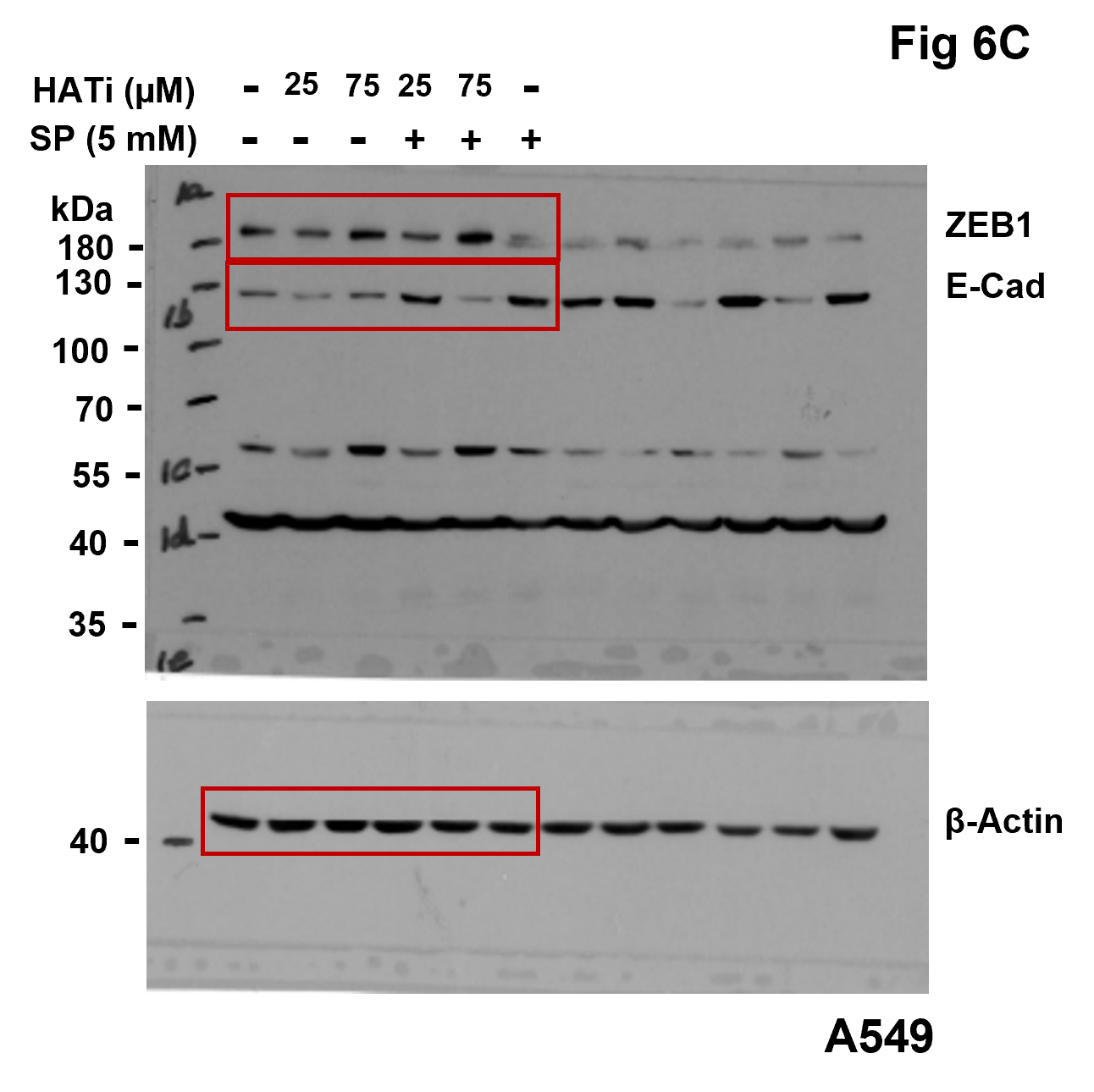

Supplement: Supplementary file 9 — Source Data for Figure 6 [file EMMM-15-e17836-s003.zip › Figure_6/Fig_6C/Fig_6C.tif]

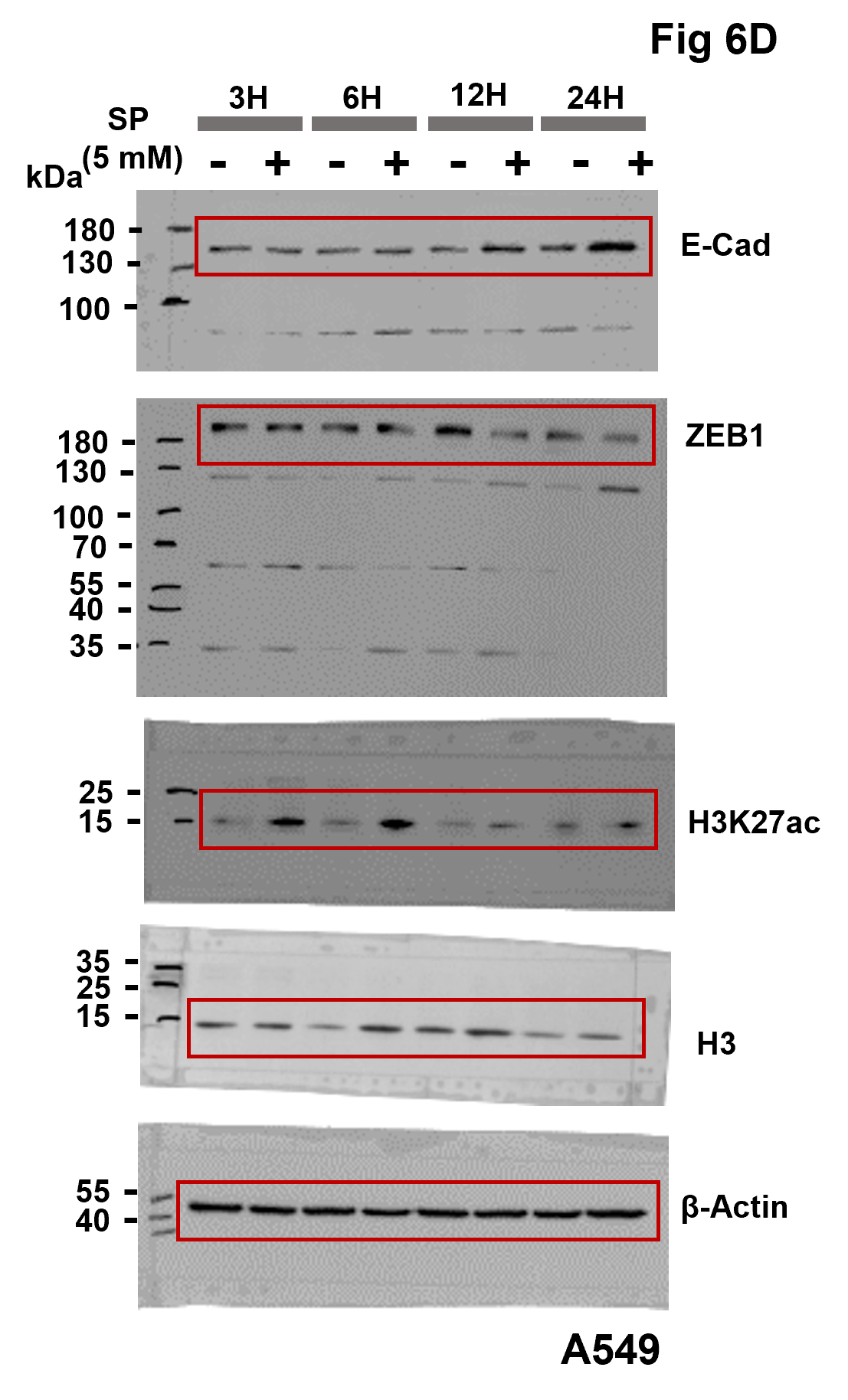

Supplement: Supplementary file 9 — Source Data for Figure 6 [file EMMM-15-e17836-s003.zip › Figure_6/Fig_6D/Fig_6D.tif]

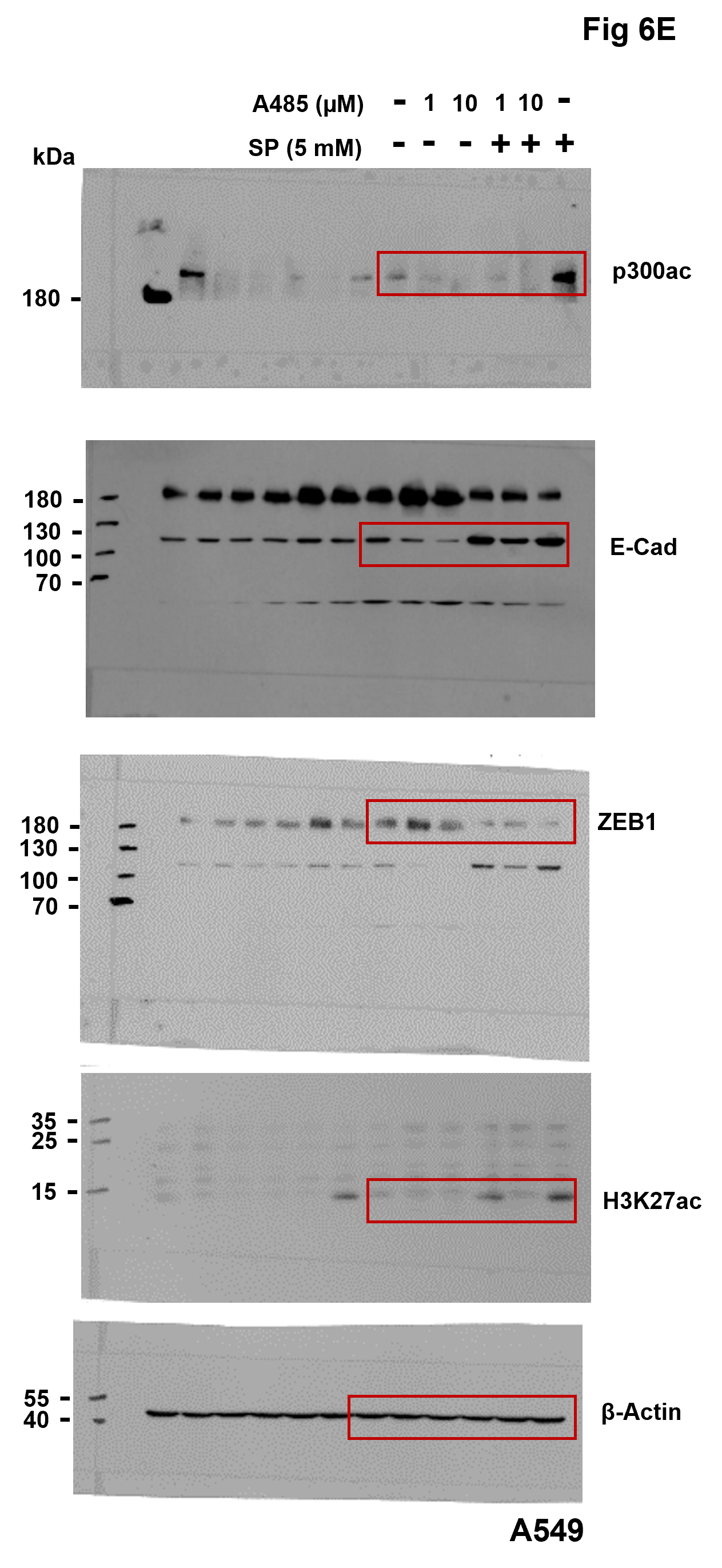

Supplement: Supplementary file 9 — Source Data for Figure 6 [file EMMM-15-e17836-s003.zip › Figure_6/Fig_6E/Fig_6E.tif]

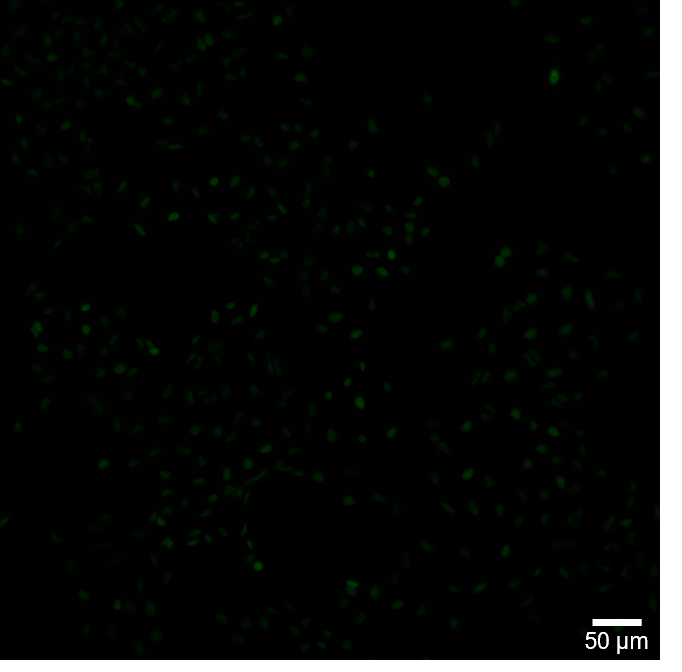

Supplement: Supplementary file 9 — Source Data for Figure 6 [file EMMM-15-e17836-s003.zip › Figure_6/Fig_6G/A549_A485.tif]

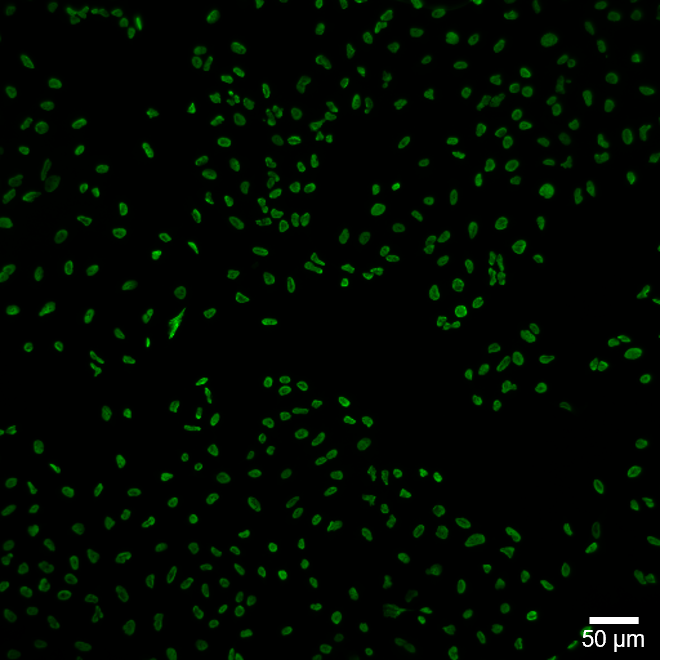

Supplement: Supplementary file 9 — Source Data for Figure 6 [file EMMM-15-e17836-s003.zip › Figure_6/Fig_6G/A549_A485_SP.tif]

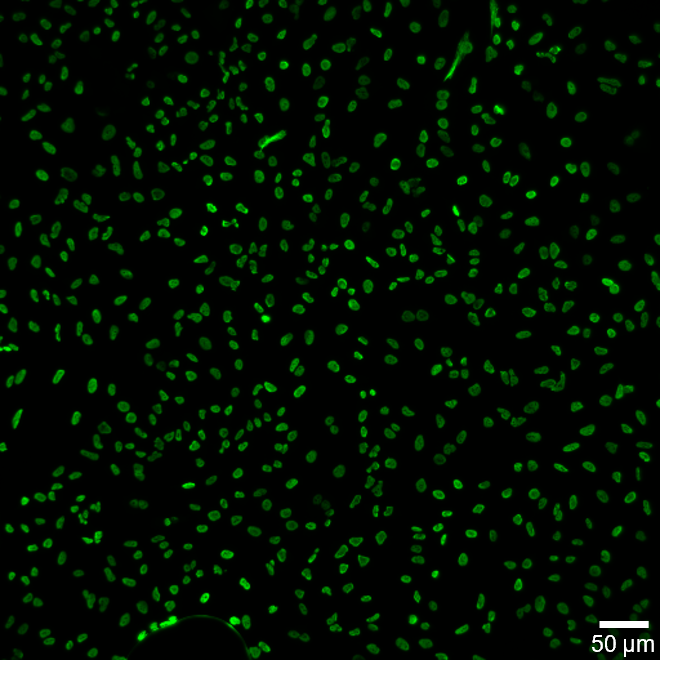

Supplement: Supplementary file 9 — Source Data for Figure 6 [file EMMM-15-e17836-s003.zip › Figure_6/Fig_6G/A549_Control.tif]

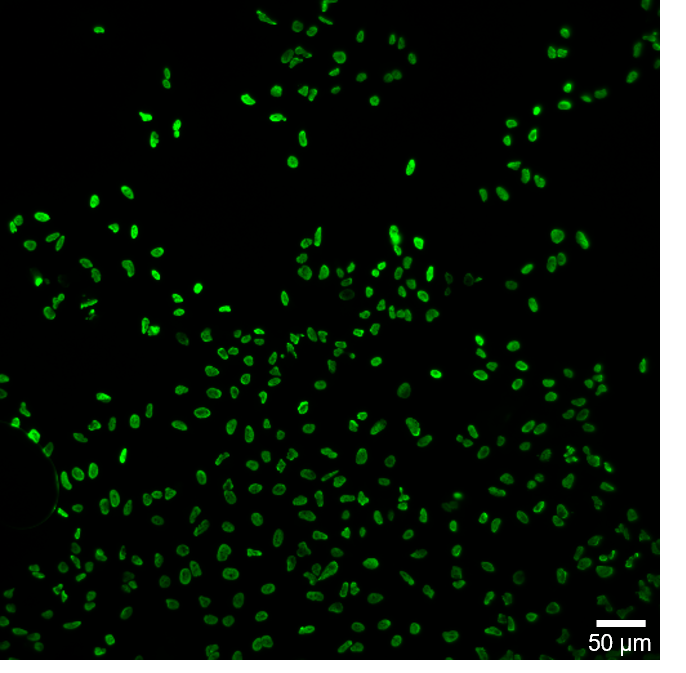

Supplement: Supplementary file 9 — Source Data for Figure 6 [file EMMM-15-e17836-s003.zip › Figure_6/Fig_6G/A549_SP.tif]
