# Supplementary material for: Propionate reinforces epithelial identity and reduces aggressiveness of lung carcinoma
Source: EMBO Mol Med. 2023 Sep 28;15(12):e17836. doi: 10.15252/emmm.202317836 (PMC10701619; doi:10.15252/emmm.202317836)
Supplement: Supplementary file 11 [file EMMM-15-e17836-s005.pdf]

EMBO Molecular Medicine

Propionate reinforces epithelial identity and reduces aggressiveness of lung carcinoma

Vignesh Ramesh, Paradesi Gollavilli, Luisa Pinna, Mohammad Aarif Siddiqui, Adriana Turtos, Francesca Napoli, Yasmin Antonelli, Aldo Leal-Egaña, Jesper Havelund, Simon Jakobsen, Elisa Boiteux, Marco Volante, Nils Færgeman, Ole Jensen, Rasmus Siersbaek, Kumar Somyajit, and Paolo Ceppi
DOI: 10.15252/emmm.202317836

Corresponding author: Paolo Ceppi (pceppi@bmb.sdu.dk)

Review Timeline:

Submission Date:	11th Apr 23
Editorial Decision:	5th May 23
Revision Received:	3rd Aug 23
Editorial Decision:	31st Aug 23
Revision Received:	5th Sep 23
Accepted:	7th Sep 23

Editor: Lise Roth

Transaction Report:

(Note: With the exception of the correction of typographical or spelling errors that could be a source of ambiguity, letters and reports are not edited. Depending on transfer agreements, referee reports obtained elsewhere may or may not be included in this compilation. Referee reports are anonymous unless the Referee chooses to sign their reports.)

5th May 2023

Dear Dr. Ceppi,

Thank you for the submission of your manuscript to EMBO Molecular Medicine. We have now received feedback from the three reviewers who agreed to evaluate your manuscript. As you will see from the reports below, the referees acknowledge the interest of the study and are overall supporting publication of your work pending appropriate revisions.

Addressing the reviewers' concerns in full will be necessary for further considering the manuscript in our journal, and acceptance of the manuscript will entail a second round of review. EMBO Molecular Medicine encourages a single round of revision only and therefore, acceptance or rejection of the manuscript will depend on the completeness of your responses included in the next, final version of the manuscript. For this reason, and to save you from any frustrations in the end, I would strongly advise against returning an incomplete revision.

If you would like to discuss further the points raised by the referees, I am available to do so via email or video. Let me know if you are interested in this option.

Revised manuscripts should be submitted within three months of a request for revision; they will otherwise be treated as new submissions, except under exceptional circumstances in which a short extension is obtained from the editor.

When submitting your revised manuscript, please carefully review the instructions that follow below. We perform an initial quality control of all revised manuscripts before re-review; failure to include requested items will delay the evaluation of your revision.

We require:

1) A .docx formatted version of the manuscript text (including legends for main figures, EV figures and tables). Please make sure that the changes are highlighted to be clearly visible.

2) Individual production quality figure files as .eps, .tif, .jpg (one file per figure). For guidance, download the 'Figure Guide PDF' (https://www.embopress.org/page/journal/17574684/authorguide#figureformat).

3) At EMBO Press we ask authors to provide source data for the main figures. Our source data coordinator will contact you to discuss which figure panels we would need source data for and will also provide you with helpful tips on how to upload and organize the files.

4) A .docx formatted letter INCLUDING the reviewers' reports and your detailed point-by-point responses to their comments. As part of the EMBO Press transparent editorial process, the point-by-point response is part of the Review Process File (RPF), which will be published alongside your paper.

5) A complete author checklist, which you can download from our author guidelines (https://www.embopress.org/page/journal/17574684/authorguide#submissionofrevisions). Please insert information in the checklist that is also reflected in the manuscript. The completed author checklist will also be part of the RPF.

6) It is mandatory to include a 'Data Availability' section after the Materials and Methods. Before submitting your revision, primary datasets produced in this study need to be deposited in an appropriate public database, and the accession numbers and database listed under 'Data Availability'. Please remember to provide a reviewer password if the datasets are not yet public (see https://www.embopress.org/page/journal/17574684/authorguide#dataavailability).
Note that the Data Availability Section is restricted to new primary data that are part of this study.

7) For data quantification: please specify the name of the statistical test used to generate error bars and P values, the number (n) of independent experiments (specify technical or biological replicates) underlying each data point and the test used to calculate p-values in each figure legend. The figure legends should contain a basic description of n, P and the test applied. Graphs must include a description of the bars and the error bars (s.d., s.e.m.). Please provide exact p values.

8) Our journal encourages inclusion of *data citations in the reference list* to directly cite datasets that were re-used and obtained from public databases. Data citations in the article text are distinct from normal bibliographical citations and should directly link to the database records from which the data can be accessed. In the main text, data citations are formatted as follows: "Data ref: Smith et al, 2001" or "Data ref: NCBI Sequence Read Archive PRJNA342805, 2017". In the Reference list, data citations must be labeled with "[DATASET]". A data reference must provide the database name, accession number/identifiers and a resolvable link to the landing page from which the data can be accessed at the end of the reference. Further instructions are available at .

9) We replaced Supplementary Information with Expanded View (EV) Figures and Tables that are collapsible/expandable online. A maximum of 5 EV Figures can be typeset. EV Figures should be cited as 'Figure EV1, Figure EV2" etc... in the text and their respective legends should be included in the main text after the legends of regular figures.
- For the figures that you do NOT wish to display as Expanded View figures, they should be bundled together with their legends in a single PDF file called *Appendix*, which should start with a short Table of Content. Appendix figures should be referred to in the main text as: "Appendix Figure S1, Appendix Figure S2" etc.
- Additional Tables/Datasets should be labeled and referred to as Table EV1, Dataset EV1, etc. Legends have to be provided in a separate tab in case of .xls files. Alternatively, the legend can be supplied as a separate text file (README) and zipped together with the Table/Dataset file.
See detailed instructions here:

10) For more information: There is space at the end of each article to list relevant web links for further consultation by our readers. Could you identify some relevant ones and provide such information as well? Some examples are patient associations, relevant databases, OMIM/proteins/genes links, author's websites, etc...

11) Author contributions: CRediT has replaced the traditional author contributions section because it offers a systematic machine readable author contributions format that allows for more effective research assessment. Please remove the Authors Contributions from the manuscript and use the free text boxes beneath each contributing author's name in our system to add specific details on the author's contribution. More information is available in our guide to authors.

12) Disclosure statement and competing interests: We updated our journal's competing interests policy in January 2022 and request authors to consider both actual and perceived competing interests. Please review the policy https://www.embopress.org/competing-interests and update your competing interests if necessary.

13) Every published paper now includes a 'Synopsis' to further enhance discoverability. Synopses are displayed on the journal webpage and are freely accessible to all readers. They include a short stand first (maximum of 300 characters, including space) as well as 2-5 one-sentences bullet points that summarizes the paper. Please write the bullet points to summarize the key NEW findings. They should be designed to be complementary to the abstract - i.e. not repeat the same text. We encourage inclusion of key acronyms and quantitative information (maximum of 30 words / bullet point). Please use the passive voice. Please attach these in a separate file or send them by email, we will incorporate them accordingly.

Please also suggest a striking image or visual abstract to illustrate your article as a PNG file 550 px wide x 300-600 px high.

14) As part of the EMBO Publications transparent editorial process initiative (see our Editorial at http://embomolmed.embopress.org/content/2/9/329), EMBO Molecular Medicine will publish online a Review Process File (RPF) to accompany accepted manuscripts.
In the event of acceptance, this file will be published in conjunction with your paper and will include the anonymous referee reports, your point-by-point response and all pertinent correspondence relating to the manuscript. Let us know whether you agree with the publication of the RPF and as here, if you want to remove or not any figures from it prior to publication.
Please note that the Authors checklist will be published at the end of the RPF.

EMBO Molecular Medicine has a "scooping protection" policy, whereby similar findings that are published by others during review or revision are not a criterion for rejection. Should you decide to submit a revised version, I do ask that you get in touch after three months if you have not completed it, to update us on the status.

I look forward to receiving your revised manuscript.

With kind regards,

Lise

Lise Roth, PhD
Senior Editor
EMBO Molecular Medicine

***** Reviewer's comments *****

Referee #1 (Comments on Novelty/Model System for Author):

Adequate experimental processes

Referee #1 (Remarks for Author):

Review of the manuscript entitled " Propionate reinforces epithelial identity and reduces
aggressiveness of lung carcinoma" authored by Vignesh Ramesh et al.

Herein authors identify an inverse association between short-chain fatty acids, mainly propionate and butanoate, and the EMT program in non-small cell lung cancer (NSCLC) in patients.

By mean of an ample set of experimental approaches, including in vitro experiments -with NSCLC cells - where propionate promotes epithelial transcriptional program and reduces aggressiveness and chemo-resistant EMT phenotype-; and in vivo preclinical models - where propionate decreases the metastatic potential and lymph node spread in nude mice and a genetic NSCLC mouse model, respectively-; the authors nicely highlight the therapeutic potential of propionate administration in reducing NSCLC aggressiveness which merit further investigation in cancer patients.

Interestingly, they reveal that propionate treatment induces a chromatin remodelling epigenetic modification -H3K27 acetylation in the CDH1 promoter- as underlying mechanism, at least, partially implicated in the epithelial reversion from EMT.

Using bioinformatics approaches and public data bases, authors find that low levels of propionate correlate with poor survival outcomes in NSCLC cancer patients. Thus, they identify a SCFA network -mainly propionate- which may be promising to prevent NSCLC recurrence.

In general, I find the findings interesting and that could be relevant and valuable in a clinical setting.

Overall, the results are clear, and the manuscript is well organized and well written.
Conclusions are supported by the experimental results where propionate may be proposed as a complementary treatment to avoid tumor recurrence.

Nevertheless, there are minor concerns that should be addressed.

.-Please, indicate why the NSCLC cell lines NCI-H358, HCC827, and A549 are selected for the in vitro experiments.

.-Being metabolic reprogramming one of the hallmarks of cancer, it should be desirable to demonstrate the effect of propionate treatment in cell bioenergetics (oxidative phosphorylation or aerobic glycolysis). Does propionate promote oxphos vs aerobic glycolysis? Does propionate promote increased oxphos with increase on ROS from mitochondria? I am curious if scavenging ROS revert the effect of propionate.

.-Authors indicate propionate pretreatment sensitizes NSCLC cells to cisplatin. Due to the relevance of immunotherapy in lung cancer, is there any link between SCFA and clinical benefits of immune checkpoint inhibitors. This should be discussed in the discussion section.

- It should be interesting to include a final paragraph highlighting the relevance of modifiable risks factors associated to lifestyle, such as diet -(Fibre-microbiome-SCFA) and exercise (that affect systemic lipid metabolism homeostasis, inducing lipolysis to release FAs and Ac-CoA through -oxidation. Thus, Ac-CoA may be available for the synthesis of ketone bodies -such as propionate, acetoacetate and β-OHB- in the liver, being systemically available for other tissues.

Referee #2 (Comments on Novelty/Model System for Author):

Model systems could be improved, as detailed in the remarks.

Referee #2 (Remarks for Author):

In this report, authors describe a new role for short-chain fatty acids in supporting the differentiated epithelial phenotype and

opposing EMT/mesenchymal phenotype in lung carcinoma cells. They found that propionate reduced invasiveness and resistance in vitro in lung cancer cell lines and impacted lymph node invasion and metastasis in two mice models. Finally, they identify H3K27 acetylation by p300 as the mediator associated with propionate signaling, chromatin remodeling and EMT suppression. These results are new and significant. EMT pathways controls cell plasticity, an essential feature of cancer cells with a strong clinical relevance. Numerous pathways can induce or inhibit EMT pathways, they typically trigger several families of transcriptional factors involved in all described in vivo EMT situations during development. Chromatin reorganization is also a typical feature of the EMT process, leading to the activation or repression of target genes.

Overall, the manuscript provides new and relevant information concerning lung carcinoma progression. The novelty in this report is the EMT emphasis in vitro and in vivo and the intriguing link to chromatin conformation. These questions are pertinent and important for all cancer researchers. However, several points should be answered to support the strength of the results and conclusions.

1) Literature
Interactions between fatty acid and EMT pathways represent a significant and intriguing new aspect of epithelial differentiation. They have been studied by several authors not cited in this report. In lung cancer, acetate was surprisingly described to inhibit TGFb -induced EMT in the same cell line A549 used in this report (Lyu et al MBC 2022 doi. 10.1091/mbc.E22-02-0066). In different context, palmitate induces EMT in hepatocellular carcinoma (Nath et al Sci Rep, 2015. doi.org/10.1038/srep14752) . Also, fatty acid oxydation has been shown to be critical for endothelial-mesenchymal transition in heart valves (Xiong et al Mol Cell 2018, doi.org/10.1016/j.molcel.2018.01.010. These results should be mentioned in the manuscript.

2) Some problems are linked to the western blotting results shown in Fig 2 and 5. On Fig 2, Ecad expression level appears to be upregulated after 48-72h on the Western blot, even when no treatment is applied. Ecad expression level after 72h without treatment is above the level seen after 24h with 5mM SP, or after 48h with 1 mM SP. Similar range is seen with SB. This suggests that A549 cells modulate their epithelial differentiation with culture time with or without treatment with a comparable extent. Why is Ecad upregulated? On Fig 5, we see the reverse: Ecad expression level appears to be downregulated with time after 3 days. In both cases, Western quantification is missing and it is not clear how many duplicate experiments were performed.

3) All EMT cases during development involve at least one member from the EMT associated trancription factor families (EMT-TF) Snail, Zeb and Twist. However, these genes also have some functions unrelated to the classic EMT process, for example in fibroblast and immune cells. It would appear important to better define putative interactions between SCFA and EMT master genes pathways. Can the SCFA "shortcut" the EMT-TF pathways to target HDAC and impact chromatin configuration? This is suggested by the authors ("SP preferentially promotes an epithelial-specific gene expression program through epigenetic reprogramming") but not supported by data. Results obtained with Zeb1 are rather confusing. Expression pattern of all three families EMT-TF should be gathered to evaluate their potential involvement.

4) Authors suggest that SCFA impact on epithelial cell differentiation could represent a general mechanism involving EMT processing (l390:" The effect appears to be a general phenomenon"), but at the same time they mention a specific targeting of " the pulmonary alveolar type-II cell-type markers implying the establishment of epithelial integrity in lungs" (l392) by the propionate treatment that would increase specifically the airway epithelial cell-type genes expression. The text should be consistent and make a stand, mentioning distinct hypothesis. It would be helpful to check how treatment can impact some other markers for lung epithelial cell progenitors (stemness), also linked to EMT pathways

5) Mouse models support the authors finding but do not demonstrate any EMT involvement. The expression of EMT features in the tumors before and after propionate treatment should be checked by classic immunolocalization (or any other approach) to estimate the level of cell depolarization, dissociation and expression of EMT functional markers.

6) In Fig 2. wound healing experiments are conclusive in showing a delay in cell sheet spreading after SP treatment, but they do not indicate a migration halt. It would be important to look at higher magnification to check if the cells are individualized or migrate as an epithelial sheet. Also, the number of repeat experiments should be mentioned.

7) To study cell plasticity, an essential feature of EMT, it would be important to insert A549 spheroids into 3D collagen gels to monitor and quantify migration and invasive pattern, with or without treatment.

Minor points

1) In Fig 1F, "Propanoate" is used for propionate found everywhere else in the manuscript. One word should be consistently used.

2) It is not clear what should be seen in Fig 3i. A stronger histological staining and additional higher magnification pictures would be helpful.

3) Fig3H. It is not clear how many mice were used for this survival study.

4) Fig 5E higher magnification pictures would be useful to assert ZO-1 and Epcam localization and organization pattern.

5) Discussion is overall too long and redundant. It should be more focused on this manuscript findings. For example, the section l409-l426 about SP and cisplatin resistance is quite interesting, but does not imply EMT pathways. Similarly, the section about SCFA availability (l428-l445) is rather unclear.

6) In Fig S1, Zeb2 and Axl are found to be downregulated by TGFb treatment. This is not the consensus response described in other reports studying EMT pathways. Any comments?

7) It should be mentioned that most cancer data sets used in this study involve a mix of stroma and tumor cells, an element to consider in the discussion.

8) The statement l409." Our lung experimental metastasis model showed that SP can inhibit metastasis." is too strong. SP treatment has an impact, but does not appear to inhibit metastasis overall.

Referee #3 (Remarks for Author):

The epithelial-to-mesenchymal transition (EMT) is a cellular program that cancer cells can utilize to gain metastatic ability. However, targeting the EMT can be tricky since its regulation is primarily mediated by transcription factors, which are challenging to inhibit. In contrast, metabolic enzymes may represent promising druggable targets for inhibiting EMT. Therefore, investigating the role of metabolic processes in the EMT program could lead to identifying new drug targets.
In their study, Ramesh et al. aimed to uncover unknown metabolic processes involved in the EMT program through transcriptome analysis. Their findings revealed that the propionate and butanoate metabolism gene set negatively correlated with the EMT gene set. Further experiments demonstrated that treatment with propionate and butanoate induced the cells to acquire more epithelial characteristics. Additionally, propionate inhibited metastasis and sensitized the cells to cisplatin. Finally, they show that propionate reduces lung cancer EMT by broad chromatin remodeling. However, all of the experiments were conducted by treating epithelial cells with fatty acids, and the role of the endogenous propionate and butanoate in the EMT program is unclear. Additionally, the effect of these fatty acids on mesenchymal-like lung cancer cells needs to be investigated. Thus, the role of endogenous propionate and butanoate in EMT program regulation remains unclear and deserves investigation.

Major comments
1. The authors suggest that the short-chain fatty acids induce the cells to become more epithelial. For example, in Figure 2, they demonstrate that treatment of A549, SK-MES-1, and NCI-H23 cells with propionate and butanoate induces the cells to gain more epithelial characteristics. However, the biological meaning of this finding is unclear as these cells are already in the epithelial state. Therefore, these experiments should also be conducted in lung cancer cell lines in a partially mesenchymal state. Specifically, these experiments will determine whether these short-chain fatty acids can induce the MET program.
2. The authors analyzed gene expression databases to identify the metabolic processes. They assumed that short-chain fatty acids are regulated at the transcription level. However, they did not validate or elaborate on this analysis. For example, which enzymes in the short-chain fatty acid biosynthesis pathway are downregulated during the EMT program or in epithelial vs. mesenchymal cells? What will be the outcome of their ectopic expression in epithelial and mesenchymal cells? What will be the outcome of manipulating the enzymes synthesizing these fatty acids? Will the cells induce the EMT program?
3. One of the main themes in this manuscript is that the short-chain fatty acids function as inhibitors of the EMT. However, the physiological relevance of these findings is still missing as they did not validate any changes in the fatty acid intracellular levels. For example, does the intracellular level of propionate and butanoate reduce during the EMT?
4. The role of these short-chain fatty acids as regular of the EMT program is needed to be elaborated. Indeed, Figures 2H-I show that SP and SB inhibit EMT markers expression upon TGFß treatment. However, this was demonstrated only on two markers which is insufficient to conclude. Additionally, showing changes in the marker's expression is not enough to conclude any effect on the EMT program, as the effect on the cell's migratory capabilities is needed to be examined.
Minor comments
1. The HR (hazard ratio) value is absent from all of the Kaplan-Meier plots that pertain to survival rates.
2. In Figures 2B and 2C, why do E-cadherin levels change over time in samples without the short-fatty acids treatment? Additionally, the ZEB1 blot is problematic. In Figure 2C treatment, its expression levels don't change, and in Figure 2B, there is inconsistency with SP doses. Additional mesenchymal markers are needed to clarify this point.

Response to the Reviewers Comments:

We thank the Editor and Reviewers for the overall positive comments and their important suggestions, which improved the manuscript's content and readability. We have now addressed all the suggestions by highlighting the text with major changes.

Referee #1 (Comments on Novelty/Model System for Author):

Adequate experimental processes

Referee #1 (Remarks for Author):

Review of the manuscript entitled " Propionate reinforces epithelial identity and reduces aggressiveness of lung carcinoma" authored by Vignesh Ramesh et al.

Herein authors identify an inverse association between short-chain fatty acids, mainly propionate and butanoate, and the EMT program in non-small cell lung cancer (NSCLC) in patients.

By mean of an ample set of experimental approaches, including in vitro experiments -with NSCLC cells - where propionate promotes epithelial transcriptional program and reduces aggressiveness and chemo-resistant EMT phenotype-; and in vivo preclinical models - where propionate decreases the metastatic potential and lymph node spread in nude mice and a genetic NSCLC mouse model, respectively-; the authors nicely highlight the therapeutic potential of propionate administration in reducing NSCLC aggressiveness which merit further investigation in cancer patients.

Interestingly, they reveal that propionate treatment induces a chromatin remodelling epigenetic modification -H3K27 acetylation in the CDH1 promoter- as underlying mechanism, at least, partially implicated in the epithelial reversion from EMT.

Using bioinformatics approaches and public data bases, authors find that low levels of propionate correlate with poor survival outcomes in NSCLC cancer patients. Thus, they identify a SCFA network -mainly propionate- which may be promising to prevent NSCLC recurrence.

In general, I find the findings interesting and that could be relevant and valuable in a clinical setting.

Overall, the results are clear, and the manuscript is well organized and well written. Conclusions are supported by the experimental results where propionate may be proposed as a complementary treatment to avoid tumor recurrence.

Nevertheless, there are minor concerns that should be addressed.

1. Please, indicate why the NSCLC cell lines NCI-H358, HCC827, and A549 are selected for the in vitro experiments.

Response R1.1 We obtained the gene expression profiles of three NSCLC cell lines mentioned by the Reviewer (NCI-H358, HCC827 and A549) treated with TGF-β from a publicly available database (GEO – GSE49644) as part of a validation of the EMT gene signature obtained from Mak et al., 2016 employed in the current study (**Appendix Fig S1A-C, Appendix Table S2**).

For the further in vitro validation, A549, SKMES1, NCI-H520, NCI-H23, CALU-1 and H1299 were used to substantiate and study the molecular level processes of SCFA in lung cancer. These cell lines were used because they represent the two main NSCLC histotypes, adenocarcinoma and squamous cell carcinoma. This has now been better highlighted in the revised version of the manuscript (Page: 7; Line: 156-173).

2. Being metabolic reprogramming one of the hallmarks of cancer, it should be desirable to demonstrate the effect of propionate treatment in cell bioenergetics (oxidative phosphorylation or aerobic glycolysis). Does propionate promote oxphos vs aerobic glycolysis? Does propionate promote increased oxphos with increase on ROS from mitochondria? I am curious if scavenging ROS revert the effect of propionate.

Response R1.2 This is an interesting suggestion and we have now carried out a Seahorse metabolic flux analysis to investigate cell bioenergetics upon treatment with sodium propionate (SP). A549 cells were treated with SP 5mM for 24 hours, and OCR (oxygen consumption rate) and ECAR (extracellular acidification rate) were analyzed. Interestingly, we found propionate did not increase but rather slightly decreased ATP-linked respiration compared to the control (**Fig R1A**), while we did not observe changes in ECAR activity (**Fig R1B**). Previous studies showed that treatment of HCT116 (colorectal cancer) showed an increase in the OCR although minimally at 10 mM propionate concentration (Høgh *et al*, 2020). In another study, treatment of primary human epidermal keratinocytes (HEK) with butyrate showed a significant reduction in the basal respiration and maximal respiratory capacity (Trompette *et al*, 2022). We therefore believe the response to propionate could be cell line- and tissue-specific.

Further, we treated A549 cells with SP for 72 hours followed by the measurement of reactive oxygen species level using flow cytometry (DCFDA/H2DCFDA assay). Again, we did not observe any significant increase in the ROS levels (**Fig R1C**). Cisplatin was used as a positive control for the ROS detection. Thus, ROS is not majorly altered with propionate treatment, and therefore we did not pursue the suggested scavenging experiment. We decided to not add these data to the final version of the manuscript for space limitation. Thanks for the suggestion.

Figure R1

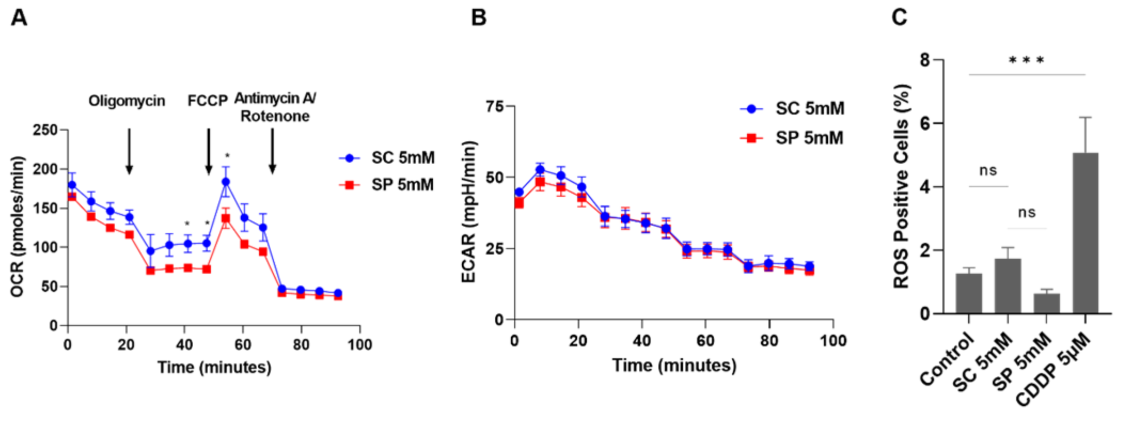

Figure R1. Oxidative phosphorylation and aerobic glycolysis analysis of A549 cells treated with SP. A, B. A549 cells were treated with SP (5 mM) for 24 hours followed by seahorse analysis with injection of oligomycin (1 μM), FCCP (1 μM) and Antimycin A and rotenone (1 μM). C. ROS measurement in A549 cells treated with SP (5 mM) for 72 hours. ns – not significant. *** - $p<0.001$.

3. Authors indicate propionate pretreatment sensitizes NSCLC cells to cisplatin. Due to the relevance of immunotherapy in lung cancer, is there any link between SCFA and clinical benefits of immune checkpoint inhibitors. This should be discussed in the discussion section.

Response R1.3 This is an important suggestion. In fact, we already pointed out the importance of the positive link between SCFAs and the clinical benefits of immune checkpoint inhibitors in the discussion of the previous version. For instance, *"Recently, gut microbiota metabolomics of NSCLC patients undergoing immunotherapy with nivolumab showed that long-term responders were majorly characterized with the presence of SCFA metabolites (propionic, butyric, acetic and valeric acids) with beneficial effects (Botticelli et al, 2020)"* (Page: 21; Line: 516-519). However, we have also included few additional references in the discussion to strengthen the importance of these SCFAs in aiding therapeutic efficacy by sensitizing NSCLC to immunotherapy. For example, *"Again NSCLC patients responding to programmed cell death protein 1 (PD-1) antibody therapy were found to have higher baseline levels of fecal SCFAs concentration (Zizzari et al, 2020). Similarly, immunotherapy for several solid cancers including lung adenocarcinoma with immune checkpoint inhibitors (nivolumab or pembrolizumab) exhibiting improved PD-1 inhibitor efficacy showed high baseline fecal concentrations of SCFAs mainly propionic, butyric, valeric and acetic acid with longer progression-free survival (Nomura et al, 2020)"* (Page: 21; Line: 520-526). All these reports support the notion that SCFAs could improve the therapeutic efficacy of primary line treatment options.

4. It should be interesting to include a final paragraph highlighting the relevance of modifiable risks factors associated to lifestyle, such as diet -(Fibre-microbiome-SCFA) and exercise (that affect systemic lipid metabolism homeostasis, inducing lipolysis to release FAs and Ac-CoA through β-oxidation. Thus, Ac-CoA may be available for the synthesis of ketone bodies -such as propionate, acetoacetate and β-OHB- in the liver, being systemically available for other tissues.

Response R1.4 We agree with this view and thank the Reviewer for highlighting the requirement of dietary and physical activity as important factors in cancer mitigation. This is an important suggestion, with many potential beneficial implications for the patients. We have now included these modifiable factors in the discussion of the revised manuscript as follows:

"Diet is intimately connected with the microbial community (diversity and abundance) in humans especially with the gut microbiota, and dietary changes could contribute to the microbial profiles during diseased conditions. Epidemiological data shows that high-fiber diets are related to a lower cancer incidence and the anti-cancer effect is significantly due to the formation of SCFAs from high-fiber diet (Mirzaei et al, 2021). It has been shown that mice fed with probiotic supplementation increased the SCFA producing bacteria (propionate and butyrate) in the gut with the inhibition of lung metastasis of melanoma cells (Chen et al, 2021) implicating diet-microbiome-SCFA link" (Page: 22; Line: 530-537).

"Similarly, evidences showed that physical activity such as exercise can alter the composition of the gut microbiota, and eventually increase the SCFA production as an additional substrate for metabolism (Song & Chan, 2019)" (Page: 22; Line: 538-540).

"Since propionate is a biotic compound and a safe food ingredient with therapeutic ability (Rangan & Mondino, 2022), the beneficial effects reported here make propionate a promising avenue for further clinical exploration as a treatment option for NSCLC patients. Alternative

strategies like consuming fiber-enriched diet and physical activities resulting in high SCFA content in the system could offer a realistic approach in cancer prevention and treatment" (Page: 23; Line: 556-561).

Referee #2 (Comments on Novelty/Model System for Author):

Model systems could be improved, as detailed in the remarks.

Referee #2 (Remarks for Author):

In this report, authors describe a new role for short-chain fatty acids in supporting the differentiated epithelial phenotype and opposing EMT/mesenchymal phenotype in lung carcinoma cells. They found that propionate reduced invasiveness and resistance in vitro in lung cancer cell lines and impacted lymph node invasion and metastasis in two mice models. Finally, they identify H3K27 acetylation by p300 as the mediator associated with propionate signaling, chromatin remodeling and EMT suppression. These results are new and significant. EMT pathways controls cell plasticity, an essential feature of cancer cells with a strong clinical relevance. Numerous pathways can induce or inhibit EMT pathways, they typically trigger several families of transcriptional factors involved in all described in vivo EMT situations during development. Chromatin reorganization is also a typical feature of the EMT process, leading to the activation or repression of target genes.

Overall, the manuscript provides new and relevant information concerning lung carcinoma progression. The novelty in this report is the EMT emphasis in vitro and in vivo and the intriguing link to chromatin conformation. These questions are pertinent and important for all cancer researchers. However, several points should be answered to support the strength of the results and conclusions.

1. Literature: Interactions between fatty acid and EMT pathways represent a significant and intriguing new aspect of epithelial differentiation. They have been studied by several authors not cited in this report. In lung cancer, acetate was surprisingly described to inhibit TGFb -induced EMT in the same cell line A549 used in this report (Lyu et al MBC 2022 doi. 10.1091/mbc.E22-02-0066). In different context, palmitate induces EMT in hepatocellular carcinoma (Nath et al Sci Rep, 2015. doi.org/10.1038/srep14752) . Also, fatty acid oxydation has been shown to be critical for endothelial-mesenchymal transition in heart valves (Xiong et al Mol Cell 2018, doi.org/10.1016/j.molcel.2018.01.010. These results should be mentioned in the manuscript.

Response R2.1 As suggested by the reviewer, we have included the mentioned articles in the discussion of the current revised manuscript as follows:

1. *"Previous study showed that acetate could inhibit TGFβ1-induced EMT features (Lyu et al, 2022). However, in our study, we did not find any modulation in E-cadherin or ZEB1 levels upon sodium acetate treatment. Further, the study utilized 40 mM of sodium acetate to inhibit TGFβ1-induced EMT features which is 40-fold higher concentration than used in the current study. Therefore, acetate's role in inhibiting EMT in lung cancer cannot be ruled out as higher concentration could have an impact in inhibiting EMT in lung cancer cells potentially through a similar mechanism of propionate and butanoate"* (Page: 20; Line: 495-502). We thank the reviewer for highlighting this important reference in the current study.

2. "*Elevation in the long-chain free fatty acid palmitate has been shown to promote EMT in hepatocellular carcinoma via Wnt/β-catenin and TGF-β signaling (Nath et al, 2015)"* (Page: 17; Line: 415-417).

3. *"On the other hand, a decrease in the fatty acid oxidation was found during endothelial-mesenchymal transition by altering the intracellular levels of acetyl-coA (Xiong et al, 2018)"* (Page: 17; Line: 417-419).

2. Some problems are linked to the western blotting results shown in Fig 2 and 5. On Fig 2, Ecad expression level appears to be upregulated after 48-72h on the Western blot, even when no treatment is applied. Ecad expression level after 72h without treatment is above the level seen after 24h with 5mM SP, or after 48h with 1 mM SP. Similar range is seen with SB. This suggests that A549 cells modulate their epithelial differentiation with culture time with or without treatment with a comparable extent. Why is Ecad upregulated? On Fig 5, we see the reverse: Ecad expression level appears to be downregulated with time after 3 days. In both cases, Western quantification is missing and it is not clear how many duplicate experiments were performed.

Response R2.2 This is an important observation, which gives us opportunity to explain. Cells in **Fig 2A-C** were harvested from low to high confluence every 24h until 72h and show quite consistently an increase in the E-cadherin levels in a time-dependent manner without treatment, as noted, primarily due to the increase in the cell-cell contact establishment during the confluence attainment. We therefore consider changes in the E-cadherin levels and EMT markers by SP or SB with respect to the control of the specific time point. By contrast, in **Fig 5A**, we did not observe any up-regulation of E-cadherin over 12 days treatment in the controls. However, for this long-term experiment the cells were split every 3 days using 0.5 mM EDTA with the confluence of around 90-95%, and re-seeded for propagation in a split ratio of 1:3 to 1:5 with the SP treatment. This was necessary because E-cadherin is degraded with the use of trypsin, but not with EDTA. So, the experiment in **Fig 5A** is not directly comparable to those in **Fig 2**, and the observed difference in the level of E-cadherin between 3, 6, 9 and 12 days is most likely due to variability in the confluence during harvesting. Again, we emphasize that the increase in E-cadherin is primarily compared with the control at that specific time point to highlight the reinforcement of E-cadherin or inhibition of EMT, and that the treatment itself does not alter the confluency. We detailed the experimental procedure of EDTA-based harvesting of cells in the method section (Page: 25; Line: 598-607) and discussed about this in the revised manuscript (Page: 18; Line: 425-429).

Moreover, as requested by the Reviewer, we have included the Image J quantifications for the figures (**Figs 2B, 2C** and **5A**) and the level of E-cadherin or ZEB1 is compared to the respective time point controls after normalizing to β-Actin (**Figs R2 and R3**), and quantifications have been added as fold changes in the revised manuscript (**Fig 2B, 2C, 5A**).

To further confirm the increase of E-cadherin with SP treatment, we performed q-PCR analysis of E-cadherin in A549 cells treated with SP 5mM for 72h and found a similar level of alterations at the transcript level (**Fig R4).** The result is included in the revised manuscript (Page: 8; Line: 180-182 and **Fig EV1G**). This indicates the robustness of the E-Cadherin increase induced by propionate with an independent method.

Regarding the repeats, all the western blots in the manuscript were done at least 3 independent times, as shown below for these specific experiments (**Fig R2 and R3**) and the legend has been

now updated with the number of repeats performed in the revised manuscript (Page: 57; Line: 1458-1460).

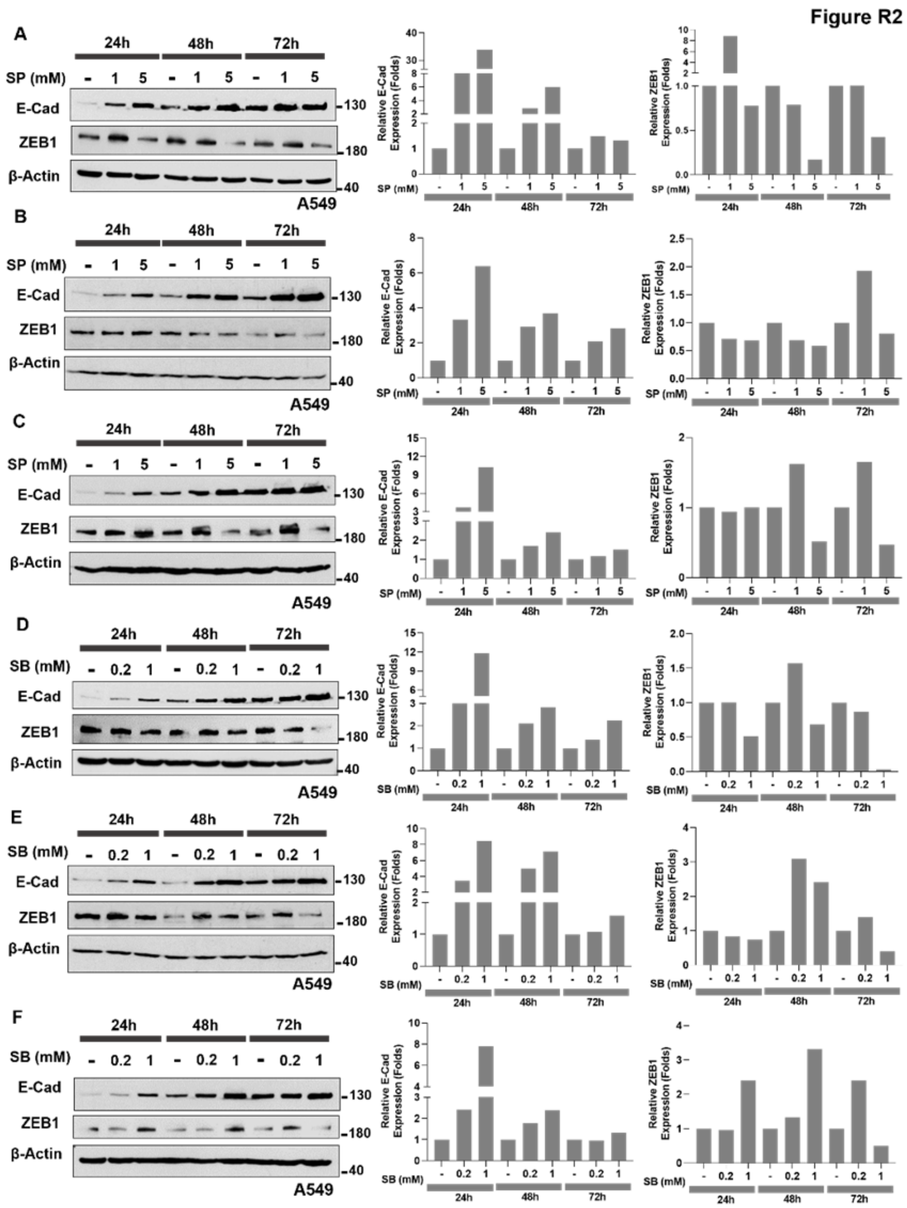

Figure R2. In vitro treatment effect of SCFAs, propionate or butanoate, on EMT markers gene expression. A-F. Western blot analysis of E-cadherin and ZEB1 protein levels in A549 cells treated with sodium propionate (SP) (A-C) or sodium butanoate (SB) (D-F) in the indicated dose- and time-dependent manner for 3 independent times. Left panel shows the blot while middle and right panel shows the quantification for E-Cadherin and ZEB1 levels, respectively. β-Actin was used as an internal control and used it to normalize the protein levels of EMT markers. Relative expression is indicated in comparison to the control of respective time points.

Figure R3

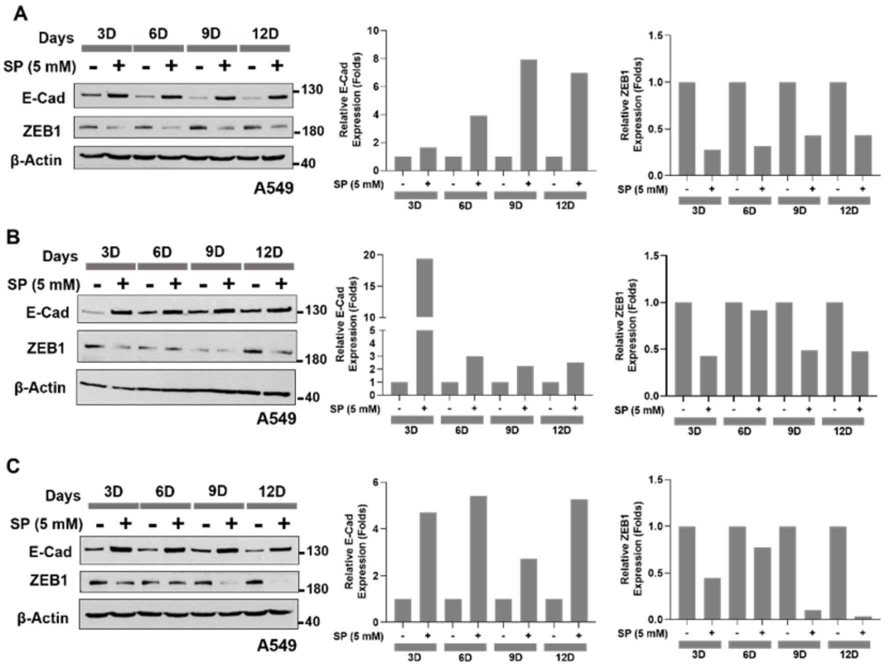

Figure R3. In vitro treatment effect of propionate for 12 days on the EMT markers gene expression. A-C. Western blot analysis of E-cadherin and ZEB1 protein expression in time series in A549 cell line treated with sodium propionate (SP) at 5 mM for 12 days. The experiment was performed for 3 independent times. Left panel shows the blot while middle and right panel shows the quantification for E-cadherin and ZEB1 levels, respectively. β-Actin was used as an internal control and used to normalize the protein levels. Relative expression is indicated in comparison to the control of respective time points.

Figure R4

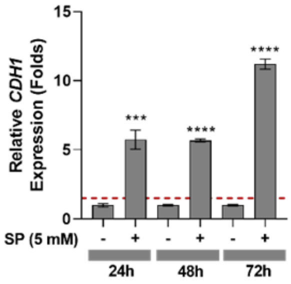

Figure R4. In vitro treatment effect of propionate for 3 days on the E-cadherin gene expression. Real-time quantitative PCR analysis of epithelial gene (*CDH1*) in A549 cell line treated with sodium propionate (SP) at 5 mM for 3 days. *GAPDH* was used as an internal control. Red dotted line represents the fold change cut-off at 1.5. Significance was calculated using unpaired t-test. *** - $p<0.001$, **** - $p<0.0001$.

3. All EMT cases during development involve at least one member from the EMT associated transcription factor families (EMT-TF) Snail, Zeb and Twist. However, these genes also have some functions unrelated to the classic EMT process, for example in fibroblast and immune cells. It would appear important to better define putative interactions between SCFA and EMT master genes pathways. Can the SCFA "shortcut"

the EMT-TF pathways to target HDAC and impact chromatin configuration? This is suggested by the authors ("SP preferentially promotes an epithelial-specific gene expression program through epigenetic reprogramming") but not supported by data. Results obtained with Zeb1 are rather confusing. Expression pattern of all three families EMT-TF should be gathered to evaluate their potential involvement.

Response R2.3 This is a very good point that offers us the opportunity to clarify. As suggested by the Reviewer, we have performed western blot analysis of members from all the families of EMT-associated transcription factors (ZEB1, ZEB2, SNAIL, SLUG and TWIST1) upon SP treatment. As a result, an overall reduction of EMT-TF in the 72h time course was observed. As seen with ZEB1, in fact, there was a consistent down-regulation of TWIST1 and ZEB2, and to a minimal extent of SLUG with 72h SP treatment (**Fig R5**), while SNAIL was found up-regulated. Therefore, while our data suggest that epigenetic reprogramming may begin at an earlier phase of the SP treatment (**Fig 6D**) and its effects on EMT are rescuable with p300 inhibitors (**Fig 6E**), there could still be a possibility of parallel involvement of EMT-TFs by SP in inhibiting the EMT properties. For this reason, we decided to replace the word "preferentially" with "in part" to describe the epigenetic reprogramming of the epithelial-specific gene expression in inhibiting mesenchymal features (Page: 15; Line: 373-374) in the sentence mentioned by the Reviewer. The result is included in the revised manuscript (Page: 8; Line: 182-186; **Appendix Fig S2D**)

We agree that it would be very interesting in future to study the dynamic role of SP in epigenetic reprogramming and EMT-transcription factors modulation in inhibiting EMT. For instance, there could be a cross-regulation between these entities. In addition, it would be essential to elucidate why and how SNAIL up-regulation happens in a cellular environment that is moving towards EMT suppression (another feedback loop?). All these aspects may be object of a future dedicated investigation. This has now been included in the discussion section of the manuscript (Page: 20; Line: 491-493). Thanks a lot for the important input.

Figure R5

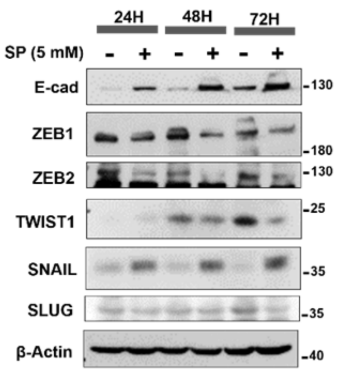

Figure R5. In vitro treatment effect of propionate for 3 days on the EMT-associated transcription factors expression. Western blot analysis of E-cadherin and EMT-transcription factors (ZEB1, ZEB2, TWIST1, SNAIL and SLUG) in A549 cells treated with sodium propionate (SP) at 5 mM in the indicated time-dependent manner. β-Actin was used as an internal control.

4. Authors suggest that SCFA impact on epithelial cell differentiation could represent a general mechanism involving EMT processing (l390:" The effect appears to be a general phenomenon"), but at the same time they mention a specific targeting of " the pulmonary alveolar type-II cell-type markers implying the establishment of epithelial integrity in lungs" (l392) by the propionate treatment that would increase specifically the airway

epithelial cell-type genes expression. The text should be consistent and make a stand, mentioning distinct hypothesis. It would be helpful to check how treatment can impact some other markers for lung epithelial cell progenitors (stemness), also linked to EMT pathways

Response R2.4 We apologize for the confusion in the text. We wanted to emphasize the fact that SP's effect could be a general phenomenon as we could observe it with other cancer cell lines such as with pancreatic cancer cell line (**Appendix Figs S2A-C**). However, specifically in the context of lung, we wanted to highlight the epithelial-inducing phenomenon of SP on the airway epithelial cell-type (pulmonary alveolar type-II cell type) which plays an important role in the alveoli integrity and function. To avoid confusion for the general readers, we have rephrased the sentences in the current revised manuscript (Page: 18; Line: 437-441) as follows:

"The epithelial reinforcement could also be observed in other cancer types such as in pancreatic cancer cell line. In the context of lung, propionate treatment increased the genes expression of pulmonary alveolar type-II cell-type implying the establishment of epithelial integrity in lungs"

As requested, we have now tested the lung epithelial cell progenitor genes expression (Rawlins, 2008) in the RNA-seq profile of 3 days and 12 days SP treated A549 cells. We observed a significant decrease in the progenitor genes expression at 12 days (**Fig R6A**). In addition, we also analyzed the expression of cancer stemness marker genes expression related to EMT (ALDH1A1 and ALDH3A1) (Ramesh *et al*, 2020; Fan *et al*, 2021). Interestingly, there was a very significant decrease in these cancer stem cell markers genes implying that propionate may also inhibit stemness (**Figs R6B-C**). Nevertheless, further experiments are needed in future to understand the mechanistic basis of propionate in inhibiting the stemness property of lung cancer. The results are included in the revised manuscript (Page: 13; Line: 318-322; **Fig 5K**, **Figs EV3F** and **EV3G**).

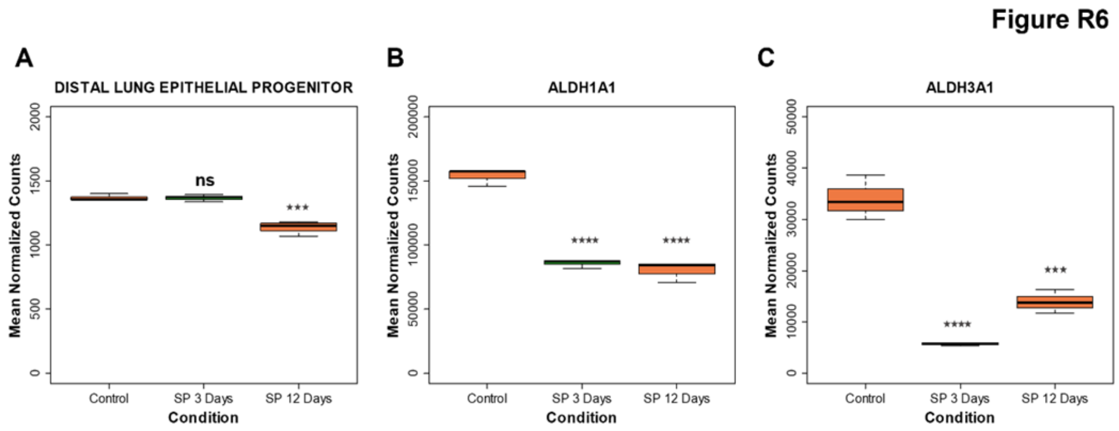

Figure R6. Propionate's effect on lung epithelial progenitor and cancer stemness gene expression. A-C. Box plot visualization of the distal lung epithelial progenitor genes (A), ALDH1A1 (B) and ALDH3A1 (C) in RNA-seq profile of SP treated A549 cell line for 3 days and 12 days. Significance was calculated using un-paired t-test between the SP treated cells and the control. ns – non-significant, *** - p<0.001, **** - p<0.0001.

5. Mouse models support the authors finding but do not demonstrate any EMT involvement. The expression of EMT features in the tumors before and after propionate treatment should be checked by classic immunolocalization (or any other approach) to

estimate the level of cell depolarization, dissociation and expression of EMT functional markers.

Response R2.5 As suggested by the Reviewer, we analyzed by IHC the expression pattern of epithelial cell adhesion molecule (EPCAM) protein in the KPL mouse lung cancer tissues treated with SP compared to controls. An increase in the expression of EPCAM in SP treated mouse samples with a trend for significance (p=0.08) was found (**Fig R7A**). To further analyze the samples in a more genome-wide manner, we performed RNA-seq analysis on the same lung tumor tissues. As expected, we found top significantly enriched molecular processes to be cell adhesion in SP administered KPL mouse tumor samples compared to the control (**Figs R7B-D**). Further, we also observed significantly down-regulated mesenchymal genes such as CLDN2 (Wei *et al*, 2021), GPX2 (Peng *et al*, 2023) and PCLAF (Wang *et al*, 2016). All these validate the SP mode of enhanced epithelial features in inhibiting EMT. The results are included in the revised manuscript (Page: 10; Line: 244-249; **Appendix Figs S3D-F**).

Figure R7

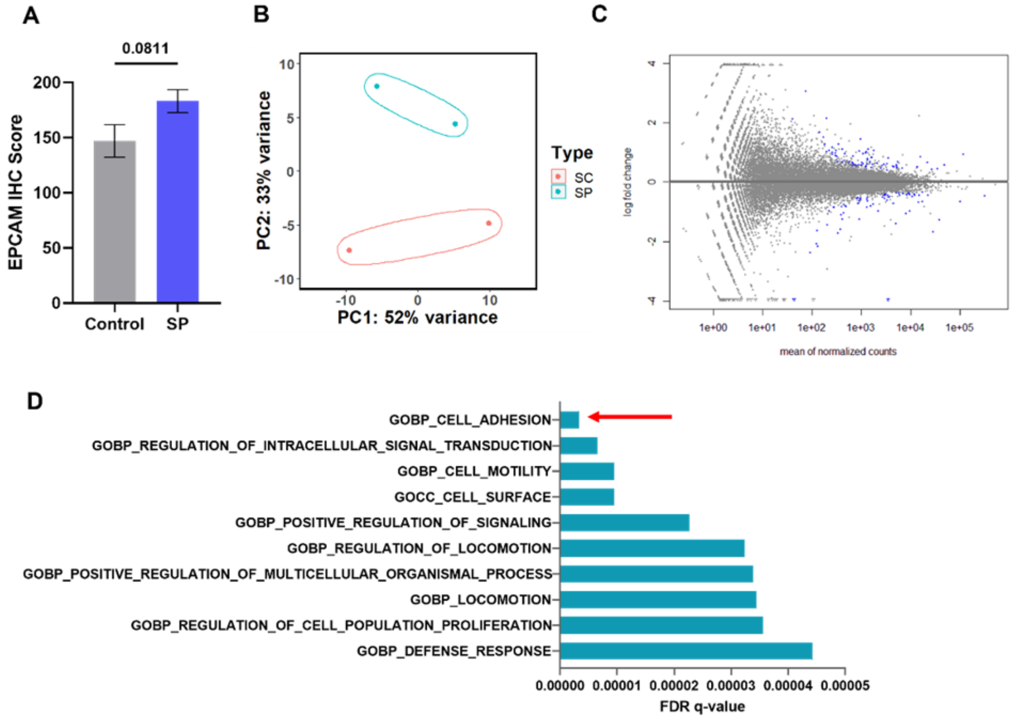

Figure R7. A. Immunohistochemistry staining of EPCAM in lung tissue of KPL-mouse administered with SP. Significance was calculated using un-paired t-test between the SP treated cells and the control. B. Principal component analysis of RNA-seq expression profile of lung tissue samples from Cas9-C57BL/6 mice with lung tumorigenesis intubated with AAV-KPL virus and administered with sodium propionate (SP) in drinking water. C. M-A plot of differential gene expression between sodium chloride and sodium propionate administered Cas9-C57BL/6 mice with lung tumorigenesis intubated with AAV-KPL virus. D. Gene-set ontology of differentially expressed genes from RNA-seq expression profile of lung tissue samples from Cas9-C57BL/6 mice with lung tumorigenesis intubated with AAV-KPL virus and administered with sodium propionate (SP) in drinking water.

6. In Fig 2. wound healing experiments are conclusive in showing a delay in cell sheet spreading after SP treatment, but they do not indicate a migration halt. It would be

important to look at higher magnification to check if the cells are individualized or migrate as an epithelial sheet. Also, the number of repeat experiments should be mentioned.

Response R2.6 It is true that SP treatment delays but do not halt the migration. We have now provided magnified images of the cells at the early stages of migration with SP treatment to better visualize the migration front, as requested (**Fig R8A**). For space limitations, these magnified pictures have not been added in the manuscript. Migration experiment was performed at least 3 independent times observing significant inhibition in migratory ability of A549 cell line with SP treatment (see 3 replicates in **Figs R8B-D**), and this has now been included in the revised manuscript of figure legend **Fig 2** (Page: 57; Line: 1458-1460). In addition, we also performed an additional migration experiment suggested by Reviewer 3 (Please see Response R3.3). TGFβ1-induced EMT cells were found inhibited during migration by SP or SB when treated in combination or pre-treatment scenarios (**Fig R18**).

Figure R8

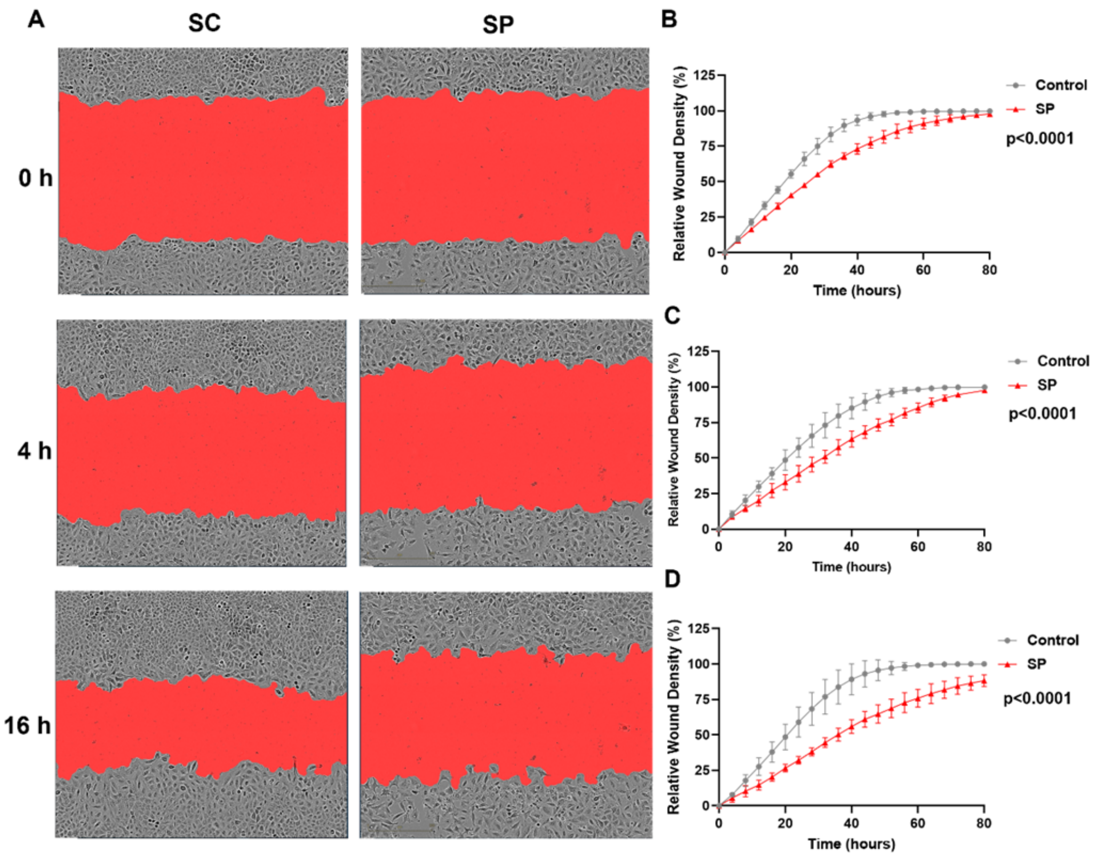

Figure R8. Cell migration analysis in A549 cell line treated with sodium propionate for 3 days. A. Magnified images represent the wound width (highlighted by red region) at the indicated time points in A549 cells treated with SP for 3 days for migration assay. B-D. Line plots depict the rate of wound healing, expressed as relative wound density in A549 cells treated with SP over the course of 72 hours from making the wound for migration assay.

7. To study cell plasticity, an essential feature of EMT, it would be important to insert

A549 spheroids into 3D collagen gels to monitor and quantify migration and invasive pattern, with or without treatment.

Response R2.7 Thanks for this relevant suggestion. As requested by the Reviewer, we tried to study the cell plasticity of A549 spheroids embedded in the 3D collagen gels with the treatment of SP (at 5 mM). Briefly, A549 spheroids were seeded on a coverslip coated with collagen type I and on the top, a drop of collagen was added forming a 3D milieu. However, we did not find any effect of SP on the migration (**Fig R9A**). This could be due to weak diffusion of propionate into the 3D collagen scaffolds due to the charge of the polymers. Therefore, we tried few other alternative approaches using 2D culturing of spheroids with collagen wherein the spheroids were anchored to a flat surface by coating the coverslip with collagen type I. Here, we observed a significant reduction in the invasive ability of the cells treated with SP (**Fig R9B-C**). In another approach, we seeded 2500 cells/well in a 96-well ultra-low adherent plate and allowed cells to form spheroids for 3 days. After 3 days, we treated the spheroids with SP and/or TGF-β1 and layered them with Matrigel. The spheroids were then examined by live-cell imaging. The results showed that TGF-β1-induced invasive ability was strongly inhibited by co-treatment with SP (**Fig R10**), as also observable on the microscopic pictures. We believe these data are sufficient to indicate a proof-of-concept role of SP on modulating cell plasticity in spheroids settings, while more conditions and spheroid assays could be required in future to fully elucidate the role of SP in inhibiting the cell plasticity. The results of **Fig R10** have been included in the revised manuscript (Page: 9; Line: 205; **Fig EV1M**).

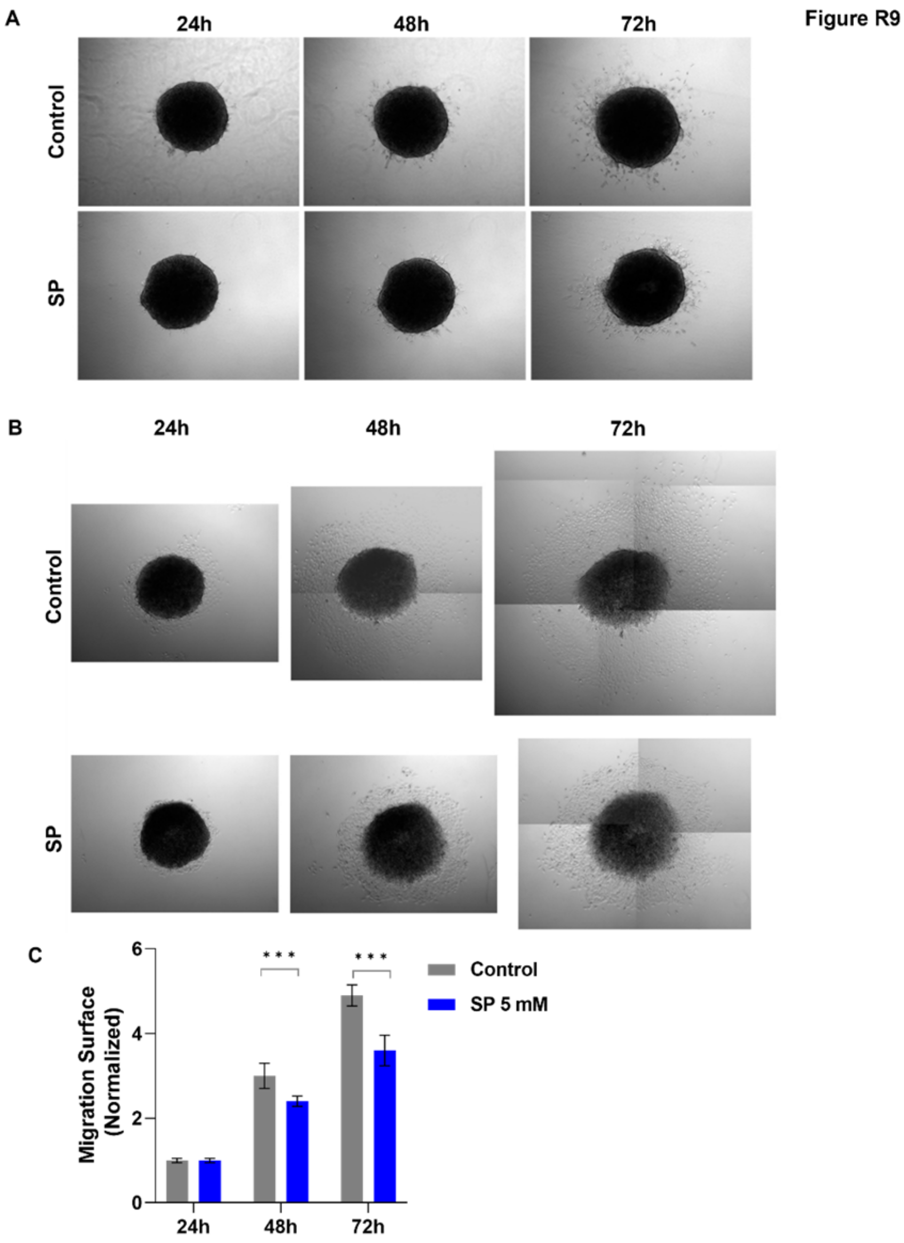

Figure R9. Spheroid assay in A549 cells treated with sodium propionate for 3 days. A. Images representing the A549 spheroids inserted into 3D collagen gels treated with sodium propionate (5 mM) for 3 days (n=5). B. Images representing the A549 spheroids anchored to the collagen type I (n=5) treated with SP (5 mM). C. Quantification of migratory pattern of A549 spheroids by anchoring to the collagen type I treated with SP (5 mM). *** p-value < 0.001.

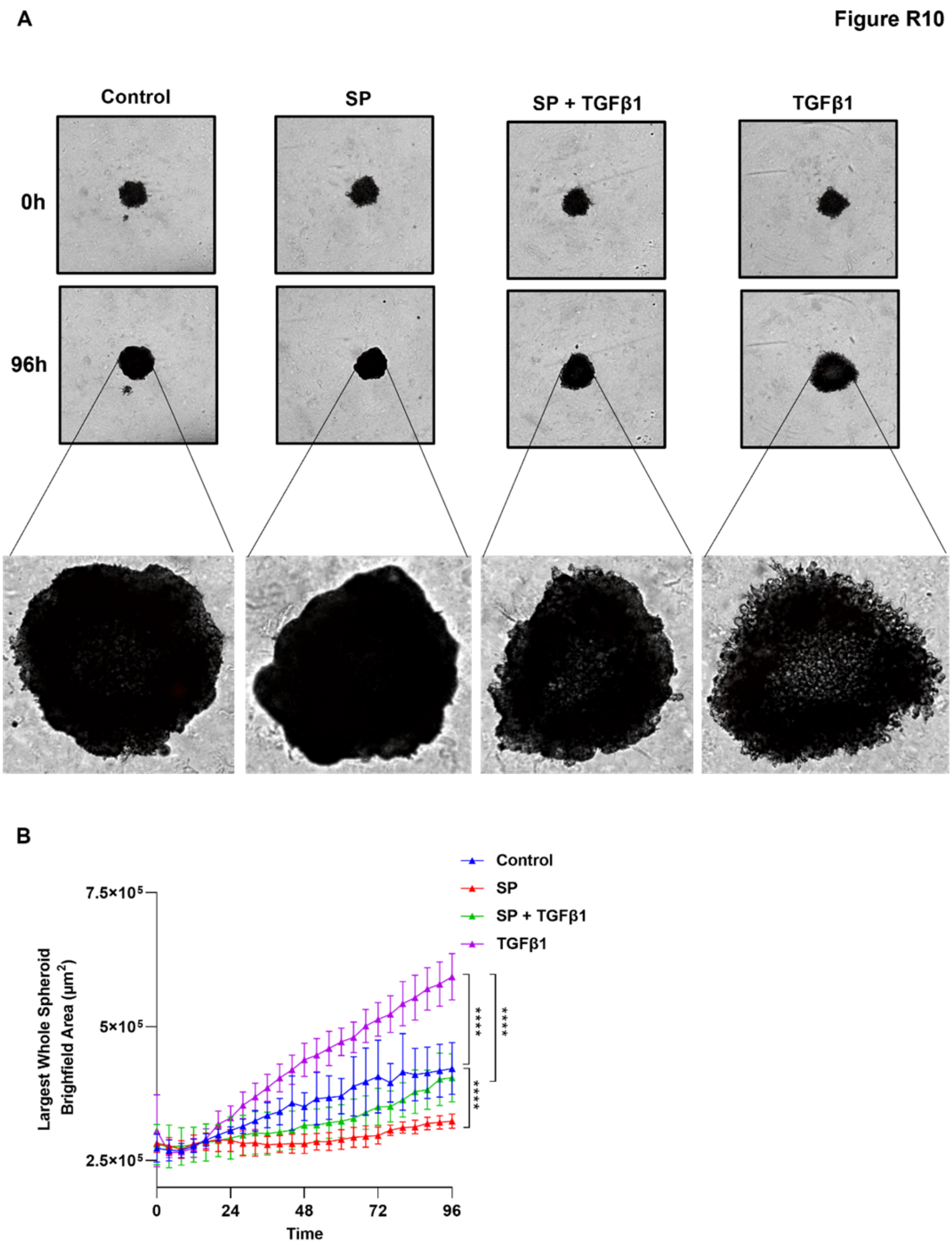

Figure R10. Spheroid assay in A549 cells treated with sodium propionate (SP) and/or TGF-β1. A. Images representing the A549 spheroids (n=4) treated with SP (5 mM) and/or TGF-β1 (2 ng/ml) for 4 days. Inserts show the magnified spheroids with invasion in TGF-β1 treated wells and inhibition by SP when co-treated SP with TGF-β1. B. Quantification of spheroid brightfield area for A549 spheroids treated with SP and/or TGF-β1. **** p-value < 0.0001.

Minor points

1. In Fig 1F, "Propanoate" is used for propionate found everywhere else in the manuscript. One word should be consistently used.

Response R2.8 We have now changed the word "Propanoate" in **Fig 1F**, and thereafter, we consistently used the word "Propionate" in the manuscript text except in the **Figs 1A-C** as the signature is named in the KEGG repository as "Propanoate".

2. It is not clear what should be seen in Fig 3i. A stronger histological staining and additional higher magnification pictures would be helpful.

Response R2.9 In the current revised manuscript **Fig 3I**, we have now used red arrows to point out the metastatic cells in the lymph nodes of control mice (LN-Control) as can be observed in **Fig R11**.

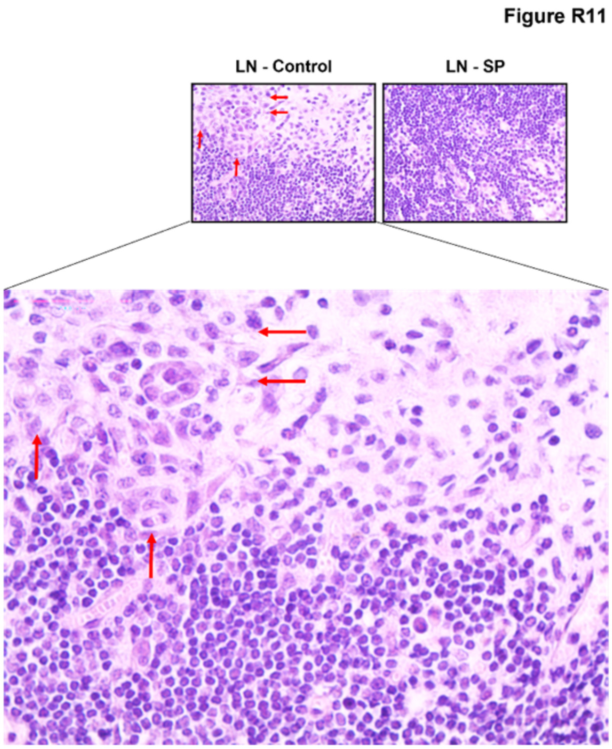

Figure R11. Representative H&E images of lung cancer metastasis to axial lymph nodes in KPL mice of control (LN-Control) condition while no metastasis in the sodium propionate (LN-SP) condition. Scale bar represents 200 μm. Red arrows point to the metastatic cells in the lymph nodes.

3. Fig3H. It is not clear how many mice were used for this survival study.

Response R2.10 We thank the reviewer for pointing out this. In **Fig 3H**, we have used n=7 mice per group. This is now appended to the revised manuscript in **Fig 3H**, and also in the corresponding legend of the revised manuscript text (Page: 58; Line: 1493-1495).

4. Fig 5E higher magnification pictures would be useful to assert ZO-1 and Epcam localization and organization pattern.

Response R2.11 We have now provided higher magnification pictures of ZO-1 and EPCAM staining to visualize the localization and organization pattern of the epithelial markers. We detect an increase in the expression of ZO-1 and EPCAM in the membrane regions with SP treatment, indicating the functional level process of epithelial feature establishment with SP (**Fig R12**). We have also provided the magnified images in the revised manuscript (**Fig EV2K).**

Figure R12

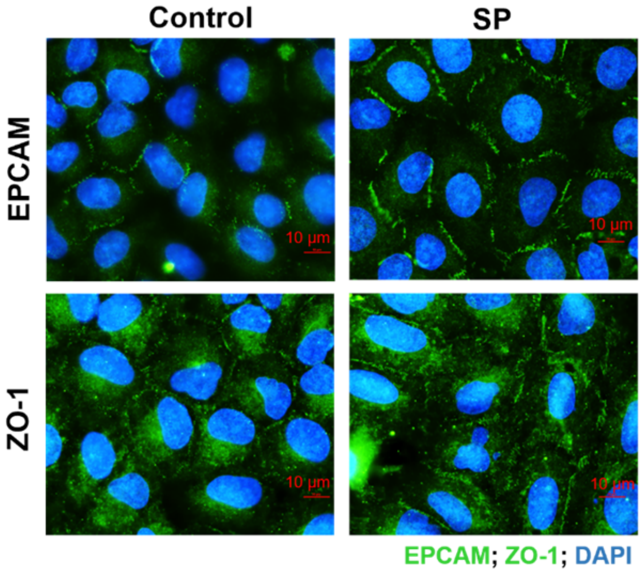

Figure R12. Magnified images of immunofluorescence staining of EPCAM and ZO-1 in A549 cell line treated with sodium propionate (SP) at 5 mM for 3 days. DAPI was used as a nuclear stain. Scale bar represents 10 µm.

5. Discussion is overall too long and redundant. It should be more focused on this manuscript findings. For example, the section l409-l426 about SP and cisplatin resistance is quite interesting, but does not imply EMT pathways. Similarly, the section about SCFA availability (l428-l445) is rather unclear.

Response R2.12 As suggested, we have now improved the discussion section by highlighting the findings in the study with more focused discussion. We have also rephrased the section of SCFA availability (Page: 22; Line: 540-545), and SP's involvement in cisplatin sensitization (Page: 19; Line: 466-470). However, with the additional revision experiments and additional citations requested the discussion is still long.

6. In Fig S1, Zeb2 and Axl are found to be downregulated by TGFb treatment. This is not the consensus response described in other reports studying EMT pathways. Any comments?

Response R2.13 Good observation. Indeed, it is well known that ZEB2 and AXL are targets of TGF-β1. The mRNA expression profile of NCI-H358, HCC827 and A549 treated with TGF-β1

used in **Appendix Figs S1A-C** of the study were obtained from the publicly available database Gene Expression Omnibus (GEO-GSE49644). We utilized this profile to examine the overall changes in the EMT gene signature obtained from (Mak *et al*, 2016) as a validation. But we are unsure why AXL and ZEB2 are found down-regulated. However, in our own hands we also found AXL and ZEB2 were not increased with TGF-β1 treatment in A549 cell line (**Fig R13**). In the previous literature, we also found a similar case wherein A549 treated with TGF-β1 did not induce AXL by immunoblotting (Choi *et al*, 2021). We believe this requires further experiments to justify these EMT markers regulations in A549 cells with the treatment of TGF-β1, although this has no impact on our manuscript. We did not modify the manuscript.

Figure R13

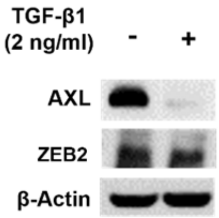

Figure R13. Western blot analysis of AXL and ZEB2 in A549 cells treated with TGF-β1 (2 ng/ml) for 72 hours. β-Actin was used as an internal control.

7. It should be mentioned that most cancer data sets used in this study involve a mix of stroma and tumor cells, an element to consider in the discussion.

Response R2.14 As suggested by the Reviewer, we have included a statement in the discussion section (Page: 17; Line: 404-409) that *"tumor gene expression profiles used in the study from the publicly available database could involve a mix of stroma and tumor cells, and the potential contribution of the tumor microenvironment remains to be investigated".*

8. The statement l409." Our lung experimental metastasis model showed that SP can inhibit metastasis." is too strong. SP treatment has an impact, but does not appear to inhibit metastasis overall.

Response R2.15 As correctly noted by the Reviewer, we have toned down the phrase in line l409: "**Our lung experimental metastasis model showed that SP can inhibit metastasis**" to "***our lung experimental metastasis model showed that SP could reduce the metastasis***" (Page: 19; Line: 460-462).

Referee #3 (Remarks for Author):

The epithelial-to-mesenchymal transition (EMT) is a cellular program that cancer cells can utilize to gain metastatic ability. However, targeting the EMT can be tricky since its regulation is primarily mediated by transcription factors, which are challenging to inhibit. In contrast, metabolic enzymes may represent promising druggable targets for inhibiting EMT. Therefore, investigating the role of metabolic processes in the EMT program could lead to identifying new drug targets. In their study, Ramesh et al. aimed to uncover unknown metabolic processes involved in the EMT program through transcriptome analysis. Their findings revealed that the propionate and butanoate metabolism gene set negatively correlated with the EMT gene set. Further experiments demonstrated that treatment with propionate and butanoate induced the cells to acquire more epithelial characteristics. Additionally, propionate inhibited metastasis and sensitized the cells to cisplatin. Finally, they show that propionate reduces lung cancer EMT by

broad chromatin remodeling. However, all of the experiments were conducted by treating epithelial cells with fatty acids, and the role of the endogenous propionate and butanoate in the EMT program is unclear. Additionally, the effect of these fatty acids on mesenchymal-like lung cancer cells needs to be investigated. Thus, the role of endogenous propionate and butanoate in EMT program regulation remains unclear and deserves investigation.

Major comments

1. The authors suggest that the short-chain fatty acids induce the cells to become more epithelial. For example, in Figure 2, they demonstrate that treatment of A549, SK-MES-1, and NCI-H23 cells with propionate and butanoate induces the cells to gain more epithelial characteristics. However, the biological meaning of this finding is unclear as these cells are already in the epithelial state. Therefore, these experiments should also be conducted in lung cancer cell lines in a partially mesenchymal state. Specifically, these experiments will determine whether these short-chain fatty acids can induce the MET program.

Response R3.1 As rightly pointed out, it is true that investigating the propionate's and butanoate's ability to increase the epithelial features should be done in a mesenchymal or in a partial-EMT cell (highly-aggressive and highly-metastatic state (Pastushenko *et al*, 2018)). However, previous study on scoring EMT in both clinical samples and in cancer cell line encyclopedia (CCLE) across several cancer types, showed that NSCLC is situated in between epithelial and mesenchymal state implying a partial EMT mode (Tan *et al*, 2014). Similarly, another study predicted and validated the partial EMT state of A549 cell line where it was observed to co-express both epithelial and mesenchymal markers (George *et al*, 2017), data also confirmed by our own single cell RNA-seq (Page: 13; Line: 301-308).

Anyway, based on this relevant comment, in the current revised manuscript we have now included treatment with SCFAs (SP and SB) of additional mesenchymal-like NSCLC cells CALU-1 (Thomson *et al*, 2008) and H1299 (Tan *et al*, 2018). As a result, although we observed an increase in the epithelial markers expression with the SP treatment (E-cadherin, GRHL1 and EPCAM), less to no difference in the mesenchymal markers (ZEB1, BCAT1 and NNMT) was found in CALU-1. The epithelial reinforcement effect was not observed with SB except for E-cadherin (**Fig R14A-B**). In case of H1299, we did not observe any increase of E-cadherin with SP/SB, but only a moderate EPCAM up regulation. Similarly, we did not observe any changes in the mesenchymal markers (ZEB1 and BCAT1) in this cell line (**Fig R14C-D**). Overall, there was a no or lower effect on mesenchymal-like NSCLC cell lines compared to partial EMT cell line A549. The results have been now appended in the current revised manuscript (Page: 7; Line: 164-173; **Figs EV1D** and **EV1E**).

It also needs to be considered that in the previous version of the manuscript we also included an experiment wherein we induced A549 cells to become fully mesenchymal by treating with TGF-β1, and then treated the cells with SP or SB (**Figs EV1I** and **EV1J** in the revised manuscript). Those experiments also revealed that SP and SB could not revert the EMT program when the same A549 cells were already induced in the mesenchymal state. Altogether, our data seem to indicate that SCFA SP and SB could better inhibit the partial-EMT state while have no or less effect when the cells are transformed to full mesenchymal state. This could be in the future better mechanistically elucidated, and this observation could be important in the selection of the patients to be enrolled in clinical trials testing SCFA and has been now added in the discussion (Page: 18; Line: 429-434). Thanks for the important input.

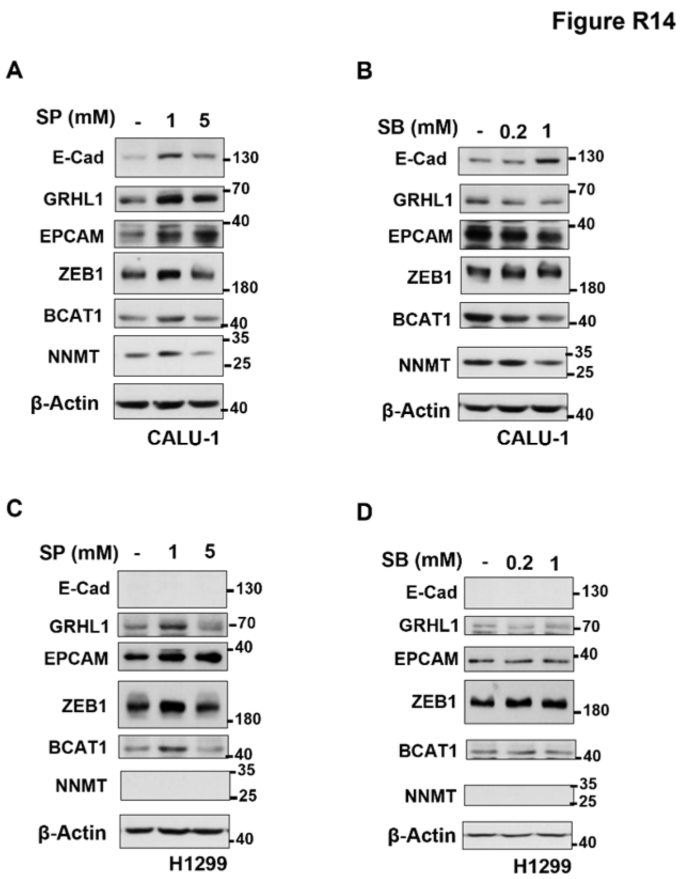

Figure R14. A-D. Western blot analysis of epithelial (E-Cadherin, GRHL1 and EPCAM) and mesenchymal (ZEB1, BCAT1 and NNMT) markers in CALU-1 (A and B) and in H1299 (C and D) cell lines treated with sodium propionate (SP) or sodium butanoate (SB) in the indicated dose-dependent manner for 48 hours. β-Actin was used as an internal control.

2. The authors analyzed gene expression databases to identify the metabolic processes. They assumed that short-chain fatty acids are regulated at the transcription level. However, they did not validate or elaborate on this analysis. For example, which enzymes in the short-chain fatty acid biosynthesis pathway are downregulated during the EMT program or in epithelial vs. mesenchymal cells? What will be the outcome of their ectopic expression in epithelial and mesenchymal cells? What will be the outcome of manipulating the enzymes synthesizing these fatty acids? Will the cells induce the EMT program?

Response R3.2 Very good point. We previously carried out knock down of metabolic enzymes involved in main (PCCB, MCEE and MUT) and shunt (HIBCH and ALDH6A1) propionate metabolism and did not observe any change in E-cadherin/ ZEB1 in cells treated with SP (**Appendix Figs S6B** and **S6C**). Interestingly, it has recently been shown that deregulation in the propionate metabolism can contribute to an anaplerotic reaction in the accumulation of methylmalonic acid (MMA) in breast and lung cancer leading to EMT and metastasis (Gomes *et al*, 2022). In apparent contrast to this, we observe that treatment with propionate results in the epigenetic involvement in inducing epithelial identity implying a dual and opposing effect on EMT. This has been detailed in the discussion section of the manuscript (Page: 19; Line: 448-455).

Following this comment, we now tested if the treatment of propionate has any increase in the metabolic pathway enzymes. We treated A549 cell line with SP and found a relative increase in the PCCA and PCCB although only at later time points (**Fig R15**), implicating that probably epigenetic involvement overrides the metabolic counterpart and reinforces the epithelial identify of the cells. However, these may be crucial aspects in the cellular EMT control in lung cancer and beyond, which would require a dedicated effort in the future, including, for instance, the genetic and pharmacological manipulation of these enzymes in vitro and in vivo, as mentioned by the Reviewer. Our lab is working on it, for instance with the employment of custom lung tumor-specific knockout models via CRISPR/Cas9. We have highlighted about this in the discussion (Page: 14; Line: 339-340 and **Appendix Fig S6D**).

Figure R15

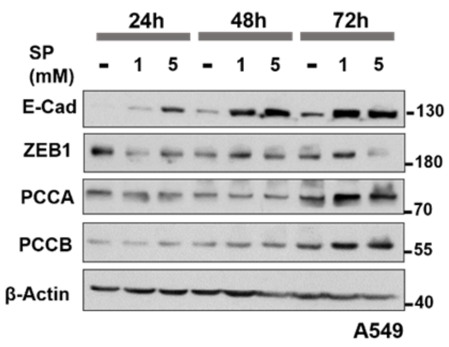

Figure R15. Western blot analysis of epithelial (E-cadherin), mesenchymal (ZEB1), and propionate metabolic genes (PCCA and PCCB) in A549 cell line treated with sodium propionate (SP) in the indicated dose- and time-dependent manner. β-Actin was used as an internal control.

3. One of the main themes in this manuscript is that the short-chain fatty acids function as inhibitors of the EMT. However, the physiological relevance of these findings is still missing as they did not validate any changes in the fatty acid intracellular levels. For example, does the intracellular level of propionate and butanoate reduce during the EMT?

Response R3.3 Very important point. As suggested by the Reviewer, we have now performed an experiment to quantify intracellular propionate during EMT. We induced EMT by treating a large amount of A549 cells (around 7.5 million cells per replicate) with TGF-β1 for 4 days (**Fig R16A**), and performed mass spectrometry analysis to quantify the intracellular propionate at different time points (48h, 72h and 96h) using 13C labelled standards (**Figs R16B-C**). Since the basal level of propionate in the cells was below or around the blank estimate, we were technically unable to quantify intracellular amount of propionate (**Fig R16D**). However, we tested if the treatment of A549 cell line with sodium propionate could increase the intracellular propionate. As expected, a very significant increase (Folds=~27; p=0.0006) was detected (**Fig R16E**), implying that the quantification method is working and that the physiological origin of propionate may not be intracellular.

We believe that propionate in NSCLC might be contributed by the tumor microenvironment, and lung resident microbiota are a possible suspect due to the fact that bacteria are the natural source of propionate in the body (in the gut, for instance). [...]

[...] We have added the quantification of the levels of propionate upon treatment to the revised manuscript (Page: 8; Line: 188-191; **Fig EV1H**) because we believe it nicely confirms the high cellular uptake for potential treatment purpose (Page: 22; Line: 543-551). We did not address butanoate as the main focus of the manuscript was on propionate. Thanks for the input.

Figure R16

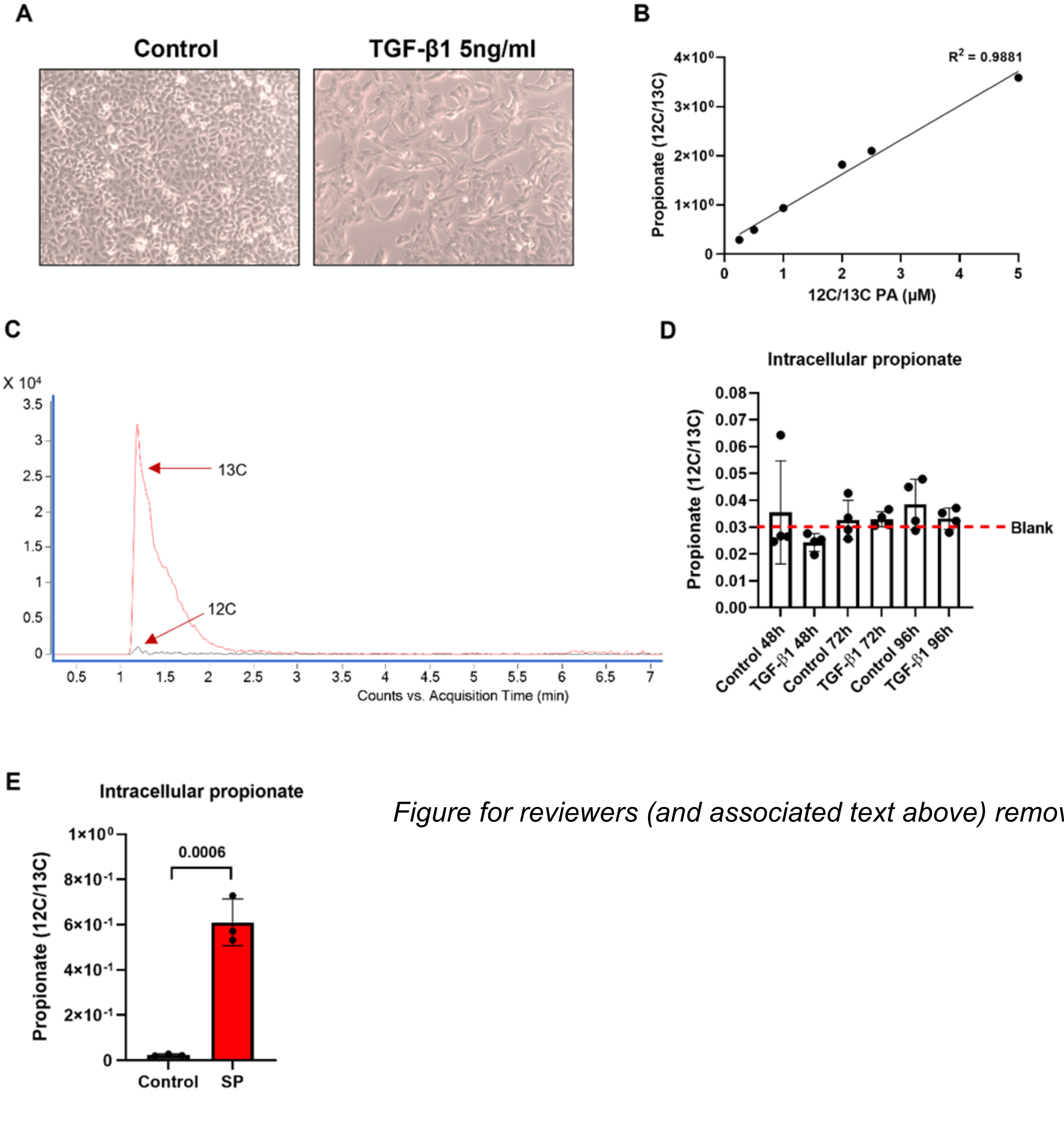

Figure for reviewers (and associated text above) removed

Figure R16. A. Images represent the A549 cell line treated with TGF-β1 (5 ng/ml) for 96 hours. B. Linear regression plot of the calibration curve of propionate (12C/13C) peak area. 13C propionate was used as an internal standard. C. Chromatogram plot of the propionate detection of 12C and 13C in A549 cell line sample. 13C propionate was used as an internal standard. Red arrows indicate the 12C and 13C in the chromatogram. D. Detection of intracellular levels of propionate in A549 cell line treated with TGF-β1 (5 ng/ml) at the indicated time points. 13C propionate was used as an internal standard. E. Detection of intracellular levels of propionate in A549 cell line treated with SP (5 mM) for 3 days. 13C propionate was used as an internal standard. F. *Figure for reviewers removed*.

4. The role of these short-chain fatty acids as regular of the EMT program is needed to be elaborated. Indeed, Figures 2H-I show that SP and SB inhibit EMT markers expression upon TGFß treatment. However, this was demonstrated only on two markers which is insufficient to conclude. Additionally, showing changes in the marker's expression is not enough to conclude any effect on the EMT program, as the effect on the cell's migratory capabilities is needed to be examined.

Response R3.4 As suggested by the Reviewer, we have included additional EMT-specific marker NNMT (Shaul *et al*, 2014; Yang *et al*, 2022) to demonstrate SP's or SB's action on EMT inhibition upon treating the A549 cell line both by pre-treatment of cells with SCFA followed by TGF-β1 treatment (**Fig R17A**) or by co-treatment of cells with SCFA and TGF-β1 (**Fig R17B**). In both cases, we observed NNMT to be down-regulated by SCFA's action.

Figure R17

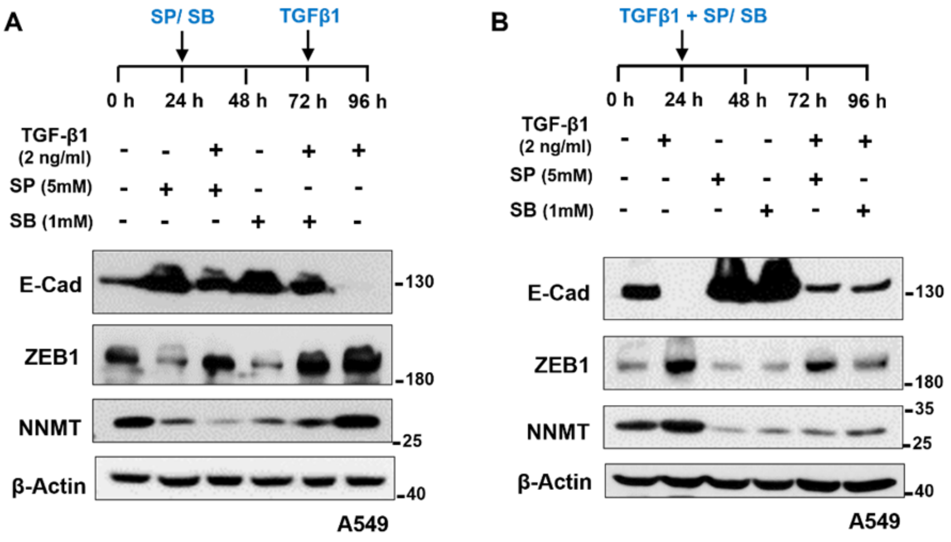

Figure R17. A. Western blot analysis of E-cadherin, ZEB1 and NNMT protein levels in A549 cells pre-treated with sodium propionate (SP, 5 mM) or sodium butanoate (SB, 1 mM) for 48 hours followed by TGF-β1 (2 ng/ml) for 24 hours. β-Actin was used as an internal control. B. Western blot analysis of E-cadherin, ZEB1 and NNMT protein levels in A549 cells co-treated with sodium propionate (SP, 5 mM) or sodium butanoate (SB, 1 mM) in combination with TGF-β1 (2 ng/ml) for 72 hours. β-Actin was used as an internal control.

We also agree that markers alone are anyway not sufficient and that functional assays like in vitro migration are more indicative of EMT. We have also now performed cell migration analysis with A549 pre-treated or co-treated with SP and TGF-β1, as suggested. As expected, we found a significant reduction in the migratory ability of the cells between SP, and SP with TGF-β1 (**Fig R18A** and **R18B**). A similar level of inhibitory effect of SB on TGF-β1 induced migration was also observed (**Fig R18C**). We have included the result of **Fig R18B** in the revised manuscript (Page: 9; Line: 203-204; **Fig 2K**). Thanks for the suggestion.

Figure R18

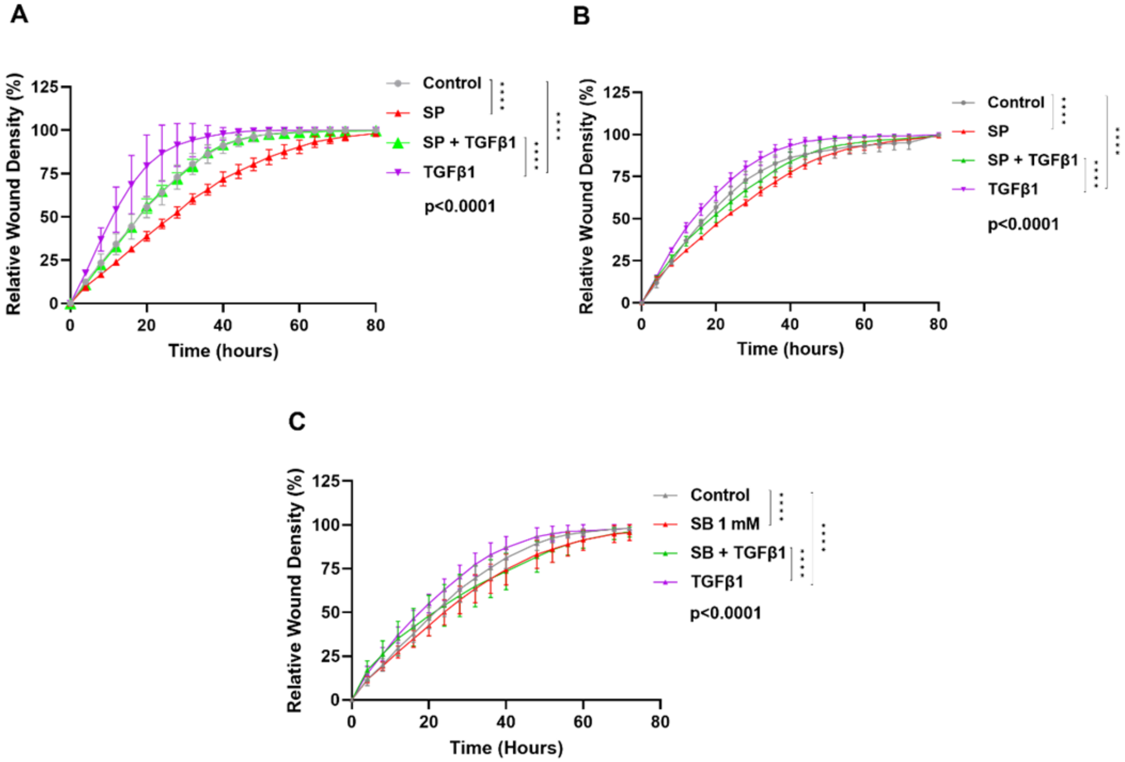

Figure R18. A-C. Line plot depicts the rate of wound healing, expressed as relative wound density from making the wound for migration assay in pre-treated A549 cells with SP for 48 hours followed by TGFβ1 for 24 hours (A), in co-treated SP and TGFβ1 A549 cells (B), and in co-treated SB and TGFβ1 A549 cells (C) over the course of 72 hours. Significance was calculated using Two-way ANOVA followed by inter-group comparison using Tukey's multiple comparison test. **** p-value < 0.0001.

Minor comments

1. The HR (hazard ratio) value is absent from all of the Kaplan-Meier plots that pertain to survival rates.

Response R3.5 We thank the reviewer for the suggestion, we have provided the hazard ratios for all the survival figures (**Fig 1F**, **Fig 3H**, and **Figs EV3H** and **EV3I**) along with the corresponding confidence intervals in the revised manuscript.

2. In Figures 2B and 2C, why do E-cadherin levels change over time in samples without the short-fatty acids treatment? Additionally, the ZEB1 blot is problematic. In Figure 2C

treatment, its expression levels don't change, and in Figure 2B, there is inconsistency with SP doses. Additional mesenchymal markers are needed to clarify this point.

Response R3.6 A similar concern was raised by the Reviewer 2 (please see more detailed response R2.2), and the increase in E-cadherin over the time is due to the culture confluence as the cells were harvested from low confluence to high confluence over the course of treatment for 72h. In case of ZEB1, which is normally a hard protein to detect, this was found down-regulated at 72h with SCFA (SP and SB) treatment in all 3 independent repeats (**Fig R2**). The down-regulation of ZEB1 in the SP treated cells is also consistent with longer treatment from 3 days to 12 days in all 3 independent repeats (**Fig R3**). We have included the quantification in the **Fig R2 and Fig R3** for E-cadherin and ZEB1.

In addition, as suggested by the Reviewer, we have also shown that SP inhibits EMT by few additional EMT-specific markers such TWIST1 and NNMT wherein these markers were down-regulated with the SP treatment (**Fig R19**). The result is included in the revised manuscript (Page: 8; Line: 186-188; **Appendix Fig S2E**).

Figure R19

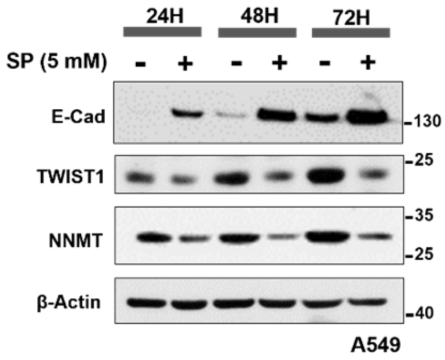

Figure R19. Western blot analysis of E-cadherin, TWIST1 and NNMT protein levels in A549 cells treated with sodium propionate (SP, 5 mM) in the indicated time-dependent manner. B-Actin was used as an internal control.

Other changes:

This resubmission work has required the involvement of new external and internal collaborators for the mass-spec quantification of propionate (3 authors from the University of Southern Denmark), the spheroid assays (2 authors from the University of Heidelberg) and for the immunohistochemistry on mouse samples (2 authors from the University of Turin), which have been added to the list of co-authors and their specific contribution has been mentioned in the dedicated section.

Rebuttal References:

Botticelli A, Vernocchi P, Marini F, Quagliariello A, Cerbelli B, Reddel S, Del Chierico F, Di Pietro F, Giusti R, Tomassini A *et al* (2020) Gut metabolomics profiling of non-small cell lung cancer (NSCLC) patients under immunotherapy treatment. *Journal of translational medicine* 18: 49

Chen L, Zhou X, Wang Y, Wang D, Ke Y, Zeng X (2021) Propionate and Butyrate Produced by Gut Microbiota after Probiotic Supplementation Attenuate Lung Metastasis of Melanoma Cells in Mice. *Molecular nutrition & food research* 65: e2100096

Choi JY, Lee H, Kwon EJ, Kong HJ, Kwon OS, Cha HJ (2021) TGFβ promotes YAP-dependent AXL induction in mesenchymal-type lung cancer cells. *Molecular oncology* 15: 679-696

Fan F, Yin R, Wang L, Zhao S, Lv D, Yang K, Geng S, Yang N, Zhang X, Wang H (2021) ALDH3A1 driving tumor metastasis is mediated by p53/BAG1 in lung adenocarcinoma. *Journal of Cancer* 12: 4780-4790

George JT, Jolly MK, Xu S, Somarelli JA, Levine H (2017) Survival Outcomes in Cancer Patients Predicted by a Partial EMT Gene Expression Scoring Metric. *Cancer research* 77: 6415-6428

Gomes AP, Ilter D, Low V, Drapela S, Schild T, Mullarky E, Han J, Elia I, Broekaert D, Rosenzweig A *et al* (2022) Altered propionate metabolism contributes to tumour progression and aggressiveness. *Nature metabolism* 4: 435-443

Høgh RI, Møller SH, Jepsen SD, Mellergaard M, Lund A, Pejtersen M, Fitzner E, Andresen L, Skov S (2020) Metabolism of short-chain fatty acid propionate induces surface expression of NKG2D ligands on cancer cells. *FASEB journal : official publication of the Federation of American Societies for Experimental Biology* 34: 15531-15546

Lyu J, Pirooznia M, Li Y, Xiong J (2022) The short-chain fatty acid acetate modulates epithelial-to-mesenchymal transition. *Molecular biology of the cell* 33

Mak MP, Tong P, Diao L, Cardnell RJ, Gibbons DL, William WN, Skoulidis F, Parra ER, Rodriguez-Canales J, Wistuba, II *et al* (2016) A Patient-Derived, Pan-Cancer EMT Signature Identifies Global Molecular Alterations and Immune Target Enrichment Following Epithelial-to-Mesenchymal Transition. *Clinical cancer research : an official journal of the American Association for Cancer Research* 22: 609-620

Mirzaei R, Afaghi A, Babakhani S, Sohrabi MR, Hosseini-Fard SR, Babolhavaeji K, Khani Ali Akbari S, Yousefimashouf R, Karampoor S (2021) Role of microbiota-derived short-chain fatty acids in cancer development and prevention. *Biomedicine & pharmacotherapy = Biomedecine & pharmacotherapie* 139: 111619

Nath A, Li I, Roberts LR, Chan C (2015) Elevated free fatty acid uptake via CD36 promotes epithelial-mesenchymal transition in hepatocellular carcinoma. *Scientific reports* 5: 14752

Nomura M, Nagatomo R, Doi K, Shimizu J, Baba K, Saito T, Matsumoto S, Inoue K, Muto M (2020) Association of Short-Chain Fatty Acids in the Gut Microbiome With Clinical Response to Treatment With Nivolumab or Pembrolizumab in Patients With Solid Cancer Tumors. *JAMA network open* 3: e202895

Pastushenko I, Brisebarre A, Sifrim A, Fioramonti M, Revenco T, Boumahdi S, Van Keymeulen A, Brown D, Moers V, Lemaire S *et al* (2018) Identification of the tumour transition states occurring during EMT. *Nature* 556: 463-468

Peng F, Xu Q, Jing X, Chi X, Zhang Z, Meng X, Liu X, Yan J, Liu X, Shao S (2023) GPX2 promotes EMT and metastasis in non-small cell lung cancer by activating PI3K/AKT/mTOR/Snail signaling axis. *FASEB bioAdvances* 5: 233-250

Ramesh V, Brabletz T, Ceppi P (2020) Targeting EMT in Cancer with Repurposed Metabolic Inhibitors. *Trends in cancer* 6: 942-950

Rawlins EL (2008) Lung epithelial progenitor cells: lessons from development. *Proceedings of the American Thoracic Society* 5: 675-681

Shaul YD, Freinkman E, Comb WC, Cantor JR, Tam WL, Thiru P, Kim D, Kanarek N, Pacold ME, Chen WW *et al* (2014) Dihydropyrimidine accumulation is required for the epithelial-mesenchymal transition. *Cell* 158: 1094-1109

Song M, Chan AT (2019) Environmental Factors, Gut Microbiota, and Colorectal Cancer Prevention. *Clinical gastroenterology and hepatology : the official clinical practice journal of the American Gastroenterological Association* 17: 275-289

Tan TZ, Miow QH, Miki Y, Noda T, Mori S, Huang RY, Thiery JP (2014) Epithelial-mesenchymal transition spectrum quantification and its efficacy in deciphering survival and drug responses of cancer patients. *EMBO molecular medicine* 6: 1279-1293

Tan X, Banerjee P, Liu X, Yu J, Gibbons DL, Wu P, Scott KL, Diao L, Zheng X, Wang J *et al* (2018) The epithelial-to-mesenchymal transition activator ZEB1 initiates a prometastatic competing endogenous RNA network. *The Journal of clinical investigation* 128: 1267-1282

Thomson S, Petti F, Sujka-Kwok I, Epstein D, Haley JD (2008) Kinase switching in mesenchymal-like non-small cell lung cancer lines contributes to EGFR inhibitor resistance through pathway redundancy. *Clinical & experimental metastasis* 25: 843-854

Trompette A, Pernot J, Perdijk O, Alqahtani RAA, Domingo JS, Camacho-Muñoz D, Wong NC, Kendall AC, Wiederkehr A, Nicod LP *et al* (2022) Gut-derived short-chain fatty acids modulate skin barrier integrity by promoting keratinocyte metabolism and differentiation. *Mucosal immunology* 15: 908-926

Wang X, Jung YS, Jun S, Lee S, Wang W, Schneider A, Sun Oh Y, Lin SH, Park BJ, Chen J *et al* (2016) PAF-Wnt signaling-induced cell plasticity is required for maintenance of breast cancer cell stemness. *Nature communications* 7: 10633

Wei M, Zhang Y, Yang X, Ma P, Li Y, Wu Y, Chen X, Deng X, Yang T, Mao X *et al* (2021) Claudin-2 promotes colorectal cancer growth and metastasis by suppressing NDRG1 transcription. *Clinical and translational medicine* 11: e667

Xiong J, Kawagishi H, Yan Y, Liu J, Wells QS, Edmunds LR, Fergusson MM, Yu ZX, Rovira, II, Brittain EL *et al* (2018) A Metabolic Basis for Endothelial-to-Mesenchymal Transition. *Molecular cell* 69: 689-698.e687

Yang C, Wang T, Zhu S, Zong Z, Luo C, Zhao Y, Liu J, Li T, Liu X, Liu C *et al* (2022) Nicotinamide N-Methyltransferase Remodeled Cell Metabolism and Aggravated Proinflammatory Responses by Activating STAT3/IL1β/PGE(2) Pathway. *ACS omega* 7: 37509-37519

Zizzari IG, Di Filippo A, Scirocchi F, Di Pietro FR, Rahimi H, Ugolini A, Scagnoli S, Vernocchi P, Del Chierico F, Putignani L *et al* (2020) Soluble Immune Checkpoints, Gut Metabolites and Performance Status as Parameters of Response to Nivolumab Treatment in NSCLC Patients. *Journal of personalized medicine* 10

31st Aug 2023

Dear Dr. Ceppi,

Thank you for the submission of your revised manuscript to EMBO Molecular Medicine, and please accept my apologies for the delay in getting back to you as the referees reports came back while I was out of the office. We have now received the reports from the 2 referees who re-reviewed your manuscript. As you will see, they are supportive of publication, and we will therefore be able to accept your manuscript once the following editorial points will be addressed:

1/ Referees: please address the remaining minor point from referee #2.

2/ Main manuscript file:
- Please address the queries from our data editors in the related Data Edited manuscript file. Remove the yellow highlights in the text and only keep in track changes mode any new modification.
- We noted a minor discrepancy in an author's name: Nils Joakim Færgeman in the manuscript vs. Nils Færgeman in the submission system.
- The manuscript details on p. 2-3 should be removed from the manuscript
- Materials and methods:
o Mice: please indicate the gender and age of the mice at the time of experiment.
o Statistics: please include information on blinding, randomization, inclusion/exclusion criteria and adjust the checklist accordingly.
- Data Availability: Please place this section after the Materials and Methods, before the Acknowledgements.
- Acknowledgements: All funding sources should be added to the submission system, and match the information provided in the manuscript (currently missing in the submission system: Interdisciplinary Center for Clinical Research of the University of Erlangen-Nuremberg).
- Please remove the Abbreviations from p. 53 (we do not have abbreviations list; abbreviations should be defined in the text the first time they are used).

3/ Figures:
- Please clearly mention in the legend of Figure EV2K that the image is re-used from Figure 5E.
- Please make sure that all figures and figure panels are referenced in the manuscript text. Currently, callouts are missing for Fig. 1G and 1H.

4/ The Paper Explained:
I introduced minor modifications in your text, please let me know if you agree with the following or amend as you see fit:
Problem
Epithelial-to-mesenchymal transition (EMT), a phenotypic developmental process, contributes to various highly aggressive features in cancer, including metastasis and chemoresistance with poor patient survival. However, pharmacological targeting of EMT in malignancies has proven very challenging. In recent years, targeting deregulated metabolic processes in cancer emerged as a realistic therapeutic strategy, though identification of precise EMT metabolic processes remains difficult.
Results
Taking advantage of hundreds of thousands of non-small cell lung cancer (NSCLC) patient-derived gene expression profiles and of numerous metabolic processes gene-sets, we performed an EMT-focused functional genomic analysis and identified negative association between EMT and short-chain fatty acids, especially propionate. Strikingly, treatment of lung cancer cell lines with propionate reinforced the epithelial transcriptional program, promoting cell-cell contact features along with the inhibition of aggressive EMT process, and sensitized the cells to cisplatin treatment. In addition, propionate reduced metastasis ability in a lung experimental metastasis model and limited lymph node metastatic spread in a genetic NSCLC mouse model. Finally, propionate was involved in chromatin remodeling with increased histone acetylation.
Impact
In this study, we identify in pre-clinical models the therapeutic relevance of the short-chain fatty acid propionate in reducing EMT-induced aggressive features of lung cancer.

5/ Synopsis:
- Thank you for providing a nice synopsis image. Please make sure that the width is 550 pixels (currently 548px) and that the text is legible.
- I added minor modifications to your text, let me know if you agree or amend as you see fit:
An EMT-centric investigation of metabolic processes in a comprehensive lung cancer transcriptome profiles collection identified an inverse association between EMT and SCFAs propionate and butyrate.
• Propionate enhances the epithelial features in vitro both at the molecular and cellular levels
• Pre-treatment of cells with propionate inhibits EMT associated processes and sensitizes the cells to the chemotherapeutic drug cisplatin

• Oral administration of propionate inhibits EMT-mediated lung colonization ability of NSCLC cells, and lymph node metastasis in a genetic mouse NSCLC model
• Molecular mechanistic investigation revealed chromatin remodelling through p300-mediated histone acetylation in E-cadherin gene regulation along with epithelial features reinforcement

6/ As part of the EMBO Publications transparent editorial process initiative (see our Editorial at http://embomolmed.embopress.org/content/2/9/329), EMBO Molecular Medicine will publish online a Review Process File (RPF) to accompany accepted manuscripts.
This file will be published in conjunction with your paper and will include the anonymous referee reports, your point-by-point response and all pertinent correspondence relating to the manuscript. Let us know whether you agree with the publication of the RPF and as here, if you want to remove or not any figures from it prior to publication.
Please note that the Authors checklist will be published at the end of the RPF.

I look forward to receiving your revised manuscript.

Yours sincerely,

Lise Roth

Lise Roth, PhD
Senior Editor
EMBO Molecular Medicine

***** Reviewer's comments *****

Referee #2 (Remarks for Author):

This completed version answers most points satisfactorily. Some new results bring up new questions, that will be ground for future works hopefully. One minor remark is that the new Fig R9 could be improved with higher contract pics to show better the crown of spreading and migrating cells in the 3D gel and on the 2D collagen substrate. The Fig R9B methodology helps to solve the difficulty of a true 3D setting, but the cell migration pattern is very similar to the mode used in wound healing assay. Based on these two pictures, it actually appears that the SP treated cells express less scattering and full cell-cell dissociation than the control cells in both situations. Because of the poor focus, it is difficult to assume, only based on the figure. This relevant data could probably be quantitated using relevant microscopy methods and/or image processing.

Referee #3 (Remarks for Author):

The manuscript is significantly improved I have no more comments.

<u>Response to the Reviewers Comments</u>:

We thank the Editor and Reviewers for the final positive comments and their important suggestions. We have now edited the manuscript as per the indications. We appreciate the possibility of publishing with EMBO Mol Med.

Referee #2 (Remarks for Author):

This completed version answers most points satisfactorily. Some new results bring up new questions, that will be ground for future works hopefully. One minor remark is that the new Fig R9 could be improved with higher contract pics to show better the crown of spreading and migrating cells in the 3D gel and on the 2D collagen substrate. The Fig R9B methodology helps to solve the difficulty of a true 3D setting, but the cell migration pattern is very similar to the mode used in wound healing assay. Based on these two pictures, it actually appears that the SP treated cells express less scattering and full cell-cell dissociation than the control cells in both situations. Because of the poor focus, it is difficult to assume, only based on the figure. This relevant data could probably be quantitated using relevant microscopy methods and/or image processing.

Response R2.1 Thanks for the positive comments and suggestions. As suggested by the Reviewer, we increased the contrast of the images and thereby quantified the migrating cells in both collagen settings (3D and 2D) for the spheroid assay. Although, there is a decreasing trend in the 3D collagen with no significance, we observed a significant decrease in the migrating pattern in the cells of the spheroids treated with SP (5 mM) for 72 hours. This implies that the effect is significant when the spheroids are grown on the 2D collagen substrate. And as rightly pointed out by the reviewer, the SP treated cells expressed less scattering with enhanced cell-cell contact in reducing the migratory ability of the cells compared to the control. However, as we already mentioned in the previous rebuttal letter, these results are probably could be limited by drug diffusion problems, which are encountered in collagen, but not in Matrigel 3D cultures. Since we are not 100% sure of this (and this is well beyond the scope of the paper) we would prefer to only add the Matrigel spheroid cultures experiment with and without TGF-beta in the paper (in **Fig EV1M**), and not include these collagen-based spheroid assays in the main manuscript. Thanks for the relevant comment.

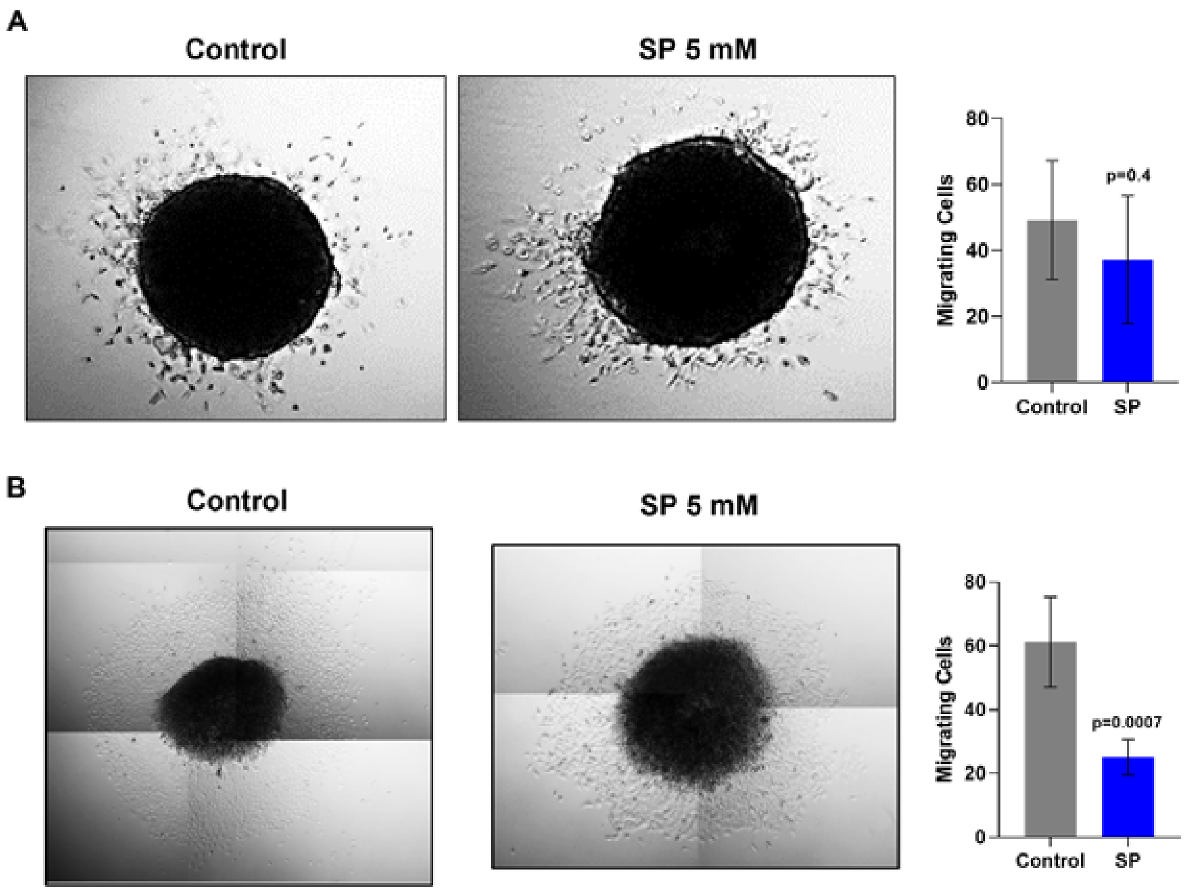

Spheroid assay in A549 cells treated with sodium propionate for 3 days. A. Left panel, Images representing the A549 spheroids inserted into 3D collagen gels treated with sodium propionate (5 mM) for 3 days (n=5). Right panel, Quantification of migratory pattern of A549 spheroids by anchoring to the collagen type I treated with SP (5 mM). B. Left panel, Images representing the A549 spheroids anchored to the collagen type I (n=5) treated with SP (5 mM). Right panel, Quantification of migratory pattern of A549 spheroids by anchoring to the collagen type I treated with SP (5 mM).

Referee #3 (Remarks for Author):

The manuscript is significantly improved I have no more comments.

Response R3.1 We thank the reviewer for the positive comment on our manuscript.

7th Sep 2023

Dear Dr. Ceppi,

Thank you for submitting your revised files. I am pleased to inform you that your manuscript is accepted for publication and is now being sent to our publisher to be included in the next available issue of EMBO Molecular Medicine!

Please read below for additional IMPORTANT information regarding your article, its publication and the production process.

Congratulations on your interesting work,

With kind regards,

Lise Roth

Lise Roth, Ph.D
Senior Editor
EMBO Molecular Medicine

Follow us on Twitter @EmboMolMed
Sign up for eTOCs at embopress.org/alertsfeeds

*** *** *** IMPORTANT INFORMATION *** *** ***

SPEED OF PUBLICATION
The journal aims for rapid publication of papers, using using the advance online publication "Early View" to expedite the process: A properly copy-edited and formatted version will be published as "Early View" after the proofs have been corrected. Please help the Editors and publisher avoid delays by providing e-mail address(es), telephone and fax numbers at which author(s) can be contacted.

Should you be planning a Press Release on your article, please get in contact with embomolmed@wiley.com as early as possible, in order to coordinate publication and release dates.

LICENSE AND PAYMENT:

All articles published in EMBO Molecular Medicine are fully open access: immediately and freely available to read, download and share.

EMBO Molecular Medicine charges an article processing charge (APC) to cover the publication costs. You, as the corresponding author for this manuscript, should have already received a quote with the article processing fee separately. Please let us know in case this quote has not been received.

Once your article is at Wiley for editorial production you will receive an email from Wiley's Author Services system, which will ask you to log in and will present you with the publication license form for completion. Within the same system the publication fee can be paid by credit card, an invoice, pro forma invoice or purchase order can be requested.

Payment of the publication charge and the signed Open Access Agreement form must be received before the article can be published online.

PROOFS

You will receive the proofs by e-mail approximately 2 weeks after all relevant files have been sent o our Production Office. Please return them within 48 hours and if there should be any problems, please contact the production office at

embopressproduction@wiley.com.

Please inform us if there is likely to be any difficulty in reaching you at the above address at that time. Failure to meet our deadlines may result in a delay of publication.

All further communications concerning your paper proofs should quote reference number EMM-2023-17836-V3 and be directed to the production office at embopressproduction@wiley.com.

EMBO Press Author Checklist

Corresponding Author Name: Paolo Ceppi
Journal Submitted to: EMBO Molecular Medicine
Manuscript Number: EMM-2023-17836

USEFUL LINKS FOR COMPLETING THIS FORM
The EMBO Journal - Author Guidelines
EMBO Reports - Author Guidelines
Molecular Systems Biology - Author Guidelines
EMBO Molecular Medicine - Author Guidelines

Reporting Checklist for Life Science Articles (updated January
This checklist is adapted from Materials Design Analysis Reporting (MDAR) Checklist for Authors. MDAR establishes a minimum set of requirements in transparent reporting in the life sciences (see Statement of Task: 10.31222/osf.io/9sm4x). Please follow the journal's guidelines in preparing your
Please note that a copy of this checklist will be published alongside your article.

Abridged guidelines for figures
1. Data
The data shown in figures should satisfy the following conditions:
- the data were obtained and processed according to the field's best practice and are presented to reflect the results of the experiments in an accurate and unbiased manner.
- ideally, figure panels should include only measurements that are directly comparable to each other and obtained with the same assay.
- plots include clearly labeled error bars for independent experiments and sample sizes. Unless justified, error bars should not be shown for technical
- if n<5, the individual data points from each experiment should be plotted. Any statistical test employed should be justified.
- Source Data should be included to report the data underlying figures according to the guidelines set out in the authorship guidelines on Data

2. Captions
Each figure caption should contain the following information, for each panel where they are relevant:
- a specification of the experimental system investigated (eg cell line, species name).
- the assay(s) and method(s) used to carry out the reported observations and measurements.
- an explicit mention of the biological and chemical entity(ies) that are being measured.
- an explicit mention of the biological and chemical entity(ies) that are altered/varied/perturbed in a controlled manner.
- the exact sample size (n) for each experimental group/condition, given as a number, not a range;
- a description of the sample collection allowing the reader to understand whether the samples represent technical or biological replicates (including how many animals, litters, cultures, etc.).
- a statement of how many times the experiment shown was independently replicated in the laboratory.
- definitions of statistical methods and measures:
  - common tests, such as t-test (please specify whether paired vs. unpaired), simple χ2 tests, Wilcoxon and Mann-Whitney tests, can be unambiguously identified by name only, but more complex techniques should be described in the methods section;
  - are tests one-sided or two-sided?
  - are there adjustments for multiple comparisons?
  - exact statistical test results, e.g., P values = x but not P values < x;
  - definition of 'center values' as median or average;
  - definition of error bars as s.d. or s.e.m.

Please complete ALL of the questions below.
Select "Not Applicable" only when the requested information is not relevant for your study.

Materials

Newly Created Materials	Information included in the manuscript?	In which section is the information available? (Reagents and Tools Table, Materials and Methods, Figures, Data Availability Section)
New materials and reagents need to be available; do any restrictions apply?	Not Applicable	

Antibodies	Information included in the manuscript?	In which section is the information available? (Reagents and Tools Table, Materials and Methods, Figures, Data Availability Section)
For **antibodies** provide the following information:<br>- Commercial antibodies: RRID (if possible) or supplier name, catalogue number and or/clone number<br>- Non-commercial: RRID or citation	Yes	Materials and Methods

DNA and RNA sequences	Information included in the manuscript?	In which section is the information available? (Reagents and Tools Table, Materials and Methods, Figures, Data Availability Section)
**Short novel DNA or RNA including primers, probes**: provide the sequences.	Not Applicable	

Cell materials	Information included in the manuscript?	In which section is the information available? (Reagents and Tools Table, Materials and Methods, Figures, Data Availability Section)
**Cell lines:** Provide species information, strain. Provide accession number in repository **OR** supplier name, catalog number, clone number, and/**OR** RRID.	Yes	Materials and Methods
**Primary cultures:** Provide species, strain, sex of origin, genetic modification status.	Yes	Materials and Methods
Report if the cell lines were recently **authenticated** (e.g., by STR profiling) and tested for mycoplasma contamination.	Yes	Materials and Methods

Experimental animals	Information included in the manuscript?	In which section is the information available? (Reagents and Tools Table, Materials and Methods, Figures, Data Availability Section)
**Laboratory animals or Model organisms:** Provide species, strain, sex, age, genetic modification status. Provide accession number in repository **OR** supplier name, catalog number, clone number, **OR** RRID.	Yes	Materials and Methods
**Animal observed in or captured from the field:** Provide species, sex, and age where possible.	Not Applicable	
Please detail **housing and husbandry conditions**.	Yes	Materials and Methods

Plants and microbes	Information included in the manuscript?	In which section is the information available? (Reagents and Tools Table, Materials and Methods, Figures, Data Availability Section)
**Plants:** provide species and strain, ecotype and cultivar where relevant, unique accession number if available, and source (including location for collected wild specimens).	Not Applicable	
**Microbes:** provide species and strain, unique accession number if available, and source.	Not Applicable	

Human research participants	Information included in the manuscript?	In which section is the information available? (Reagents and Tools Table, Materials and Methods, Figures, Data Availability Section)
If collected and within the bounds of privacy constraints report on age, sex and gender or ethnicity for all study participants.	Not Applicable	

Core facilities	Information included in the manuscript?	In which section is the information available? (Reagents and Tools Table, Materials and Methods, Figures, Data Availability Section)
If your work benefited from core facilities, was their service mentioned in the acknowledgments section?	Yes	Acknowledgement

Design

**Study protocol**	**Information included in the manuscript?**	**In which section is the information available?** (Reagents and Tools Table, Materials and Methods, Figures, Data Availability Section)
If study protocol has been **pre-registered, provide DOI in the manuscript**. For clinical trials, provide the trial registration number **OR** cite DOI.	Not Applicable	
Report the **clinical trial registration number** (at ClinicalTrials.gov or equivalent), where applicable.	Not Applicable	

**Laboratory protocol**	**Information included in the manuscript?**	**In which section is the information available?** (Reagents and Tools Table, Materials and Methods, Figures, Data Availability Section)
Provide DOI OR other citation details if **external detailed step-by-step protocols** are available.	Not Applicable	

**Experimental study design and statistics**	**Information included in the manuscript?**	**In which section is the information available?** (Reagents and Tools Table, Materials and Methods, Figures, Data Availability Section)
Include a statement about **sample size** estimate even if no statistical methods were used.	Yes	Figures and Figure legends
Were any steps taken to minimize the effects of subjective bias when allocating animals/samples to treatment (e.g. **randomization procedure**)? If yes, have they been described?	Not Applicable	
Include a statement about **blinding** even if no blinding was done.	Yes	Materials and Methods; Statistical analysis
Describe **inclusion/exclusion criteria** if samples or animals were excluded from the analysis. Were the criteria pre-established?<br><br>If sample or data points were omitted from analysis, report if this was due to attrition or intentional exclusion and provide justification.	Yes	Materials and Methods; Statistical analysis
For every figure, are **statistical tests** justified as appropriate? Do the data meet the assumptions of the tests (e.g., normal distribution)? Describe any methods used to assess it. Is there an estimate of variation within each group of data? Is the variance similar between the groups that are being statistically compared?	Yes	Figures legends

**Sample definition and in-laboratory replication**	**Information included in the manuscript?**	**In which section is the information available?** (Reagents and Tools Table, Materials and Methods, Figures, Data Availability Section)
In the figure legends: state number of times the experiment was **replicated** in laboratory.	Yes	Figure legends
In the figure legends: define whether data describe **technical or biological replicates**.	Yes	Figure legends

Ethics

**Ethics**	**Information included in the manuscript?**	**In which section is the information available?** (Reagents and Tools Table, Materials and Methods, Figures, Data Availability Section)
Studies involving **human participants**: State details of **authority granting ethics approval** (IRB or equivalent committee(s), provide reference number for approval.	Not Applicable	
Studies involving **human participants**: Include a statement confirming that **informed consent** was obtained from all subjects and that the experiments conformed to the principles set out in the WMA Declaration of Helsinki and the Department of Health and Human Services Belmont Report.	Not Applicable	
Studies involving **human participants: For publication of patient photos**, include a statement confirming that consent to publish was obtained.	Not Applicable	
Studies involving experimental **animals**: State details of **authority granting ethics approval** (IRB or equivalent committee(s), provide reference number for approval. Include a statement of compliance with ethical regulations.	Yes	Materials and Methods
Studies involving **specimen and field samples:** State if relevant **permits** obtained, provide details of authority approving study; if none were required, explain why.	Not Applicable	

**Dual Use Research of Concern (DURC)**	**Information included in the manuscript?**	**In which section is the information available?** (Reagents and Tools Table, Materials and Methods, Figures, Data Availability Section)
Could your study fall under dual use research restrictions? Please check biosecurity documents and list of **select agents and toxins** (CDC): https://www.selectagents.gov/sat/list.htm	Not Applicable	
If you used a select agent, is the security level of the lab appropriate and reported in the manuscript?	Not Applicable	
If a study is subject to dual use research of concern regulations, is the name of the **authority granting approval and reference number** for the regulatory approval provided in the manuscript?	Not Applicable	

Reporting

The MDAR framework recommends adoption of discipline-specific guidelines, established and endorsed through community initiatives. Journals have their own policy about requiring specific guidelines and recommendations to complement MDAR.

**Adherence to community standards**	**Information included in the manuscript?**	**In which section is the information available?** (Reagents and Tools Table, Materials and Methods, Figures, Data Availability Section)
State if relevant guidelines or checklists (e.g., **ICMJE, MIBBI, ARRIVE, PRISMA**) have been followed or provided.	Not Applicable	
For **tumor marker prognostic studies,** we recommend that you follow the **REMARK** reporting guidelines (see link list at top right). See author guidelines, under 'Reporting Guidelines'. Please confirm you have followed these guidelines.	Not Applicable	
For **phase II and III randomized controlled trials**, please refer to the **CONSORT** flow diagram (see link list at top right) and submit the CONSORT checklist (see link list at top right) with your submission. See author guidelines, under 'Reporting Guidelines'. Please confirm you have submitted this list.	Not Applicable	

Data Availability

**Data availability**	**Information included in the manuscript?**	**In which section is the information available?** (Reagents and Tools Table, Materials and Methods, Figures, Data Availability Section)
Have **primary datasets** been deposited according to the journal's guidelines (see 'Data Deposition' section) and the respective accession numbers provided in the Data Availability Section?	Yes	Data Availability
Were **human clinical and genomic datasets** deposited in a public access-controlled repository in accordance to ethical obligations to the patients and to the applicable consent agreement?	Not Applicable	
Are **computational models** that are central and integral to a study available without restrictions in a machine-readable form? Were the relevant accession numbers or links provided?	Yes	Materials and Methods; Data availability
If publicly available data were reused, provide the respective **data citations in the reference list**.	Yes	Results and References
